# Supplementary material for: Amygdalostriatal transition zone neurons encode sustained cue responses to guide defensive behaviors
Source: Neuron. Author manuscript; Available in PMC 2026 Sep 21. (PMC13592531; doi:10.1016/j.neuron.2026.08.012)
Supplement: Supplementary file 1 [file NIHMS2206276-supplement-MMC1.pdf]

Neuron, Volume *115*

Supplemental information

Amygdalostriatal transition zone neurons encode sustained cue responses to guide defensive behaviors

Fergil Mills, Christopher R. Lee, James R. Howe, Hao Li, Maria N. Keisler, Shan Shao, Felix H. Taschbach, Mackenzie E. Lemieux, Faith Aloboudi, Jesse White, May G. Chan, Matilde Borio, Laurel R. Keyes, Hannah S. Chen, Fabiha Bushra, Gates P. Schneider, Dani P. Lemmon, Kyung J. Lee, Alexa L. Gross, Kanha Batra, Reesha R. Patel, Meenakshi M. Asokan, Jeremy Delahanty, Christian Cazares, Christopher R. Heyman, Nicholas B. Poll, Liezl Maree, Romy Wichmann, Talmo D. Pereira, Marcus K. Benna, Cory M. Root, and Kay M. Tye

Amygdalostriatal transition zone neurons encode sustained cue responses to guide defensive behaviors

SUPPLEMENTARY FIGURES

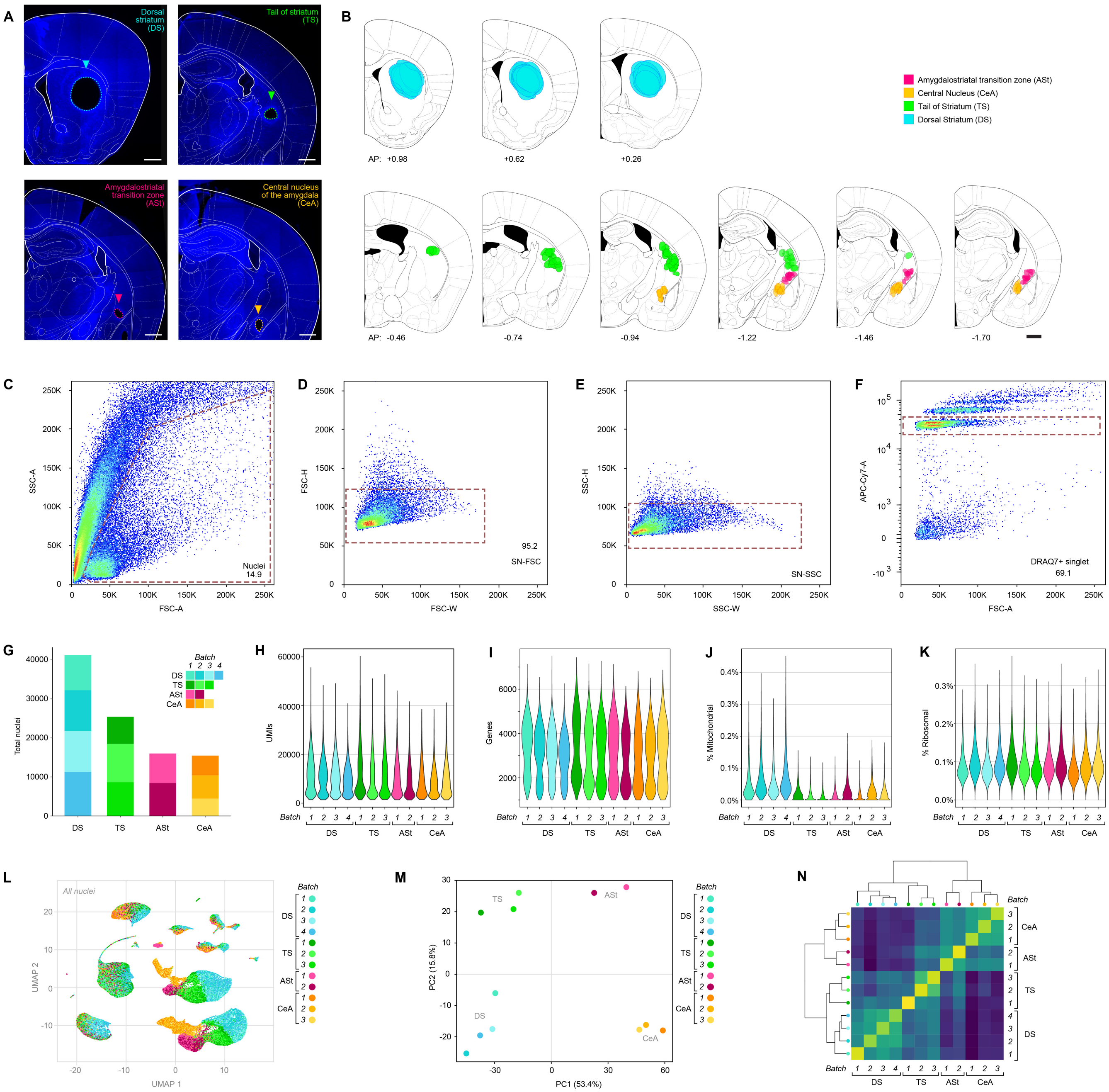

Figure S1. Quality control metrics for single-nucleus RNA sequencing

(**A**) Representative images of tissue microdissection sites from RNA sequencing target regions, the dorsal striatum (DS), tail of striatum (TS), amygdalostriatal transition zone (ASt), and central nucleus of the amygdala (CeA) (Blue = DAPI, scale bars = 500 µm). (**B**) Location of all tissue sample sites used for single-nucleus RNA sequencing, color coded by region. AP = anteroposterior distance from bregma (mm), scale bar = 500 µm. (**C-F**) Density plots outlining the gating strategy for FANS isolation of single nuclei. Nuclei isolated from regions of interest were FANS sorted using 70 µm nozzle at 52 psi. Nuclei were sorted based on size (**C**), duplicates and/or morphology (**D, E**), and by high DRAQ7 signal, which stains DNA in nuclei (**F**). Single DRAQ7^{+} events at the lowest stoichiometric fluorescence multiple were considered nuclei. (**G**) Absolute number and proportion of nuclei passing quality control filters from each batch in each region. (**H**) UMIs detected per nucleus, filtered at the median per library + five times the median absolute deviation. (**I**) Genes detected per nucleus, filtered at minimum 1000 genes. (**J**) Percent mitochondrial reads per nucleus, filtered at median per library + five times the median absolute deviation. (**K**) Percent ribosomal reads per nucleus, no quality filter applied. (**L**) UMAP of all sequenced nuclei colored by both target region and batch identity. (**M**) PCA of pseudobulk samples created from each batch, colored by both target region and batch identity. (**N**) Evaluation of transcriptional homology on a per-batch basis, where the distance matrix is based on Spearman correlation between median expression of genes on a per-region basis, and the dendrogram was created via hierarchical clustering on this correlation matrix.

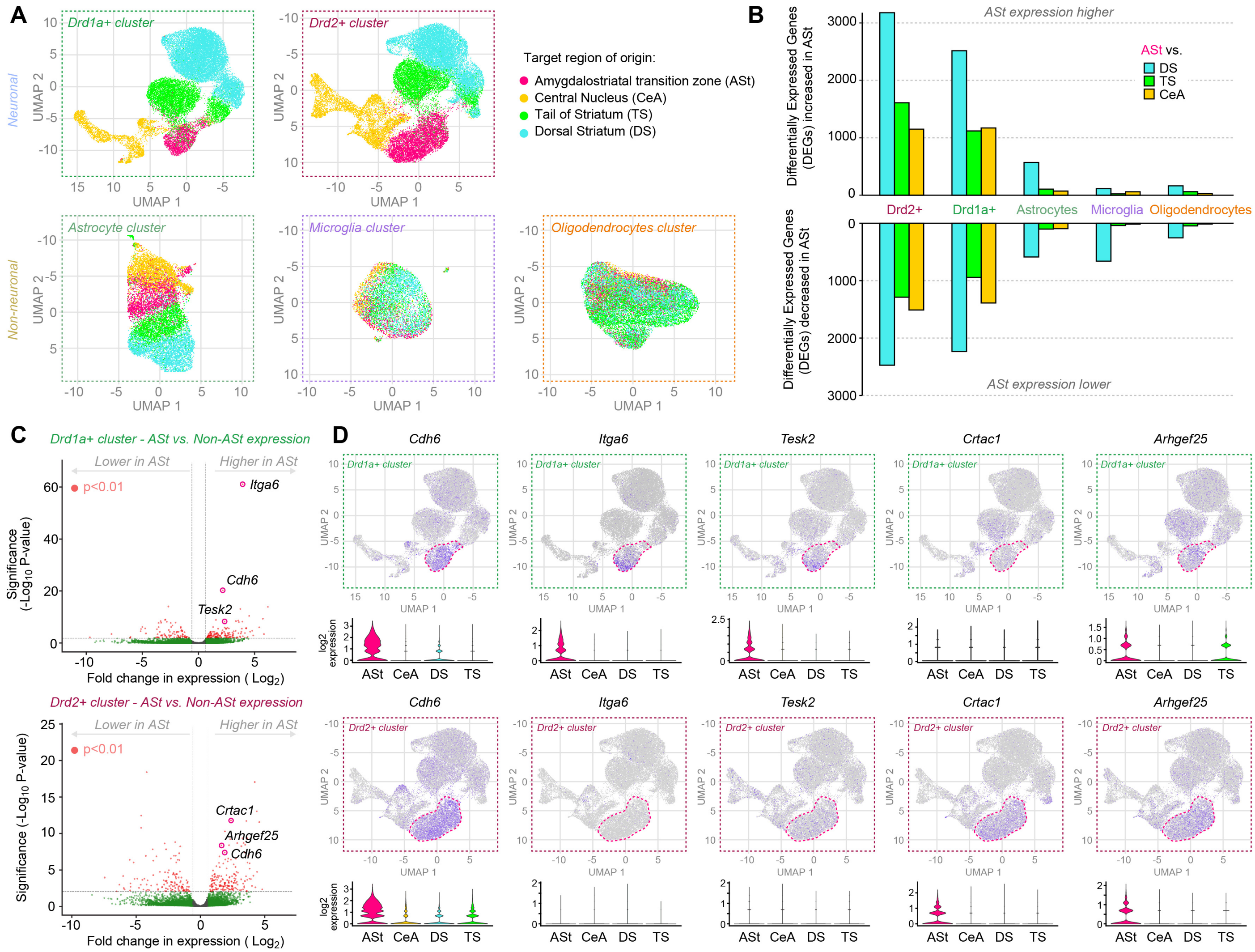

Figure S2. Single-nucleus RNA sequencing identifies unique transcriptomic signatures for ASt cells.

(**A**) UMAP projections of nuclei from the largest cell type clusters (*Drd1a*+, *Drd2*+, Astrocytes, Microglia and Oligodendrocytes) colored by tissue region of origin.
(**B**) Number of differentially expressed genes ($p < 0.01$, 1.5 log-fold change) with increased expression (top) or decreased expression (bottom) in ASt nuclei relative to nuclei from the central nucleus of the amygdala (CeA), tail of striatum (TS), and dorsal striatum (DS) within each cell type cluster.
(**C**) Volcano plot showing differentially expressed genes between ASt and non-ASt nuclei in *Drd1a*+ cluster (top) and *Drd2*+ cluster (bottom). Dashed lines indicate $p < 0.01$ and a fold-change of 1.5. Dots colored grey fail to meet either standard, dots colored green have sufficient fold change but not significance, while red dots meet both standards. Marker genes of interest for further analysis are circled.
(**D**) Expression of specific marker genes of interest in *Drd1a*+ cluster (top) and *Drd2*+ cluster (bottom) neurons. Expression is visualized via UMAP and violin plot. UMAP expression is colored based on increasing normalized expression intensity. Violin plots show smoothed expression density, colored and split based on regional identity within each cluster.

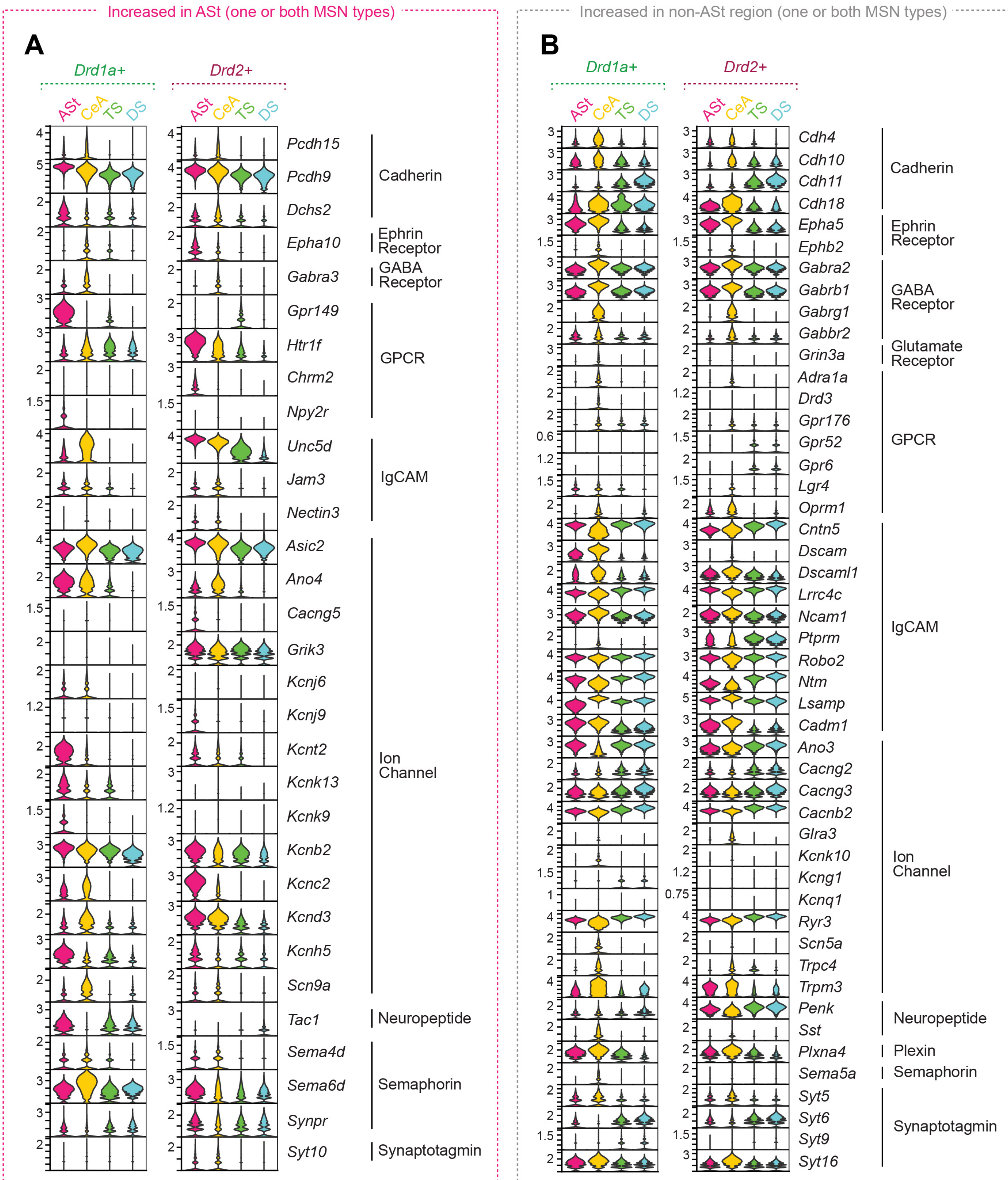

Figure S3. Differentially expressed genes of interest with neurologically-relevant function in striatal subregions of interest.

(**A**) Differentially expressed genes with increased expression in ASt falling into predefined HGNC gene families related to neurological function, increased in *Drd1a*+ and/or *Drd2*+ MSNs.
(**B**) Differentially expressed genes with increased expression in batched non-ASt regions (DS, TS, and/or CeA) falling into predefined HGNC gene families related to neurological function, increased in *Drd1a*+ and/or *Drd2*+ MSNs.

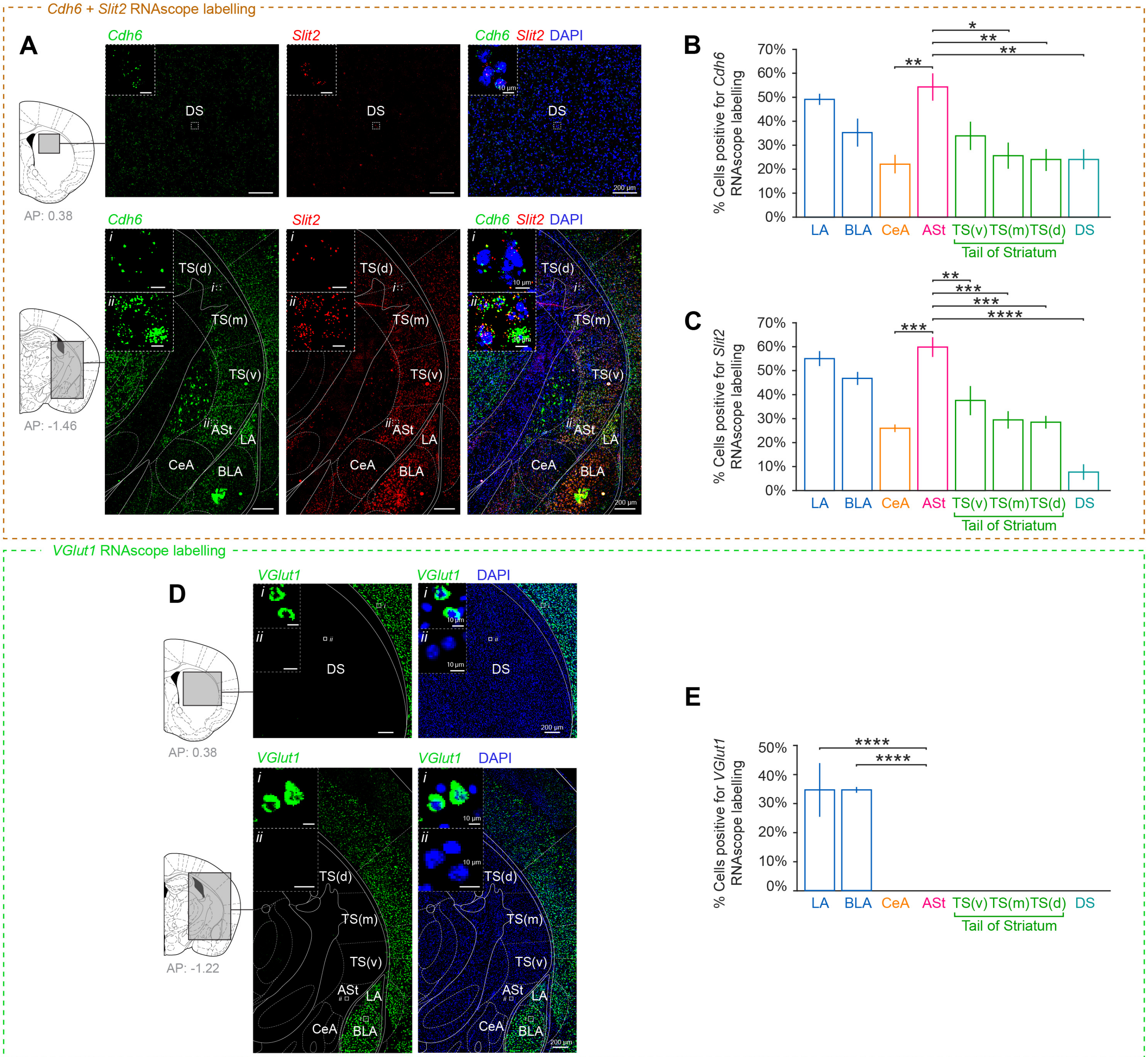

Figure S4. *Cdh6* and *Slit2* are enriched and VGlut1 is absent in the ASt

(**A**) Representative images of *in situ* RNAscope labeling of *Cdh6* RNA (green) and *Slit2* RNA (red) in striatal target regions. For analysis of dorsoventral expression gradients, the tail of striatum was spatially separated into three segments (TS(v), TS(m), TS(d)), each ~500 μm in length along the DV axis. (**B**) Relative proportion of cells in each target region positively labeled for *Cdh6* RNA. (n = 3 mice, One-way ANOVA, $F(7, 16) = 6.320$, $p = 0.0011$). (**C**) Relative proportion of cells in each target region positively labeled for *Slit2* RNA (n = 3 mice, One-way ANOVA, $F(7, 16) = 22.65$, $p = 3.6 \times 10^{-7}$). Consistent with our sequencing data, *Cdh6* and *Slit2* showed significantly greater expression in the ASt compared with more dorsal regions of the tail of striatum (TS). *Cdh6* and *Slit2* expression was also greater in the ASt than the CeA, while *Cdh6* and *Slit2* expression in the ASt and LA was similar, consistent with the LA and ASt receiving highly similar axonal inputs [25,37]. (**D**) Representative images of *in situ* RNAscope labeling of *VGlut1* RNA (green). *VGlut1* expression was not seen in the ASt, indicating the small number of *VGlut1*-expressing nuclei detected in ASt sequencing samples (see **Fig 1, E-G)** were attributable to small inclusions of tissue from adjacent regions with glutamatergic neurons.
(**E**) Relative proportion of cells in each target region positively labeled for *VGlut1* RNA. No *VGlut1*-expressing (excitatory) neurons were identified in the ASt, the CeA, or other striatal subregions. (n = 3 mice, One-way ANOVA, $F(7, 16) = 24.37$, $p = 2.1 \times 10^{-7}$). All data presented as mean ±SEM. Post-hoc comparisons between regions, Tukey's test, $*p < 0.05$, $**p < 0.01$, $***p < 0.001$, $****p < 0.0001$. For clarity, only significant pairwise comparisons with ASt are shown.

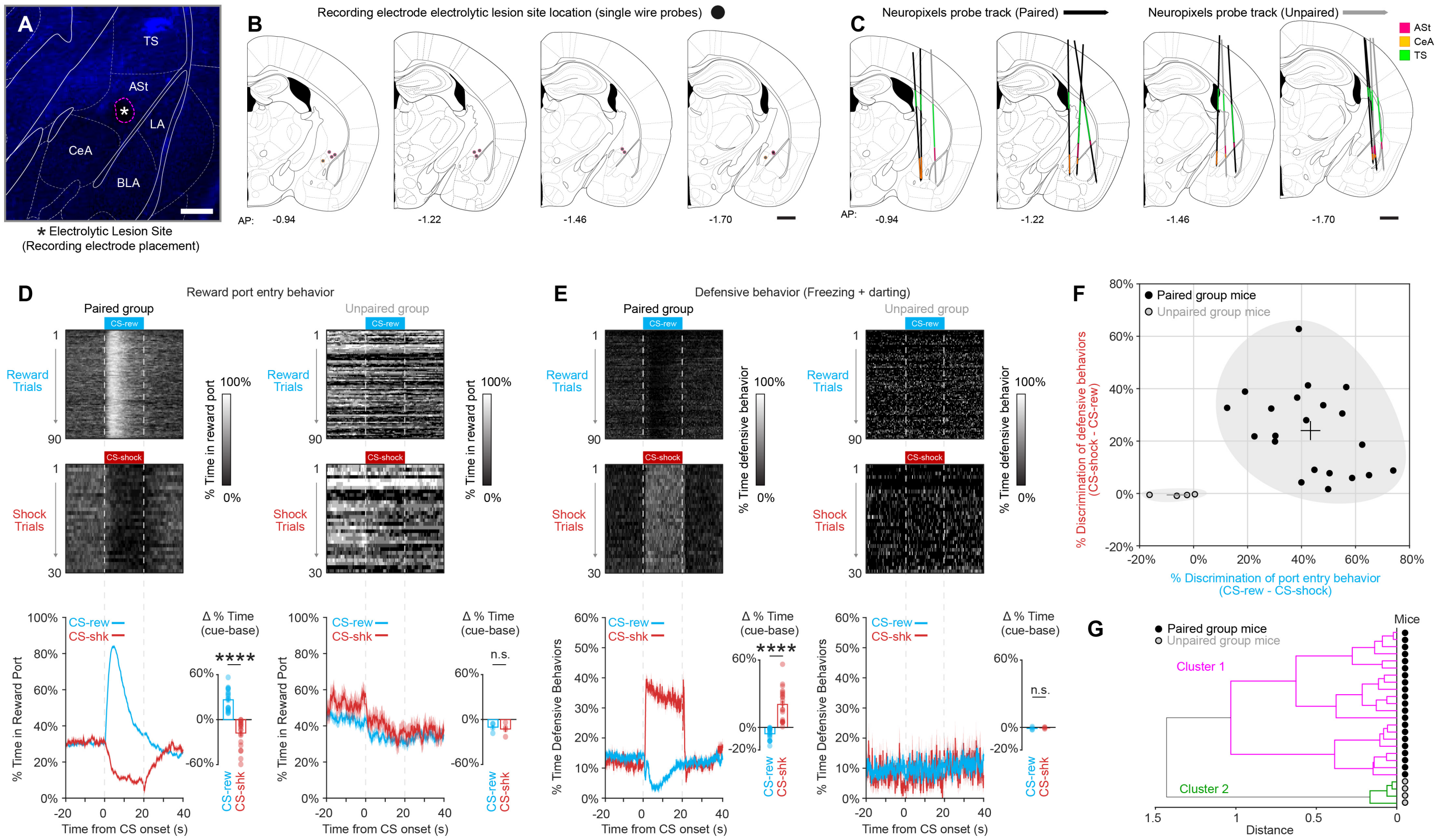

Figure S5. Histological targeting and behavioral validation for *in vivo* electrophysiology recordings.

(**A**) Representative image of electrolytic lesion site to confirm recording electrode targeting to the ASt (Blue = DAPI, Scale bar = 100 µm). (**B**) Histologically verified lesion sites of single-wire electrode placements in recording experiments. (**C**) Histologically verified probe tracks of Neuropixels probes in recording experiments. Colors indicate segment of probe that was in a target region analyzed (Pink = ASt, Green = TS, Orange = CeA). (**D-G**) Validation of conditioned behavioral responses during two-tone discrimination task in mice in electrophysiological recording experiments (Paired: n = 21 mice, Unpaired: n = 4 mice). Paired group mice showed distinct responses to the CS-shock and CS-reward tones in reward port entry behavior (Two-tailed paired t-test, $t_{20} = 12.469$, ****$p < 0.0001$) (**D**) and defensive responses (Two-tailed paired t-test, $t_{20} = 6.711$, ****$p < 0.0001$) (**E**), consistent with successful discrimination between the two tones. Neither measure differed between cue types in unpaired mice (port entry, $t_3 = 0.672$, $p = 0.550$; defensive responses, $t_3 = 0.658$, $p = 0.557$). (**F**) 'Discrimination scores' of reward port entry behavior (CS-rew − CS-shock) plotted against defensive behavior (CS-shock − CS-rew) for each mouse. Crosses indicate group mean. (**G**) Agglomerative hierarchical clustering of electrophysiology mice based solely on their defensive and reward behavior discrimination identified unpaired mice as a distinct cluster relative to all paired group mice.

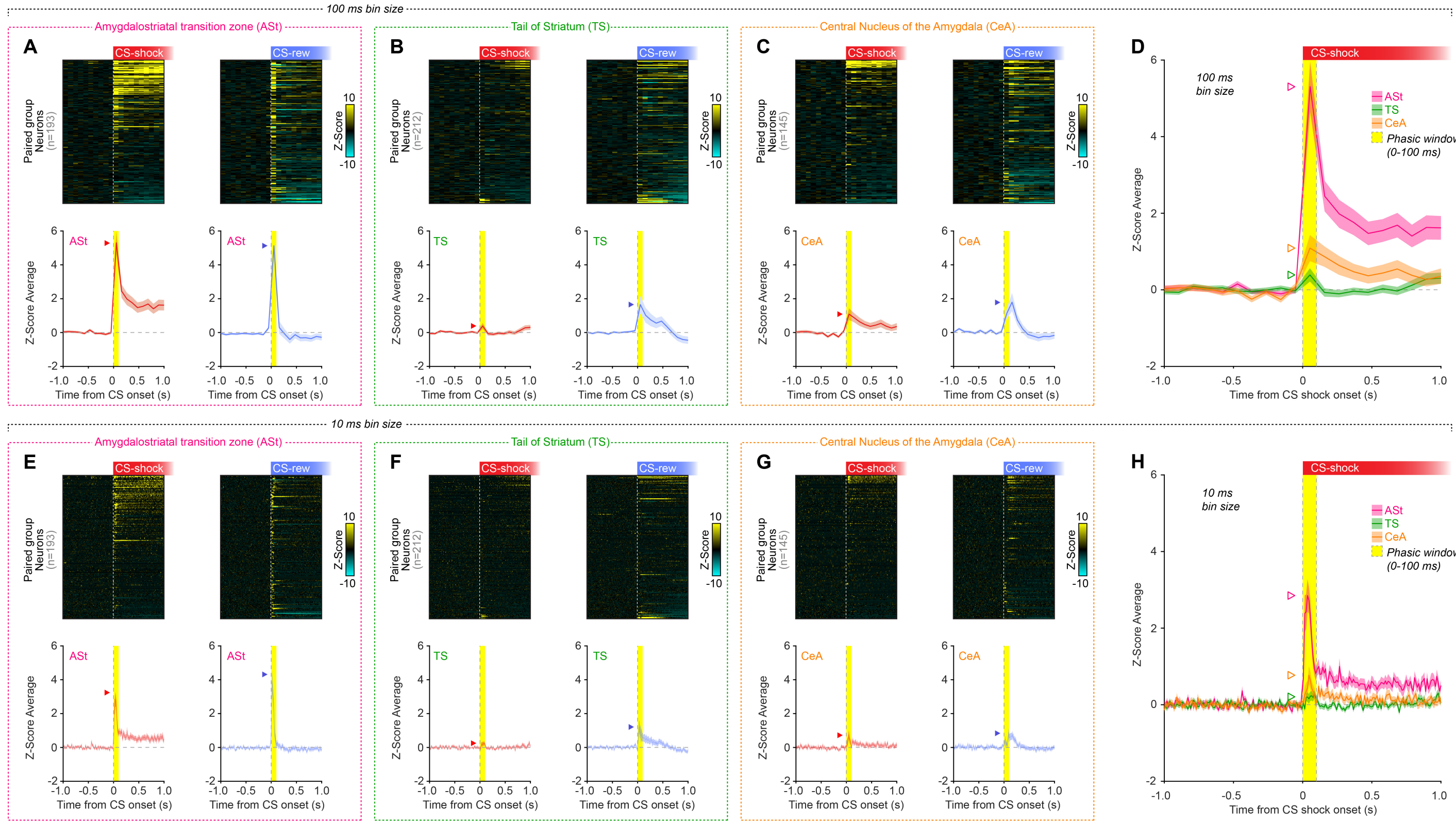

Figure S6. Phasic responses to CS-shock and CS-reward in the ASt, TS, and CeA.

(**A-C**) Heat maps and group average of z-score changes in neural firing rate in response to CS-shock and CS-reward in ASt (**A**), TS (**B**), and CeA (**C**). Data shown here are a zoomed-in visualization of data in Figure 2F,G,H. (**D**) Group average z-score changes in neural firing rate to CS-shock in ASt, TS, and CeA, plotted together for easier comparison. Heat maps and group average of z-score changes in neural firing rate, with 10 millisecond bins, in response to CS-shock and CS-reward in ASt (**E**), TS (**F**), and CeA (**G**). Data shown here are a zoomed-in visualization of data in Figure 2F,G,H. (**H**) Group average z-score changes in neural firing rate, with 10 millisecond bins, to CS-shock in ASt, TS, and CeA, plotted together for easier comparison. Heat map rows represent average z-score PSTH for individual neurons. Arrowheads show average peak z-score in the 'phasic' window (0-100 ms from CS onset). Data shown as mean ±SEM.

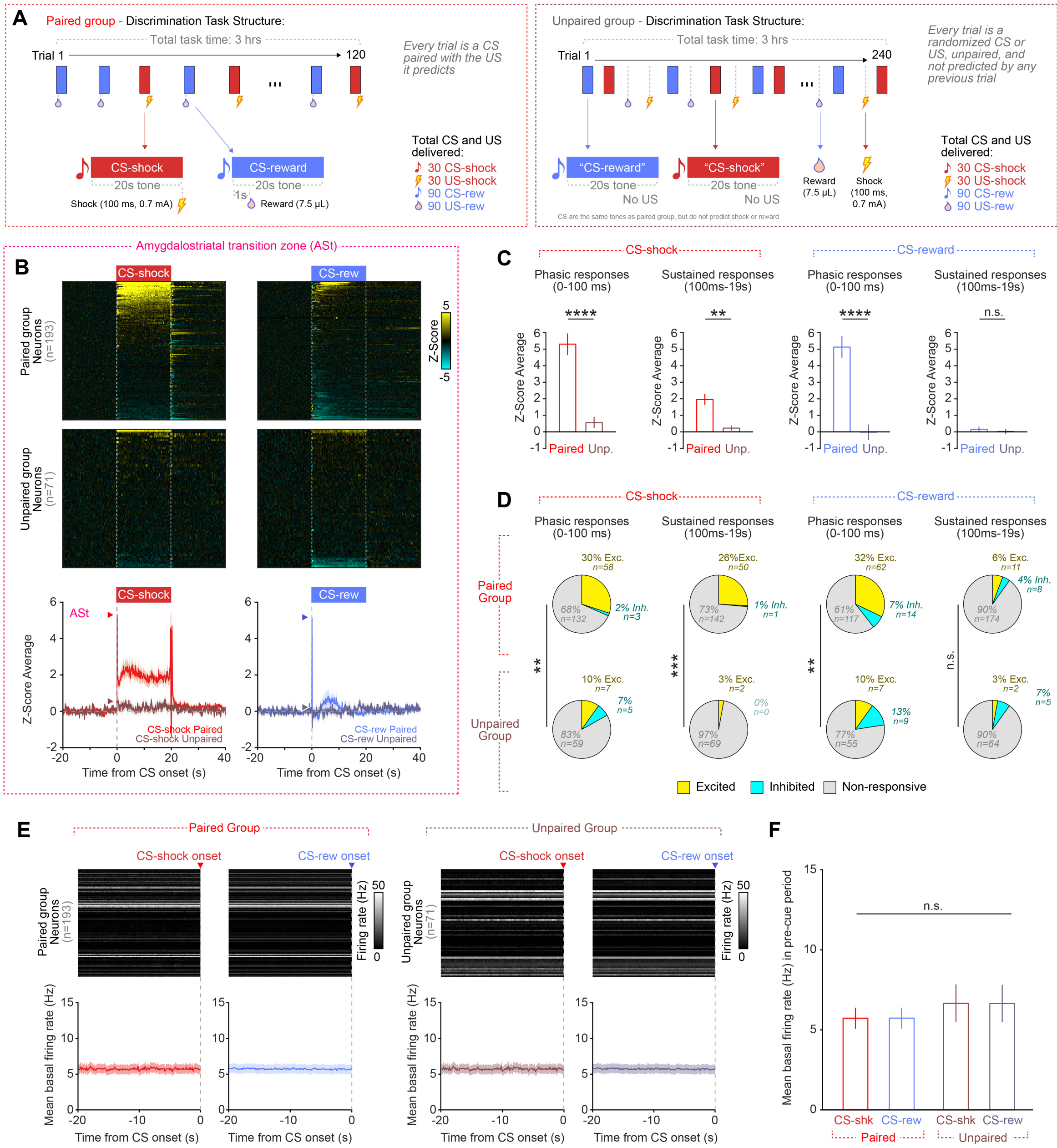

Figure S7. Differences in ASt neuron responses to stimuli in paired and unpaired controls.

(**A**) Design of two-tone discrimination task for paired and unpaired groups in *in vivo* electrophysiology recordings. (**B**) Heat maps and group average of z-score changes in neural firing rate in response to CS-shock and CS-reward in ASt paired and unpaired groups. Heatmap rows represent average z-score PSTH for individual neurons. Arrowheads show average peak z-score during phasic window (0-100 ms). (**C**) ASt neurons in paired group mice showed greater phasic responses to CS-shock (two-tailed t-test, t262 = 4.332, ****p < 0.0001), sustained responses to CS-shock (t262 = 3.109, **p = 0.0021), and phasic responses to CS-reward (t262 = 4.511, ****p < 0.0001) than unpaired controls. Sustained responses to CS-reward did not differ between groups (t262 = 0.460, p = 0.646). (**D**) Proportions of ASt neurons in the paired and unpaired groups that were significantly excited or inhibited by CS-shock or CS-reward during the phasic or sustained windows (chi-square test with Holm-Bonferroni correction; CS-shock phasic, $\chi2(2, N = 264) = 15.31$, **p = 0.0014; CS-shock sustained, $\chi2(2, N = 264) = 18.04$, ***p = 0.0005; CS-reward phasic, $\chi2(2, N = 264) = 13.86$, **p = 0.0020; CS-reward sustained, $\chi2(2, N = 264) = 1.76$, p = 0.415). **p < 0.01, ***p < 0.001. (**E**) Heat maps and group average of basal neural firing rates in the 20s window prior to CS onset, for paired and unpaired group ASt neurons. (**F**) No significant differences in basal firing rate were seen between paired and unpaired group ASt neurons, or between CS-shock and CS-reward windows (two-way mixed ANOVA, group [between-neuron] × cue window [within-neuron]: group main effect F(1,262)=0.51, p=0.476; cue main effect F(1,262)=0.01, p=0.926; group × cue interaction F(1,262)=0.12, p=0.728). Paired: n=15 mice, 193 neurons; Unpaired: n=4 mice, 71 neurons.

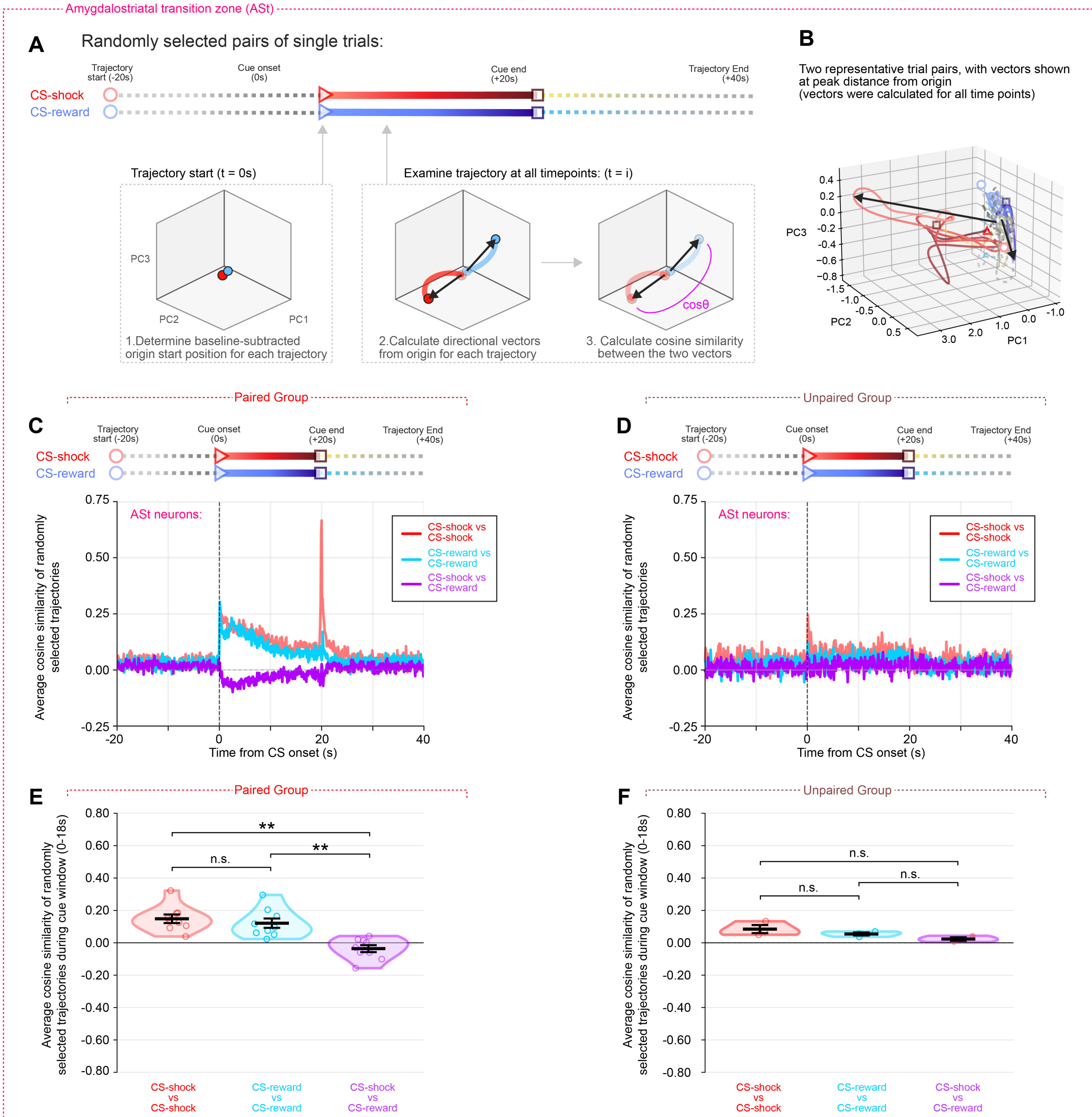

Figure S8. Single-trial population-trajectory analysis using randomly sampled trials demonstrates divergence of ASt population dynamics in CS-shock and CS-reward trials.

(**A**) Schematic of the trajectory-similarity method. Trial pairs were randomly selected, and average baseline (-20s to 0s) subtracted to determine normalized trajectory origins. For all subsequent timepoints, the instantaneous trajectory direction was calculated as a vector from the origin, and cosine similarity of the two trajectories calculated. (**B**) Example neural population trajectories for two representative CS-shock (red) and CS-reward (blue) trial pairs. For visualization, arrows indicate the trajectory direction at the time of peak distance from approximate baseline average origin. Similar calculations were performed at all timepoints across the full trajectory. (**C-D)** Average cosine similarity of randomly selected trajectory pairs across time in paired group (**C**) and unpaired group (**D**) mice. Same-CS trajectory pairs (CS-shock vs CS-shock; CS-reward vs CS-reward) show higher similarity following cue presentation, whereas opposite-CS trajectory pairs (CS-shock vs CS-reward) rapidly diverge in paired group, producing markedly lower similarity during and after the cue. In unpaired group mice, the negative divergence of CS-shock and CS-reward trial pairs was not seen. (**E-F**) Summary of mean cosine similarity in the cue window (excluding shock delivery at end of CS) in paired group (**E**) and unpaired group (**F**) mice. Paired animals show significantly reduced similarity between CS-shock and CS-reward trajectories reflecting the emergence of separable, CS-specific neural population dynamics in ASt (** $p < 0.01$ Wilcoxon signed-rank test). Data shown as mean ±SEM.

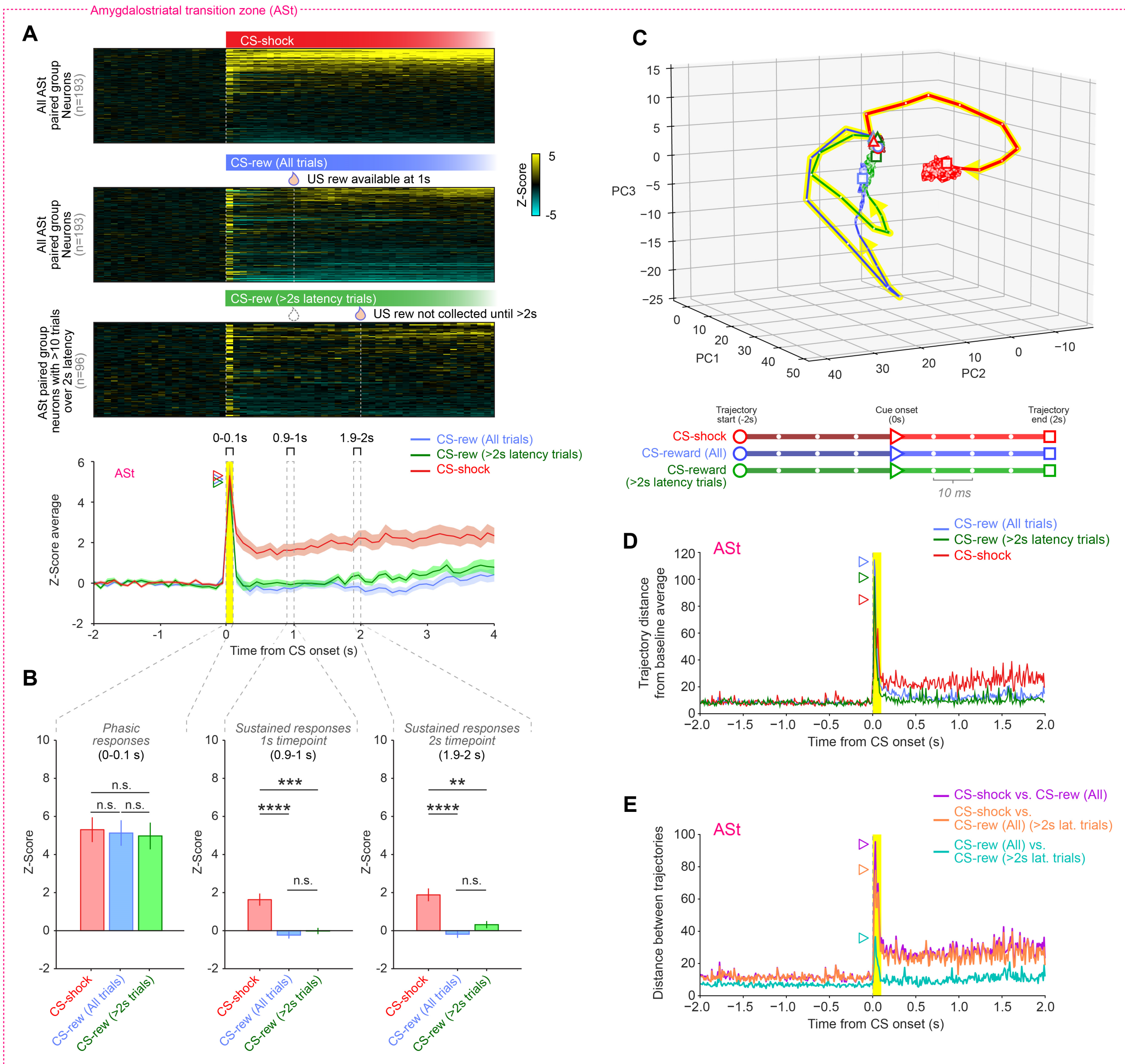

Figure S9. ASt neuron sustained activity is seen in response to CS-shock, but not CS-reward cues.

(**A**) Heat maps and group average of z-score changes in ASt neuron firing rate in response to CS-shock, CS-reward in ASt, and the subset of CS-reward trials in which the US reward was not collected until at least 2s (termed ">2s latency trials"). Arrowheads show average peak z-score during phasic window (0-100 ms). (**B**) Mean z-score responses of ASt neurons in response to CS-shock, CS-reward and CS-reward trials with >2s latency to reward collection during the phasic and sustained time windows. No significant difference in phasic responses (0-100 ms) were observed between groups. At the 1s timepoint and the 2s timepoint, sustained responses to CS-shock were present, but these were absent in the CS-reward as well as the CS-reward trials where the latency to US reward collection was >2s. Consequently, the sustained responses were only observed in response to CS-shock, and not CS-reward, including in the portion of CS-reward trials free of any US reward responses (One-way ANOVA: phasic, $F(2,479) = 0.05$, $p = 0.95$; sustained 1s timepoint, $F(2,479) = 17.38$, $p = 5.2 \times 10^{-8}$; sustained 2s timepoint, $F(2,479) = 17.40$, $p = 5.1 \times 10^{-8}$; followed by Tukey's multiple comparison test, **$p < 0.01$, ***$p < 0.001$, ****$p < 0.0001$). (**C**) Neural trajectories of neuron firing rates (10 ms bins) in the ASt for the -2s to 2s window around onset of CS-shock, CS-reward (All), and CS-reward >2s latency trials show that CS-shock trajectories move in a distinct direction from the CS-reward trajectories. Rearward-looking gaussian ($\sigma = 2.2$) smoothing with a 50 ms max distance was applied to each trajectory. (**D**) Euclidean distance of CS-shock, CS-reward (All), and CS-reward >2s trajectories from the baseline average position prior to the CS. (**E**) Pairwise Euclidean distance of CS-shock, CS-reward (All), and CS-reward >2s trajectories from each other. The two reward trials are both highly distant from CS-shock, with sharp divergence of the trajectories immediately after CS onset, which is incompatible with a 'salience' response. CS-shock and CS-reward: *n* = 15 mice, 193 neurons, CS-reward >2s latency trials: *n* = 9 mice, 96 neurons. Data presented as mean ±SEM

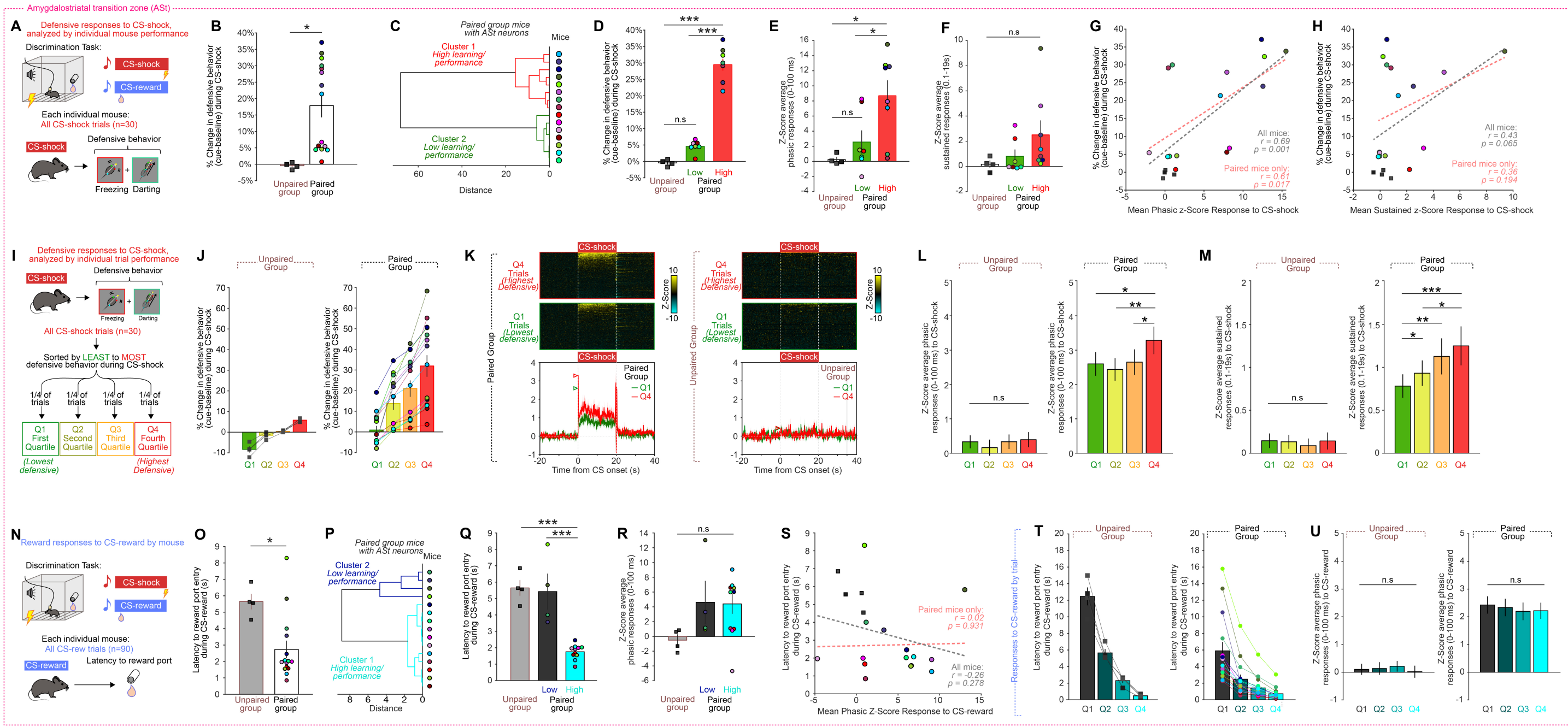

Figure S10. ASt neuron responses to CS-shock are correlated with increased defensive behavior across individual mice and trials.

(**A-H**) Analysis of ASt neuron responses to CS-shock and defensive behaviors in individual mice (**A**) Two-tone discrimination task with electrophysiology recordings. Defensive behavior was calculated as the sum of freezing and darting behavior during each CS-shock trial. (**B**) Group average and individual mouse defensive behaviors during CS-shock was higher in paired group mice than unpaired group mice (t_{17} = 2.671, p = 0.0161, Student's t-test. Paired group: n = 15 mice, Unpaired group: n = 4 mice). (**C**) Hierarchical clustering of paired group mice into "low" and "high" learner/performer clusters based on magnitude of defensive behavioral responses. (**D**) Group average defensive behaviors was significantly higher in "high" performance mice than "low" performance and unpaired mice. (One-way ANOVA, $F_{(2,16)}$ = 130.6, p < 0.0001. ***p < 0.001, Tukey's multiple comparison test. For panels D-F, Unpaired Group: n = 4 mice, "Low" paired group: n = 7 mice, "High" paired group: n = 8 mice). (**E**) Average ASt neuron phasic responses to CS-shock were significantly greater in the "high" paired group than the "low" group or unpaired controls (One-way ANOVA, $F_{(2,16)}$ = 6.023, p = 0.011. *p < 0.05, Tukey's multiple comparison test). (**F**) Average ASt neuron sustained responses to CS-shock were over 3-fold greater in the "high" paired group than the "low" group, though not significant due to greater variability in sampling of sustained-responding neurons in individual mice (One-way ANOVA, $F_{(2,16)}$ = 1.793, p = 0.198). (**G-H**) Average ASt neuron phasic responses (**G**) and sustained responses (**H**) were broadly correlated with increased defensive behavioral responses to CS-shock. (**I-M**) Analysis of ASt neuron activity within mice, across trials with low or high defensive behaviors. (**I**) Separation of CS-shock trials into quartiles from highest to lowest defensive behavior. (**J**) Average defensive behaviors during CS-shock for each quartile in unpaired and paired groups. (**K**) Heat maps (top) and group average traces of ASt neuron z-score changes (bottom) in neural firing rate in response to CS-shock in Q1 and Q4 trials of paired and unpaired group mice. Heatmap rows represent average z-score PSTH for individual neurons. Arrowheads show average peak z-score during phasic window (0-100 ms). (**L**) Average ASt neuron phasic responses were greater in behavioral quartiles with more defensive behavior in paired mice, but not unpaired controls (Repeated measures ANOVA, Unpaired: $F_{(3,\ 210)}$ = 0.521, p = 0.668, Paired: $F_{(3,\ 570)}$ = 5.46, p = 0.0010. *p < 0.05, **p < 0.01 Tukey's multiple comparison test, n = 15 mice, 191 neurons Paired group, n = 4 mice, 71 neurons Unpaired group). (**M**) Average ASt neuron sustained responses were greater in behavioral quartiles with more defensive behavior in paired mice, but not unpaired controls (Repeated measures ANOVA, Unpaired: $F_{(3,\ 210)}$ = 0.424, p = 0.736, Paired: $F_{(3,\ 570)}$ = 10.26, p < 0.0001. *p < 0.05, **p < 0.01, ***p < 0.001 Tukey's multiple comparison test. n = 15 mice, 191 neurons Paired group, n = 4 mice, 71 neurons Unpaired group). (**N-U**) Analysis of ASt neuron responses to CS-reward across mice and trials grouped by latency of reward responses. (**N**) Two-tone discrimination task with electrophysiology recordings. Reward responses were calculated as the latency to reward port entry after tone onset. (**O**) Group average latency to port entry (reward response) during CS-reward was faster in paired group mice than unpaired group mice (t_{17} = 2.803, p = 0.0122, Student's t-test, n = 15 mice paired, 4 mice unpaired). (**P**) Hierarchical clustering of paired group mice into "low" and "high" learner/performer clusters based on reward responses. (**Q**) Group average reward responses were significantly faster in "high" performance mice than "low" performance and unpaired mice. (One-way ANOVA, $F_{(2,16)}$ = 28.59, p < 0.0001. ***p < 0.001, Tukey's multiple comparison test. n = 4 mice Unpaired, n = 4 mice "low" paired group, n = 11 mice "high" paired group). (**R**) Average ASt neuron phasic responses to CS-reward were not different in the "low" or "high" reward behavior groups (One-way ANOVA, $F_{(2,16)}$ = 2.178, p = 0.1457). (**S**) There was little correlation between ASt neuron phasic responses and mean reward latency. (**T**) Average latency to port entry during CS-reward for unpaired and paired groups, separated into quartiles determined based on relative latency of reward responses (Q1 = slowest response trials, Q4 = fastest response trials). (**U**) Average ASt neuron phasic responses were unchanged across different trial quartiles, regardless of changes in reward behavior (Repeated measures ANOVA, Paired: $F_{(3,\ 570)}$ = 0.576, p = 0.631). Bar graph and trace data shown as mean ±SEM.

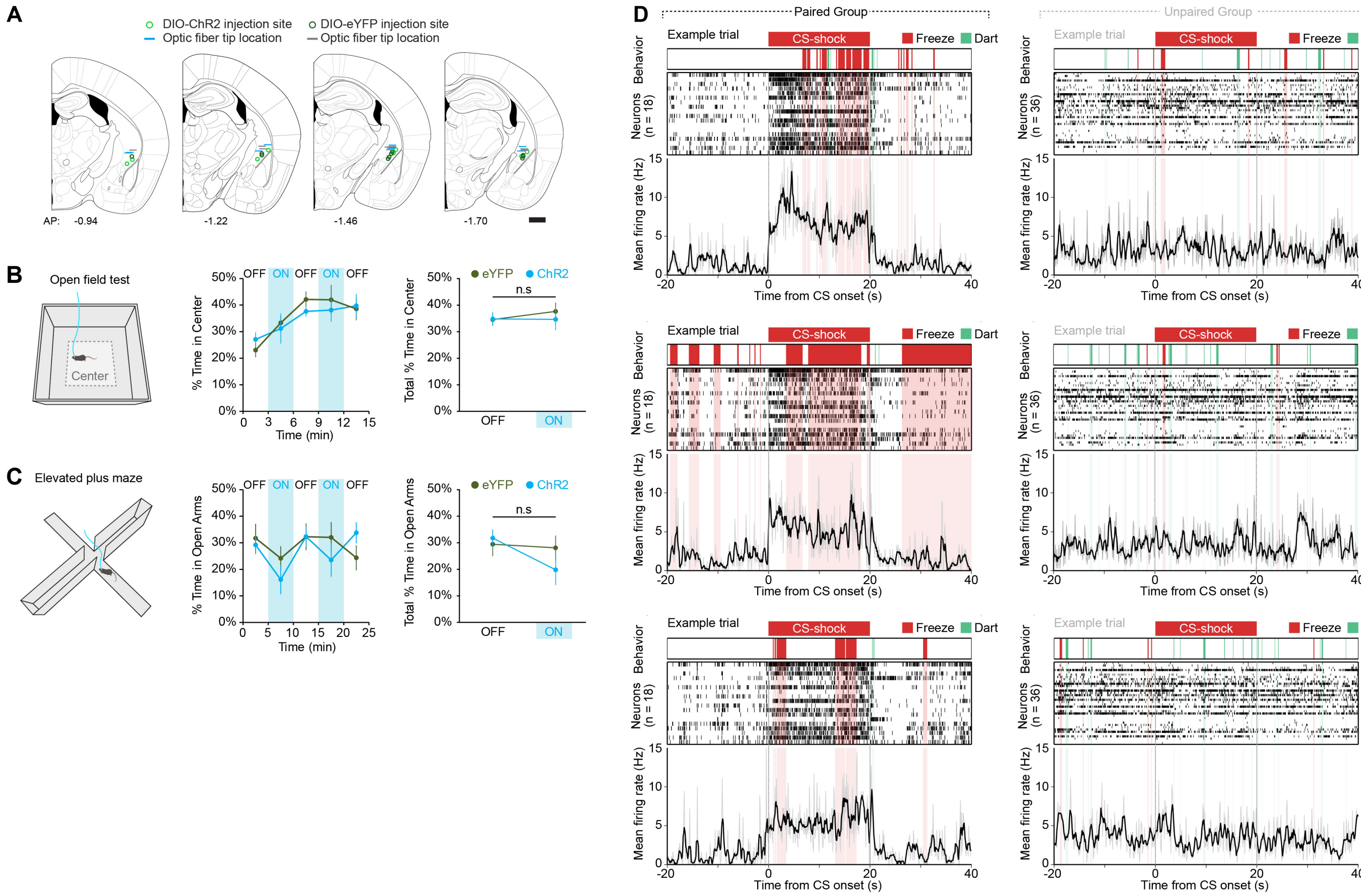

Figure S11. Optogenetic activation of ASt neurons does not affect anxiety-related behaviors.

(**A**) Histologically verified optic fiber implant locations and viral injection sites for AAV-DIO-ChR2-eYFP and AAV-DIO-eYFP controls in optogenetic excitation experiments targeting ASt neurons in *VGAT*-Cre mice. Scale bars = 500 µm.
(**B**) Optogenetic activation of ASt neurons had no effect on time spent in the center of an open field arena (Two-way ANOVA, group x laser interaction $F(1,32) = 0.11$, $p = 0.74$. ChR2: n = 8 mice, eYFP: n = 10 mice).
(**C**) Optogenetic activation of ASt neurons had no effect on the proportion of time spent in the open arms of an elevated plus maze (Two-way ANOVA, group x laser interaction $F(1,30) = 1.25$, $p = 0.27$. ChR2: n = 7 mice, eYFP: n = 10 mice).
(**D**) Additional representative trials from discrimination task experiments showing simultaneous subsecond behavior, neural spikes, and average neuron firing rate during presentation of CS-shock. In each example trial, neurons shown are all ASt neurons recorded from that mouse, during that trial.

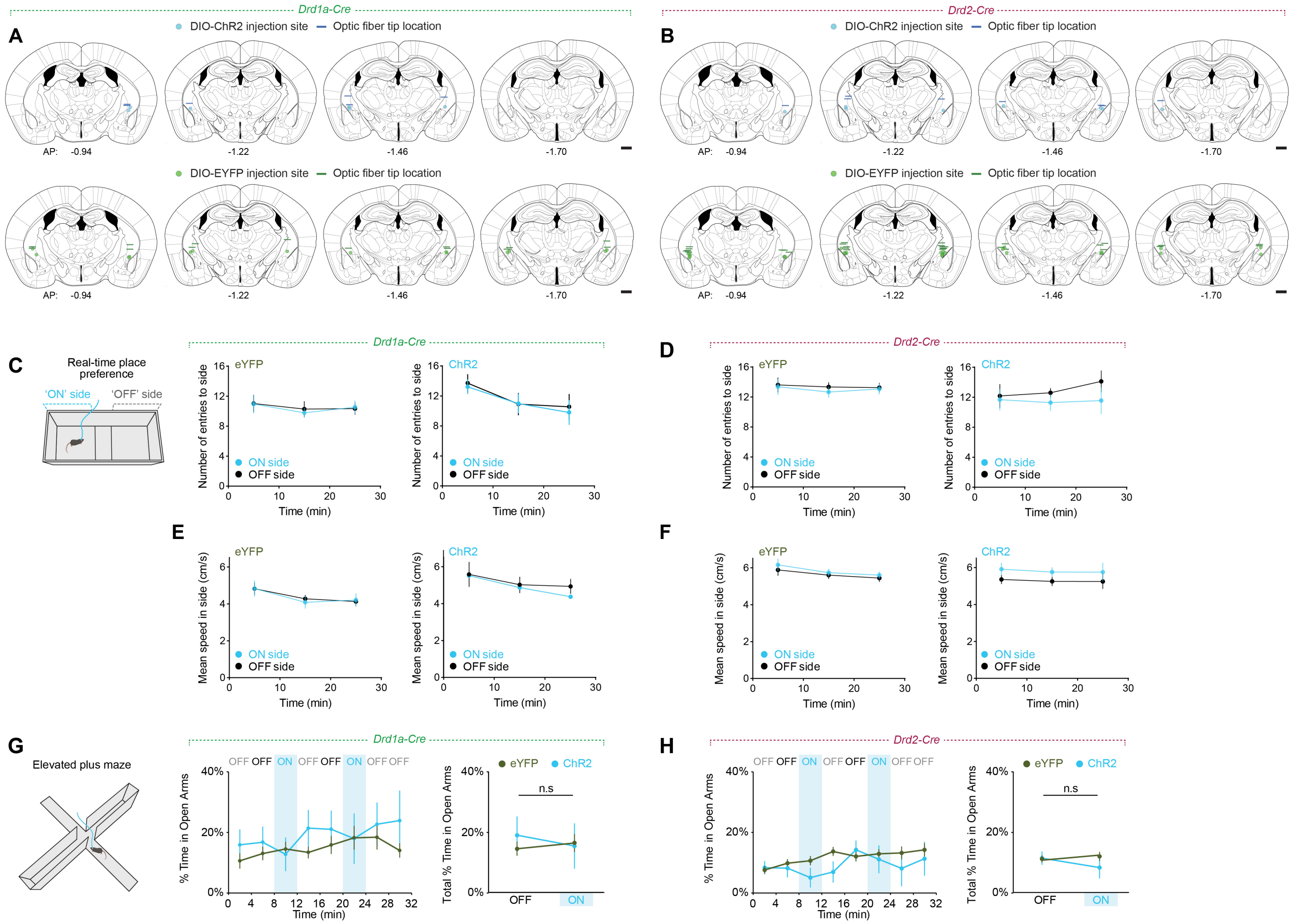

Figure S12. Optogenetic activation of *Drd1a*+ or *Drd2*+ ASt neurons does not affect side entries or overall locomotion during a real-time place preference task, or elevated plus maze behavior

(**A-B**) Histologically verified optic fiber implant locations and injection sites of AAV-DIO-ChR2-eYFP and AAV-DIO-eYFP in *Drd1a-cre* mice (**A**) and *Drd2-cre* mice (**B**). Scale bars = 500 µm.
(**C-F**) In real-time place preference tasks, optogenetic stimulation of *Drd1a*+ or *Drd2*+ ASt neurons had no significant effect on the number of entries to the 'ON' side or 'OFF' side (Two-way RM ANOVA, Drd1a-Cre ChR2: effect of side $F_{(1,8)}$ = 0.1086, p = 0.7502; eYFP: effect of side $F_{(1,12)}$ = 0.009449, p = 0.9242. Drd1a-Cre ChR2: n = 5 mice, eYFP: n = 7 mice; Two-way RM ANOVA, Drd2a-Cre ChR2: effect of side $F_{(1,10)}$ = 0.6994, p = 0.4225; eYFP: effect of side $F_{(1,30)}$ = 0.1386, p = 0.7123. Drd2a-Cre ChR2: n = 6 mice, eYFP: n = 16 mice), or the mean speed in the 'ON' side or 'OFF' side (Two-way RM ANOVA, Drd1a-Cre ChR2: effect of side $F_{(1,8)}$ = 0.2172, p = 0.6536; eYFP: effect of side $F_{(1,12)}$ = 0.0008348, p = 0.9774. Drd1a-Cre ChR2: n = 5 mice, eYFP: n = 7 mice; Two-way RM ANOVA, Drd2a-Cre ChR2: effect of side $F_{(1,10)}$ = 1.951, p = 0.1927; eYFP: effect of side $F_{(1,30)}$ = 0.3750, p = 0.5449. Drd2a-Cre ChR2: n = 6 mice, eYFP: n = 16 mice).
(**G-H**) Optogenetic stimulation of *Drd1a*+ or *Drd2*+ ASt neurons had no effect on time spent in open arms of an elevated plus maze. (Two-way ANOVA, Drd1a-Cre, group x laser interaction $F_{(1,20)}$ = 0.4057, p = 0.5314. *Drd1a*-Cre ChR2: n = 5 mice, eYFP: n = 7 mice; Two-way ANOVA, *Drd2a*-Cre, group x laser interaction $F_{(1,40)}$ = 0.9907, p = 0.3256. Drd2a-Cre ChR2: n = 6 mice, eYFP: n = 16 mice). Data presented as mean ±SEM.

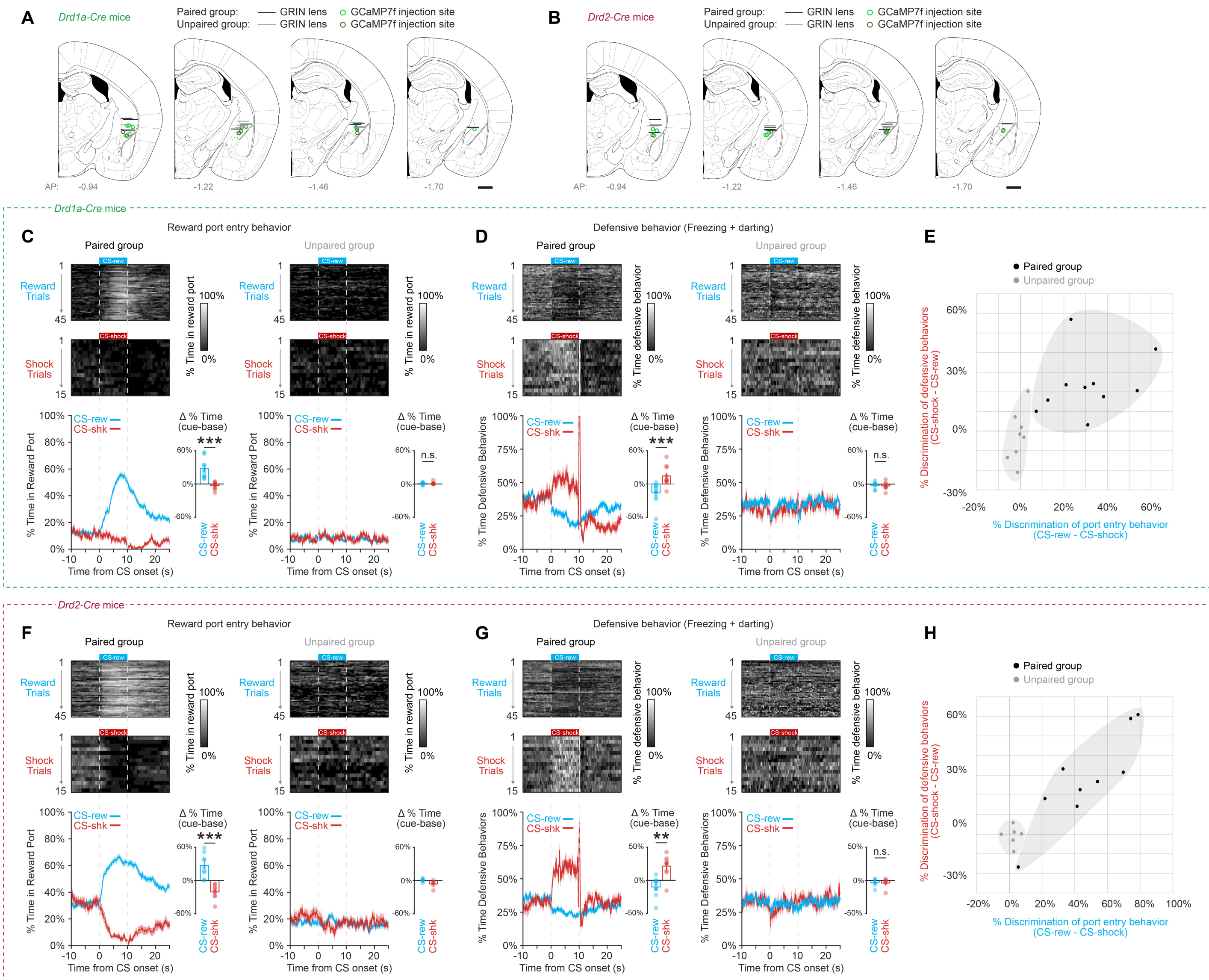

Figure S13. Targeting of ASt neurons and behavioral validation for *in vivo* calcium imaging.

(**A-B**) Histologically verified GRIN lens implant locations and AAV1-Syn-FLEX-GCaMP7f viral injection sites in recording experiments targeting *Drd1a*+ neurons (**A**) and *Drd2*+ neurons (**B**) in the ASt.
(**C-E**) Validation of conditioned behavioral responses during two-tone discrimination task in *Drd1a-Cre* mice (*Drd1a-Cre* Paired: n = 10 mice, *Drd1a-Cre* Unpaired: n = 8 mice). (**C**) Paired group mice showed distinct responses to the CS-shock and CS-reward tones in reward port entry behavior (Two-tailed paired t-test, t_9 = 6.400, ***p = 0.000125). (**D**) Paired group mice also showed distinct defensive responses to CS-shock and CS-reward tones (Two-tailed paired t-test, t_9 = 5.418, ***p = 0.0004), consistent with successful discrimination between the two tones. Neither measure differed between cue types in unpaired mice (port entry, t_7 = 0.373, p = 0.720; defensive responses, t_7 = 0.300, p = 0.773).
(**E**) 'Discrimination scores' of reward port entry behavior (CS-rew − CS-shock) plotted against defensive behavior (CS-shock − CS-rew) for each mouse.
(**F-H**) Validation of conditioned behavioral responses during two-tone discrimination task in *Drd2-Cre* mice (*Drd2-Cre* Paired: n = 9 mice, *Drd2-Cre* Unpaired: n = 6 mice). Paired group mice showed distinct responses to the CS-shock and CS-reward tones in (**F**) reward port entry behavior (Two-tailed paired t-test, t_8 = 6.43, ***p = 0.0002) and (**G**) defensive responses (Two-tailed paired t-test, t_8 = 3.381, **p = 0.0096), consistent with successful discrimination between the two tones. In unpaired mice, defensive responses did not differ between cue types (t5 = 0.110, p = 0.917); reward port entry differed slightly (t5 = 2.83, p = 0.0365), though the mean discrimination score was approximately 7-fold smaller in magnitude than in paired mice. (**H**) 'Discrimination scores' of reward port entry behavior (CS-rew − CS-shock) plotted against defensive behavior (CS-shock − CS-rew) for each mouse.

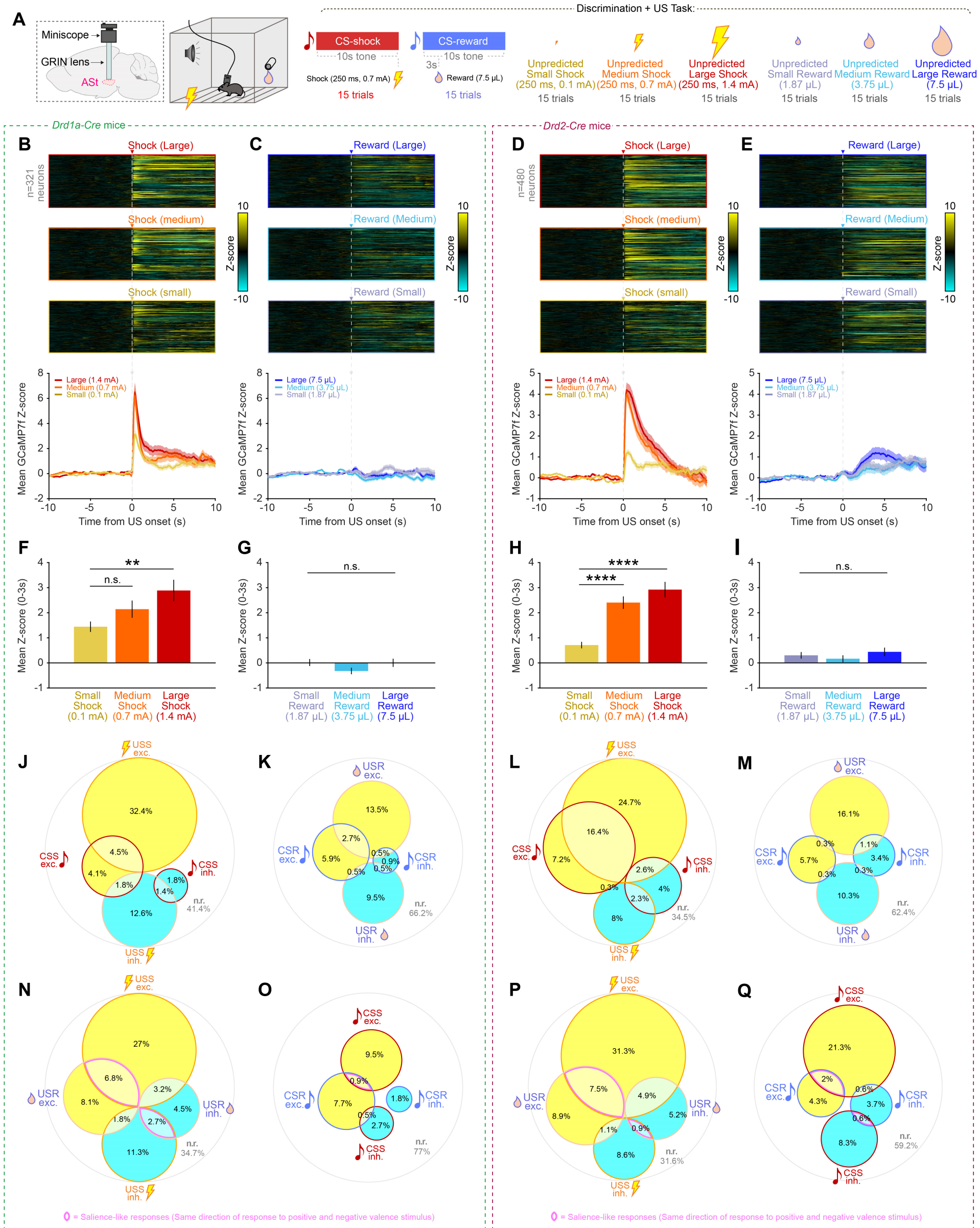

Figure S14. ASt neuron responses to unconditioned stimuli are heterogeneous and scale in magnitude for unpredicted shocks but not for unpredicted rewards.

(**A**) Experimental task design for examining US responses in *Drd1a*+ and *Drd2*+ ASt neurons. In addition to normal CS-shock and CS-reward trials, mice were also presented with unpredicted deliveries of US-shock of different magnitudes (0.1, 0.7 or 1.4 mA), and US-reward of different magnitudes (1.87, 3.75 or 7.5 µL chocolate Ensure™). (**B-E**) Individual neuron traces (top) and group averages (bottom) of z-score changes in GCaMP7f fluorescence for (**B**) *Drd1a*+ ASt neuron responses to US-shock, (**C**) *Drd1a*+ ASt neuron responses to US-reward deliveries, (**D**) *Drd2*+ ASt neuron responses to US-shock and (**E**) *Drd2*+ ASt neuron responses to US-reward deliveries. (**F-I**) Quantification of mean z-score responses to US deliveries of different magnitudes (0-3s after US onset) for (**F**) *Drd1a*+ ASt neuron responses to US-shock, (**G**) *Drd1a*+ ASt neuron responses to US-reward, (**H**) *Drd2*+ ASt neuron responses to US-shock, and (**I**) *Drd2*+ ASt neuron responses to US-reward (One-way ANOVA: (**F**) $F(2,960) = 4.53$, $p = 0.011$; (**G**) $F(2,960) = 1.77$, $p = 0.17$; (**H**) $F(2,1437) = 23.12$, $p = 1.3 \times 10^{-10}$; (**I**) $F(2,1437) = 0.86$, $p = 0.42$; followed by post hoc t-tests with Bonferroni correction for 3 comparisons, **$p < 0.01$, ****$p < 0.0001$). (**J-Q**) Euler diagrams of *Drd1a*+ ASt neuron co-responsiveness to (**J**) US-shock and CS-shock, and (**K**) US-reward and CS-reward. In all Euler diagrams, neurons were classified as 'excited' by the stimulus if their mean z-score was greater than 2.58 (which corresponds to a *p*-value of 0.01) in 0-3s post-stimulus onset window, and classified as 'inhibited' by the stimulus if their mean z-score was less than -2.58. (**L-M**) Euler diagrams of *Drd2*+ ASt neuron co-responsiveness to (**L**) US-shock and CS-shock, and (**M**) US-reward and CS-reward. (**N-O**) Euler diagrams of *Drd1a*+ ASt neuron co-responsiveness to (**N**) US-shock and US-reward, and (**O**) CS-shock and CS-reward. (**P-Q**) Euler diagrams of *Drd2*+ ASt neuron co-responsiveness to (**P**) US-shock and US-reward, and (**Q**) CS-shock and CS-reward. Pink inner borders indicate 'salience-like' responses, where neurons respond in the same direction (excited or inhibited) to positive or negative valence stimuli. For data examining US responses (panels B-I), *Drd1a*+: $n = 7$ mice, 321 neurons, *Drd2*+: $n = 8$ mice, 480 neurons. For data examining CS and US co-responsiveness (panels J-Q), only data from paired group mice (where each CS had meaning) was used: *Drd1a*+: $n = 4$ mice, 222 neurons, *Drd2*+: $n = 5$ mice, 348 neurons.

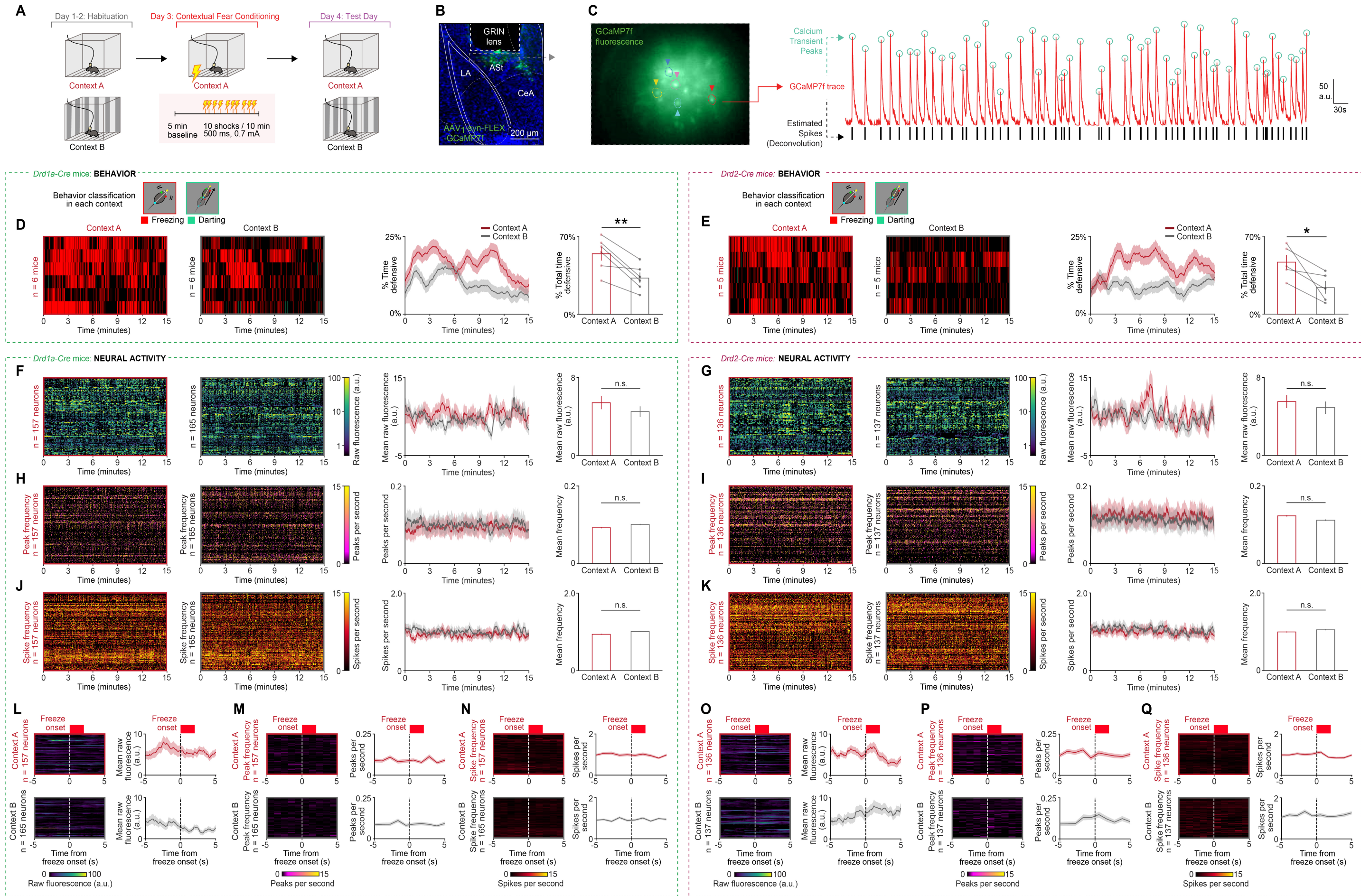

Figure S15. ASt neurons do not show changes in activity following contextual fear conditioning.

(**A**) Contextual fear conditioning task experimental design. (**B**) GCaMP7f expression and lens targeting to ASt. (**C**) Calcium imaging field of view and representative traces of fluorescence, transient peaks detected and estimated spikes during contextual fear task. (**D-E**) Heatmaps and group averages of defensive behaviors during test day in Context A (conditioned) and Context B (unconditioned), in *Drd1a*+ ASt neurons (**D**) and *Drd2*+ ASt neurons (**E**). Mice in both groups spent significantly more total time freezing in Context A (conditioned) compared to Context B (unconditioned) (paired t-test, * $p < 0.05$, ** $p < 0.01$). (**F-G**) Heatmaps and group averages of raw neuron fluorescence in Context A and Context B on test day in *Drd1a*+ ASt neurons (**F**) and *Drd2*+ ASt neurons (**G**). (**H-I**) Heatmaps and group averages of frequency of calcium transient peaks in Context A and Context B on test day in *Drd1a*+ ASt neurons (**H**) and *Drd2*+ ASt neurons (**I**). (**J-K**) Heatmaps and group averages of frequency of estimated spike firing rates in Context A and Context B on test day in *Drd1a*+ ASt neurons (**J**) and *Drd2*+ ASt neurons (**K**). For F-K, paired t-test across mice all $p > 0.05$. (**L**) Heatmaps and group averages of *Drd1a*+ ASt neuron raw fluorescence at the onset of freezing bouts in Context A and Context B. (**M**) Heatmaps and group averages of *Drd1a*+ ASt calcium transient peaks at the onset of freezing bouts in Context A and Context B. (**N**) Heatmaps and group averages of *Drd1a*+ ASt estimated spike firing rates at the onset of freezing bouts in Context A and Context B. (**O**) Heatmaps and group averages of *Drd2*+ ASt neuron raw fluorescence at the onset of freezing bouts in Context A and Context B. (**P**) Heatmaps and group averages of *Drd2*+ ASt calcium transient peaks at the onset of freezing bouts in Context A and Context B. (**Q**) Heatmaps and group averages of *Drd2*+ ASt estimated spike firing rates at the onset of freezing bouts in Context A and Context B. No overall changes in neural activity to either context, or bouts of freezing while in either context, were observed in either *Drd1a*+ or *Drd2*+ ASt neurons. *Drd1a*+ Context A: $n = 6$ mice, 157 neurons, *Drd1a*+ Context B: $n = 6$ mice, 165 neurons, *Drd2*+ Context A: $n = 5$ mice, 136 neurons, *Drd2*+ Context B: $n = 5$ mice, 137 neurons.

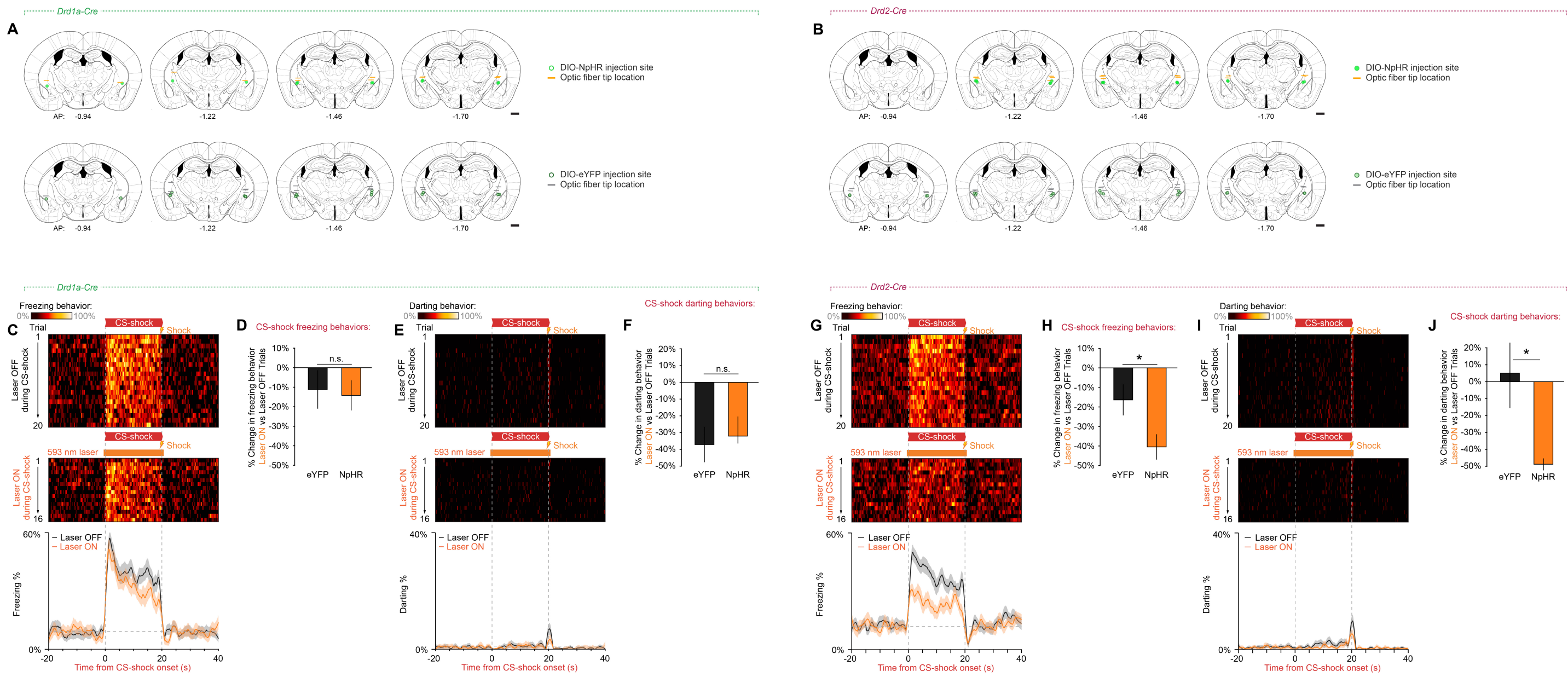

Figure S16. Targeting of *Drd1a*+ and *Drd2*+ ASt neurons and separate quantification of defensive behaviors during optogenetic inhibition.

(**A-B**) Histologically verified optic fiber implant locations and injection sites of AAV-DIO-NpHR-eYFP and AAV-DIO-eYFP in optogenetic inhibition experiments in *Drd1a-Cre* and *Drd2-Cre* mice. Scale bars = 500 µm.
(**C**) *Drd1a-Cre* freezing behavior heatmaps for CS-shock 'laser OFF' (top) and 'laser ON' trials (middle), and average freezing behavior during CS-shock trials with and without photoinhibition (bottom).
(**D**) Inhibition of Drd*1a*+ ASt neurons had no effect on change in conditioned freezing behaviors to CS-shock in NpHR mice compared with eYFP controls (NpHR: −14.2 ± 7.7%; eYFP: −11.1 ± 9.8%; t_{13} = 0.223, p = 0.827, Student's t-test; Drd1a+ NpHR: n = 6 mice, Drd1a+ eYFP: n = 9 mice).
(**E**) *Drd1a-Cre* darting behavior heatmaps for CS-shock 'laser OFF' (top) and 'laser ON' trials (middle), and average darting behavior during CS-shock trials with and without photoinhibition (bottom).
(**F**) Inhibition of Drd*1a*+ ASt neurons had no effect on change in conditioned darting behaviors to CS-shock in NpHR mice compared with eYFP controls (NpHR: −33.0 ± 8.1%; eYFP: −37.1 ± 10.5%; t_{13} = 0.279, p = 0.784, Student's t-test; Drd1a+ NpHR: n = 6 mice, Drd1a+ eYFP: n = 9 mice).
(**G**) *Drd2-Cre* freezing behavior heatmaps for CS-shock 'laser OFF' (top) and 'laser ON' trials (middle), and average freezing behavior during CS-shock trials with and without photoinhibition (bottom).
(**H**) Inhibition of *Drd2*+ ASt neurons resulted in a significantly greater reduction in conditioned freezing behaviors to CS-shock in NpHR mice compared with eYFP controls (NpHR: −40.4 ± 6.4%; eYFP: −16.3 ± 7.9%; t_{19} = 2.120, *p = 0.047, Student's t-test; Drd2+ NpHR: n = 8 mice, Drd2+ eYFP: n = 13 mice).
(**I**) *Drd2-Cre* darting behavior heatmaps for CS-shock 'laser OFF' (top) and 'laser ON' trials (middle), and average darting behavior during CS-shock trials with and without photoinhibition (bottom).
(**J**) Inhibition of *Drd2*+ ASt neurons resulted in a significantly greater reduction in conditioned darting behaviors to CS-shock in NpHR mice compared with eYFP controls (NpHR: −48.9 ± 3.5%; eYFP: 3.7 ± 19.4%; t_{19} = 2.097, *p = 0.0496, Student's t-test; Drd2+ NpHR: n = 8 mice, Drd2+ eYFP: n = 13 mice). *p < 0.05. All data shown as mean ± SEM.

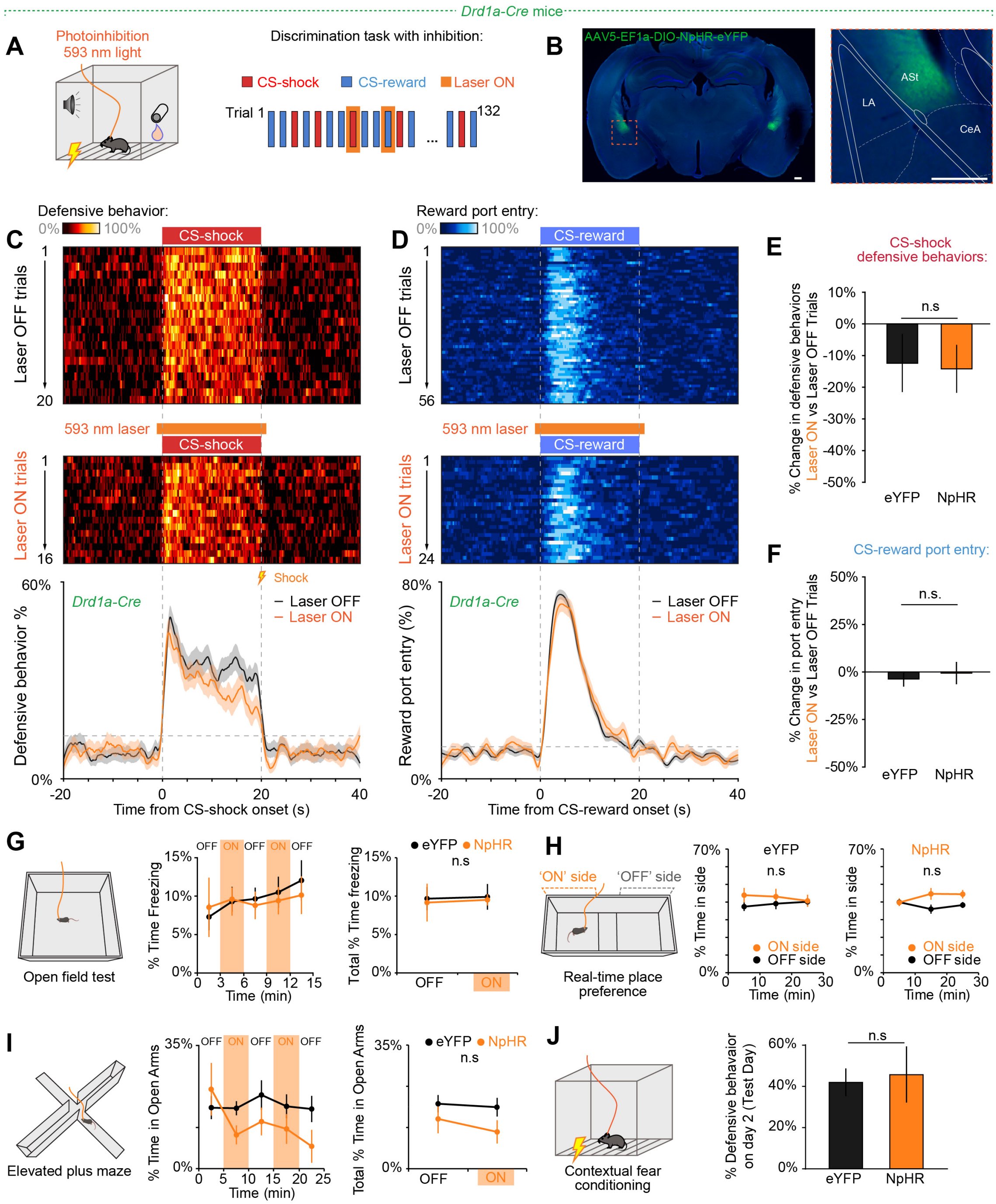

Figure S17. Optogenetic inhibition of D1+ ASt neurons does not affect behavioral responses to cues or context.

(**A**) Behavioral paradigm for selective inhibition of *Drd1a*+ ASt neurons during discrimination task. (**B**) Representative images of AAV-DIO-NpHR-eYFP expression in *Drd1a*+ ASt neurons (Scale bars = 250 µm). (**C**) Defensive behavior heatmaps during 'laser OFF' (top) and 'laser ON' trials (middle) and group averages (bottom) during CS-shock trials with and without photoinhibition. (**D**) Port entry behavior heatmaps during laser OFF and ON (top, middle) and group averages (bottom) during CS-reward trials with and without photoinhibition. (**E**) Inhibition of Drd1a+ ASt neurons had no effect on conditioned defensive behaviors to CS-shock in NpHR mice compared with eYFP controls (t_{13} = 0.137, p = 0.893, Student's t-test), and (**F**) had no effect on port entry during CS-reward (t_{13} = 0.493, p = 0.630, Student's t-test). (**G**) Optogenetic inhibition of D1+ ASt neurons had no effect on overall locomotion in an open field task. (**H**) Inhibition of Drd1a+ ASt neurons did not cause preference or aversion for an area paired with inhibition in a real-time place preference task (Two-way RM ANOVA, NpHR: effect of side F(1,6) = 4.358, p = 0.0819; eYFP: effect of side F(1,16) = 0.6248, p = 0.4408). NpHR: n = 4 mice, eYFP: n = 9 mice. (**I**) Inhibition of *Drd1a*+ ASt neurons caused no overall changes in exploration of open arms in an elevated plus maze. (**J**) Inhibition of *Drd1a*+ ASt neurons had no effect on defensive behaviors on day 2 of a contextual fear conditioning task. *Drd1a*+ NpHR: *n* = 6 mice, *Drd1a*+ eYFP: *n* = 9 mice. All data shown as mean ±SEM.
