# Supplementary material for: Amygdalostriatal transition zone neurons encode sustained cue responses to guide defensive behaviors
Source: Neuron. Author manuscript; Available in PMC 2026 Sep 21. (PMC13592531; doi:10.1016/j.neuron.2026.08.012)
Supplement: Supplementary file 2 [file NIHMS2206276-supplement-MMC2.pdf]

Mills et al. 2026

Data S1 - Appendix A

Histology for Electrophysiology lesions (single wire) and tracks (Neuropixels)

Appendix A - Histology (Electrophysiology)

C57BL6/J Paired group "Mouse ID: NPX03" Data included

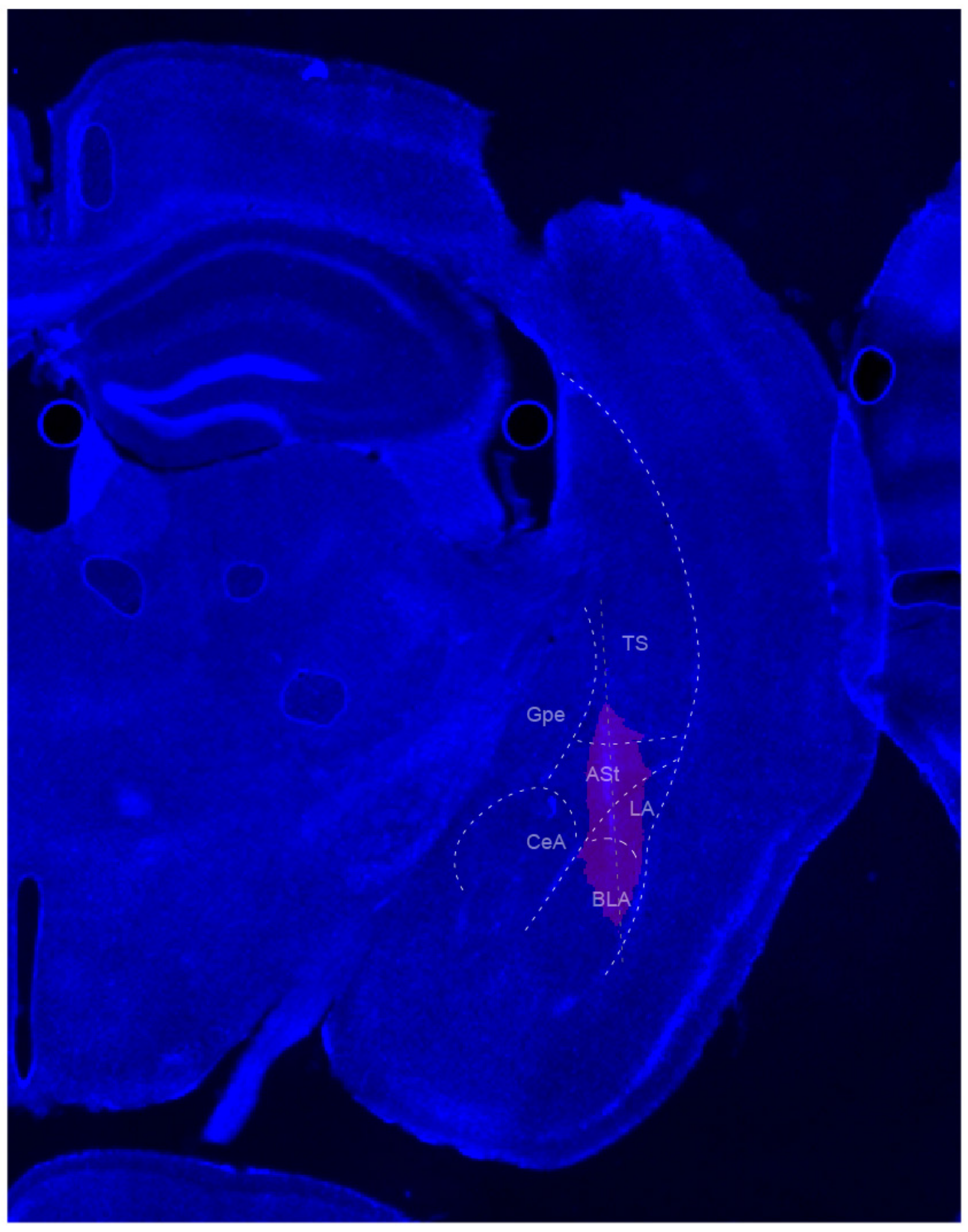

Blue = DAPI
Pink = DiI (probe)

C57BL6/J Paired group Mouse ID: NPX04 Data included

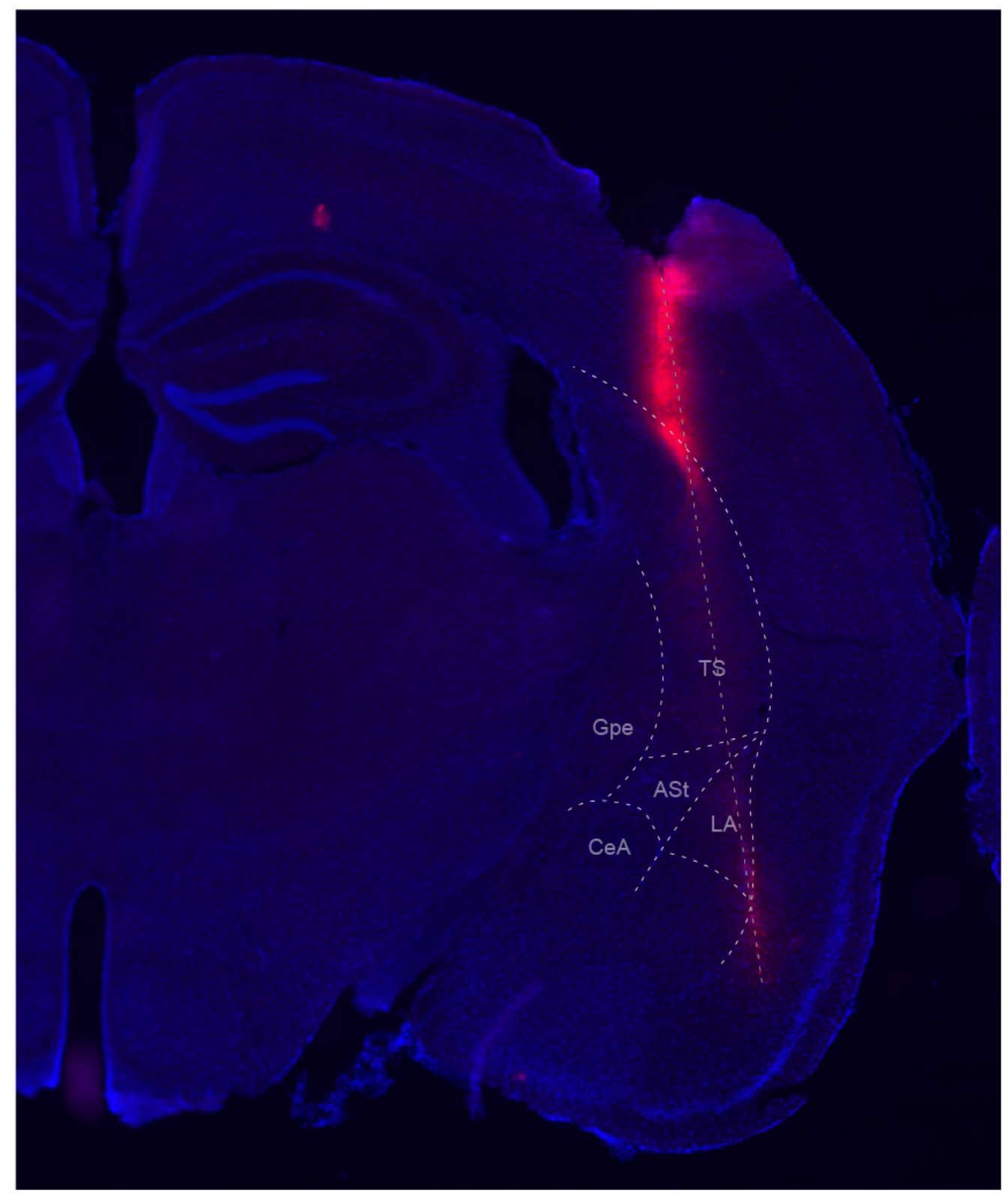

Blue = DAPI
Pink = DiI (probe)

C57BL6/J Paired group "Mouse ID: NPX05" Data included

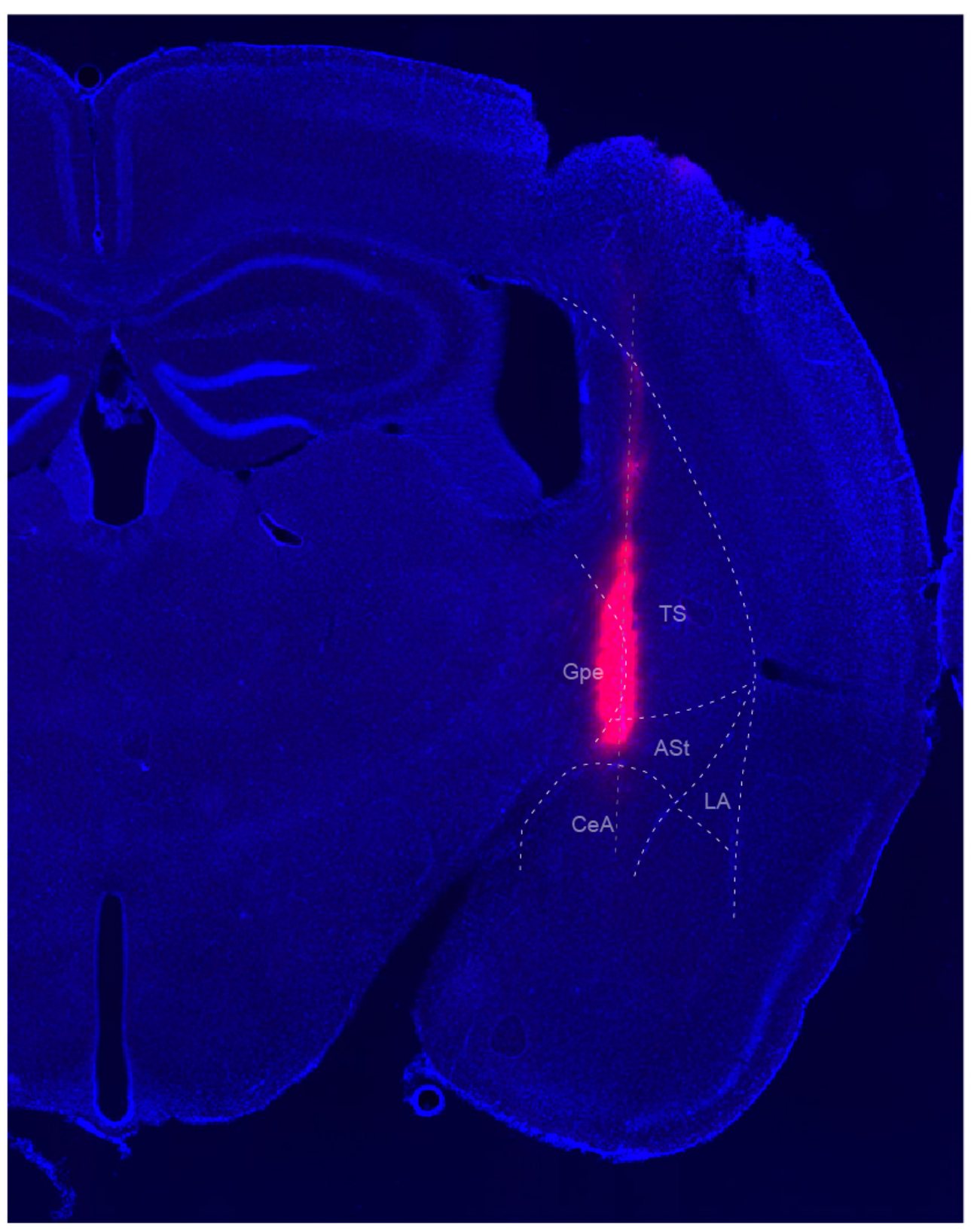

Blue = DAPI
Pink = DiI (probe)

C57BL6/J Unpaired group Mouse ID: NPX06 Data included

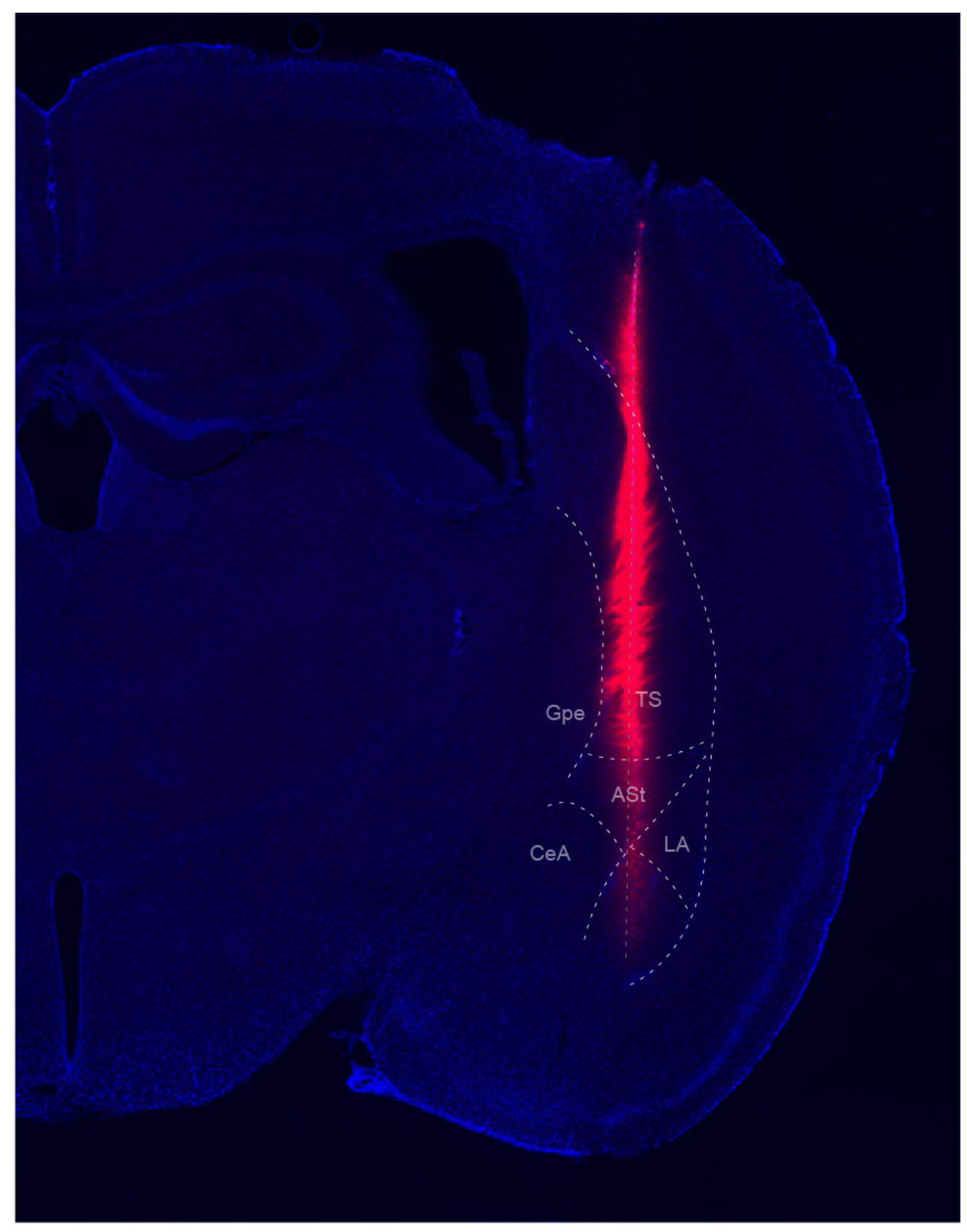

Blue = DAPI
Pink = DiI (probe)

C57BL6/J Unpaired group "Mouse ID: NPX07" Data included

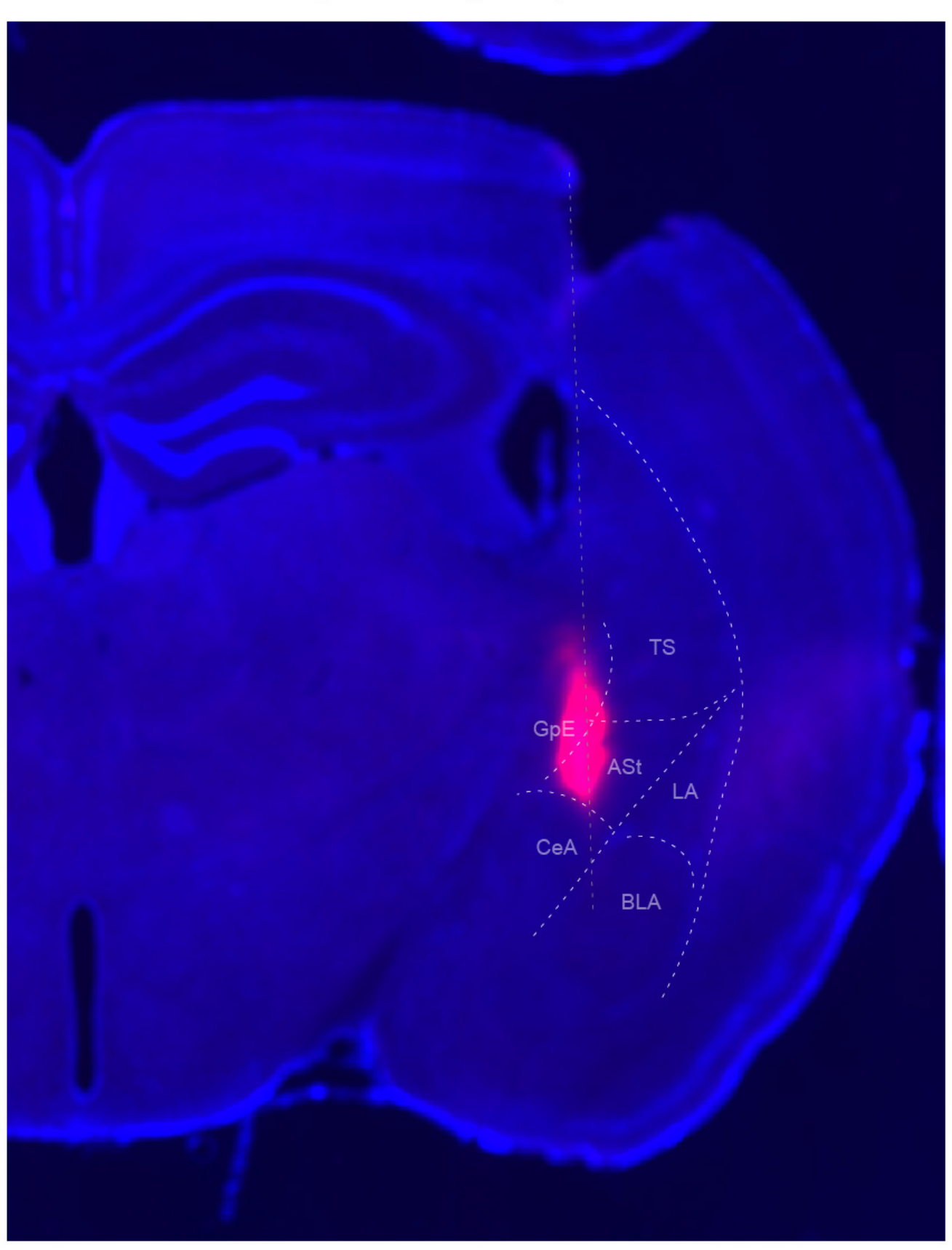

Blue = DAPI
Pink = DiI (probe)

C57BL6/J Paired group "Mouse ID: NPX_08" Data included

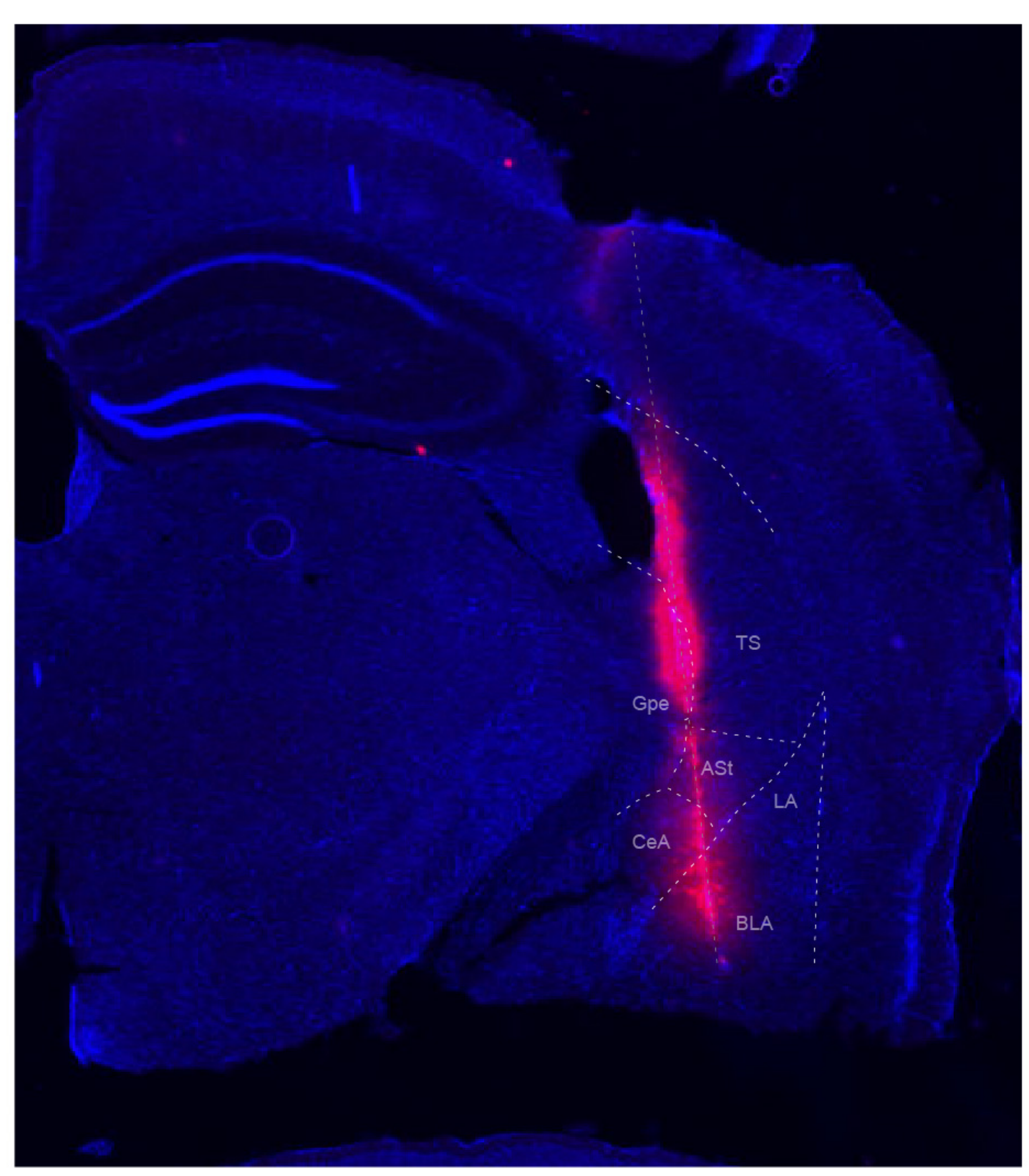

Blue = DAPI
Pink = DiI (probe)

Appendix A - Histology (Electrophysiology)

C57BL6/J Paired group "Mouse ID: NPX_09" Data included

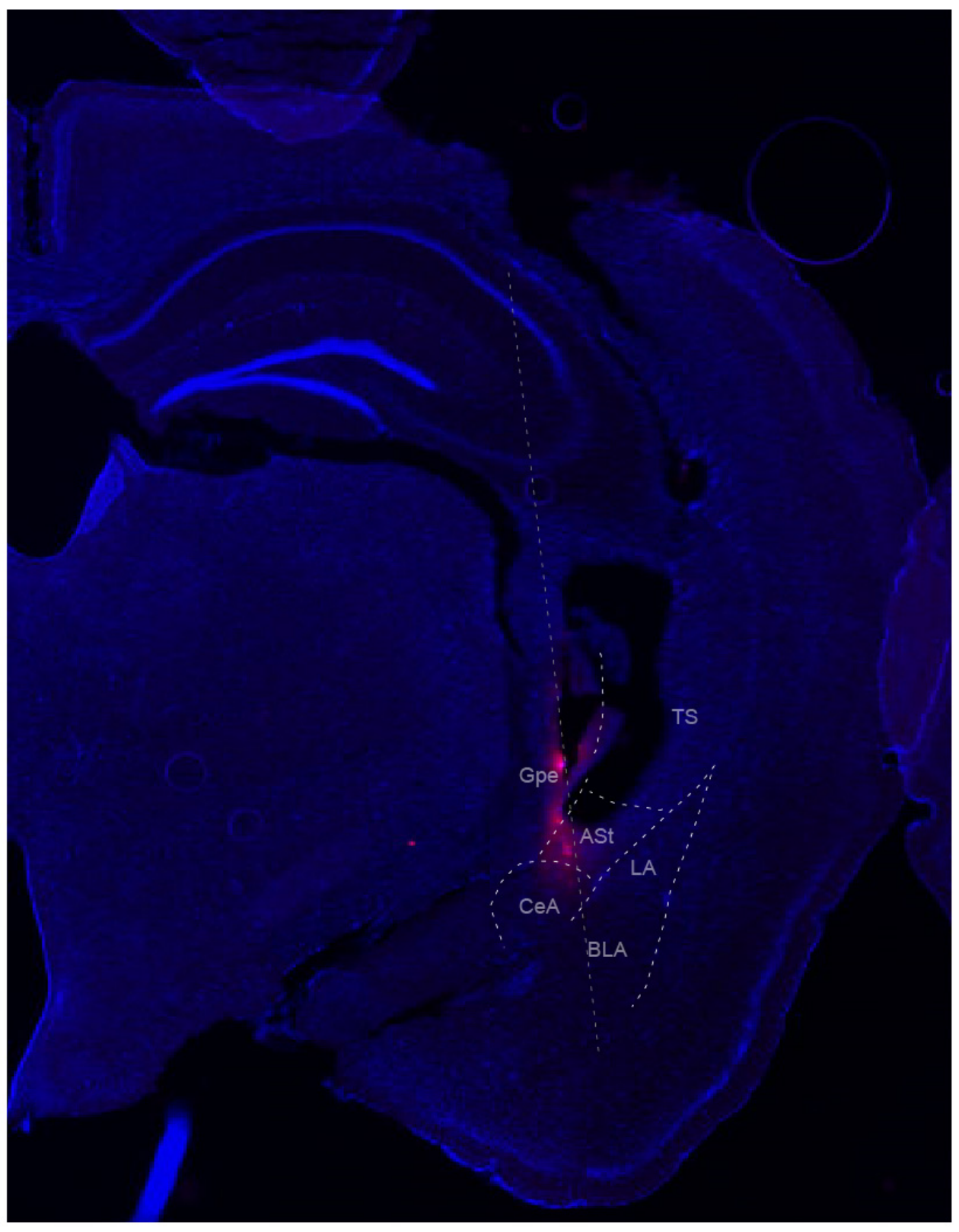

✓

Blue = DAPI
Pink = Dil (probe)

C57BL6/J Paired group "Mouse ID: NPX_10" Data included

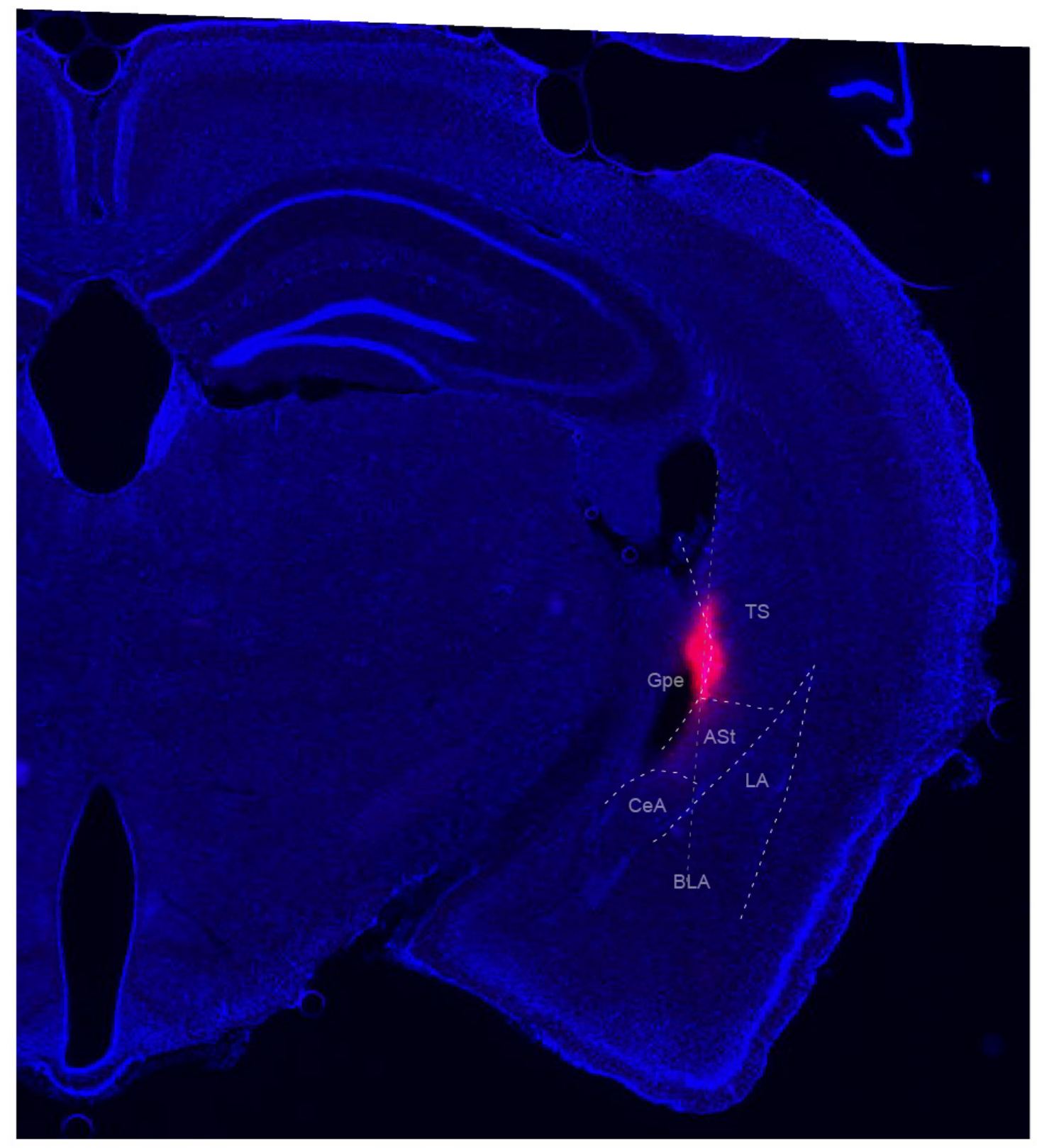

✓

Blue = DAPI
Pink = Dil (probe)

C57BL6/J Paired group "Mouse ID: NPX_11" Data included

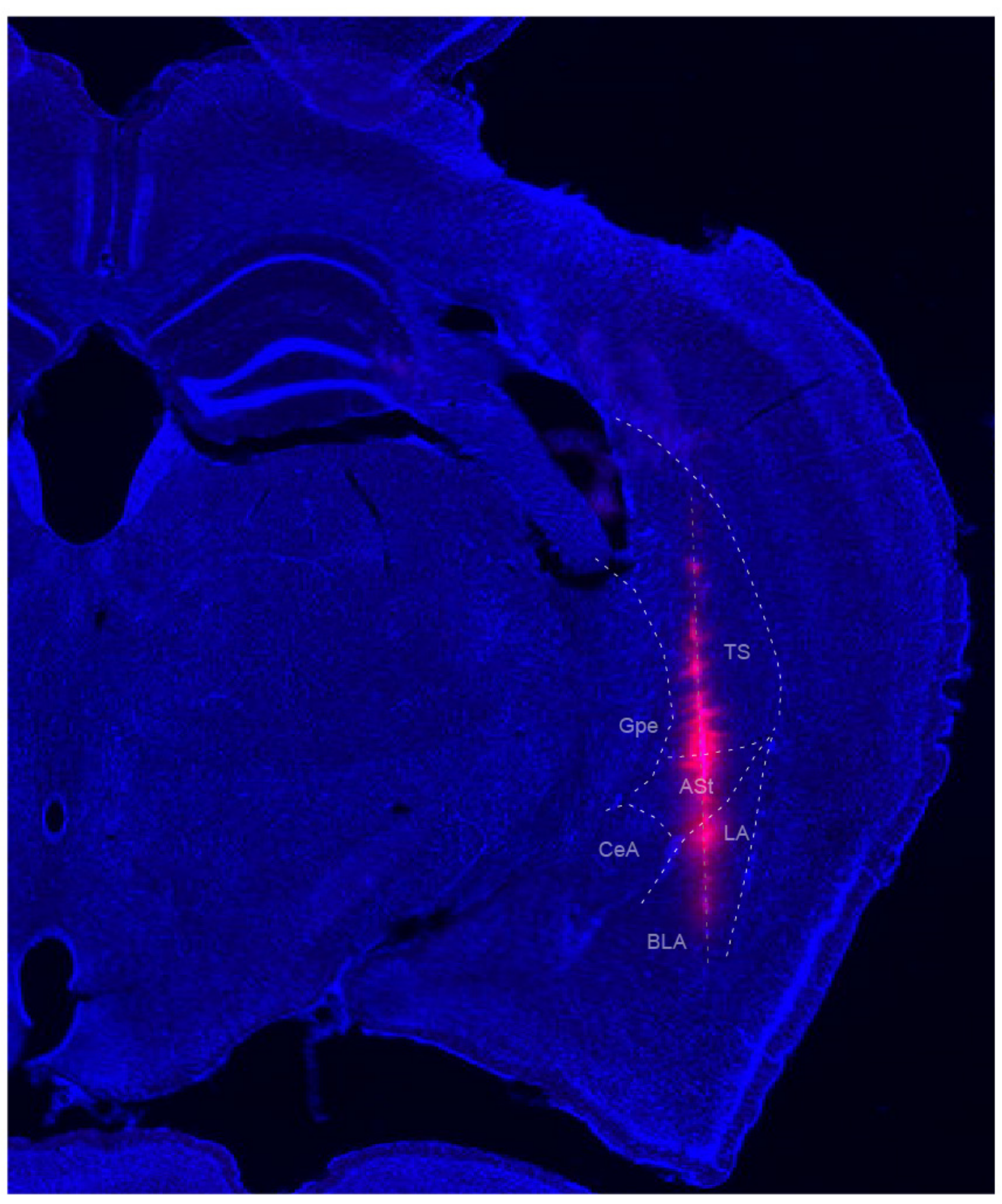

✓

Blue = DAPI
Pink = Dil (probe)

C57BL6/J Paired group "Mouse ID: NPX_CeA_01" Data included

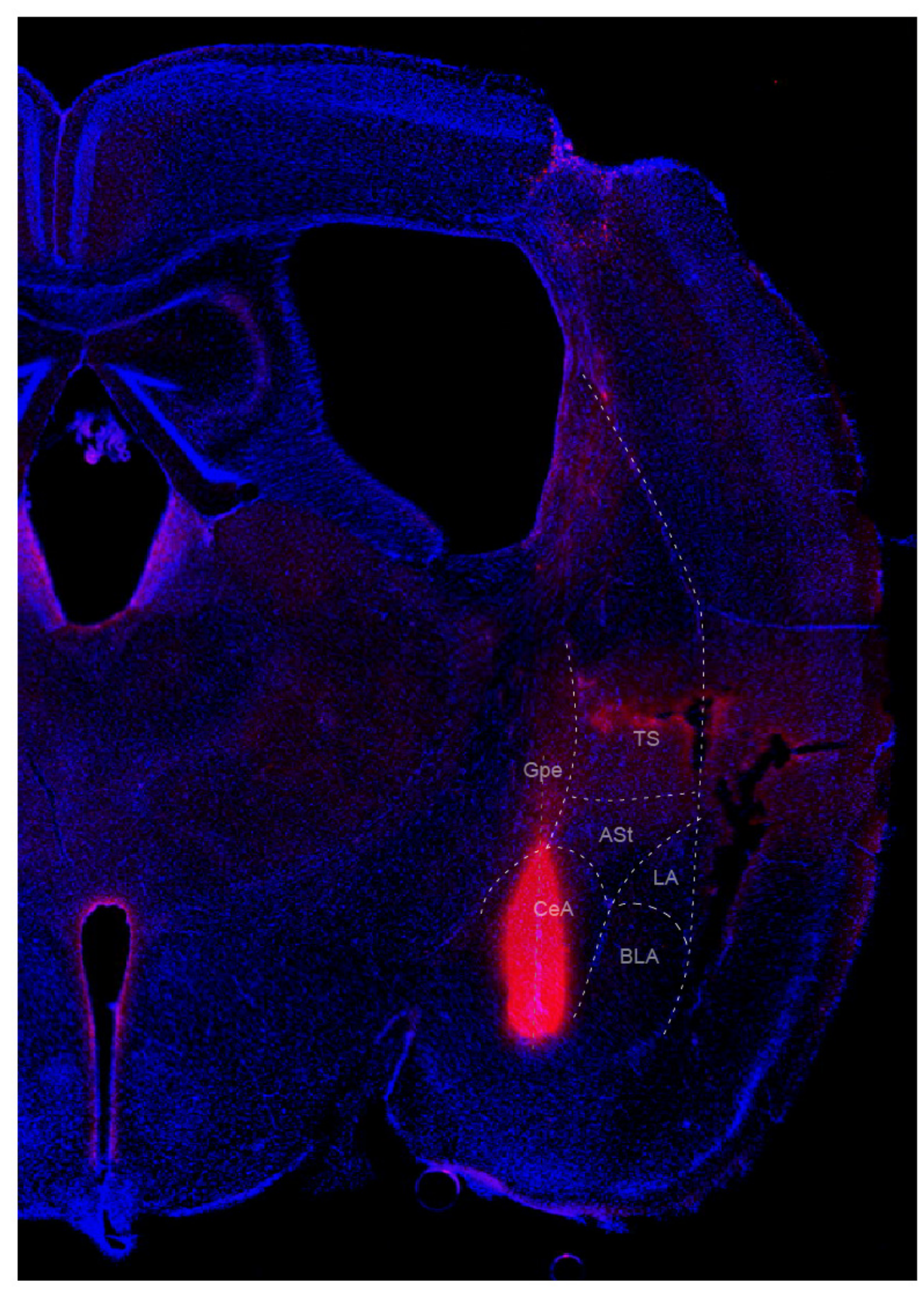

✓

Blue = DAPI
Pink = Dil (probe)

C57BL6/J Paired group "Mouse ID: NPX_CeA_02" Data included

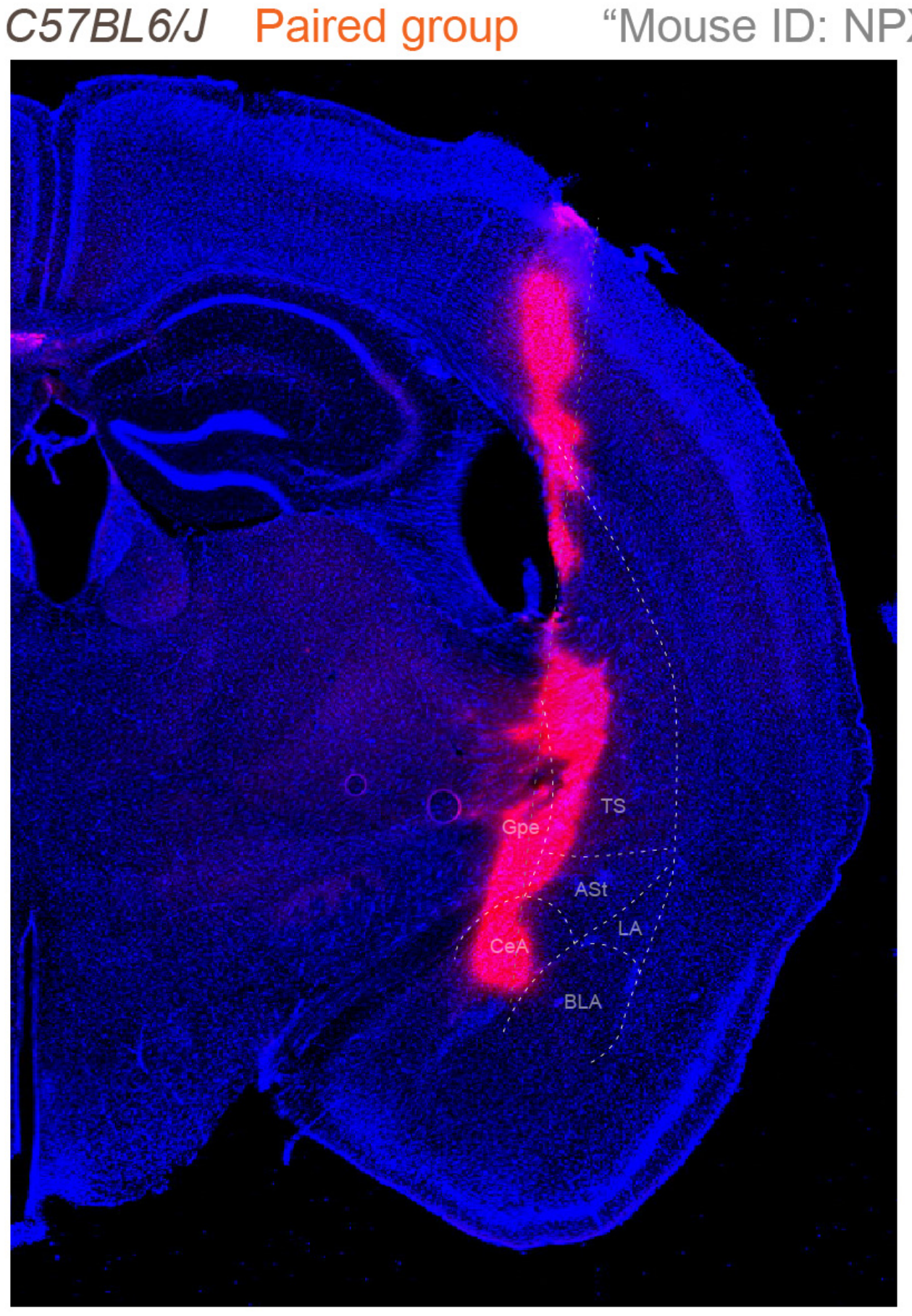

✓

Blue = DAPI
Pink = Dil (probe)

C57BL6/J Paired group "Mouse ID: NPX_CeA_04" Data included

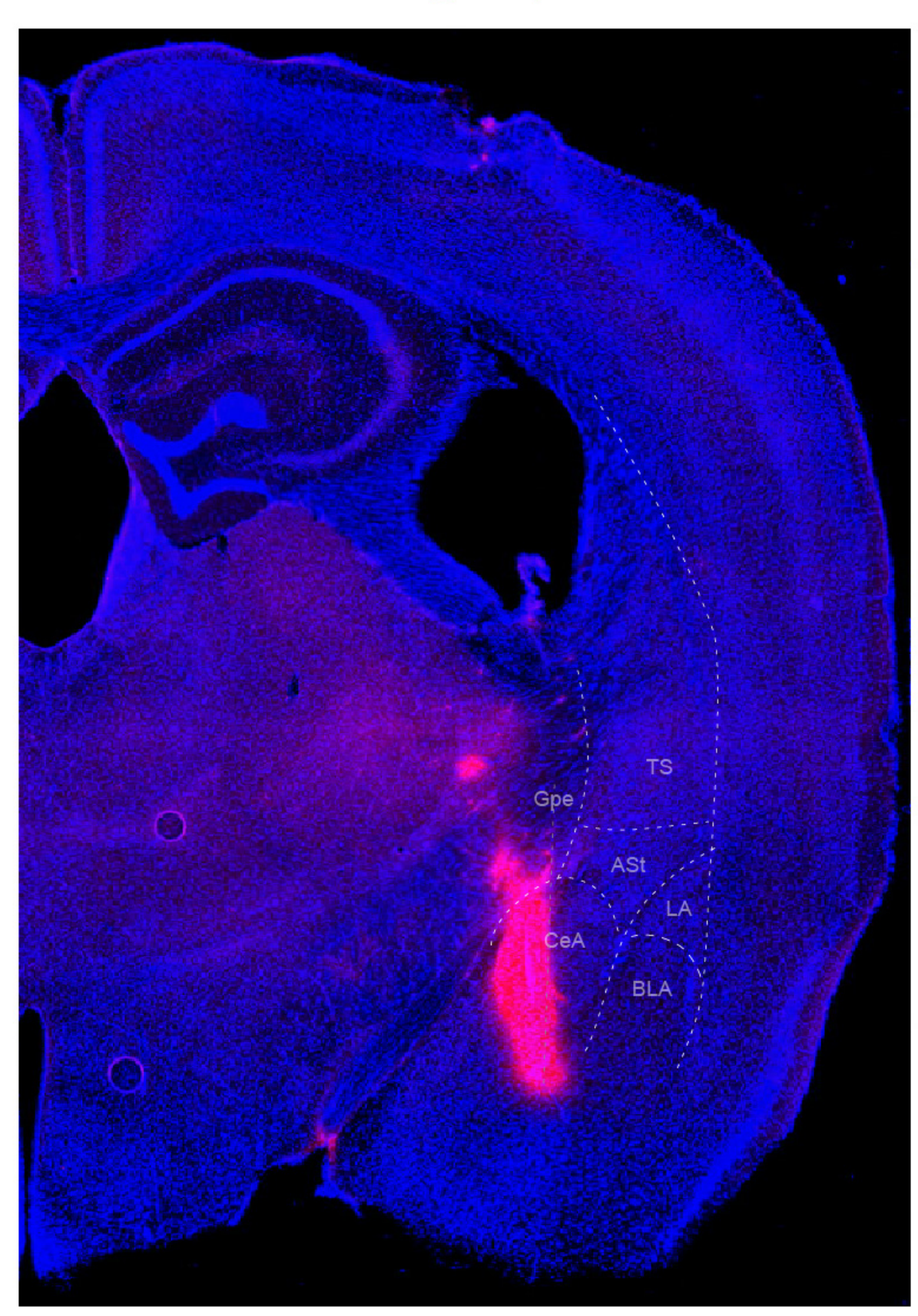

✓

Blue = DAPI
Pink = Dil (probe)

Appendix A - Histology (Electrophysiology)

C57BL6/J Paired group "Mouse ID: NPX_CeA_05" Data included

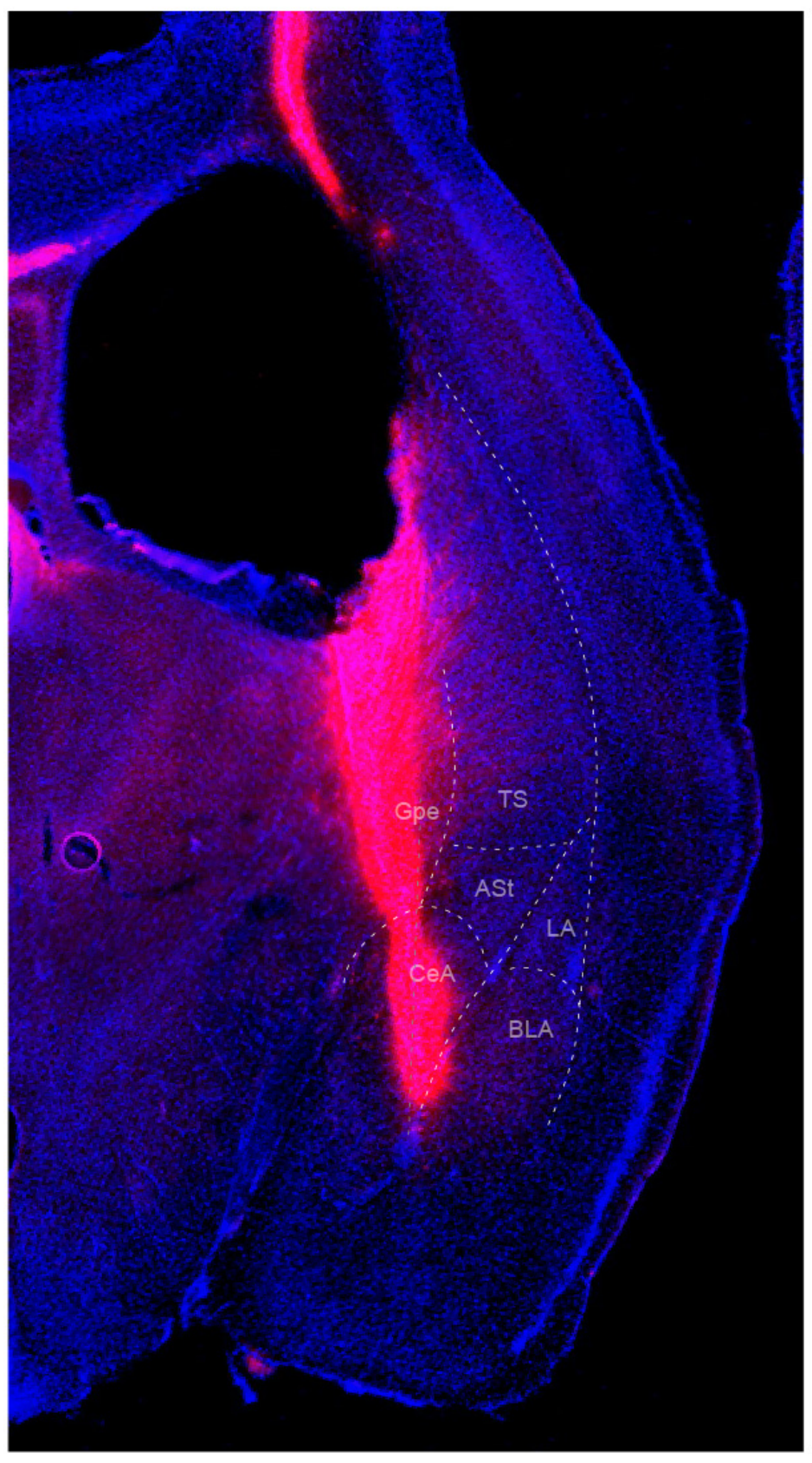

✓

Blue = DAPI
Pink = DiI (probe)

C57BL6/J Paired group "Mouse ID: 3012_innerpeace " Data included

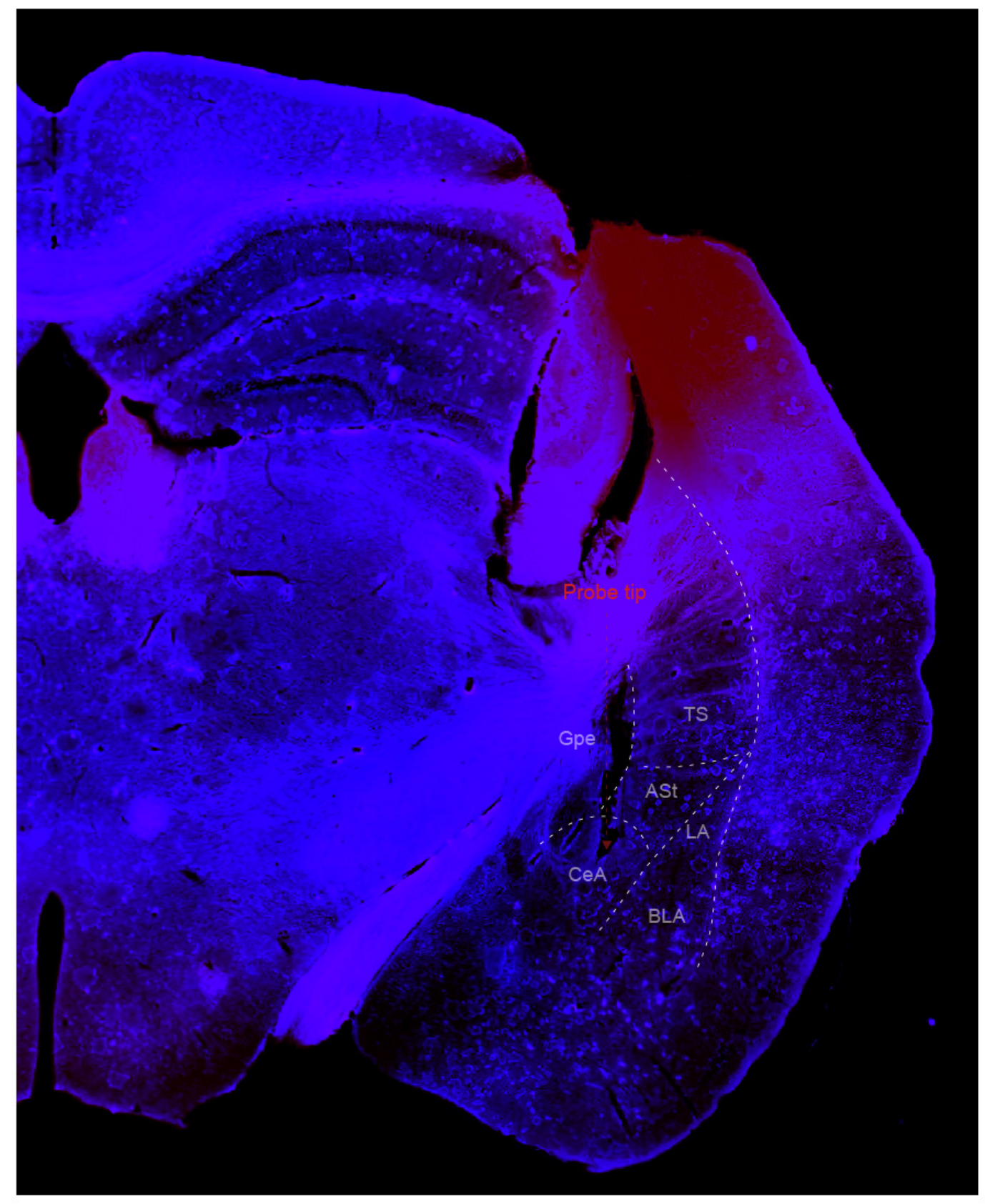

Electrolytic lesion from probe bundle tip in CeA ✓

Blue = DAPI
Red = autofluorescence (helps visualize lesion)

C57BL6/J Paired group "Mouse ID: 3014_illidan" Data included

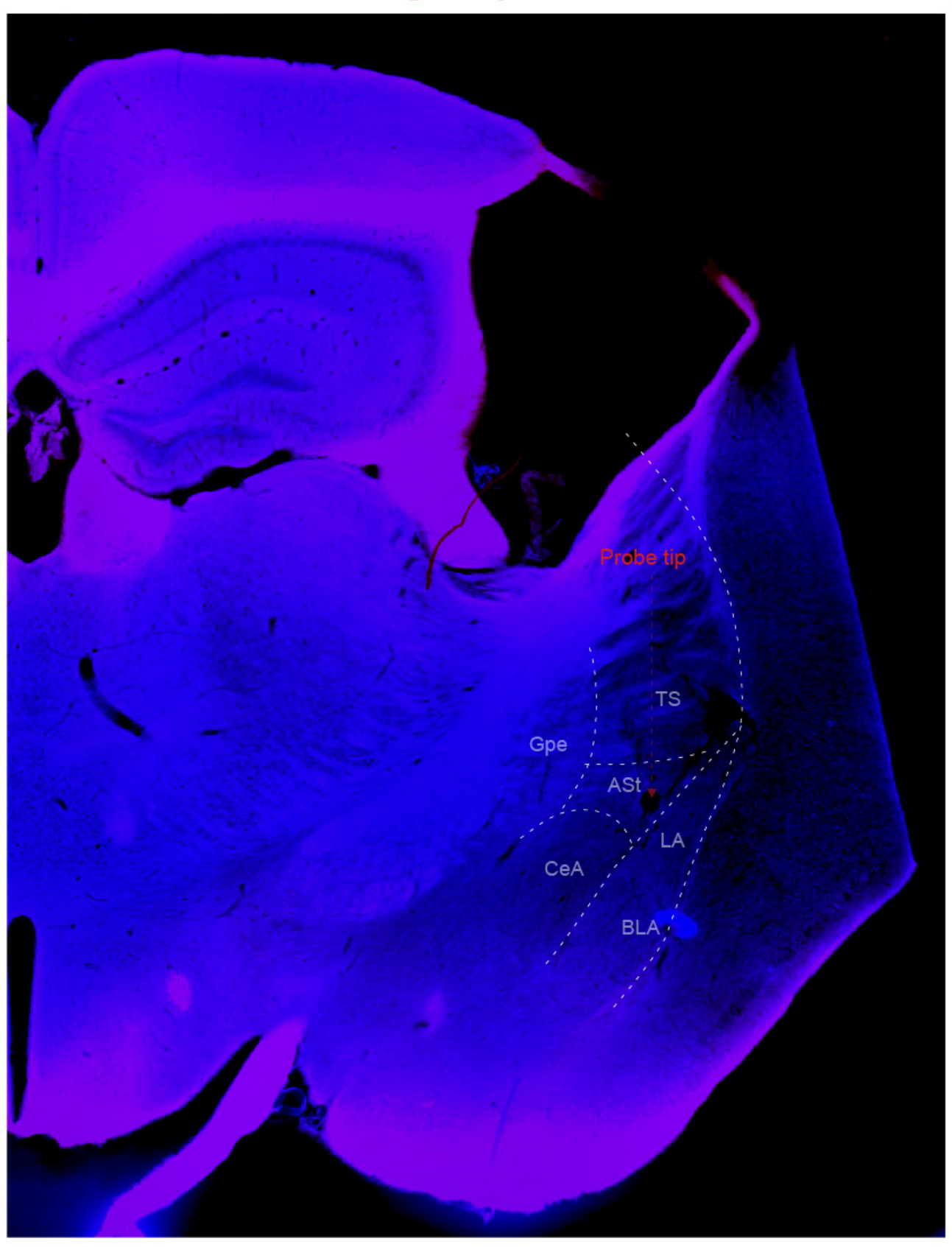

Electrolytic lesion from probe bundle tip in ASt ✓

Blue = DAPI
Red = autofluorescence (helps visualize lesion)

C57BL6/J Paired group "Mouse ID: 3016_donkey" Data included

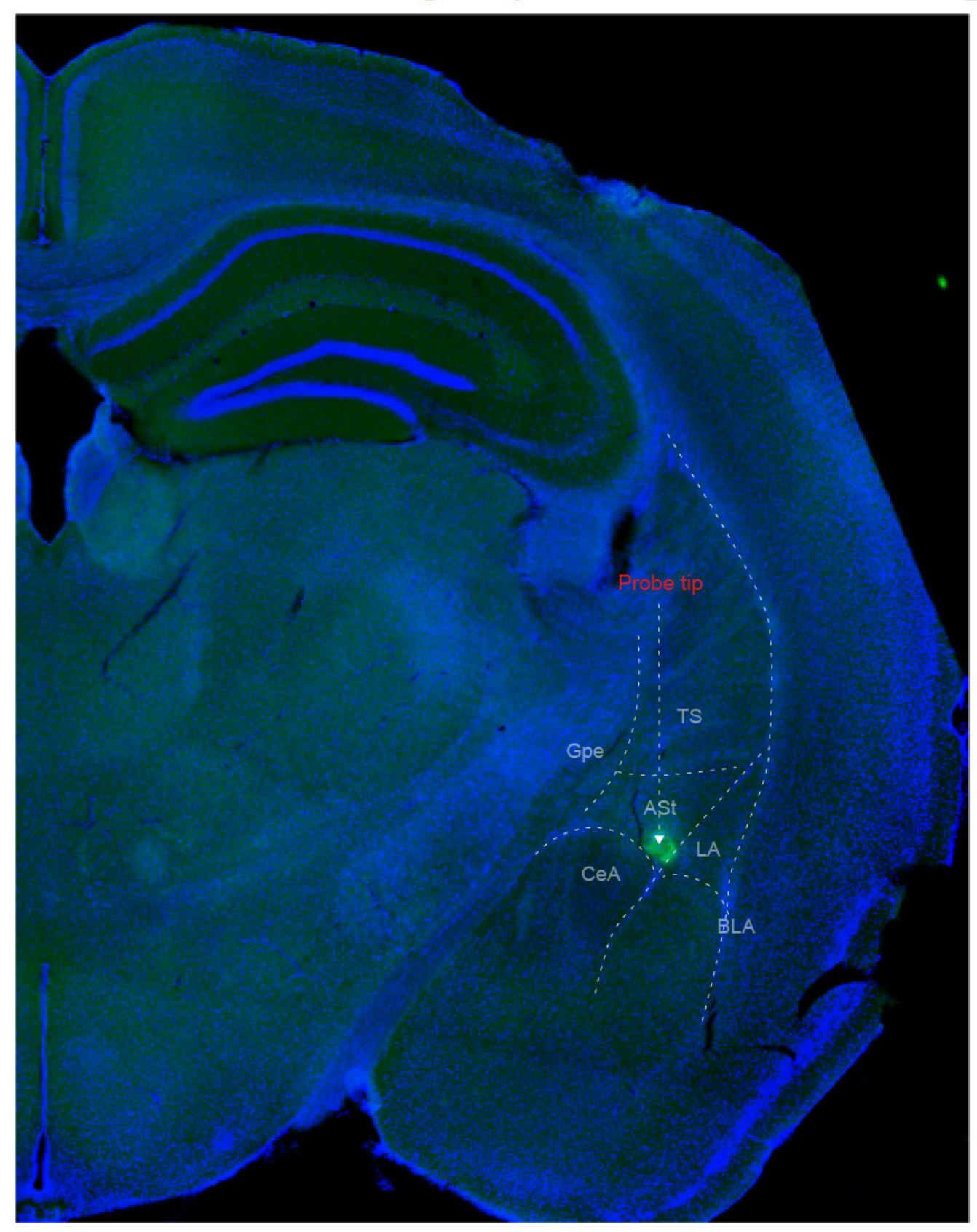

Electrolytic lesion from probe bundle tip in ASt ✓

Blue = DAPI
Green = autofluorescence (helps visualize lesion)

C57BL6/J Paired group "Mouse ID: 3024_ishmael" Data included

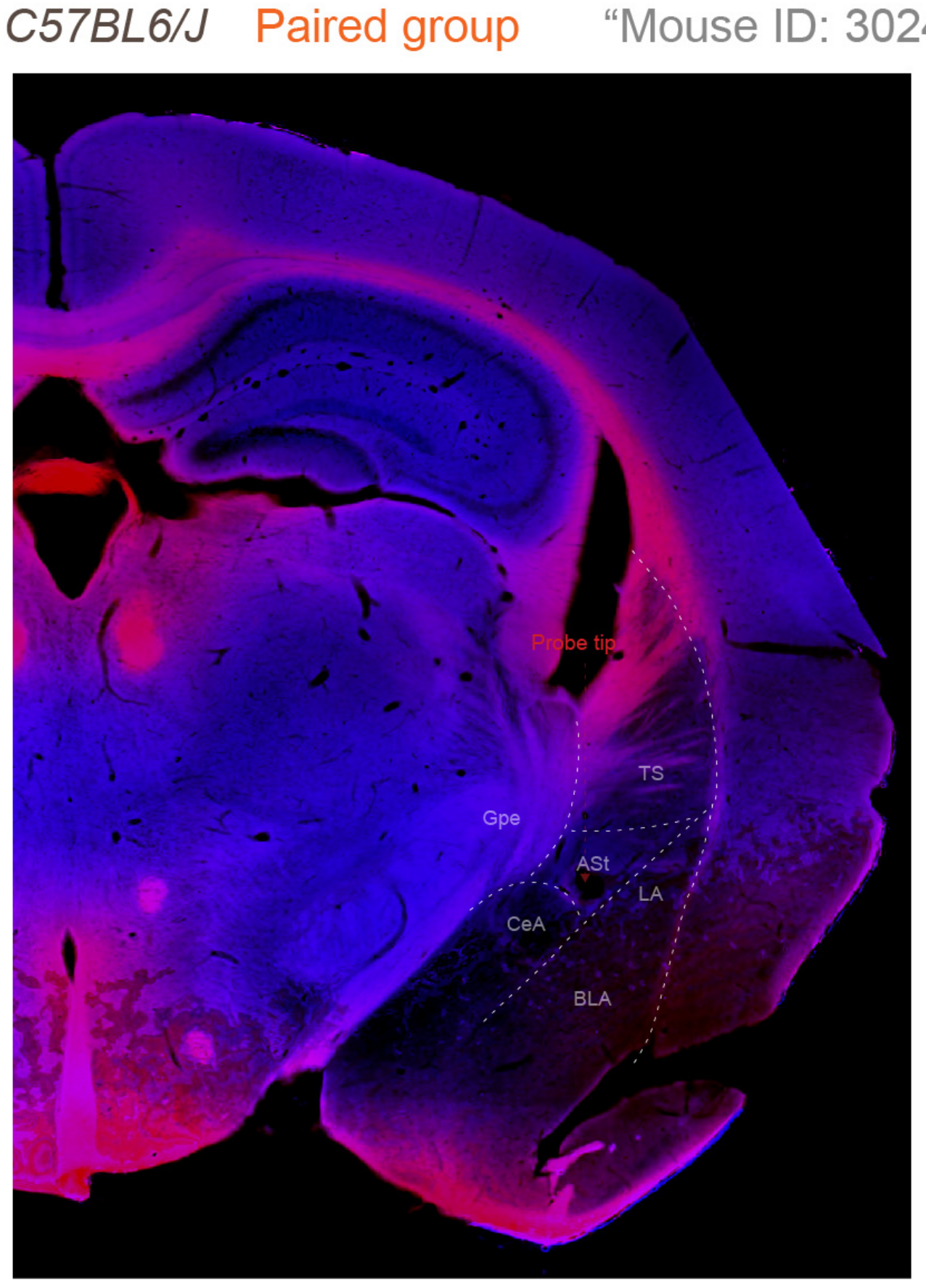

Electrolytic lesion from probe bundle tip in ASt ✓

Blue = DAPI
Red = autofluorescence (helps visualize lesion)

C57BL6/J Paired group "Mouse ID: 3026_deng" Data included

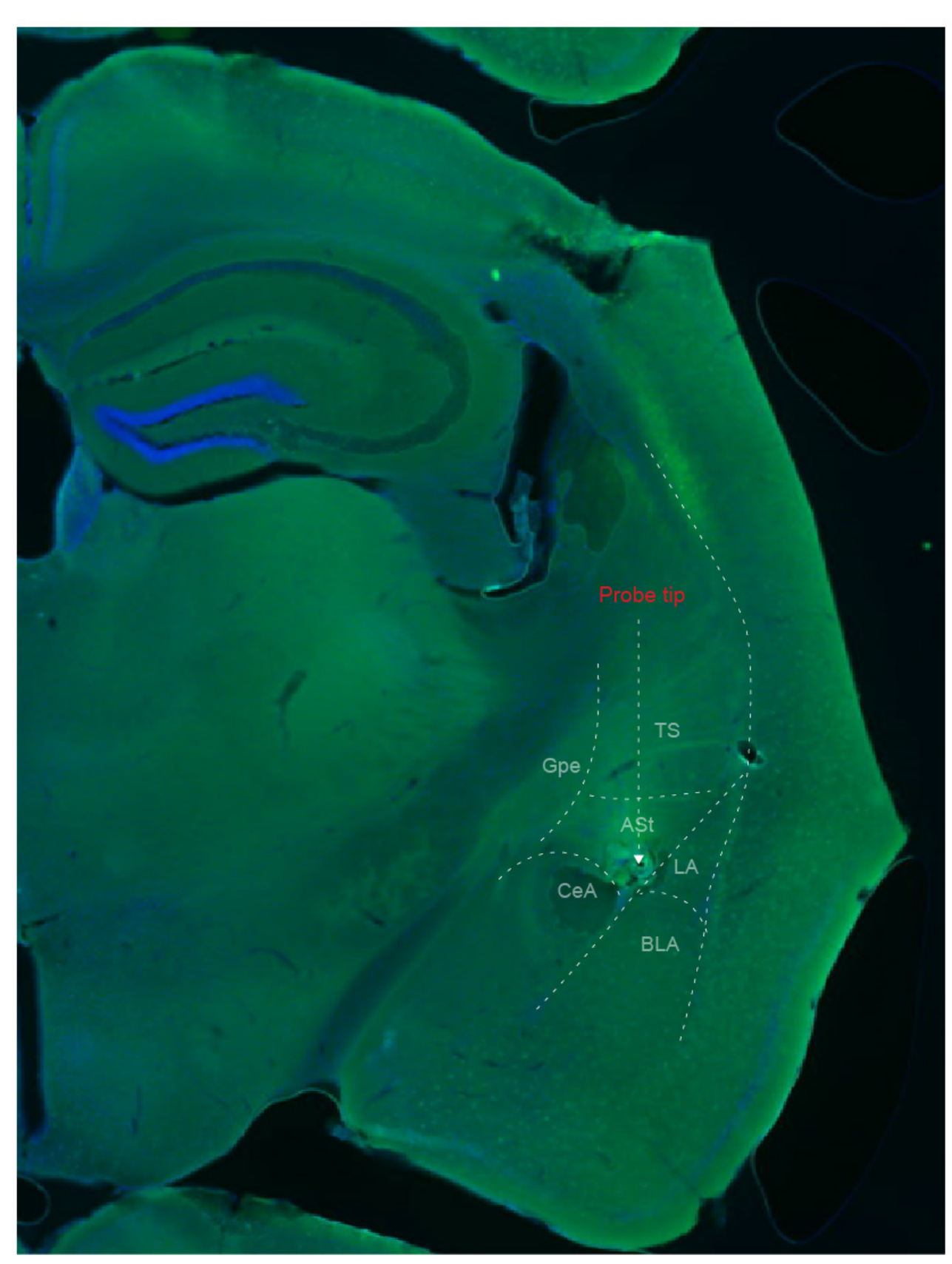

Electrolytic lesion from probe bundle tip in ASt ✓

Blue = DAPI
Green = autofluorescence (helps visualize lesion)

Appendix A - Histology (Electrophysiology)

C57BL6/J Paired group "Mouse ID: 3029_iouxio" Data included

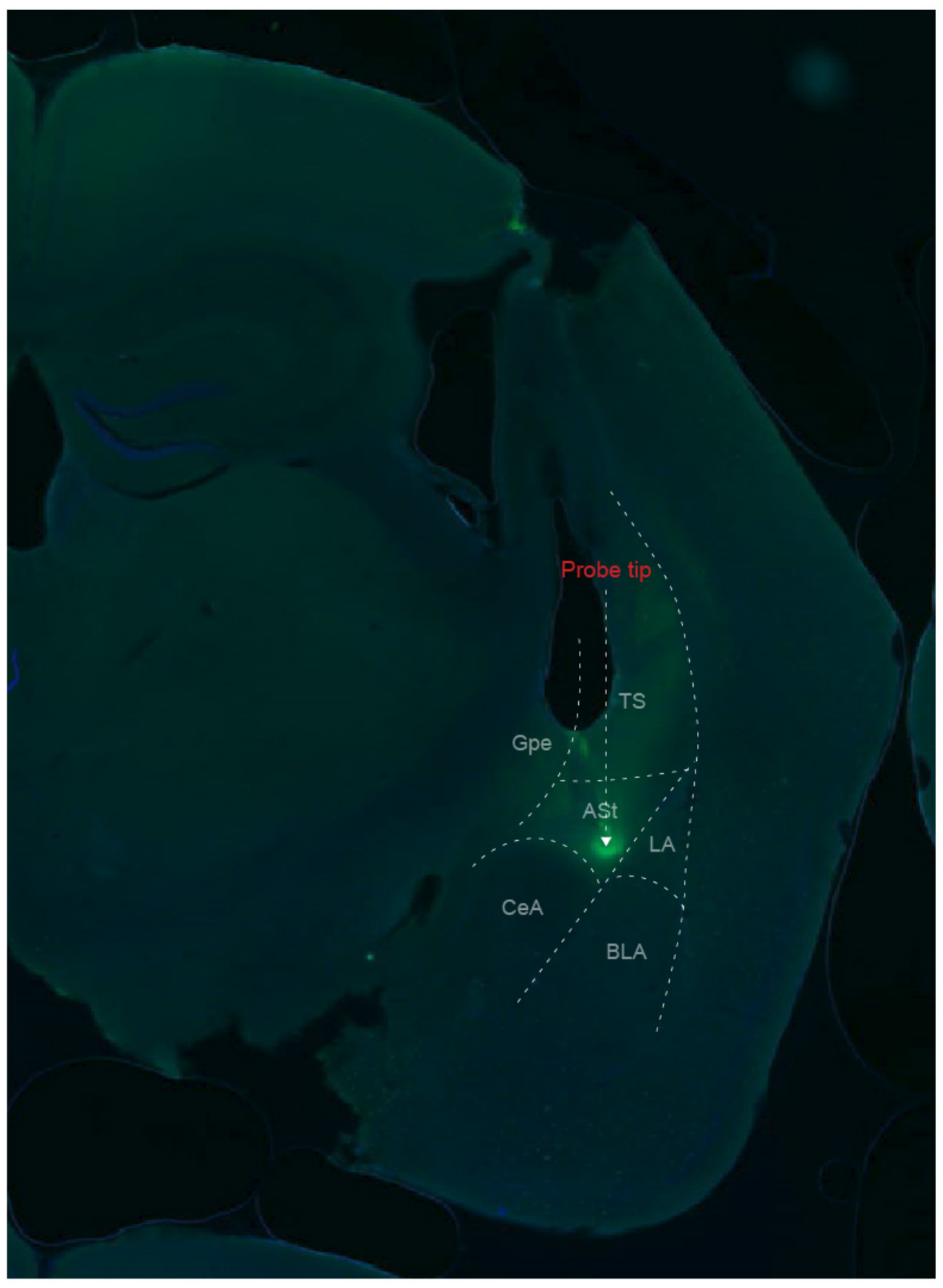

Electrolytic lesion from probe bundle tip in ASt ✓

Blue = DAPI
Green = autofluorescence (helps visualize lesion)

C57BL6/J Paired group "Mouse ID: 6197_daijobu" Data included

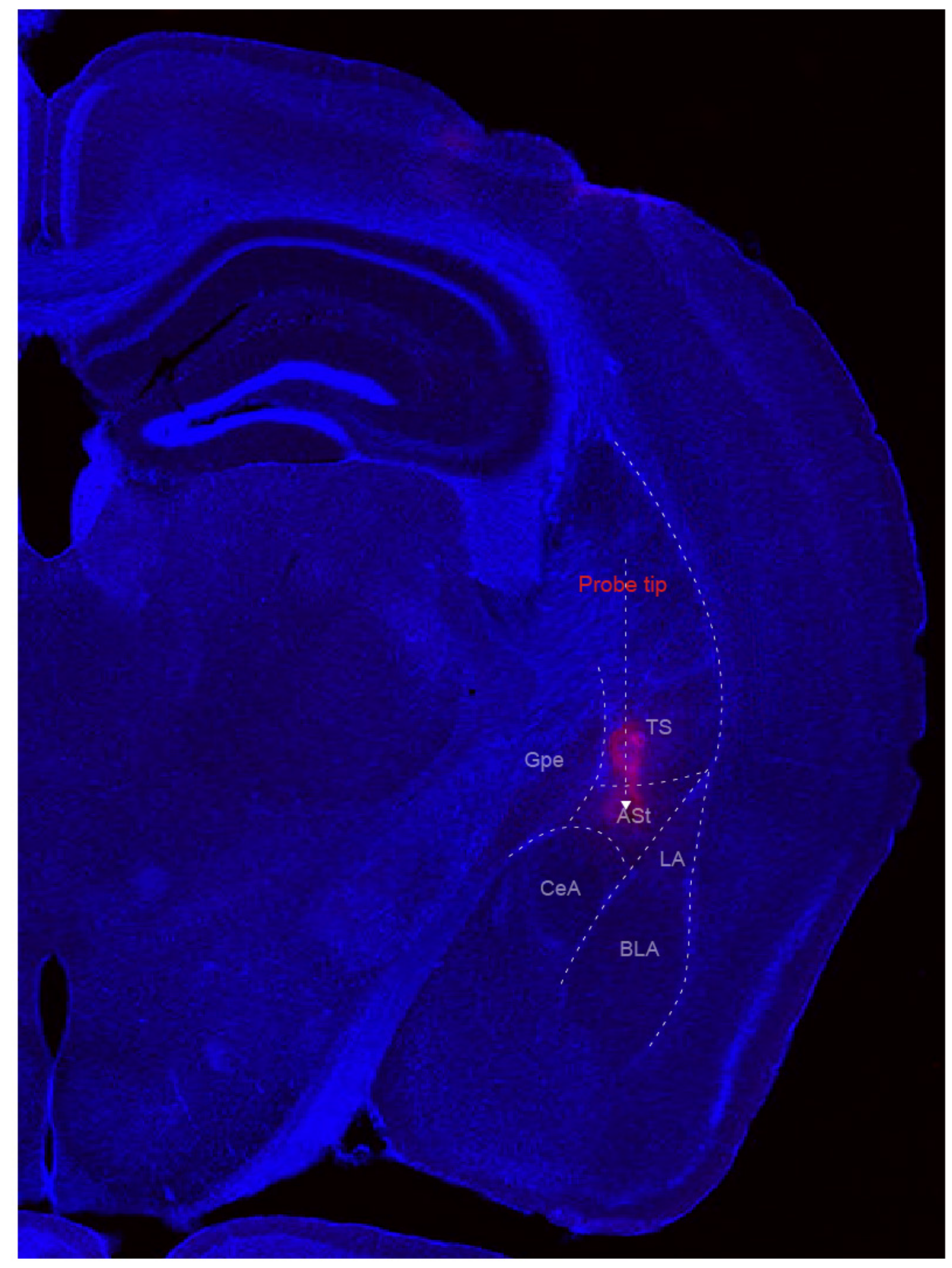

Electrolytic lesion from probe bundle tip in ASt ✓

Blue = DAPI
Red = autofluorescence (helps visualize lesion)

C57BL6/J Paired group "Mouse ID: 6253_daisy" Data included

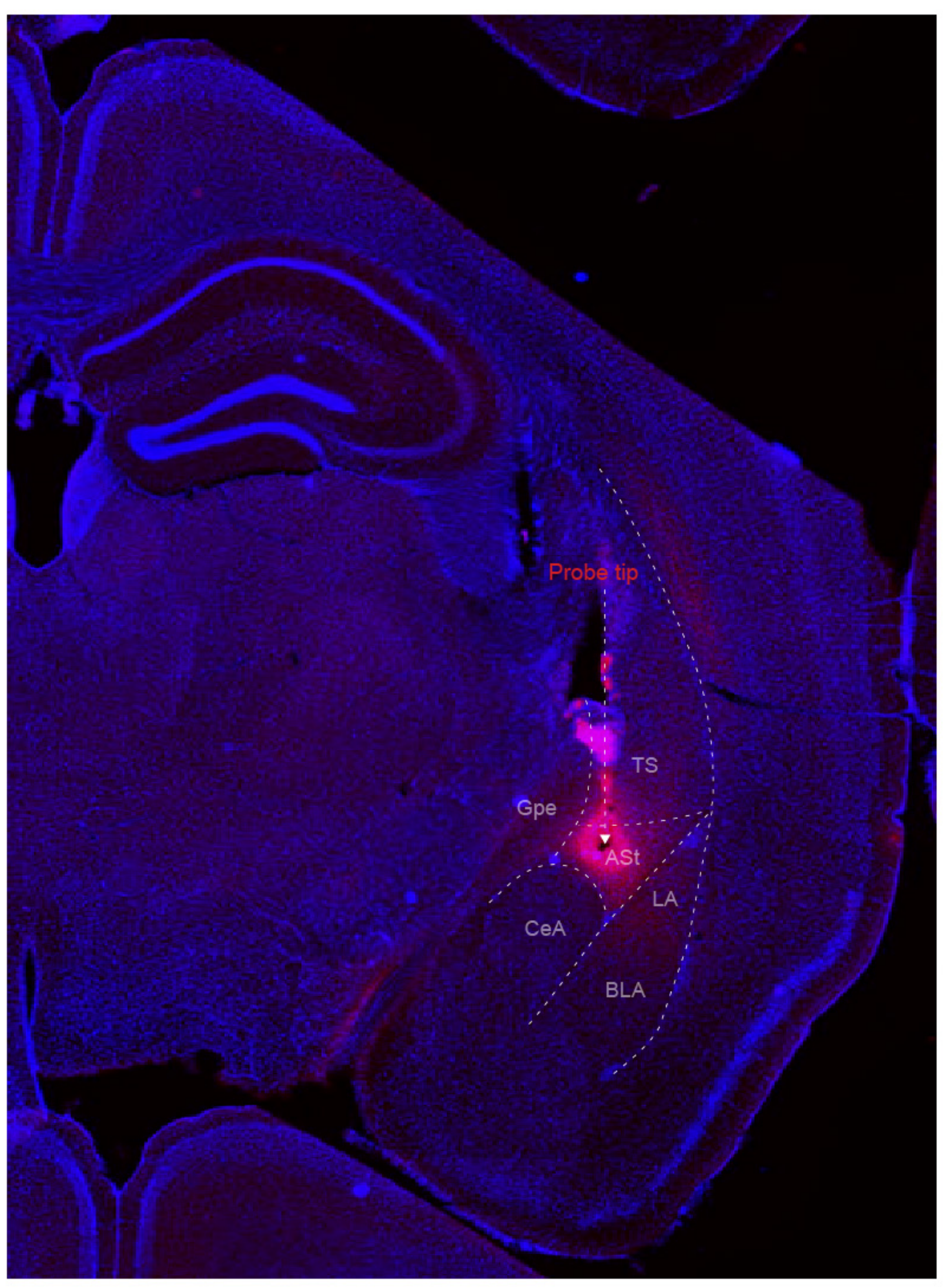

Electrolytic lesion from probe bundle tip in ASt ✓

Blue = DAPI
Red = autofluorescence (helps visualize lesion)

C57BL6/J Paired group "Mouse ID: 6429_durian" Data included

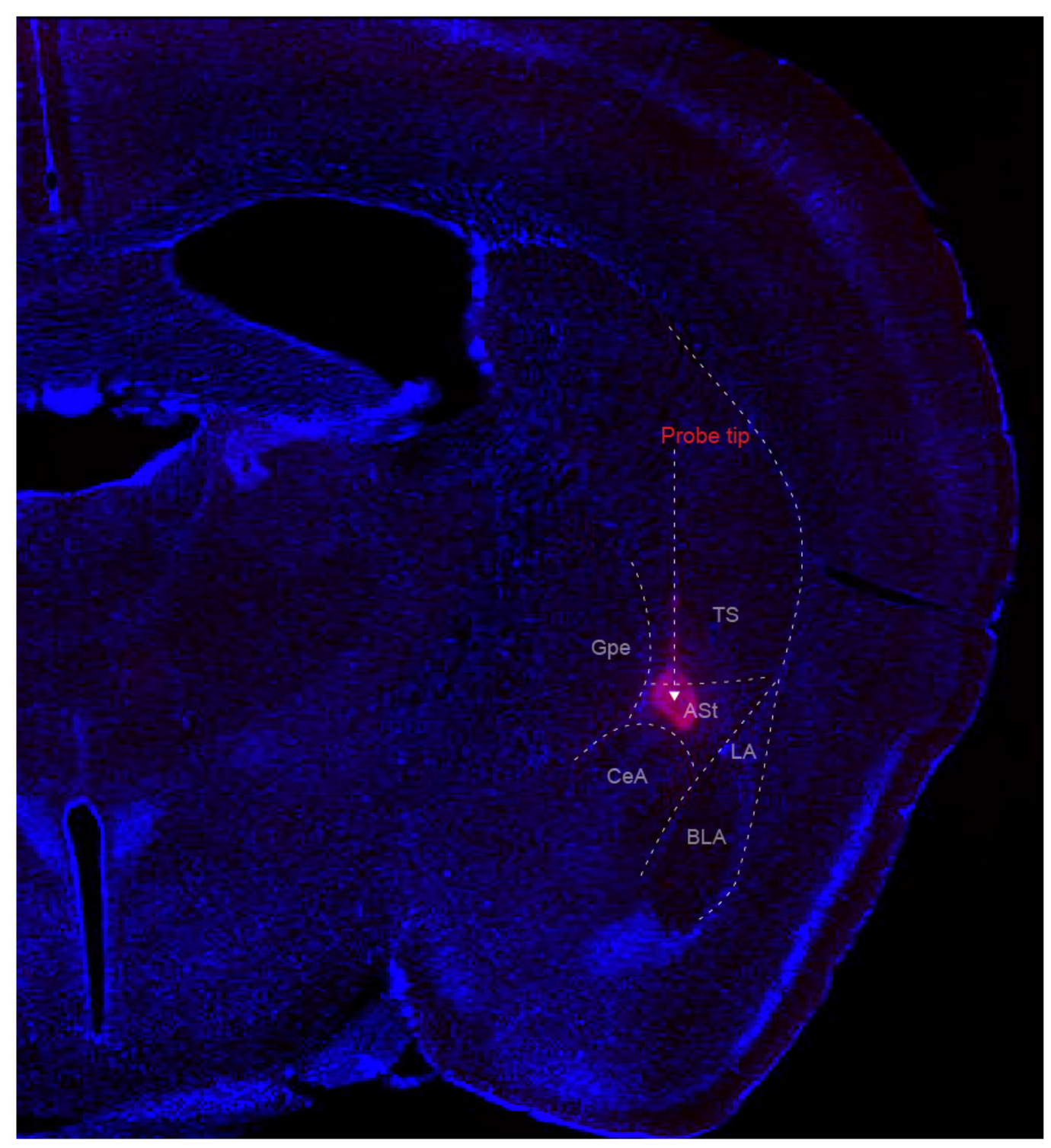

Electrolytic lesion from probe bundle tip in ASt ✓

Blue = DAPI
Red = autofluorescence (helps visualize lesion)

C57BL6/J Paired group "Mouse ID: 7008_ikari" Data included

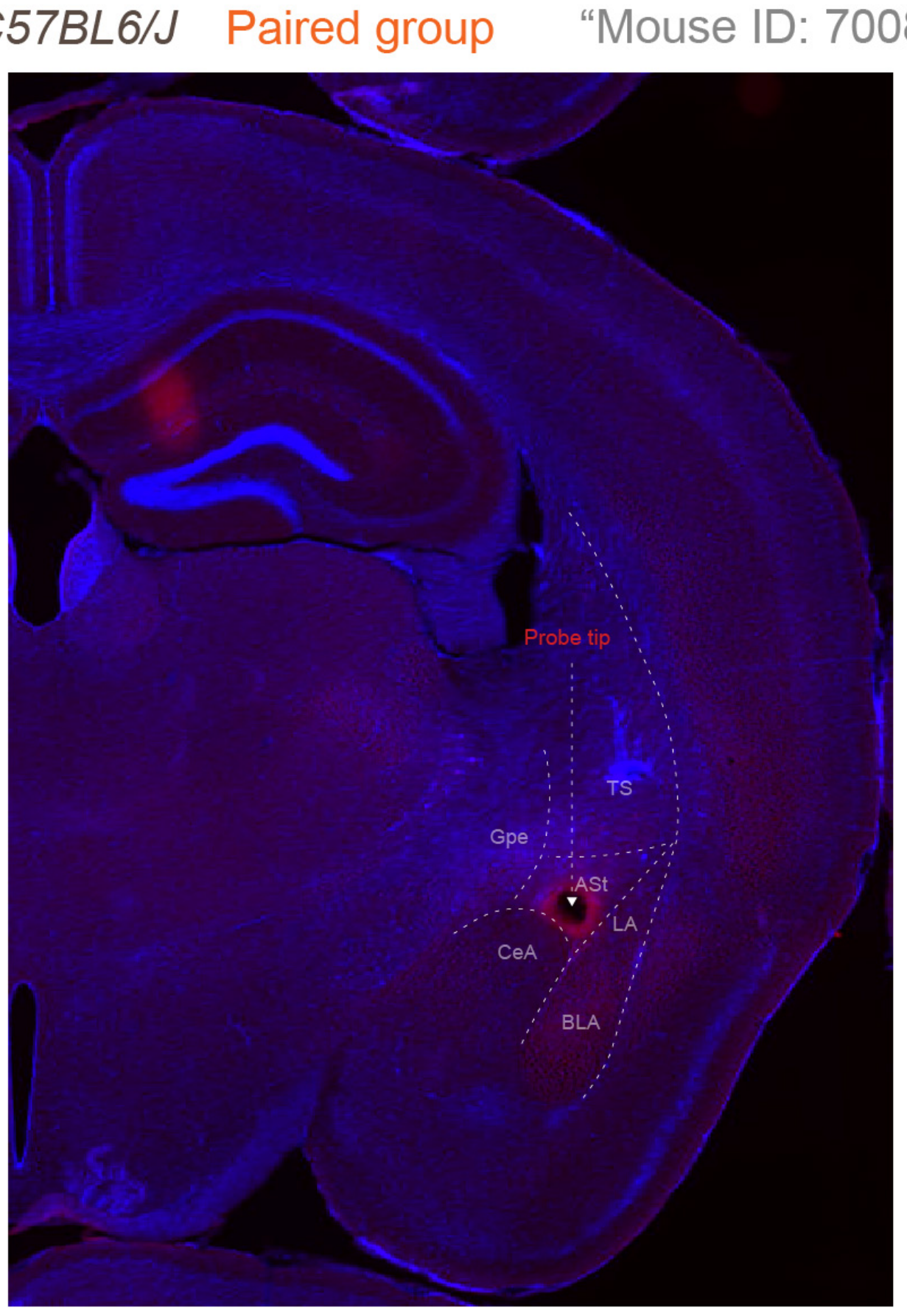

Electrolytic lesion from probe bundle tip in ASt ✓

Blue = DAPI
Red = autofluorescence (helps visualize lesion)

C57BL6/J Paired group "Mouse ID: 7049_ivy" Data included

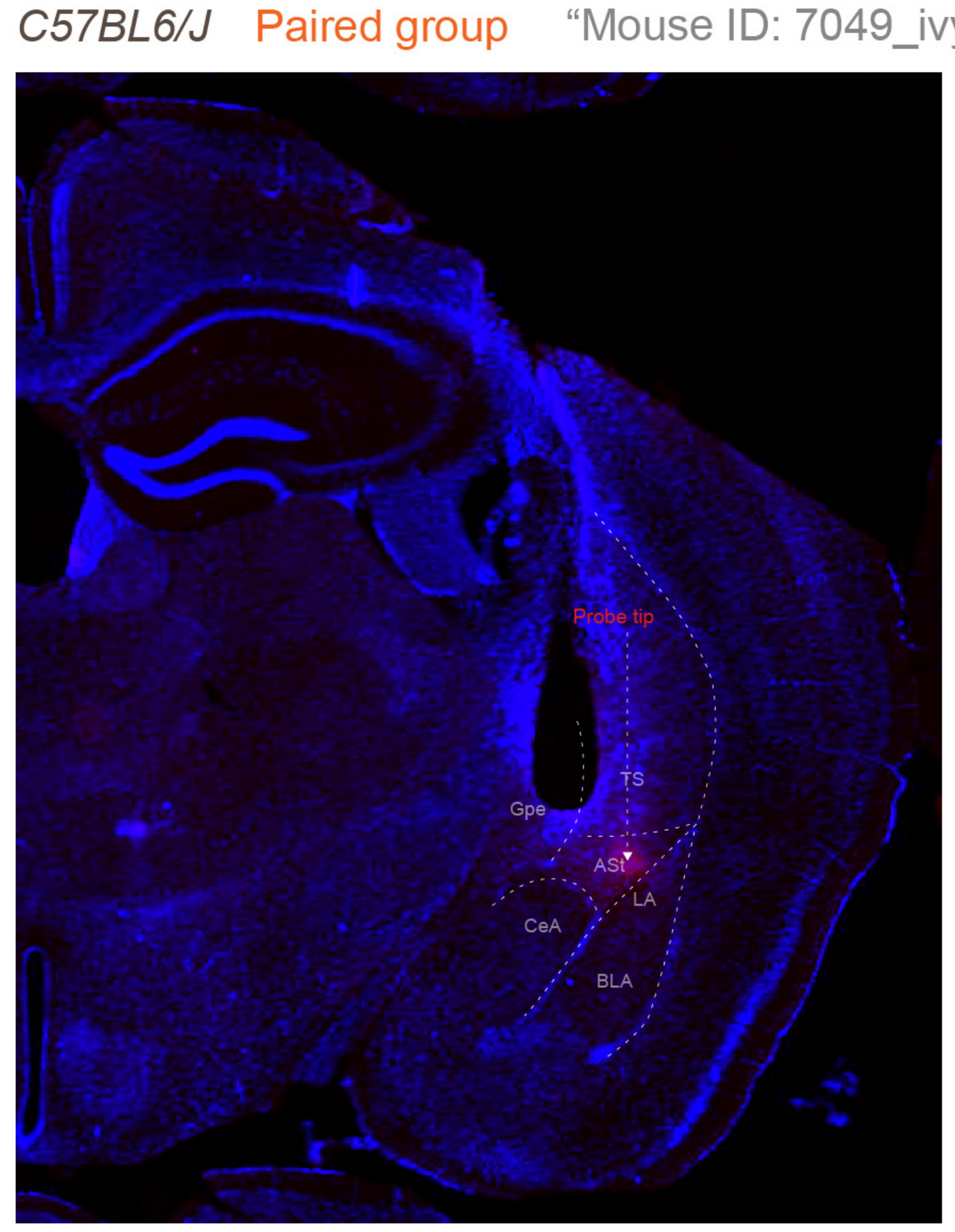

Electrolytic lesion from probe bundle tip in ASt ✓

Blue = DAPI
Red = autofluorescence (helps visualize lesion)

Appendix A - Histology (Electrophysiology)

C57BL6/J Paired group "Mouse ID: 7001_ichimoku" Data included

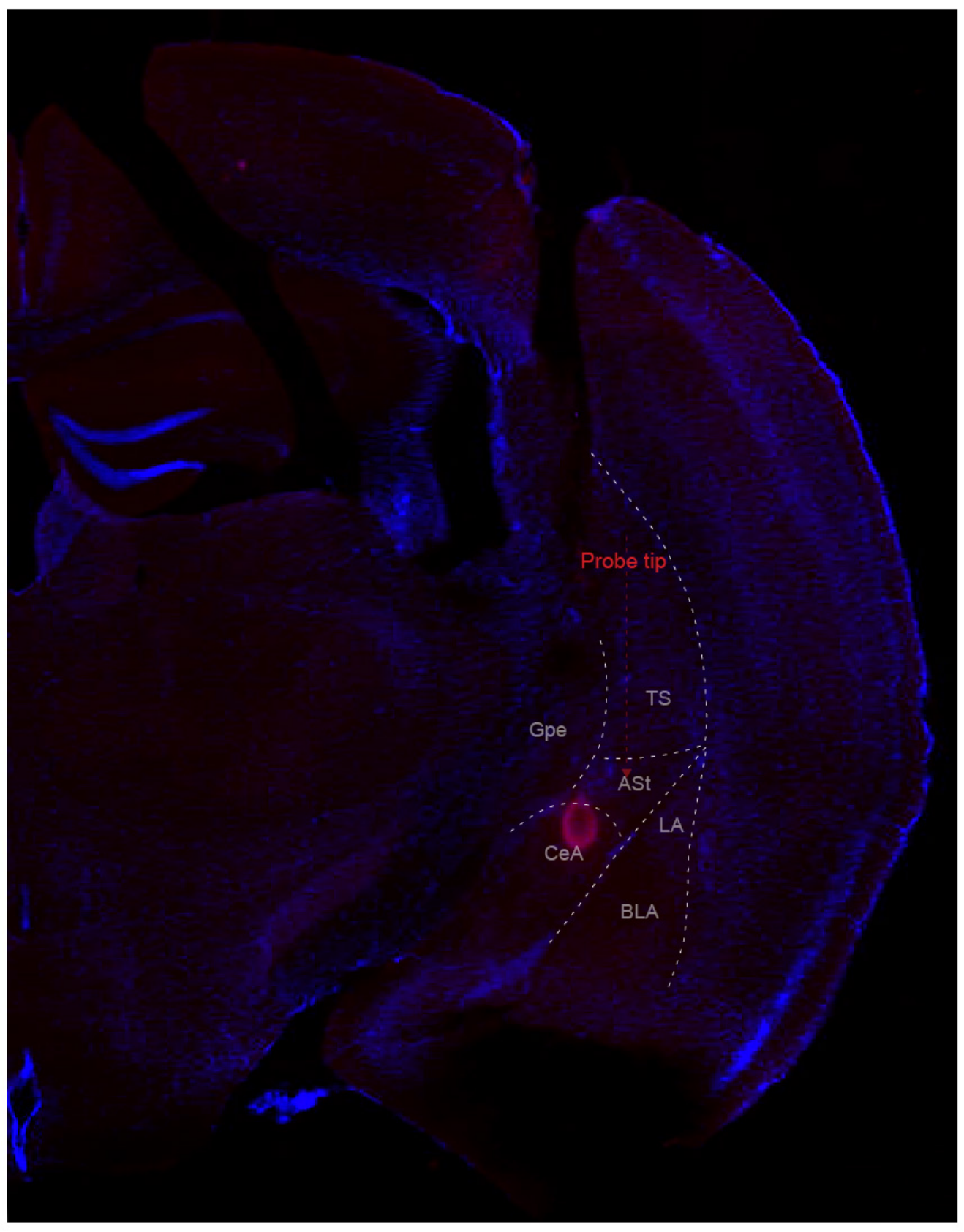

Electrolytic lesion from probe bundle tip in CeA

✓

Blue = DAPI
Red = autofluorescence (helps visualize lesion)

Mills et al. 2026

Data S1 - Appendix B

Histology for *VGAT*-Cre ChR2 mice

Appendix B - Histology - *VGAT:Cre* mice, ChR2 experiments:

VGAT - ChR2 Cohort

"Mouse ID: C701A16L"

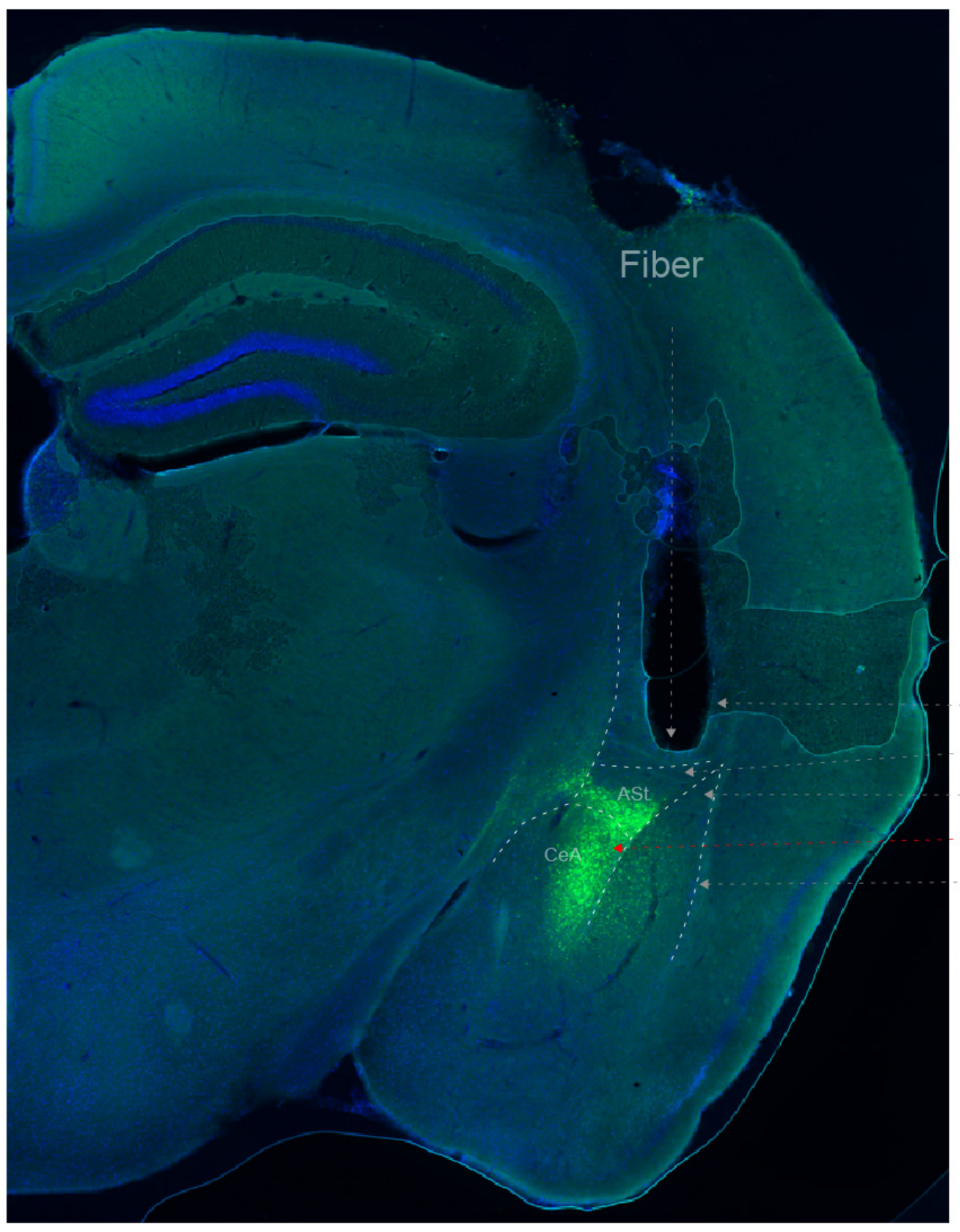

Data from this mouse were not included in the study

- TS: No expression ✓
- ASt: Ok expression ✓
- LA: Low expression ✓
- CeA: Expression ✗
- BLA: Med. expression ~

VGAT - ChR2 Cohort

"Mouse ID: C803A09R"

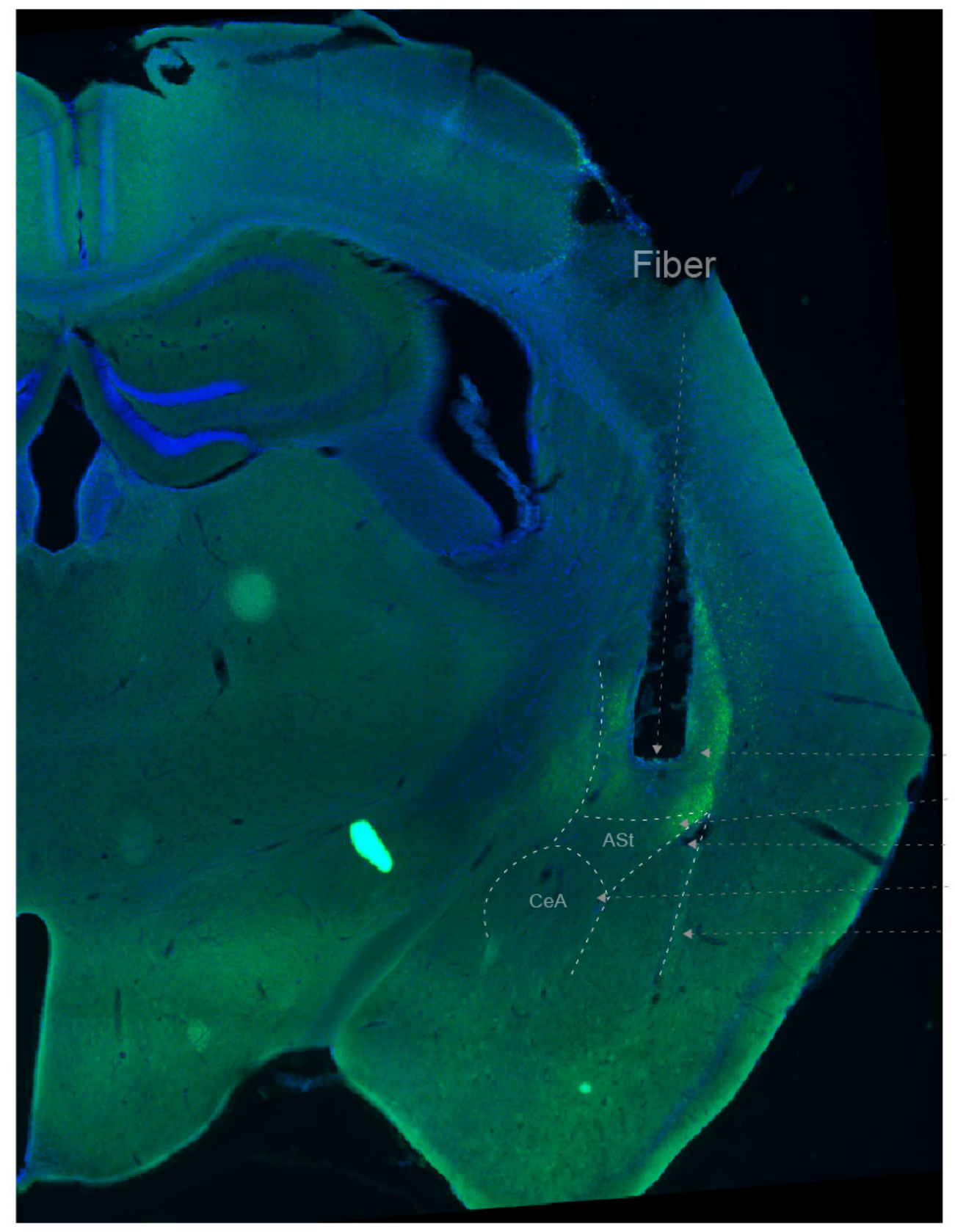

Data from this mouse were not included in the study

- TS: Some expression ✓
- ASt: Low expression ✗
- LA: Low expression ✓
- CeA: No Expression ✓
- BLA: No expression ✓

Note: background green color due to autofluorescence

VGAT - ChR2 Cohort

"Mouse ID: C803A02L"

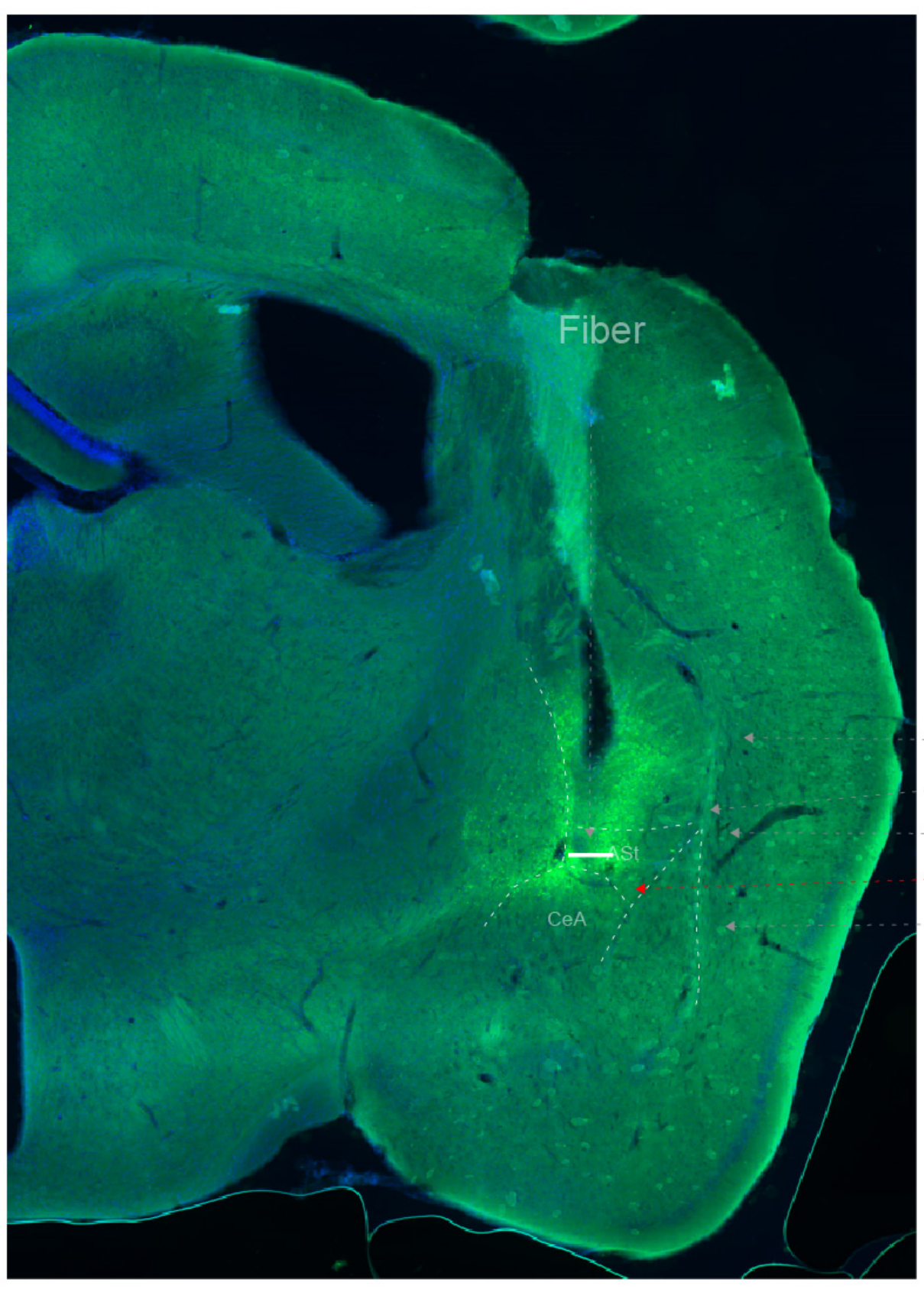

Data from this mouse were not included in the study

- TS: Some expression ~
- ASt: Ok expression ✓
- LA: Low expression ✓
- CeA: Expression ✗
- BLA: Low expression ✓

Note: background green color due to autofluorescence

VGAT - ChR2 Cohort

"Mouse ID: C703A18L"

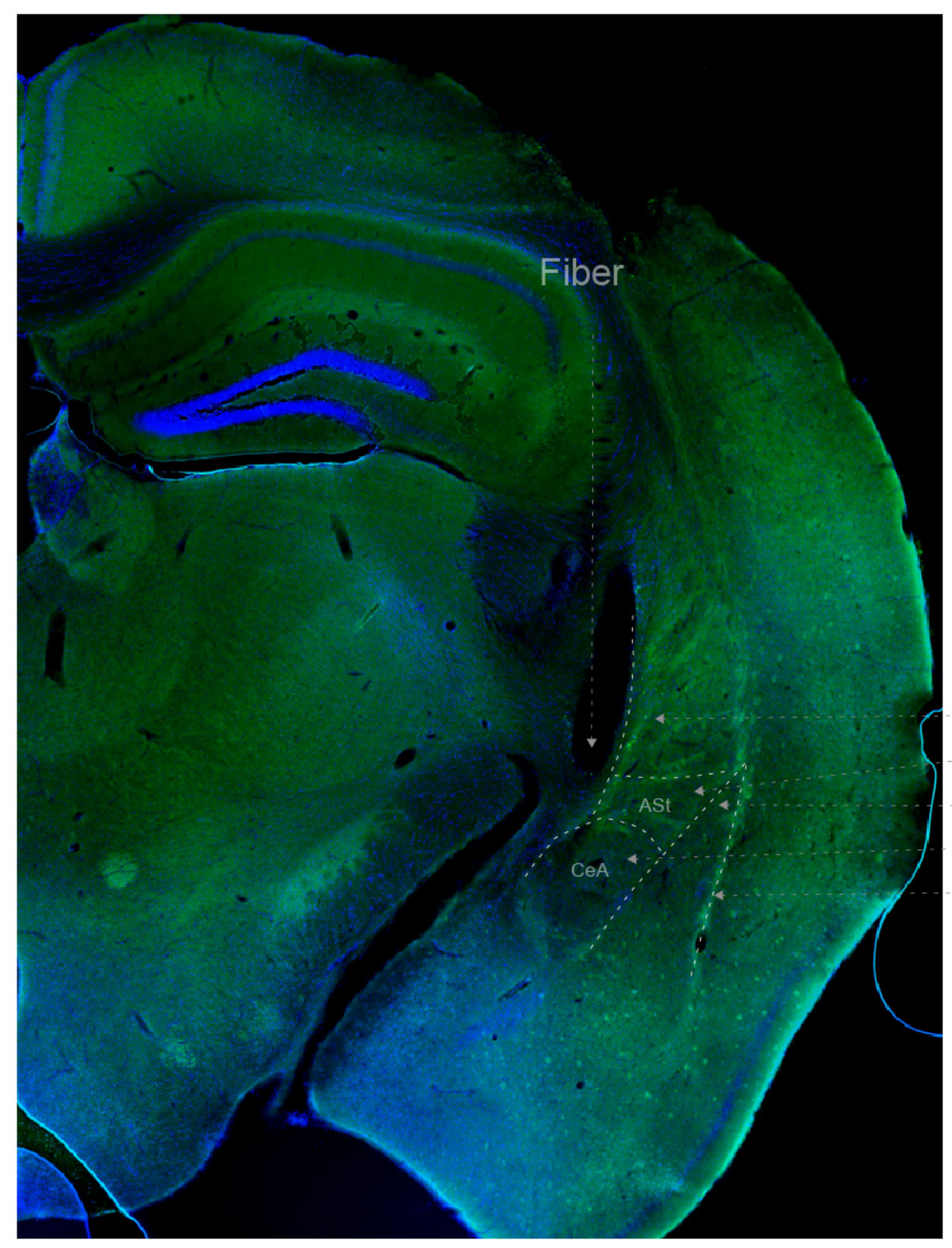

Data from this mouse were not included in the study

- TS: Almost no expression ✓
- ASt: Almost no expression ✗
- LA: Low expression ✓
- CeA: No expression ✓
- BLA: No expression ✓

Note: background green color due to autofluorescence

VGAT - ChR2 Cohort

"Mouse ID: C405A07L"

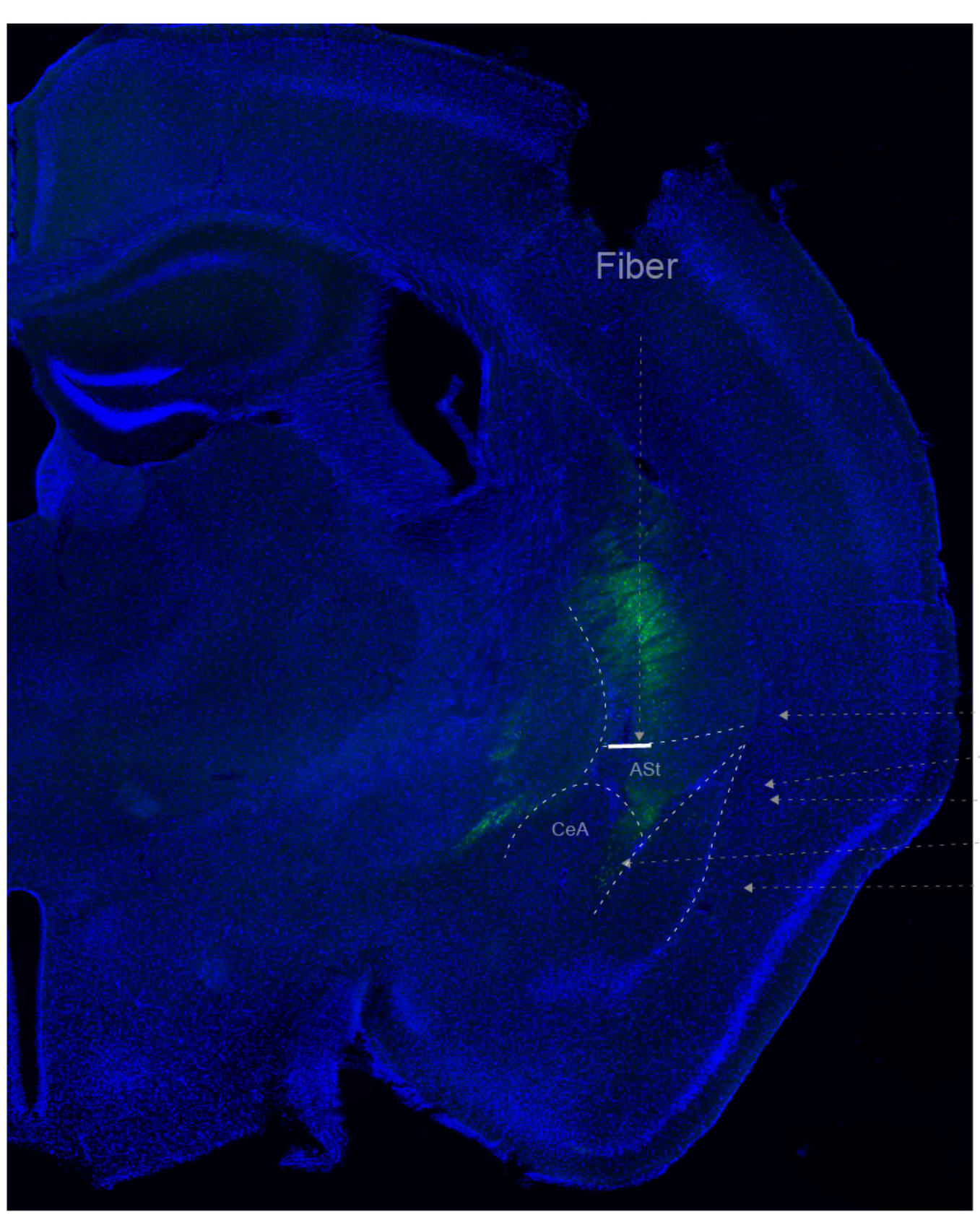

Data from this mouse were not included in the study

- TS: Some expression ✓
- ASt: Ok expression ✓
- LA: Low expression ✓
- CeA: Very slight leak ✗
- BLA: No expression ✓

VGAT - ChR2 Cohort

"Mouse ID: C601A05L"

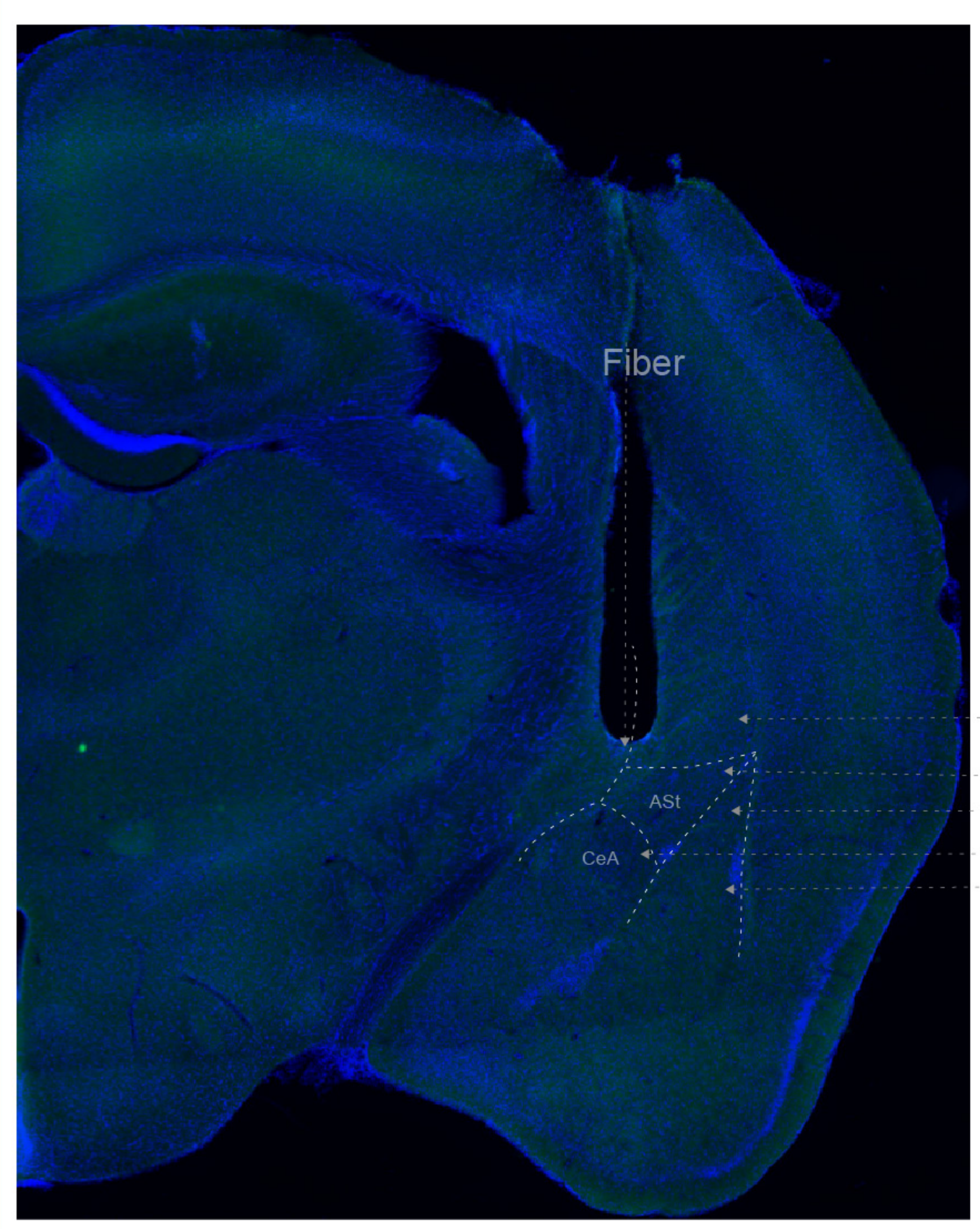

Data from this mouse were not included in the study

- TS: Low expression ✓
- ASt: No expression ✗
- LA: No expression ✓
- CeA: No expression ✓
- BLA: No expression ✓

Appendix B - Histology - *VGAT:Cre* mice, ChR2 experiments:

VGAT - ChR2 Cohort

"Mouse ID: C503A13R"

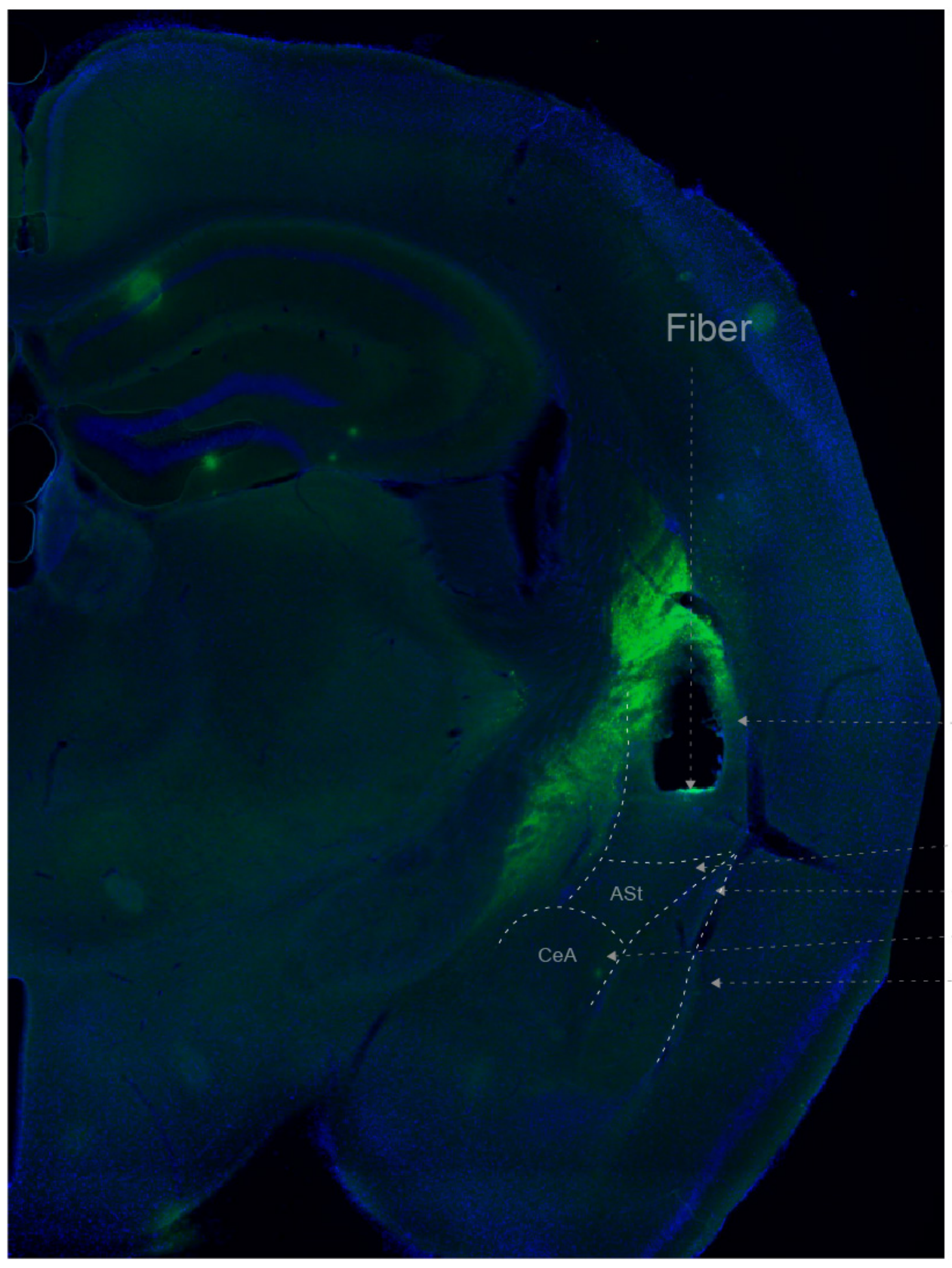

Data from this mouse were not included in the study

- TS: Excess expression but dorsal to fiber ~
- ASt: No expression ✗
- LA: No expression ✓
- CeA: No Expression ✓
- BLA: No expression ✓

VGAT - ChR2 Cohort

"Mouse ID: C501A08L"

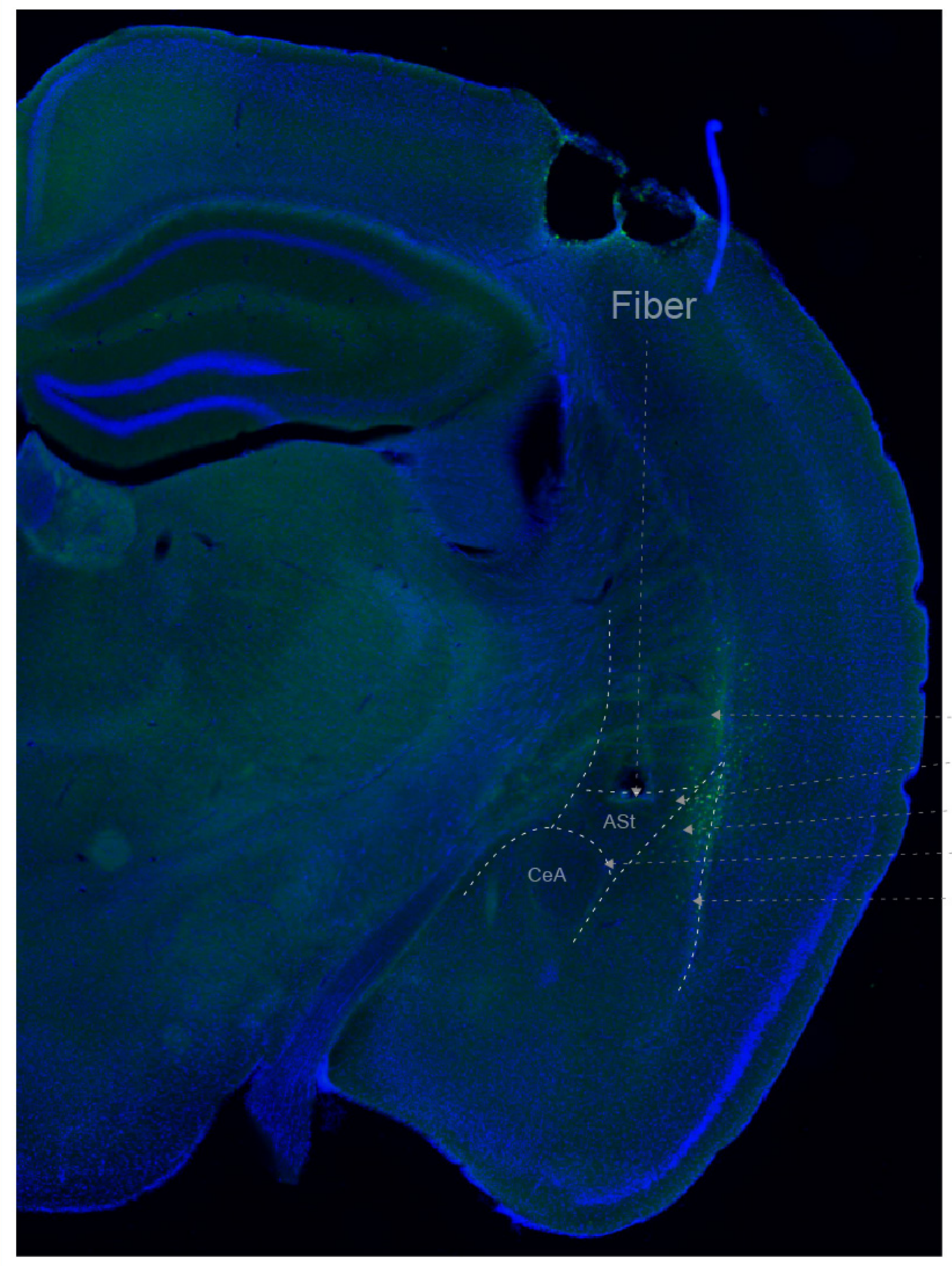

Data from this mouse were not included in the study

- TS: Some expression ✓
- ASt: Very low expression ✗
- LA: Low expression ✓
- CeA: No Expression ✓
- BLA: Very low expression ✓

VGAT - ChR2 Cohort

"Mouse ID: C401A08R"

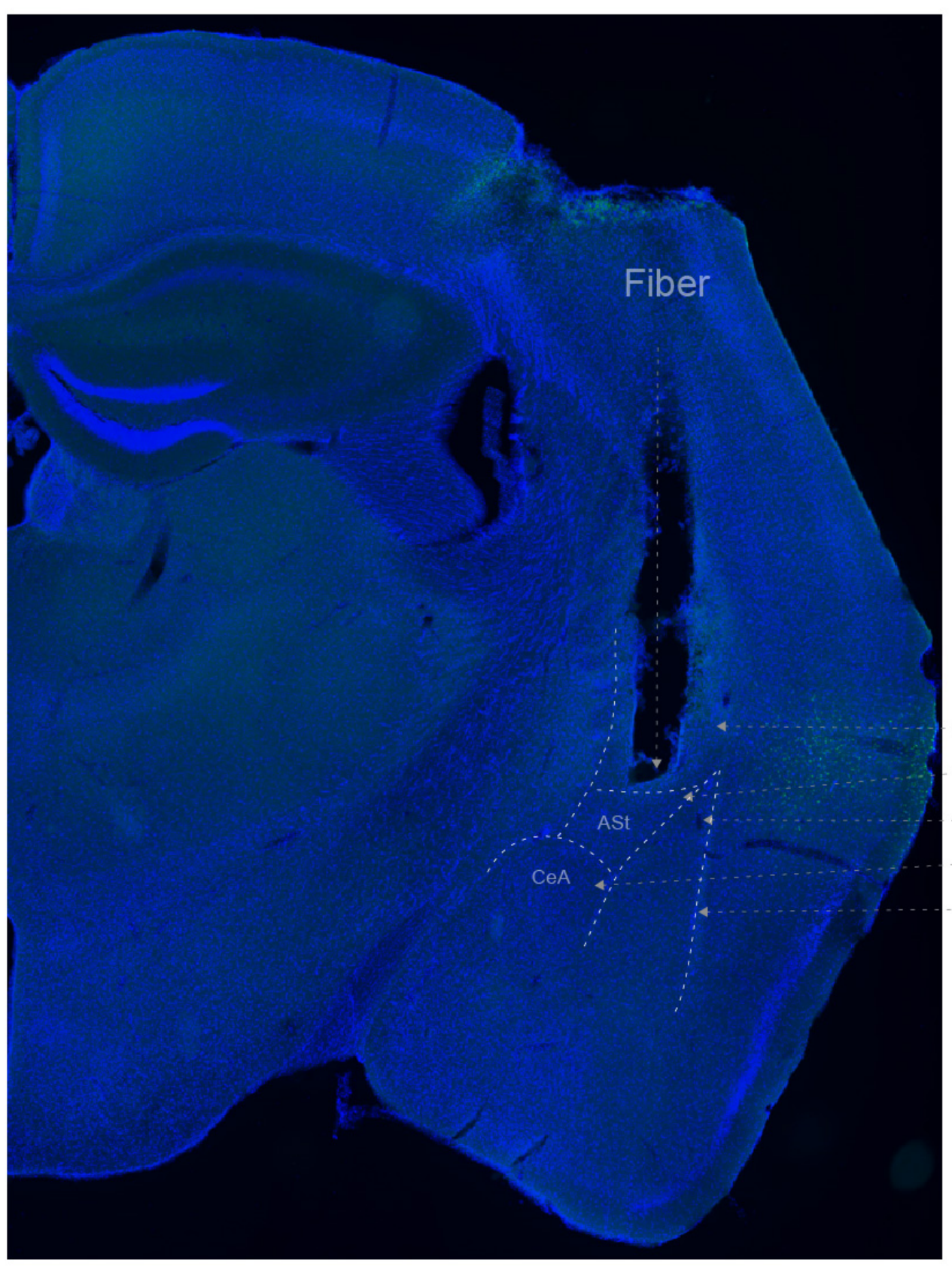

Data from this mouse were not included in the study

- TS: Faint expression ✓
- ASt: No expression ✗
- LA: Low expression ✓
- CeA: No Expression ✓
- BLA: No expression ✓

VGAT - ChR2 Cohort

"Mouse ID: C403A06R"

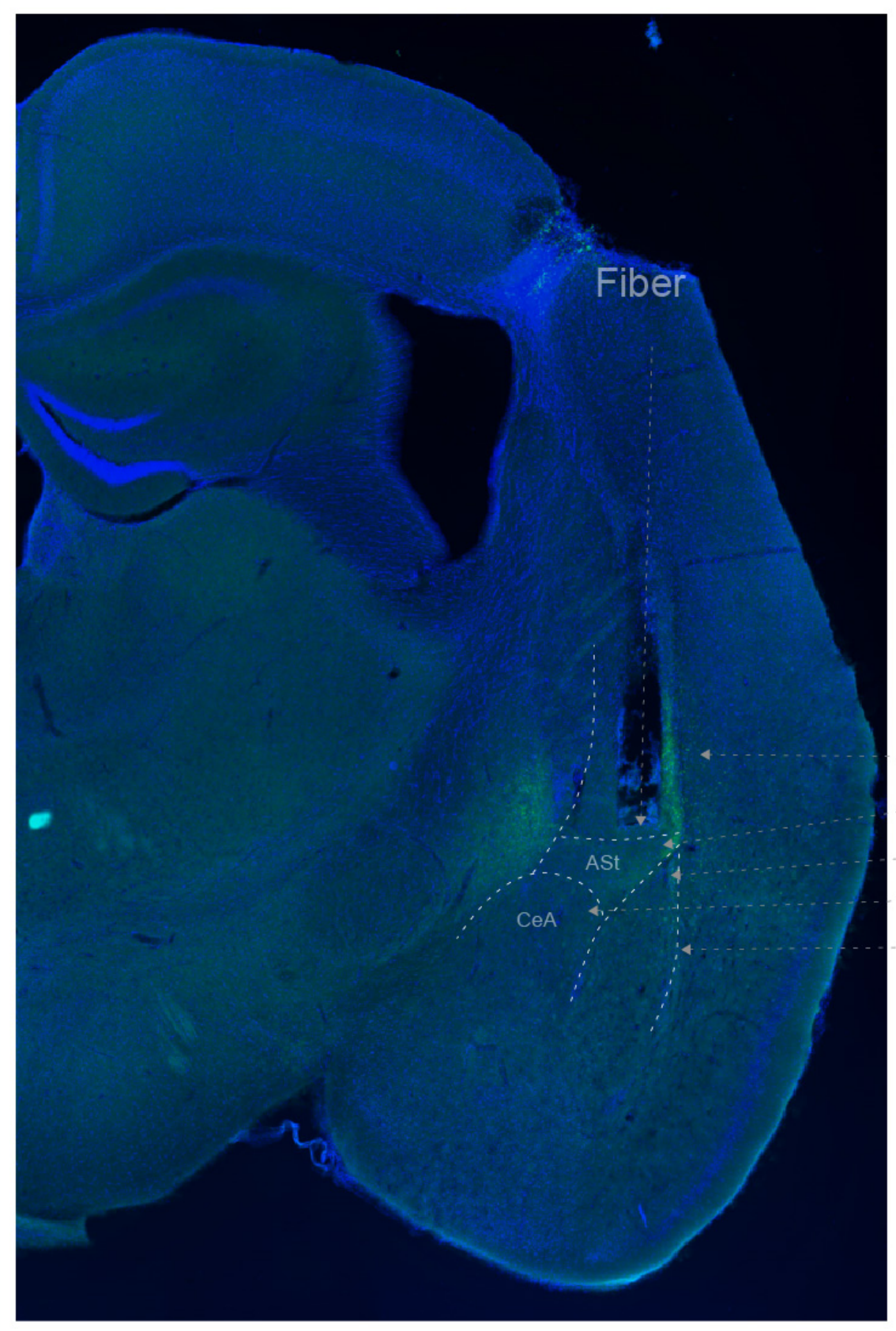

Data from this mouse were not included in the study

- TS: Some expression ✓
- ASt: Very low expression ✗
- LA: Low expression ✓
- CeA: Expression ✓
- BLA: Med. expression ✓

VGAT - ChR2 Cohort

"Mouse ID: C703A20R"

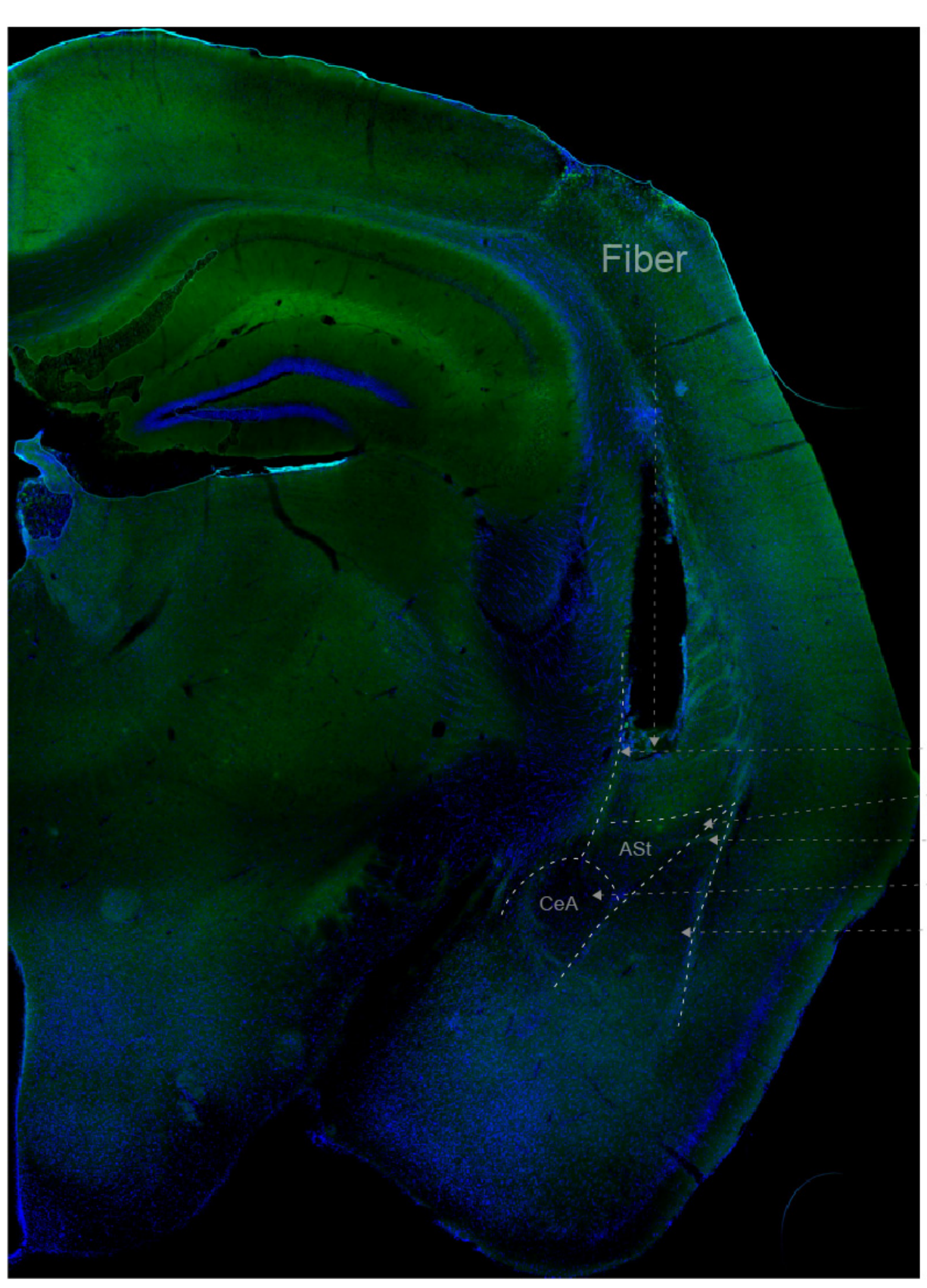

Data from this mouse were not included in the study

- TS: Some expression ✓
- ASt: Low expression ✗
- LA: No expression ✓
- CeA: No expression ✓
- BLA: No expression ✓

Note: background green color due to autofluorescence

VGAT - ChR2 Cohort

"Mouse ID: C801A10R"

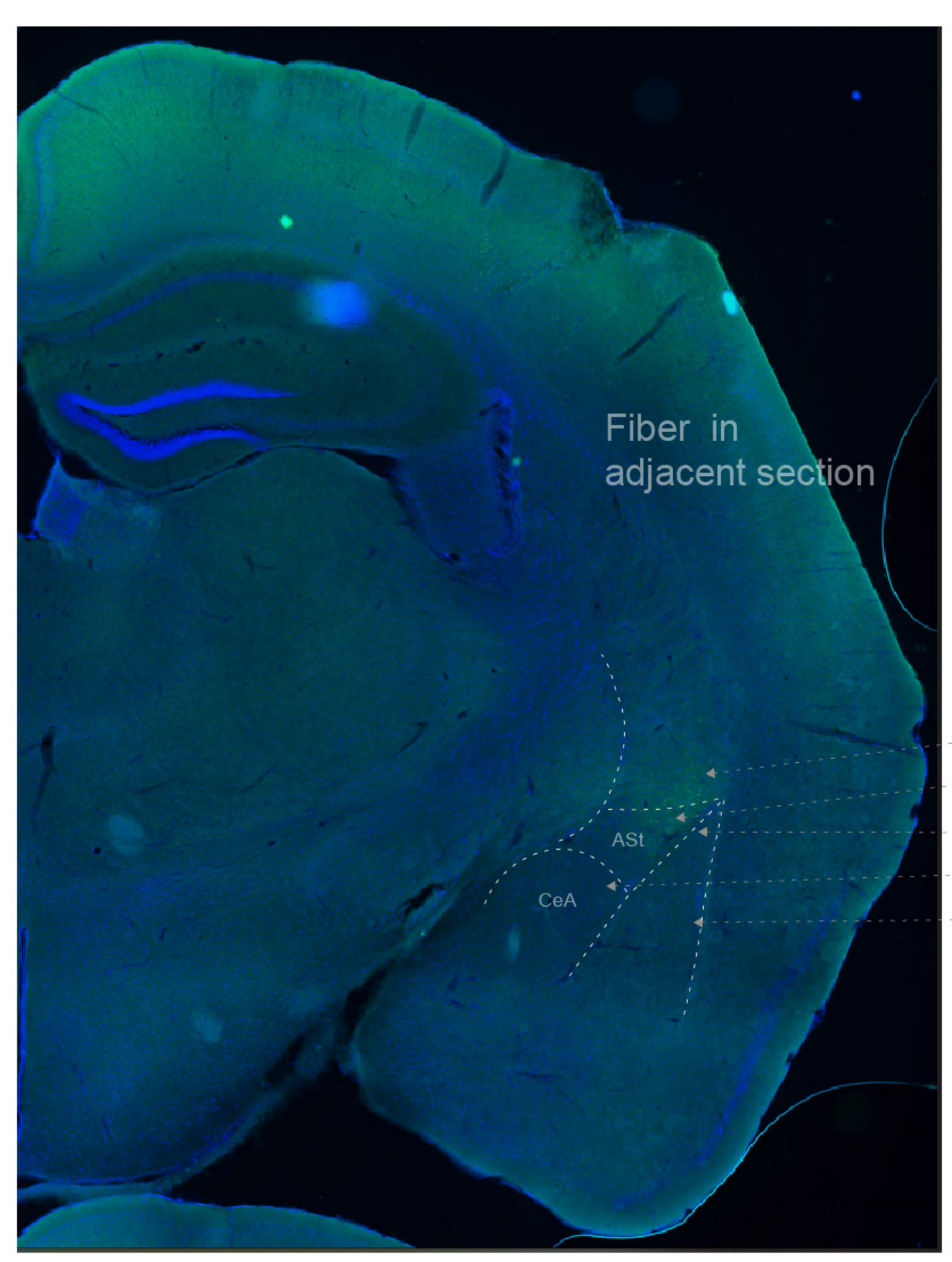

Data from this mouse were not included in the study

- TS: Almost no expression ✓
- ASt: Very low expression ✗
- LA: Low expression ✓
- CeA: No expression ✓
- BLA: No expression ✓

Appendix B - Histology - *VGAT:Cre* mice, ChR2 experiments:

VGAT - ChR2 Cohort

"Mouse ID: C701A19R"

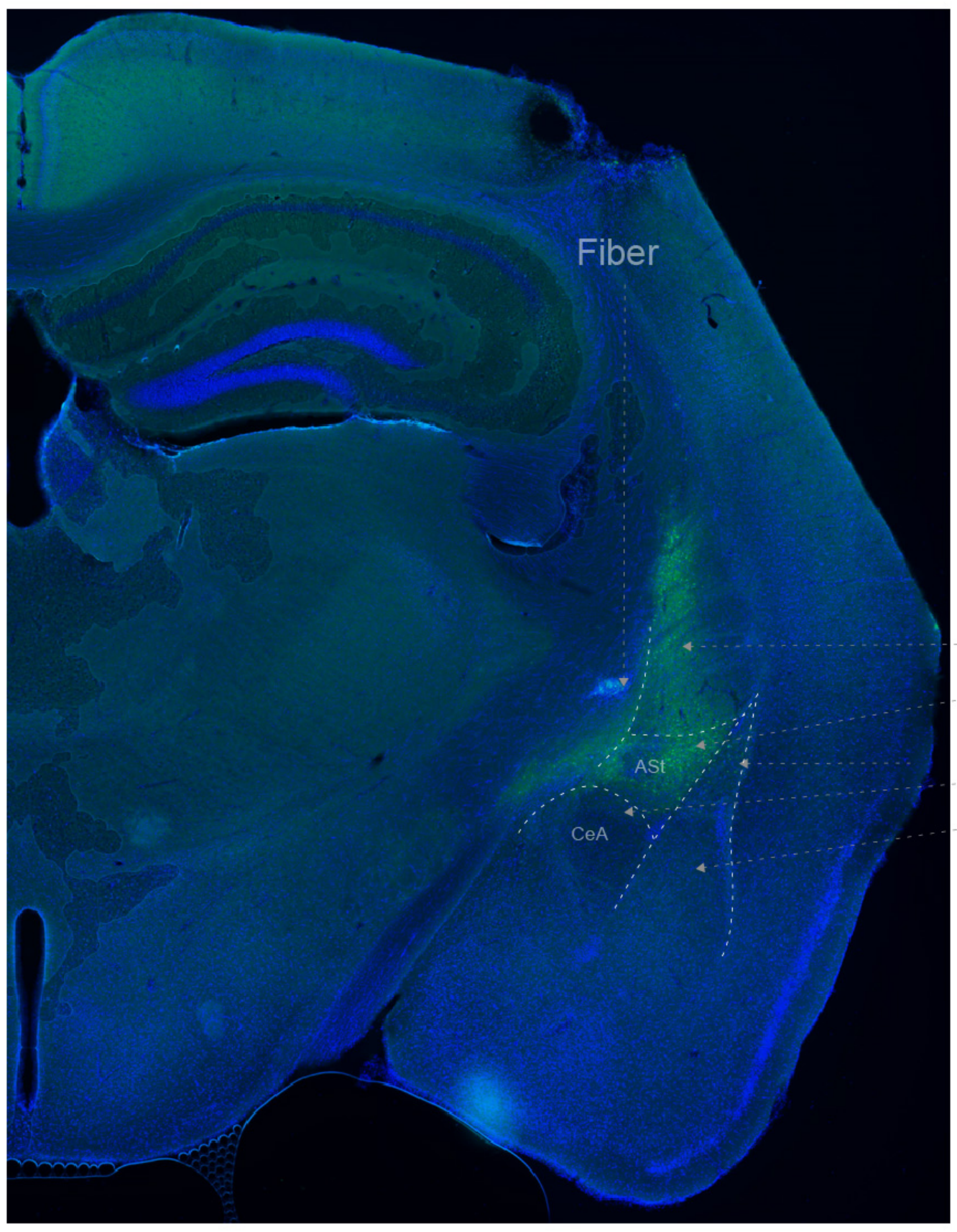

Histology okay, data included

TS: Some expression ✓
(outside of light cone from fiber)
ASt: Good expression ✓
LA: Very low expr. (GABA only) ✓
CeA: No expression ✓
BLA: No expression ✓

VGAT - ChR2 Cohort

"Mouse ID: C405A12R"

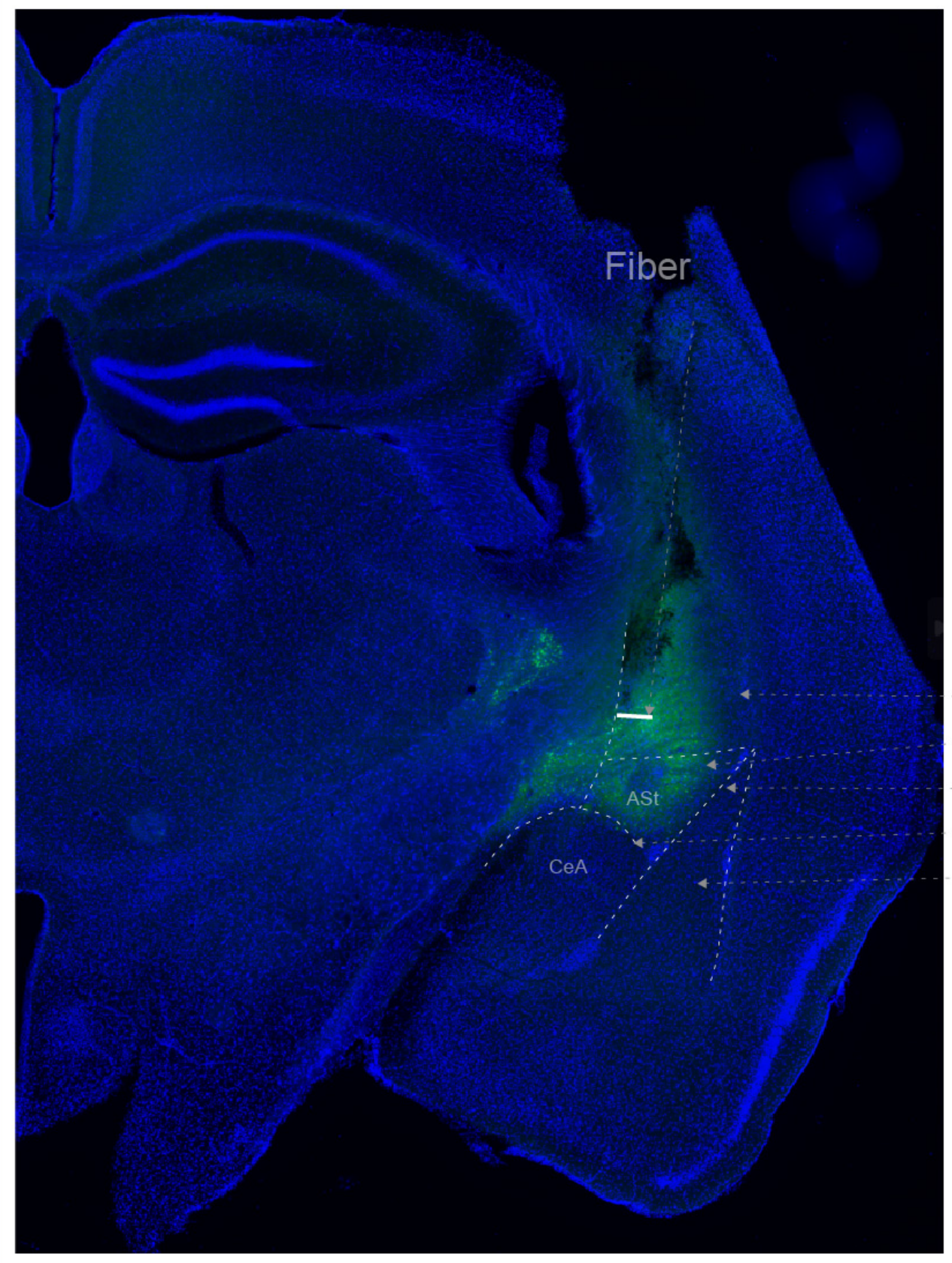

Histology okay, data included

TS: Some expression ✓
(outside of light cone from fiber)
ASt: Good expression ✓
LA: No expression ✓
CeA: No expression ✓
BLA: No expression ✓

VGAT - ChR2 Cohort

"Mouse ID: C801A09R"

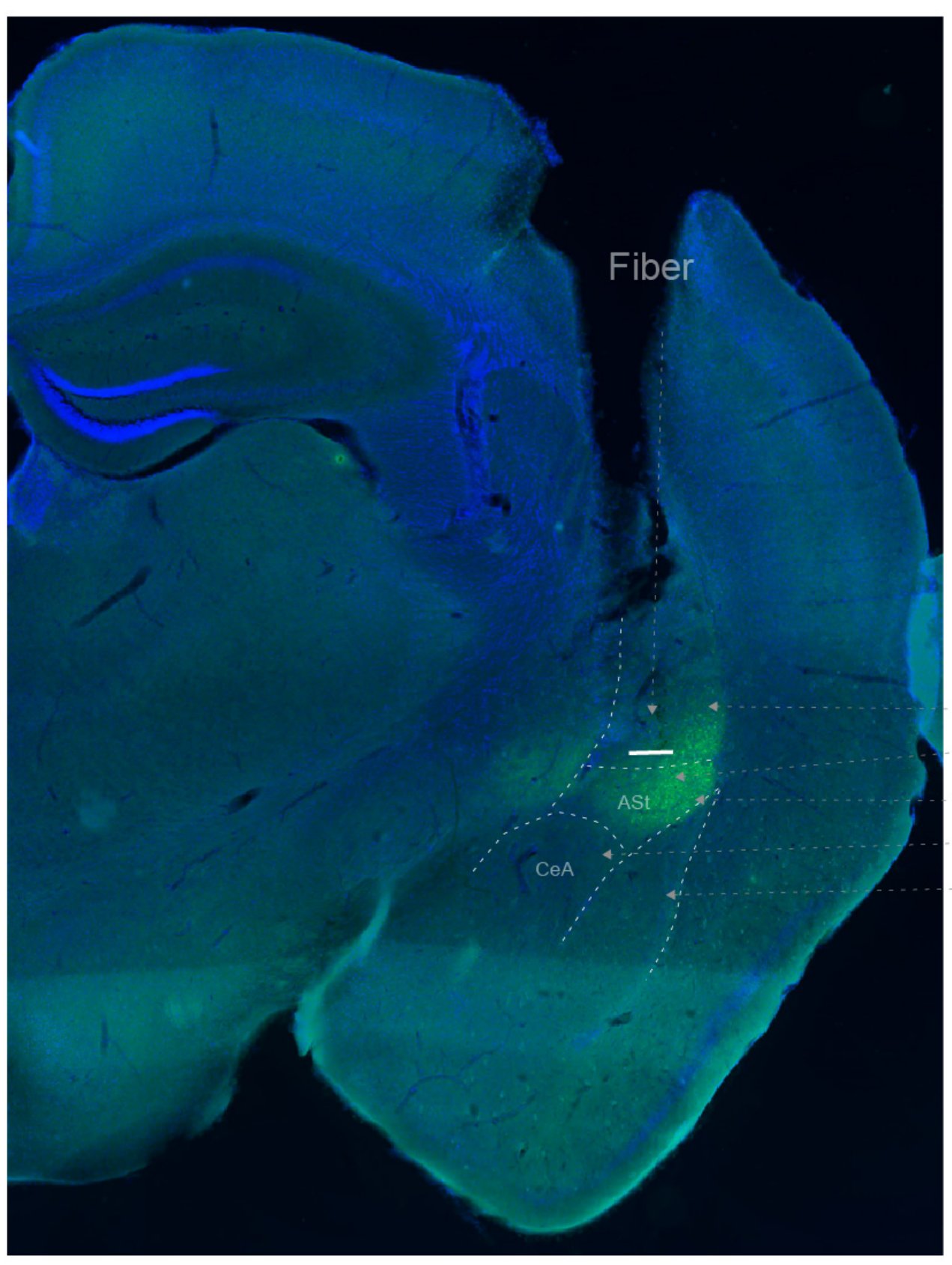

Histology okay, data included

TS: Minimal expression ✓
ASt: Good expression ✓
LA: No expression ✓
CeA: No expression ✓
BLA: No expression ✓

Note: background green color due to autofluorescence

VGAT - ChR2 Cohort

"Mouse ID: C601A06R"

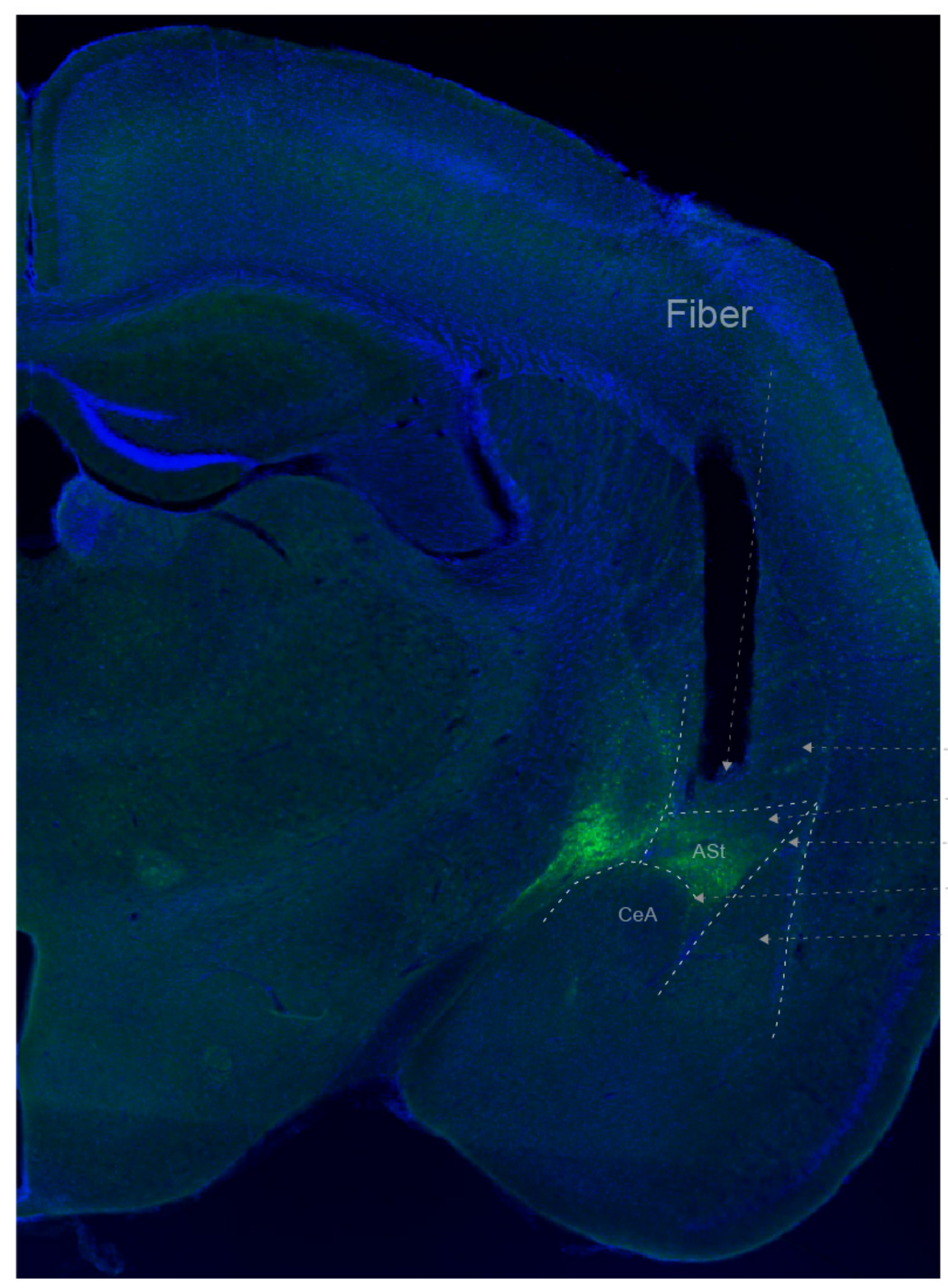

Histology okay, data included

TS: No expression ✓
ASt: Good expression ✓
LA: No expression ✓
CeA: No expression ✓
BLA: No expression ✓

VGAT - ChR2 Cohort

"Mouse ID: C501A10R"

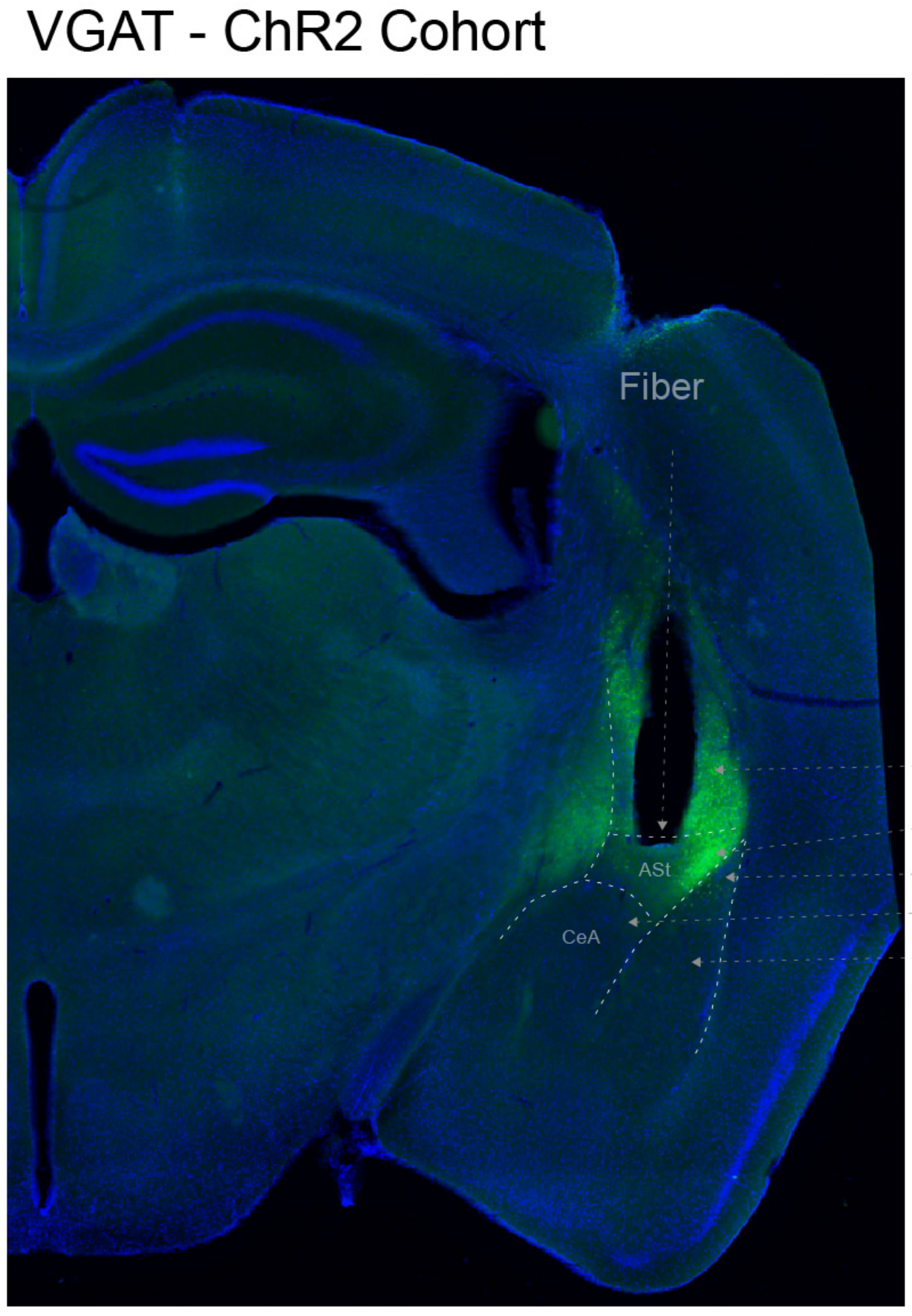

Histology okay, data included

TS: Some expression ✓
(outside of light cone from fiber)
ASt: Good expression ✓
LA: No expression ✓
CeA: No expression ✓
BLA: No expression ✓

VGAT - ChR2 Cohort

"Mouse ID: C503A13L"

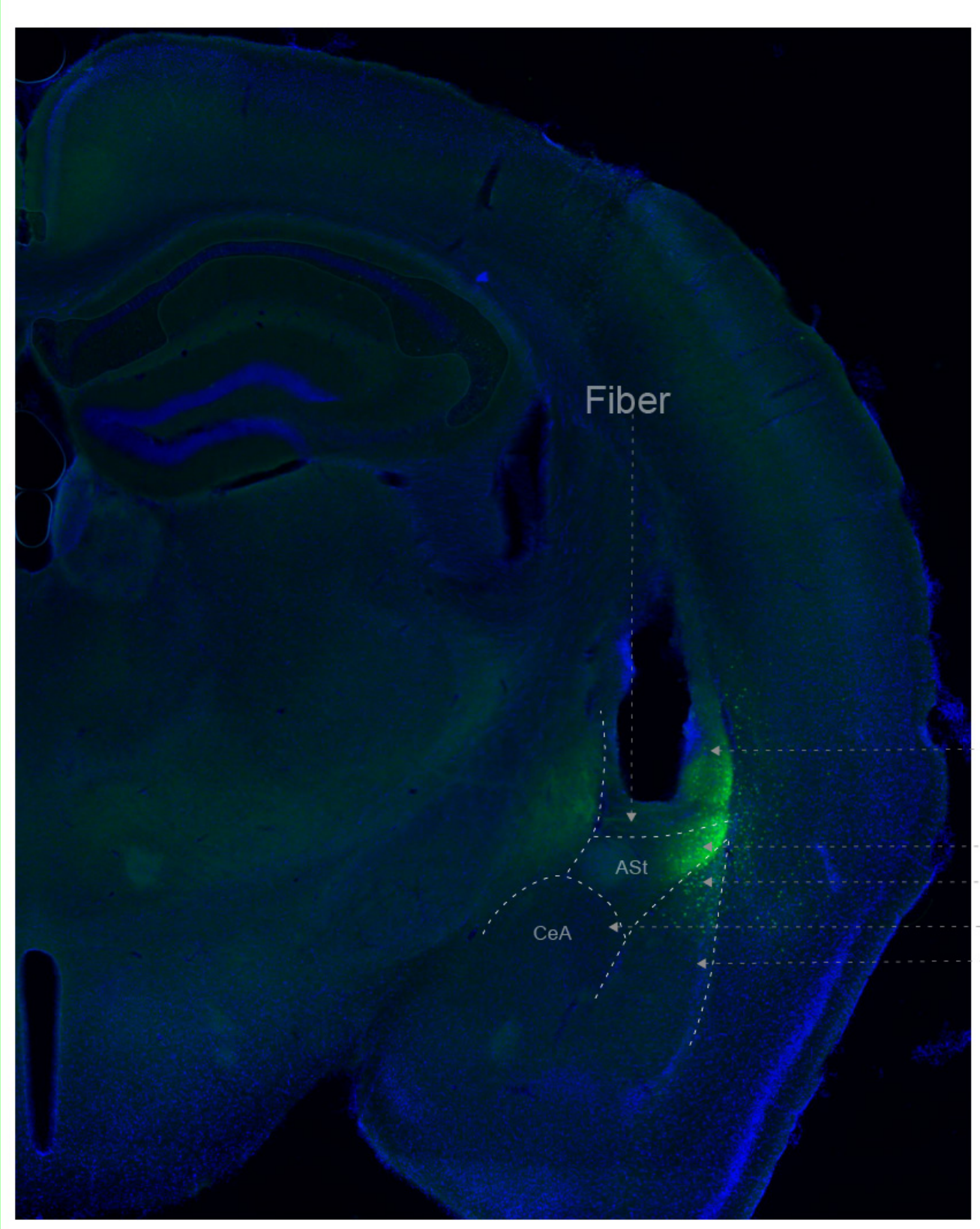

Histology okay, data included

TS: Low expression ✓

ASt: Half-expression ✓
LA: Very low expr. (GABA only) ✓
CeA: No expression ✓
BLA: No expression ✓

Appendix B - Histology - *VGAT:Cre* mice, ChR2 experiments

VGAT - ChR2 Cohort

"Mouse ID: C401A14L"

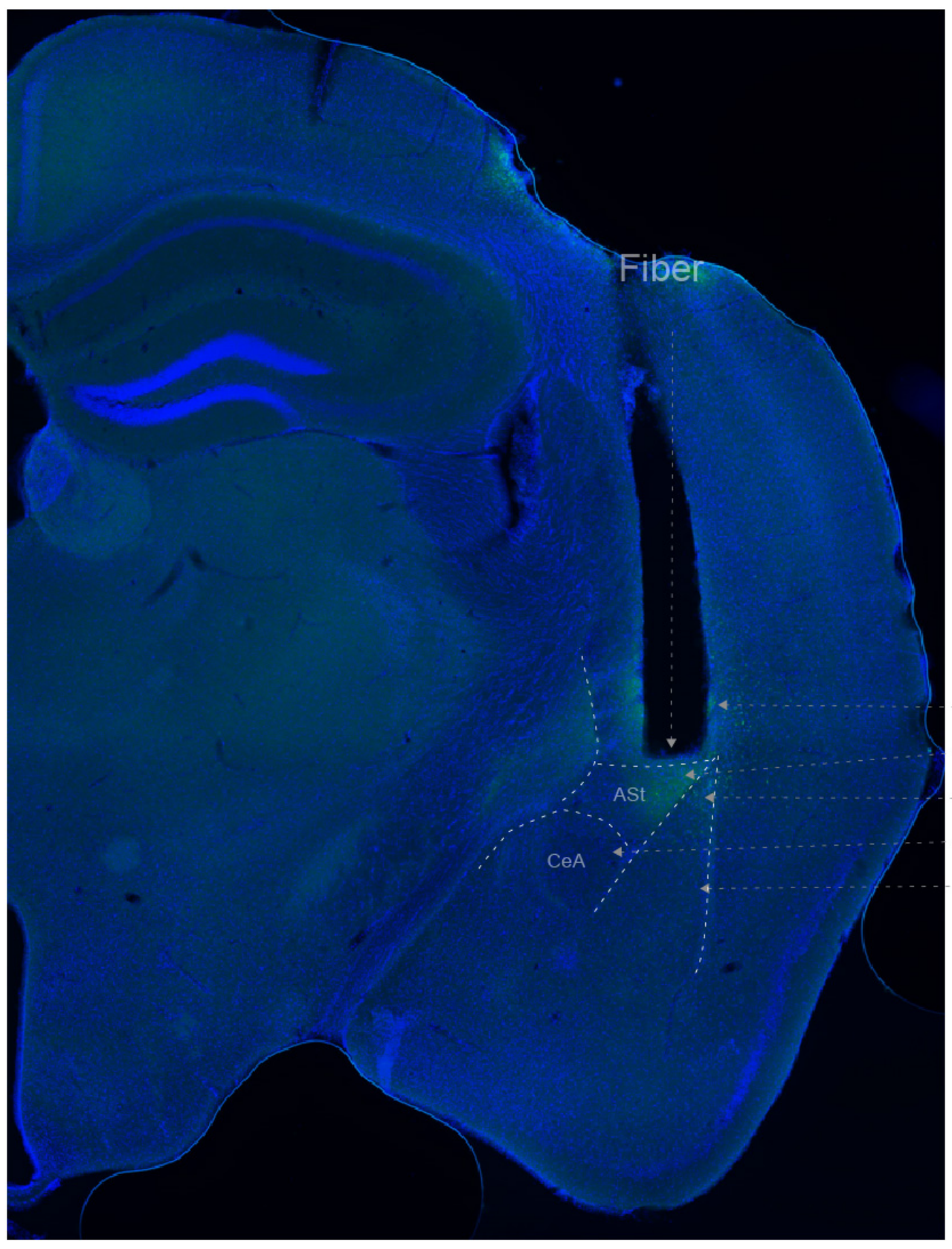

Histology okay, data included

TS: Minimal expression ✓
ASt: Okay expression ✓
LA: Very low expr. (GABA only) ✓
CeA: No expression ✓
BLA: No expression ✓

VGAT - ChR2 Cohort

"Mouse ID: C403A14L"

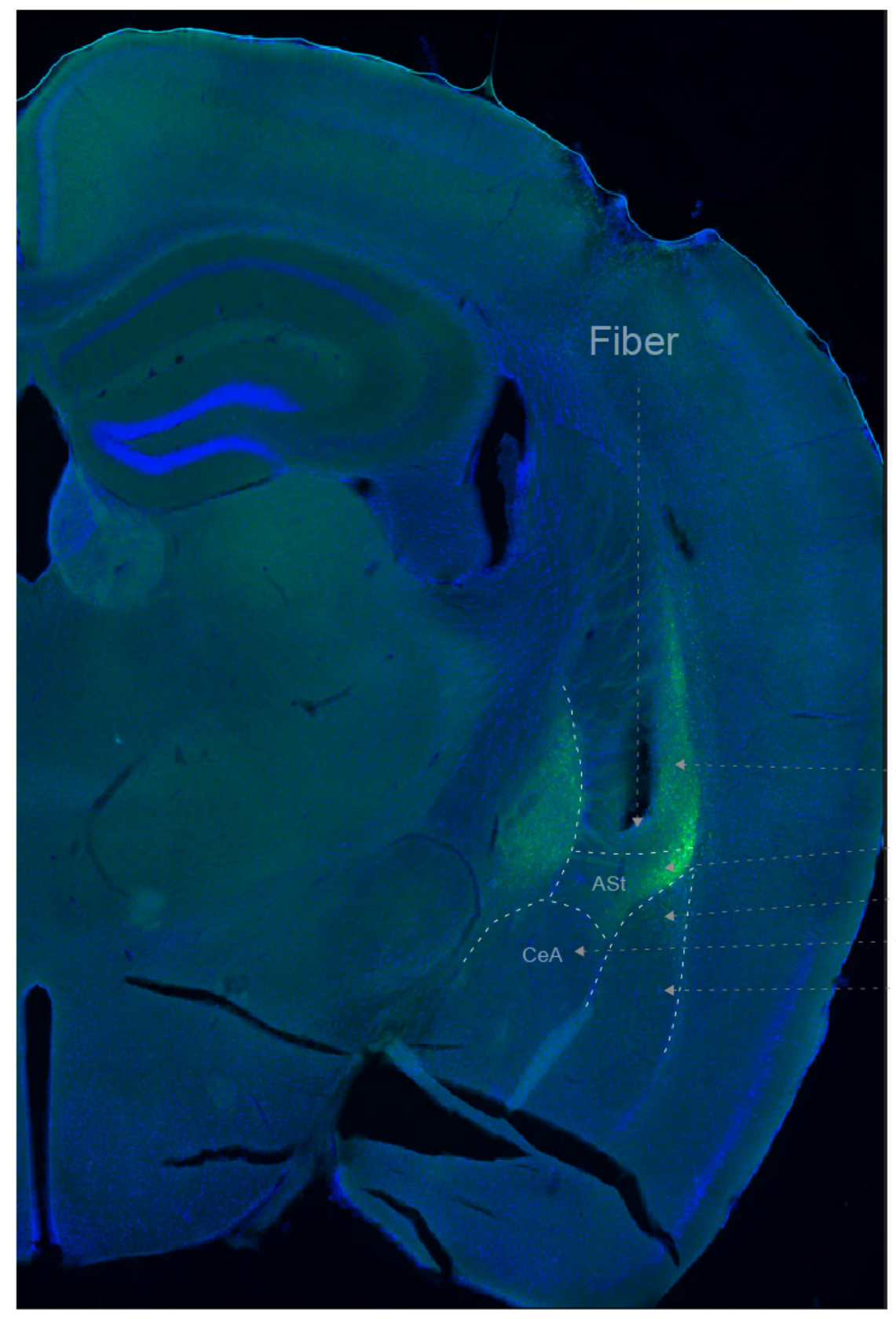

Histology okay, data included

TS: Some expression (outside of light cone from fiber) ✓
ASt: Okay expression ✓
LA: Very low expr. (GABA only) ✓
CeA: No expression ✓
BLA: No expression ✓

Mills et al. 2026

Data S1 - Appendix C
Histology for *Drd1a*-Cre and *Drd2*-Cre ChR2 mice

Appendix C - Histology - *Drd2-Cre* mice, ChR2 experiments:

Drd2+ ChR2 Cohort

"Mouse ID: D2AJ005A"

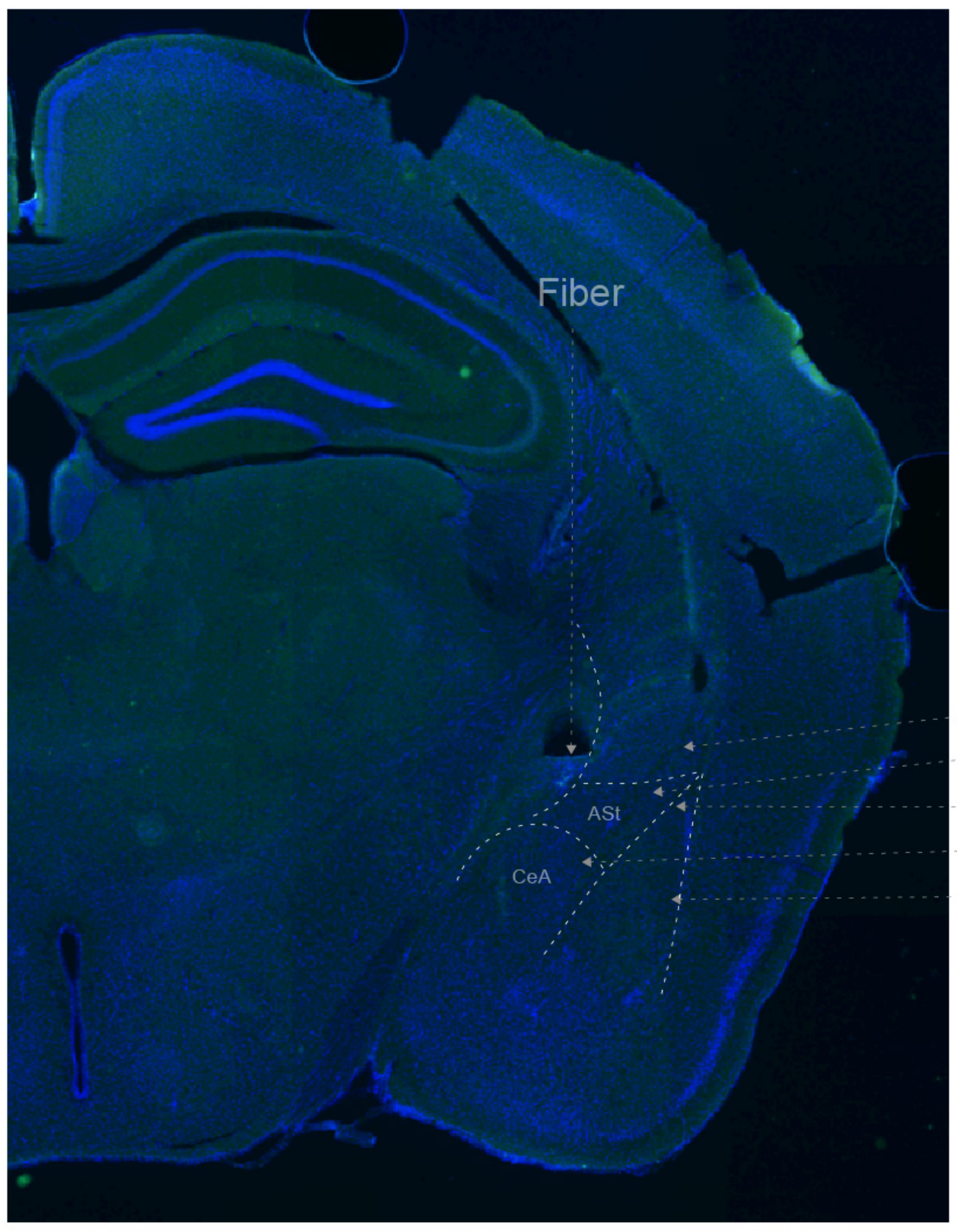

Data from this mouse were not included in the study

- TS: No expression ✓
- ASt: No expression ✗
- LA: No expression ✓
- CeA: No expression ✓
- BLA: No expression ✓

Drd2+ ChR2 Cohort

"Mouse ID: D2AJ005B"

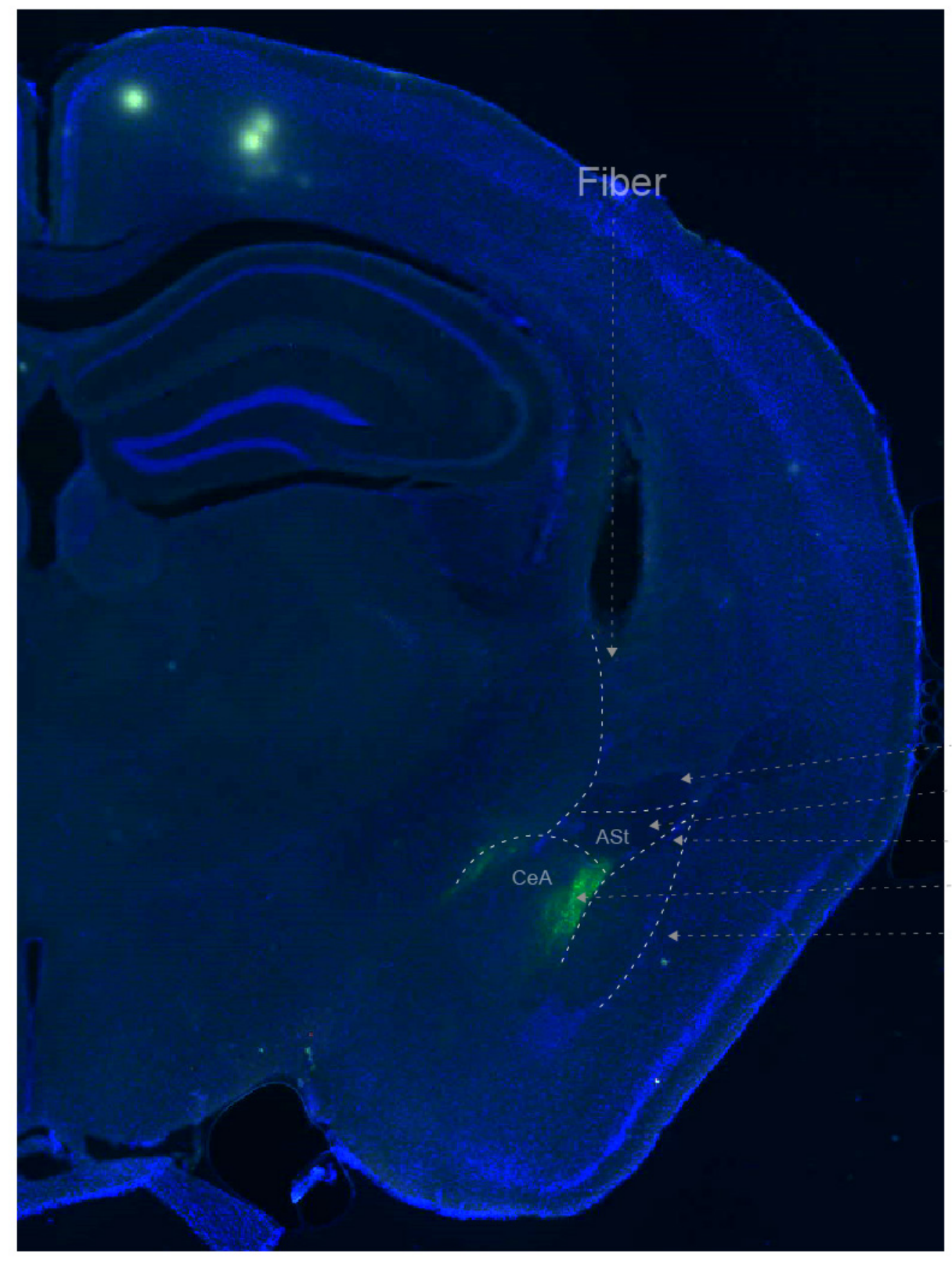

Data from this mouse were not included in the study

- TS: Almost no expression ✓
- ASt: Very low expression ✓
- LA: Low expression ✓
- CeA: Expression ✗
- BLA: No expression ✓

Drd2+ ChR2 Cohort

"Mouse ID: D2AJ033A"

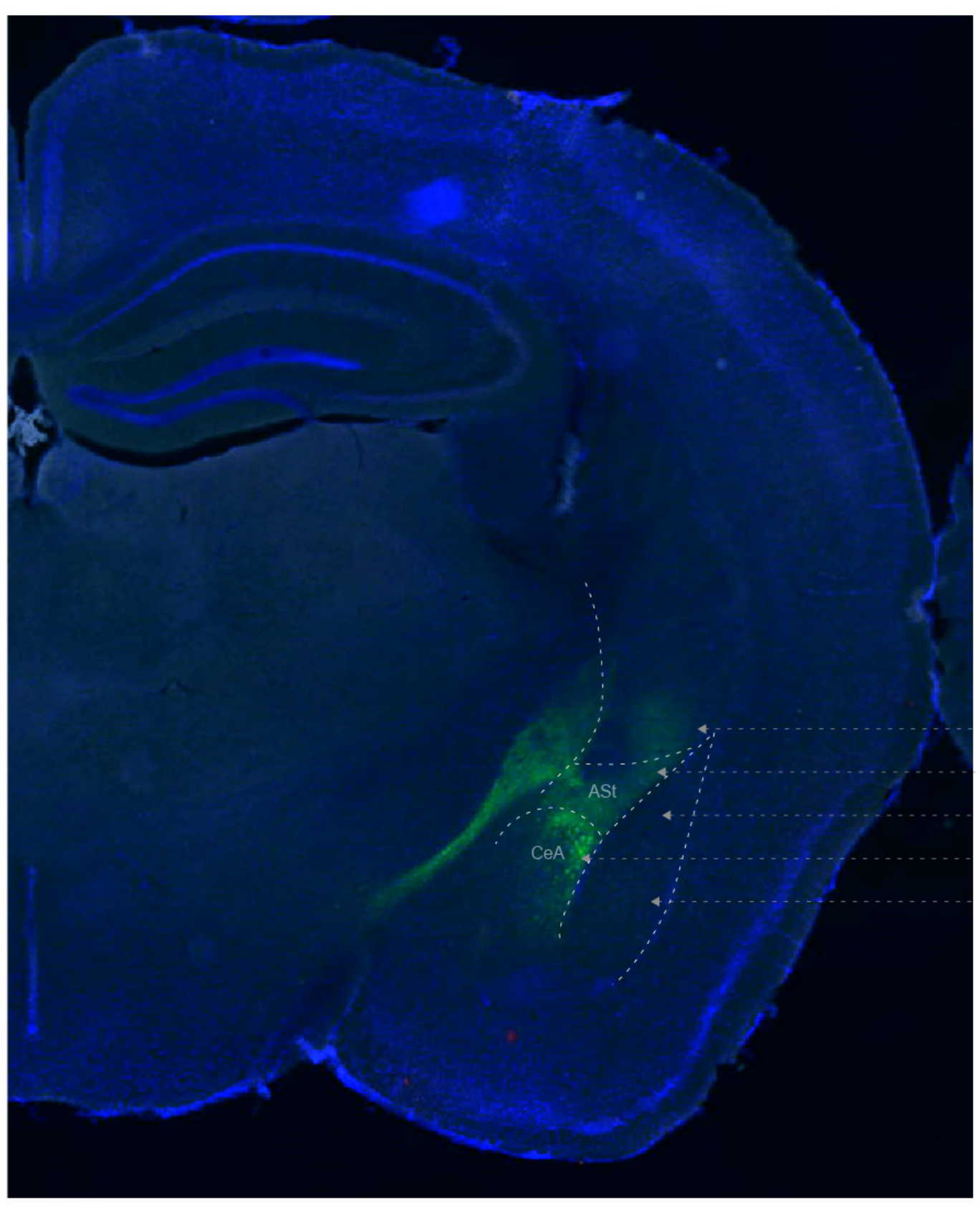

Data from this mouse were not included in the study

- TS: Low expression ✓
- ASt: Ok expression ✓
- LA: No expression ✓
- CeA: Expression ✗
- BLA: No expression ✓

Drd2+ ChR2 Cohort

"Mouse ID: D2AJ046A"

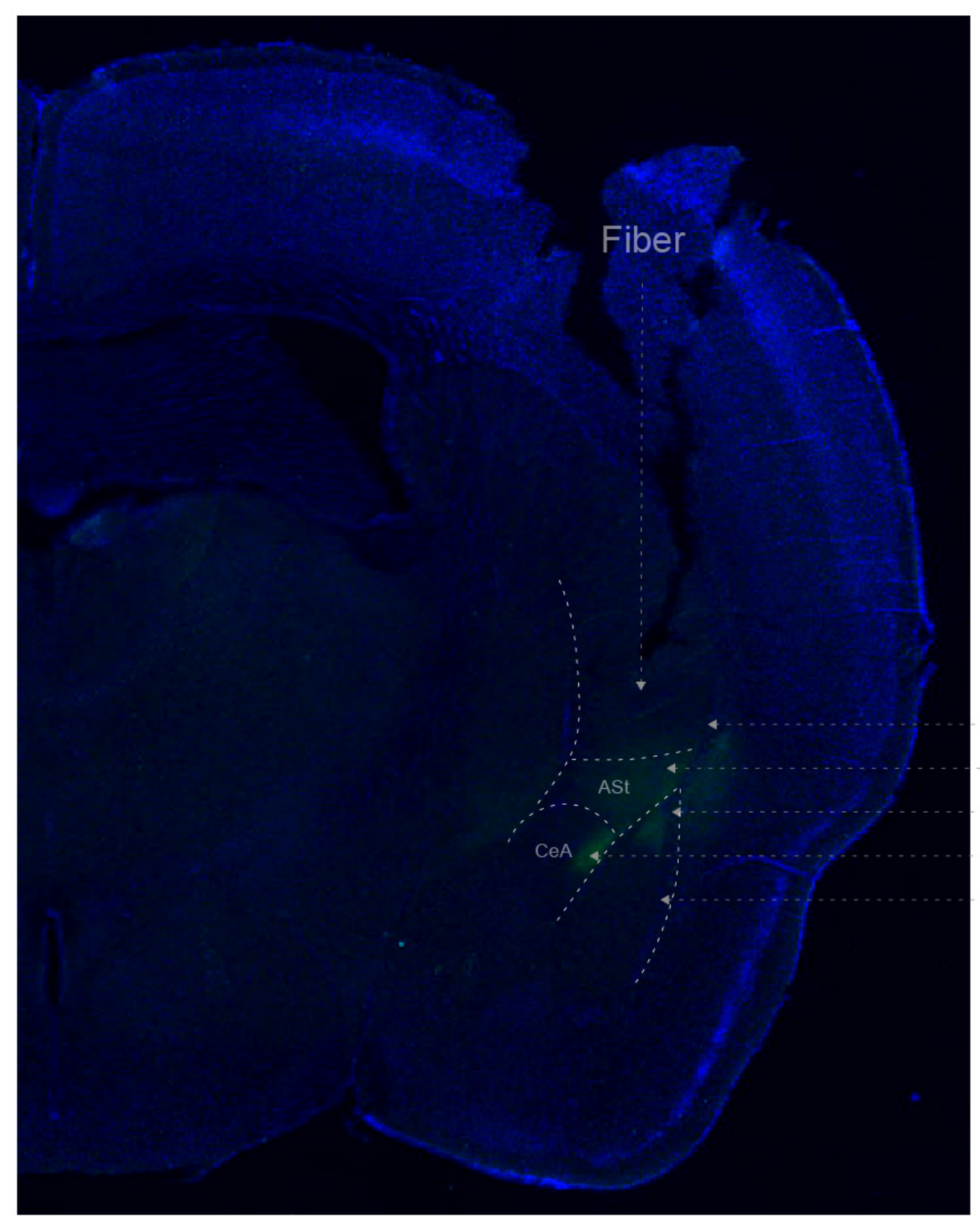

Data from this mouse were not included in the study

- TS: No expression ✓
- ASt: Ok expression ✓
- LA: Some expression ✓
- CeA: Expression ✗
- BLA: No expression ✓

Drd2+ ChR2 Cohort

"Mouse ID: D2AJ010A"

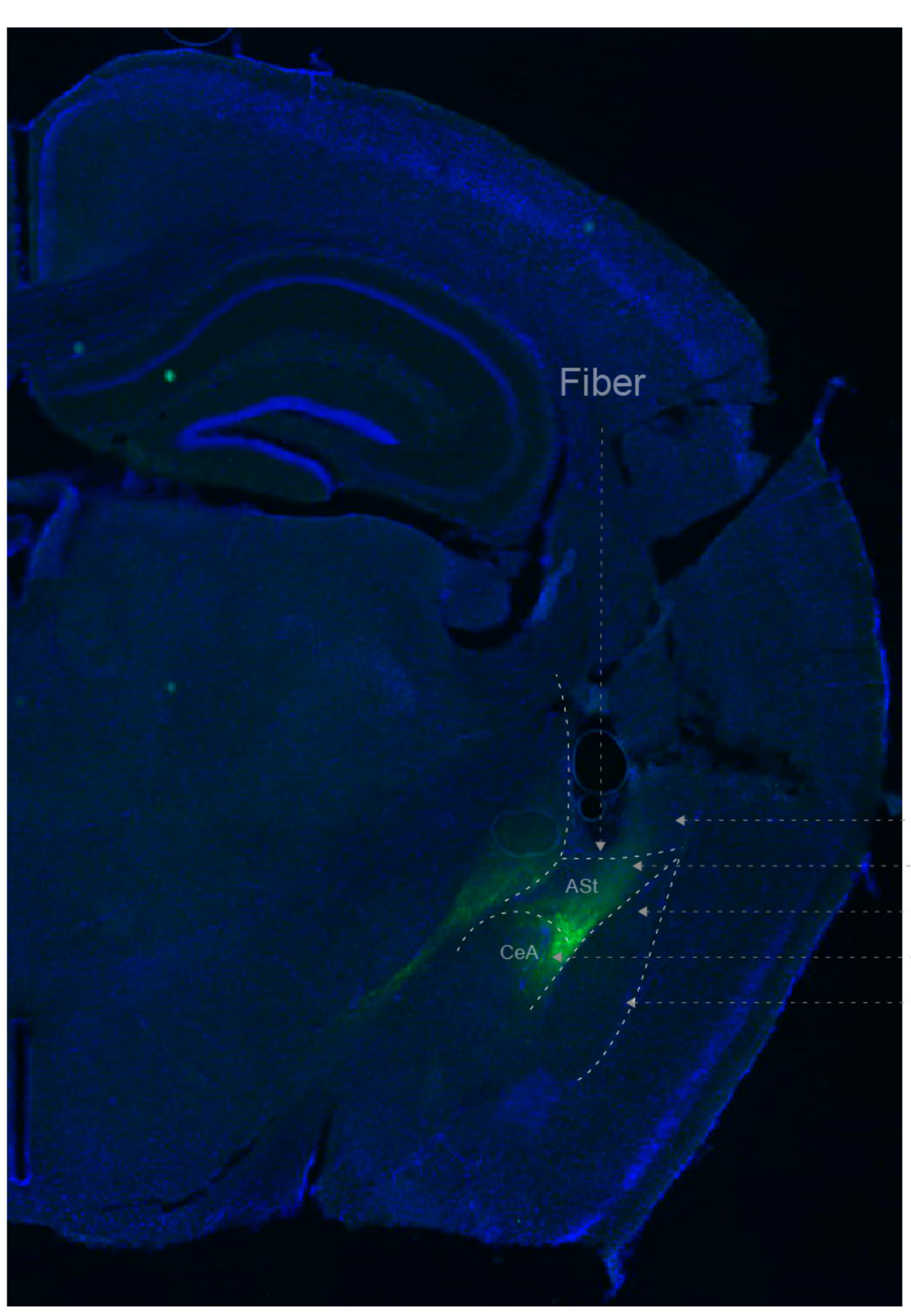

Data from this mouse were not included in the study

- TS: Slight expression ✓
- ASt: Good expression ✓
- LA: No expression ✓
- CeA: Expression ✗
- BLA: No expression ✓

Drd2+ ChR2 Cohort

"Mouse ID: D2AJ010B"

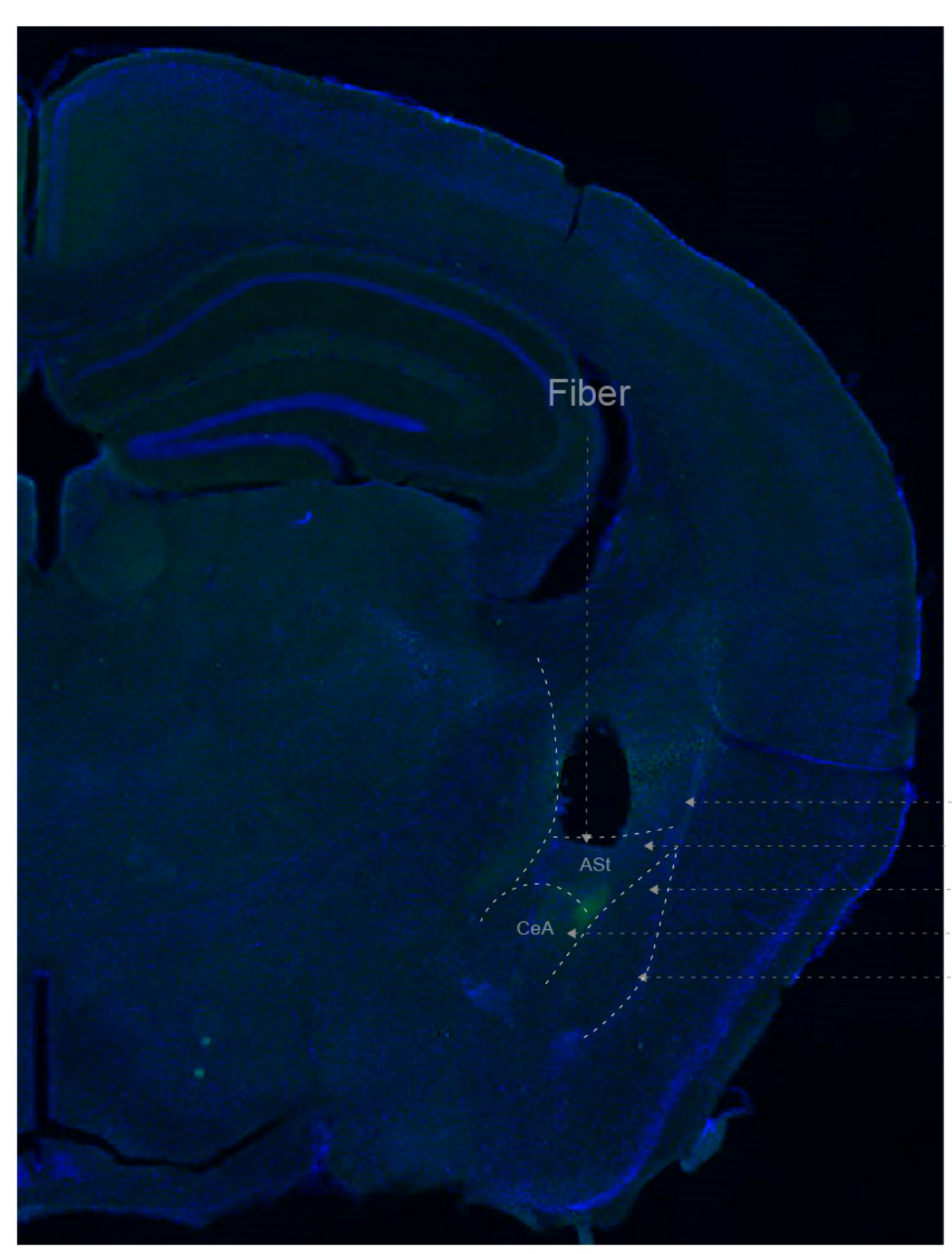

Data from this mouse were not included in the study

- TS: Slight expression ✓
- ASt: Ok expression ✓
- LA: No expression ✓
- CeA: Expression ✗
- BLA: No expression ✓

Appendix C - Histology - *Drd2-Cre* mice, ChR2 experiments:

Drd2+ ChR2 Cohort

"Mouse ID: D2AJ017A"

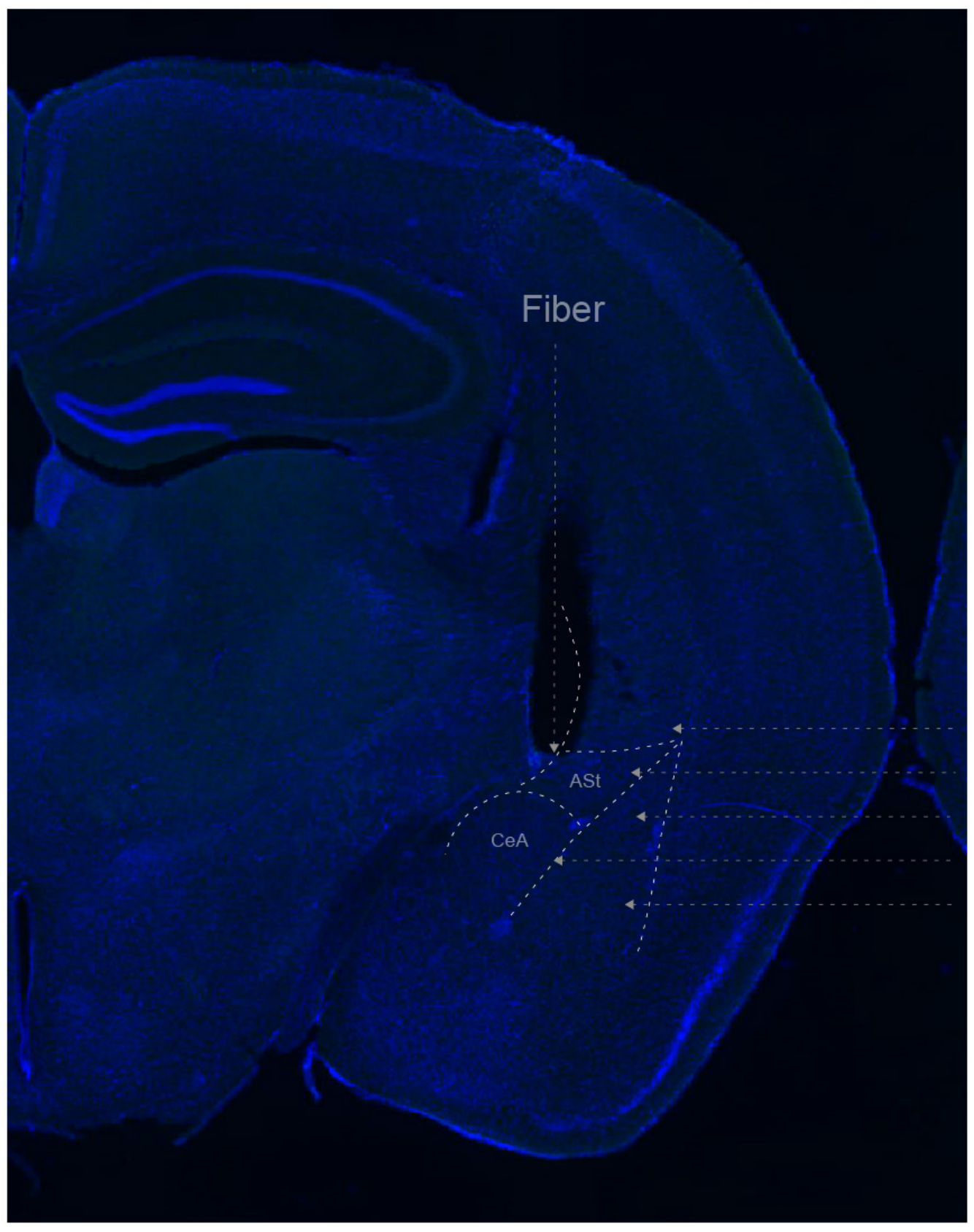

Data from this mouse were not included in the study

TS: No expression ✓
ASt: No expression ✗
LA: No expression ✓
CeA: No expression ✓
BLA: No expression ✓

Drd2+ ChR2 Cohort

"Mouse ID: D2AJ017B"

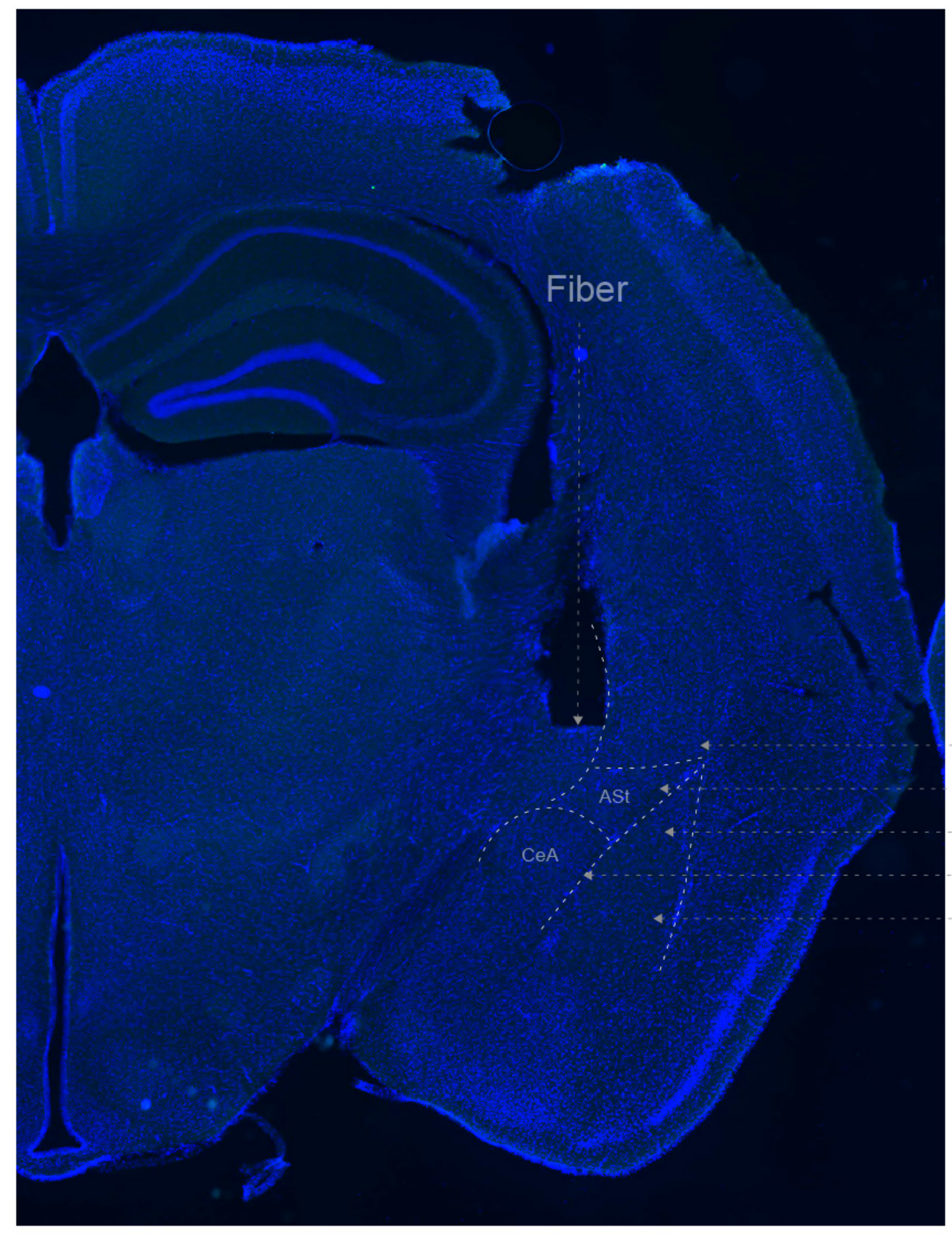

Data from this mouse were not included in the study

TS: No expression ✓
ASt: No expression ✗
LA: No expression ✓
CeA: No expression ✓
BLA: No expression ✓

Drd2+ ChR2 Cohort

"Mouse ID: D2AJ021A"

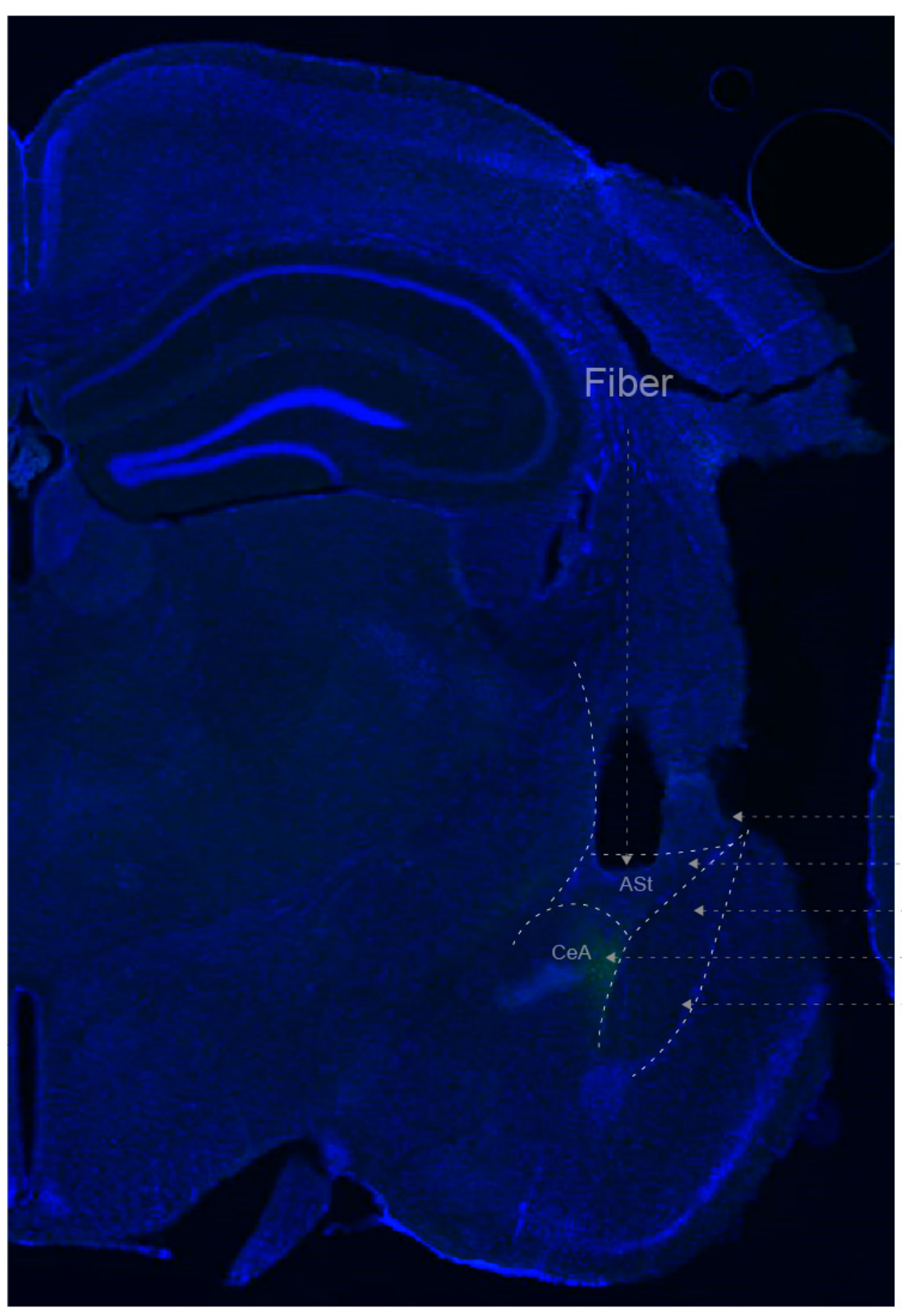

Data from this mouse were not included in the study

TS: No expression ✓
ASt: No expression ✓
LA: No expression ✓
CeA: Expression ✗
BLA: No expression ✓

Drd2+ ChR2 Cohort

"Mouse ID: D2AJ021B"

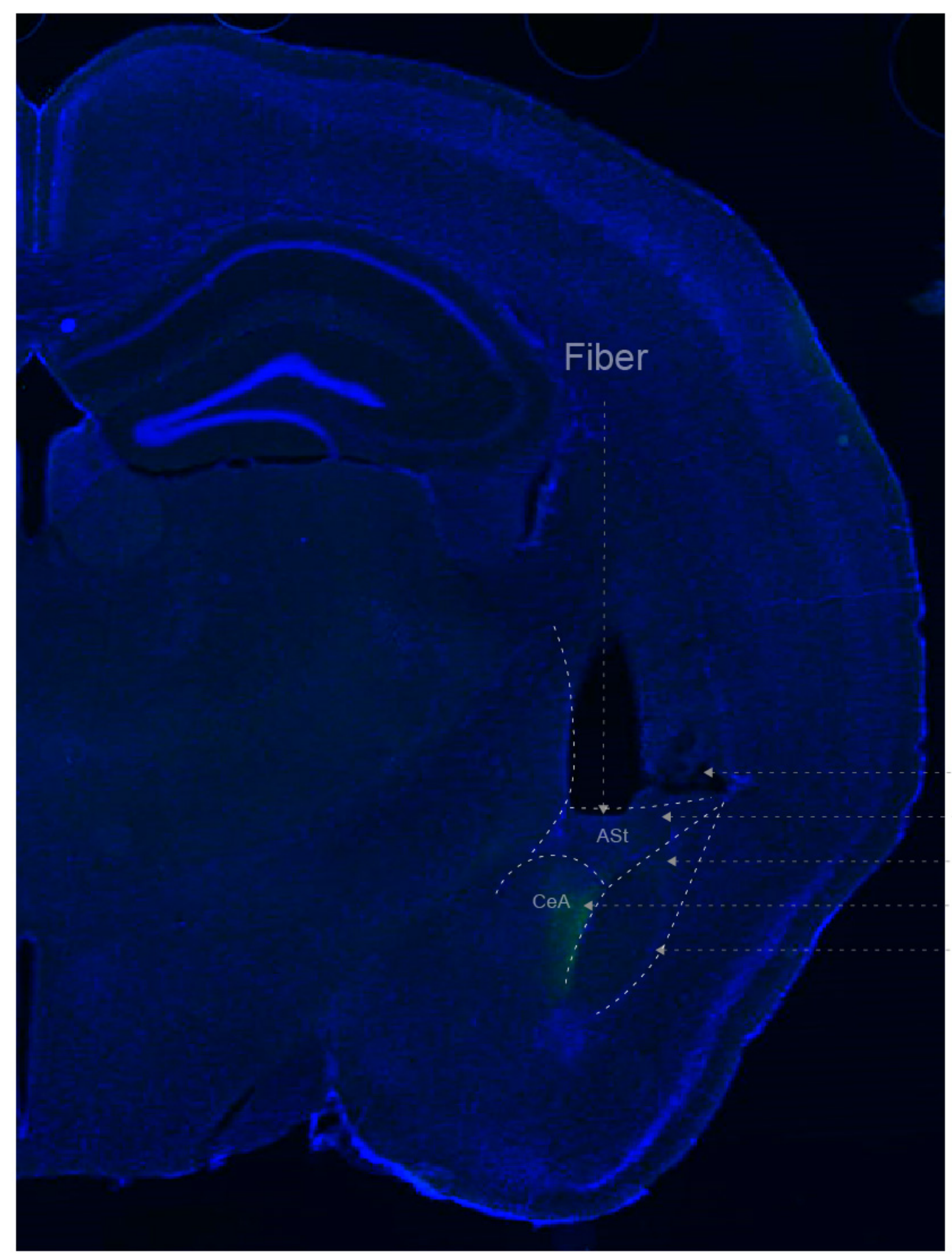

Data from this mouse were not included in the study

TS: No expression ✓
ASt: No expression ✓
LA: No expression ✓
CeA: Expression ✗
BLA: No expression ✓

Drd2+ ChR2 Cohort

"Mouse ID: D2AJ064B"

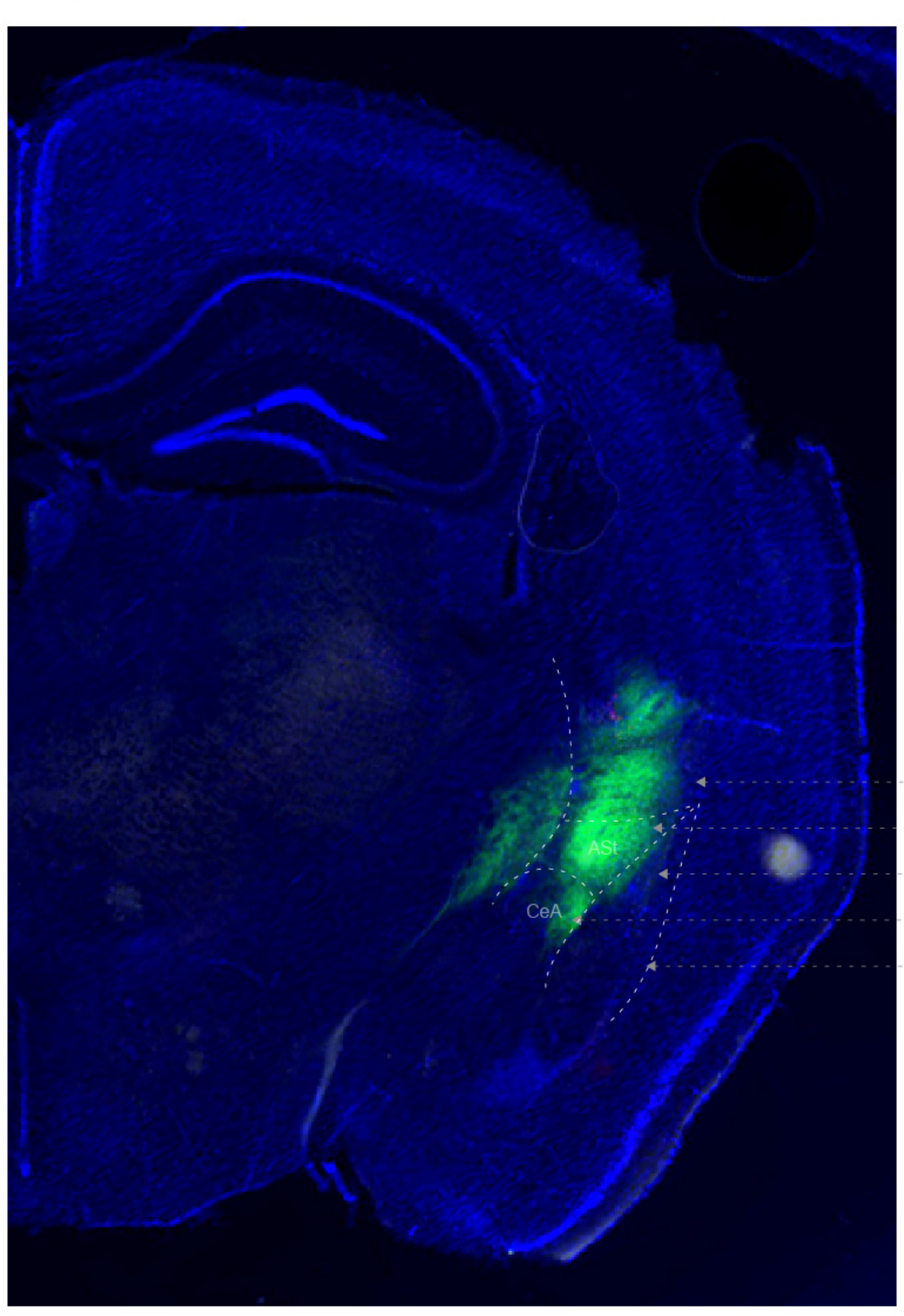

Data from this mouse were not included in the study

TS: Expression ✓
ASt: Some expression ✓
LA: No expression ✓
CeA: Expression ✗
BLA: No expression ✓

Drd2+ ChR2 Cohort

"Mouse ID: D2AJ051A"

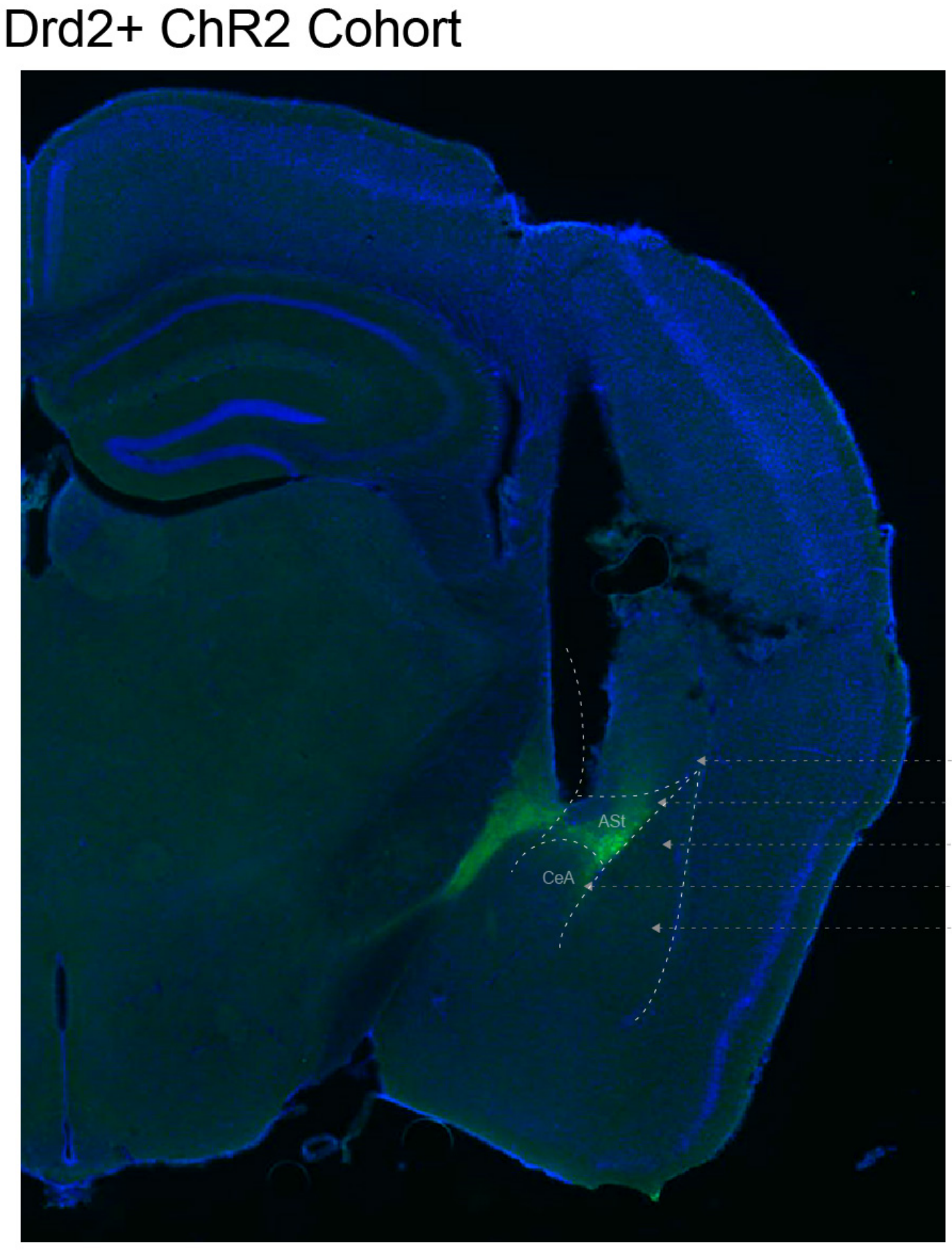

Data from this mouse were not included in the study

TS: Expression ✓
ASt: Some expression ✓
LA: No expression ✓
CeA: Expression ✗
BLA: No expression ✓

Appendix C - Histology - *Drd2-Cre* mice, ChR2 experiments:

Drd2+ ChR2 Cohort

"Mouse ID: D2BJ060L"

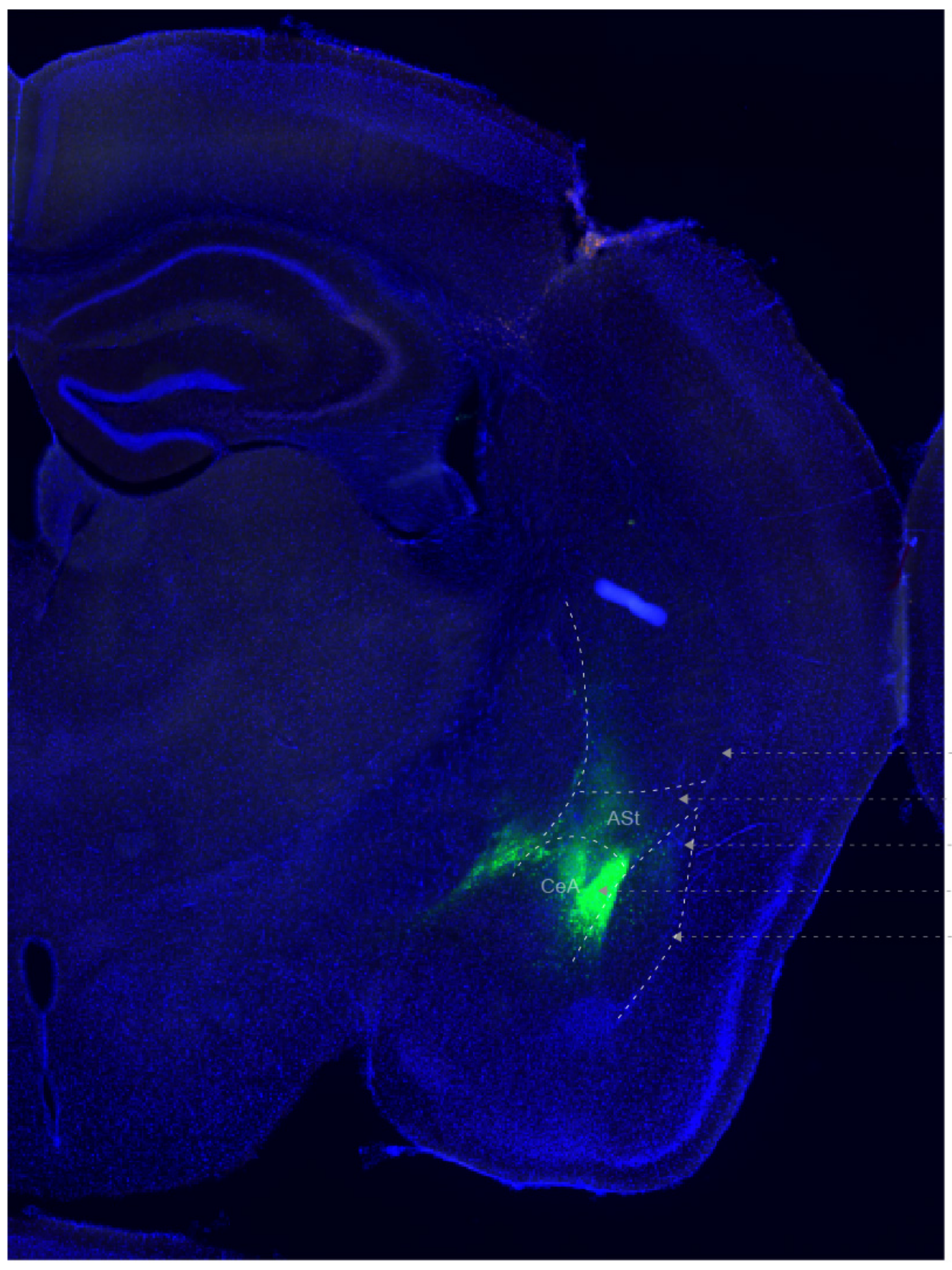

Data from this mouse were not included in the study

- TS: Low expression ✓
- ASt: Ok expression ✓
- LA: Low expression ✓
- CeA: Expression ✗
- BLA: Some expression ✓

Drd2+ ChR2 Cohort

"Mouse ID: D2BJ061R"

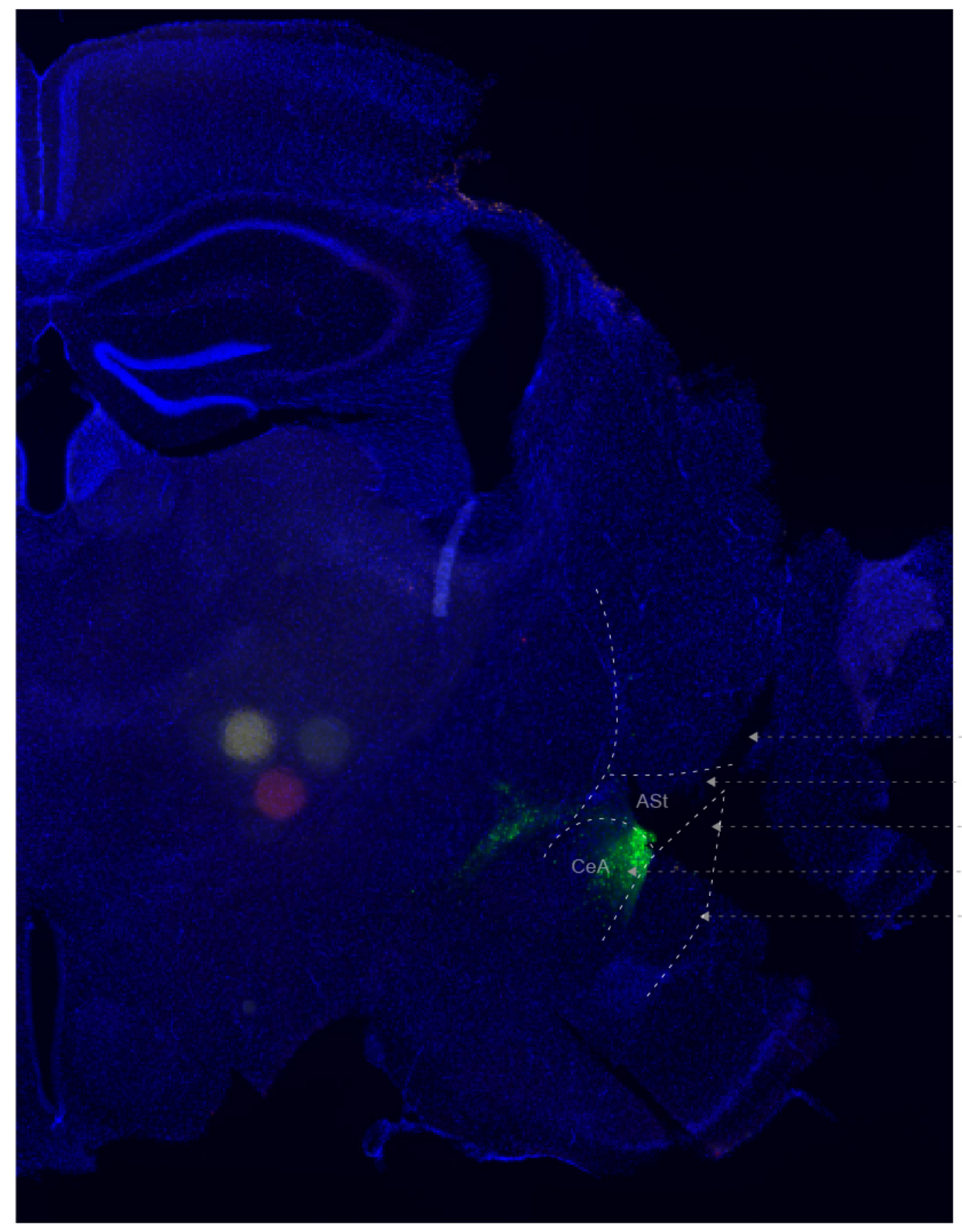

Data from this mouse were not included in the study

- TS: Low expression ✓
- ASt: Ok expression ✓
- LA: Low expression ✓
- CeA: Expression ✗
- BLA: Some expression ✓

Drd2+ ChR2 Cohort

"Mouse ID: D2BJ082R"

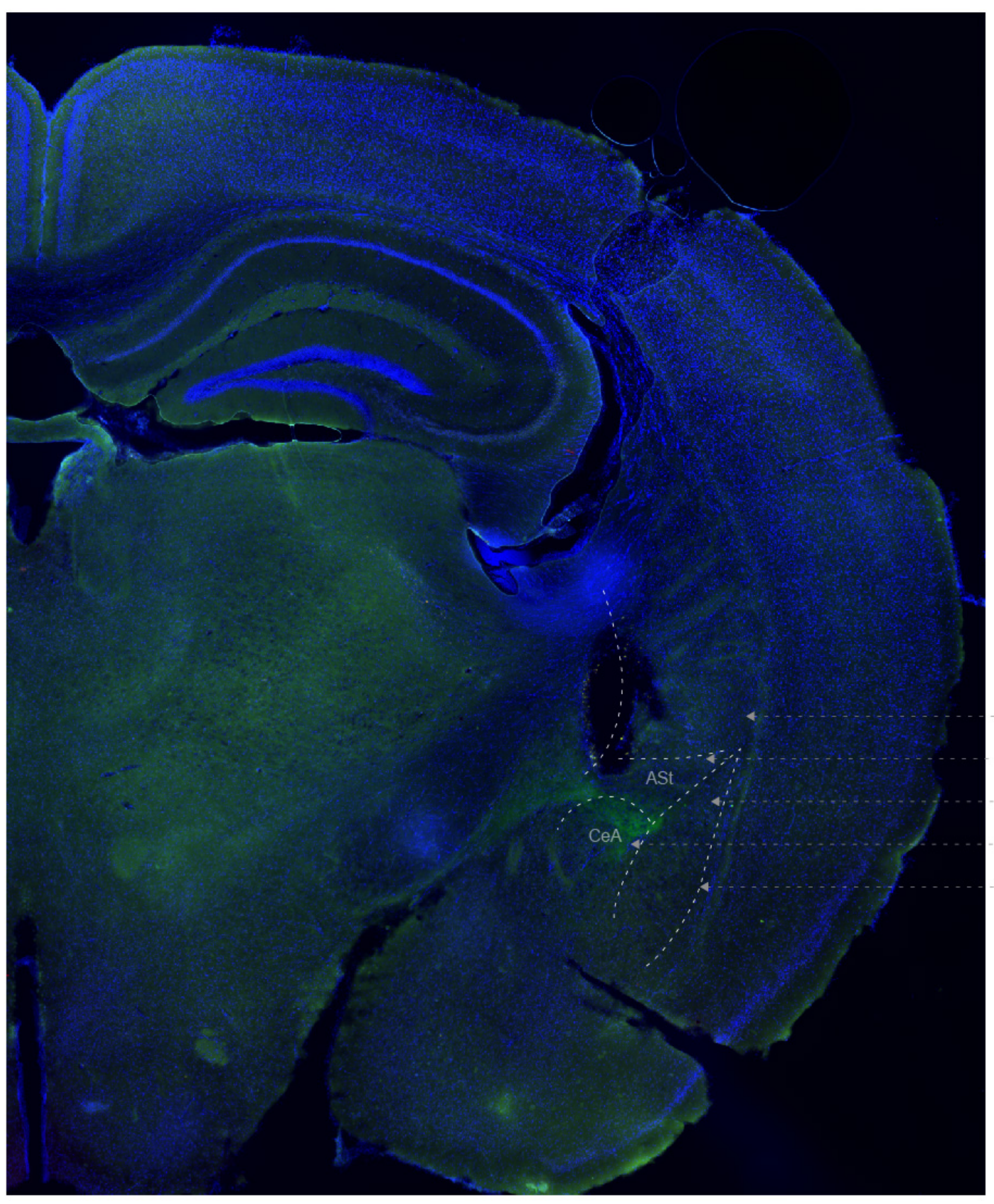

Data from this mouse were not included in the study

- TS: Expression ✓
- ASt: Some expression ✓
- LA: No expression ✓
- CeA: Expression ✗
- BLA: No expression ✓

Drd2+ ChR2 Cohort

"Mouse ID: D2BJ082L"

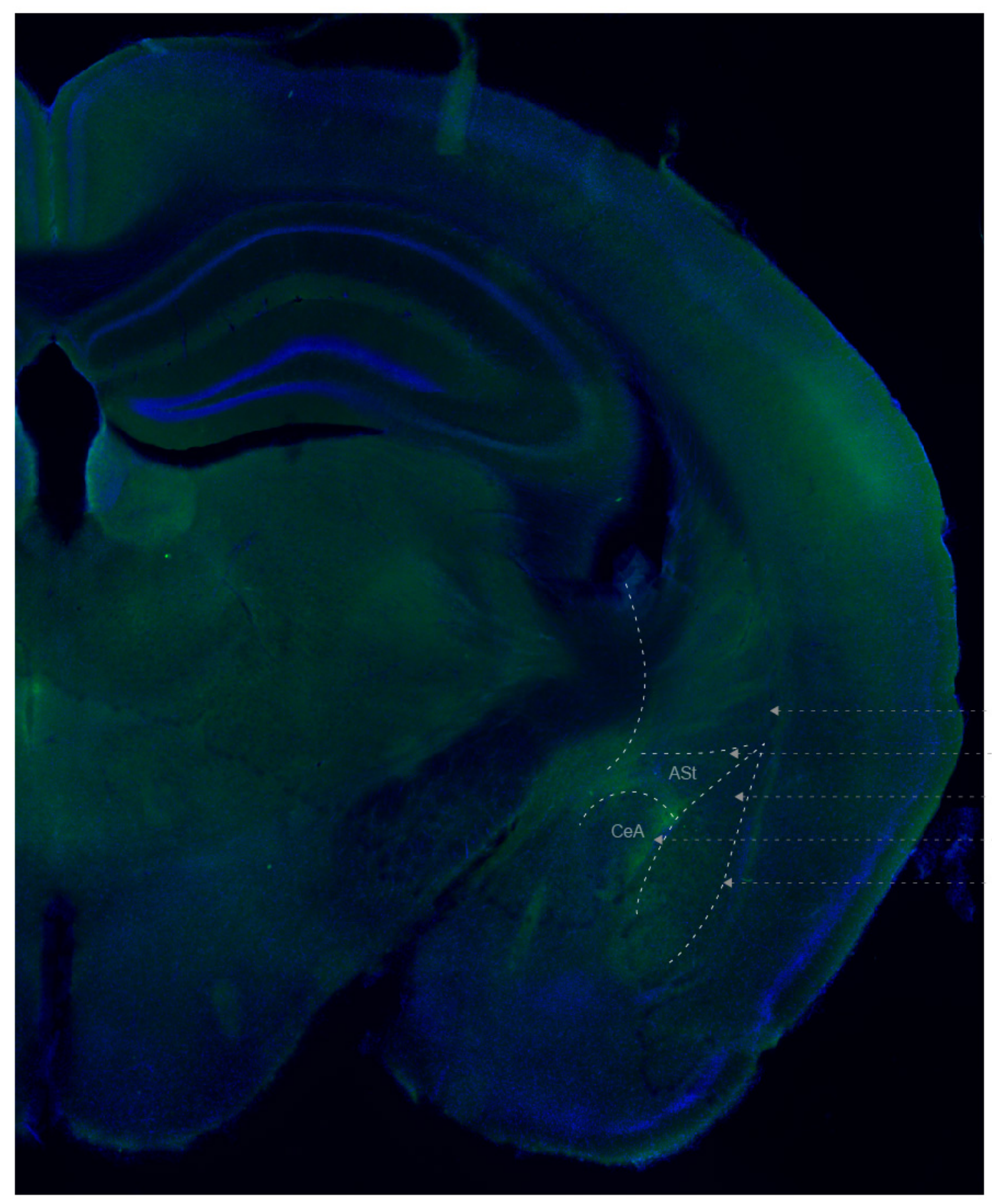

Data from this mouse were not included in the study

- TS: Expression ✓
- ASt: Some expression ✓
- LA: No expression ✓
- CeA: Expression ✗
- BLA: No expression ✓

Drd2+ ChR2 Cohort

"Mouse ID: D2BJ095R"

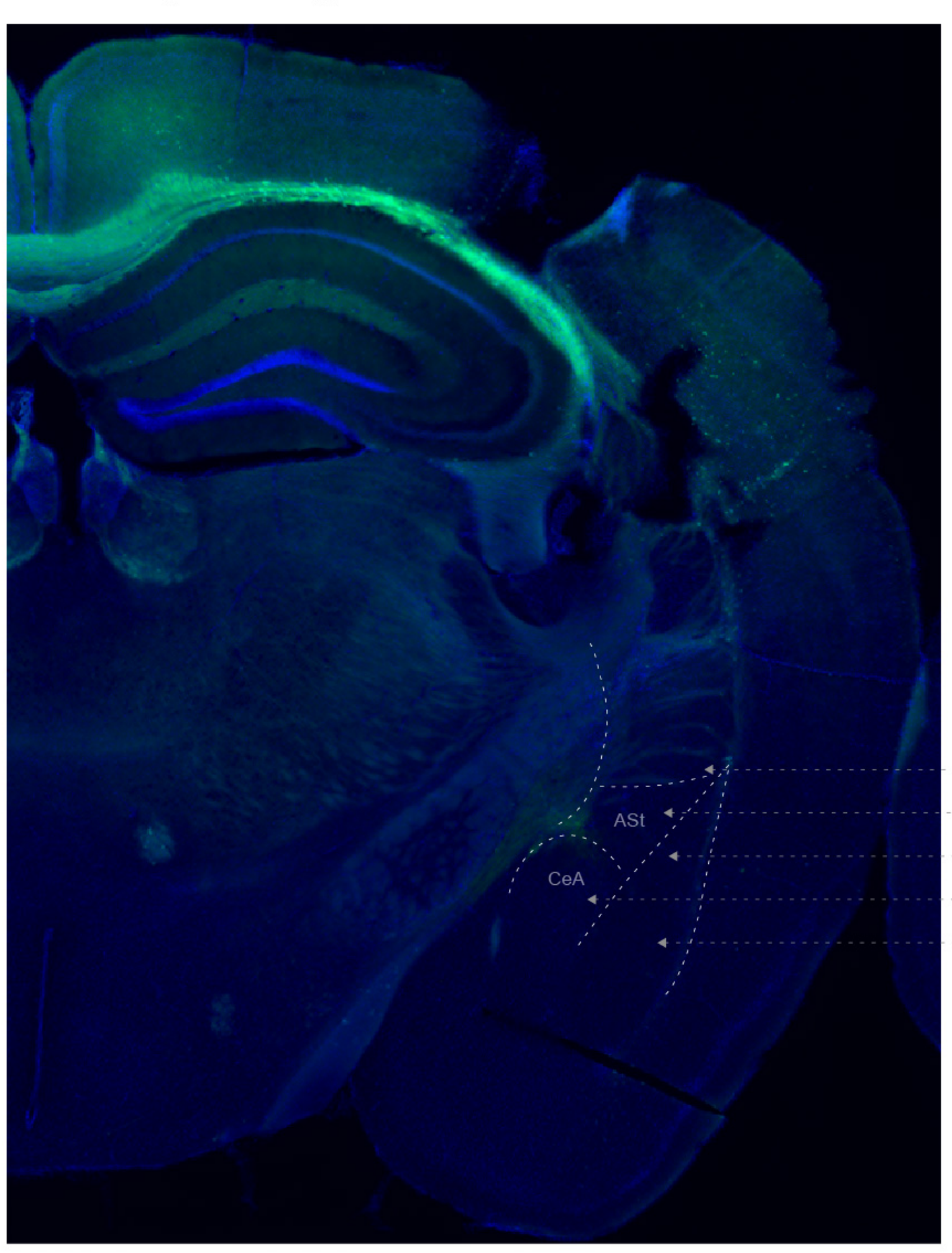

Data from this mouse were not included in the study

- TS: No expression ✓
- ASt: No expression ✗
- LA: No expression ✓
- CeA: No expression ✓
- BLA: No expression ✓

Drd2+ ChR2 Cohort

"Mouse ID: D2BJ126L"

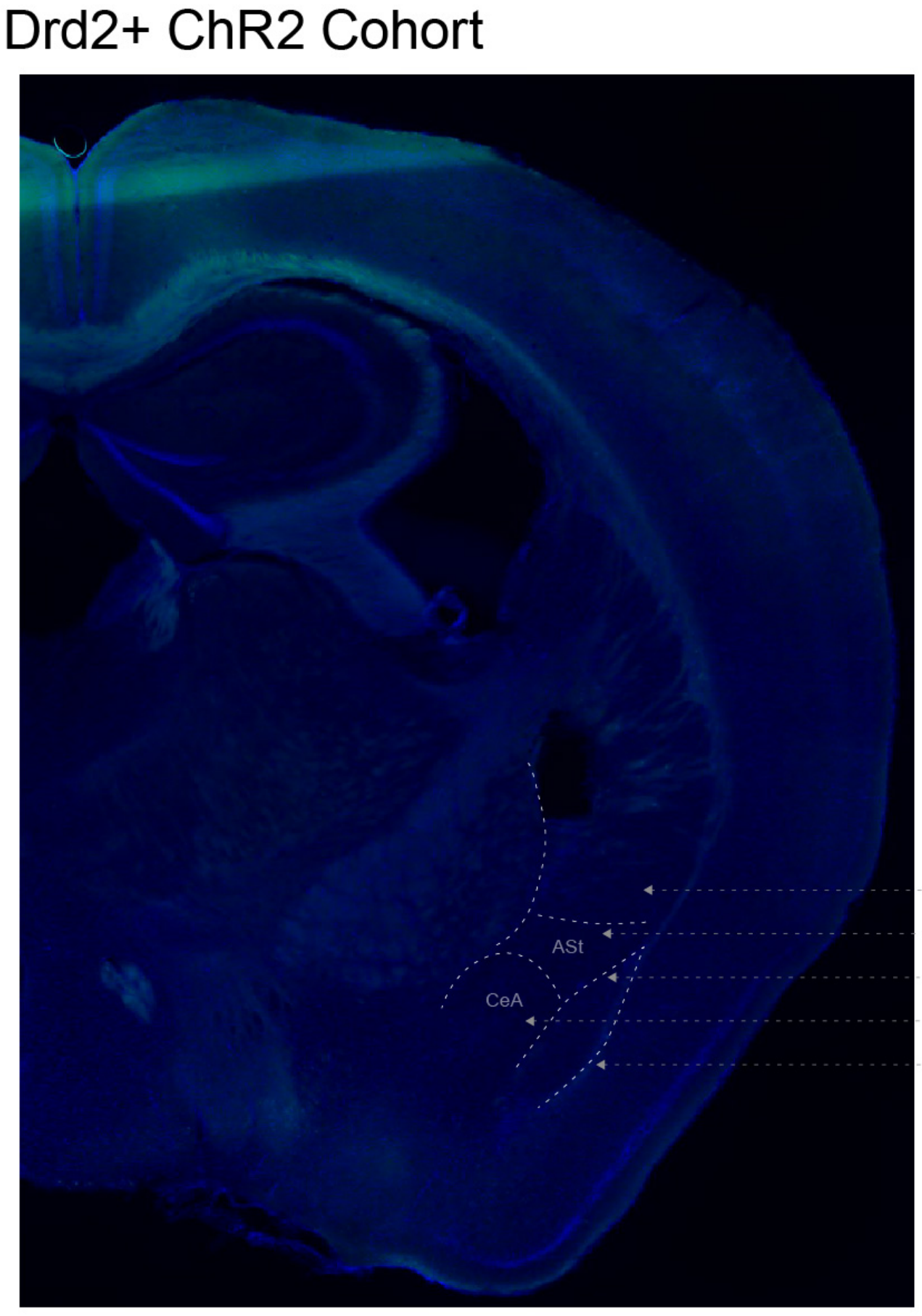

Data from this mouse were not included in the study

- TS: No expression ✓
- ASt: No expression ✗
- LA: No expression ✓
- CeA: No expression ✓
- BLA: No expression ✓

Appendix C - Histology - *Drd2-Cre* mice, ChR2 experiments:

Drd2+ ChR2 Cohort "Mouse ID: D2BJ126R"

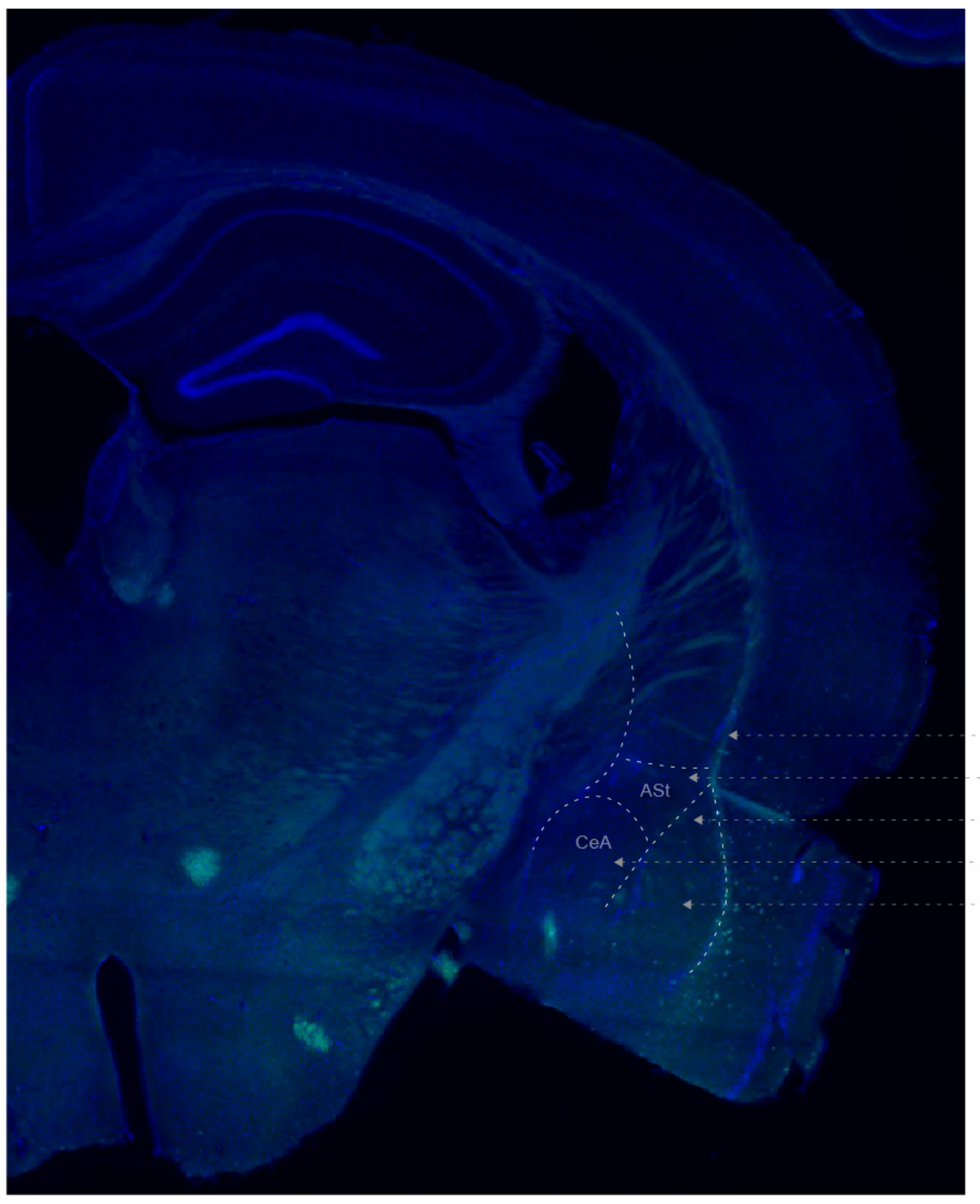

Data from this mouse were not included in the study

- TS: No expression ✓
- ASt: No expression ✗
- LA: No expression ✓
- CeA: No expression ✓
- BLA: Slight expression ✓

Drd2+ ChR2 Cohort "Mouse ID: D2BJ075L"

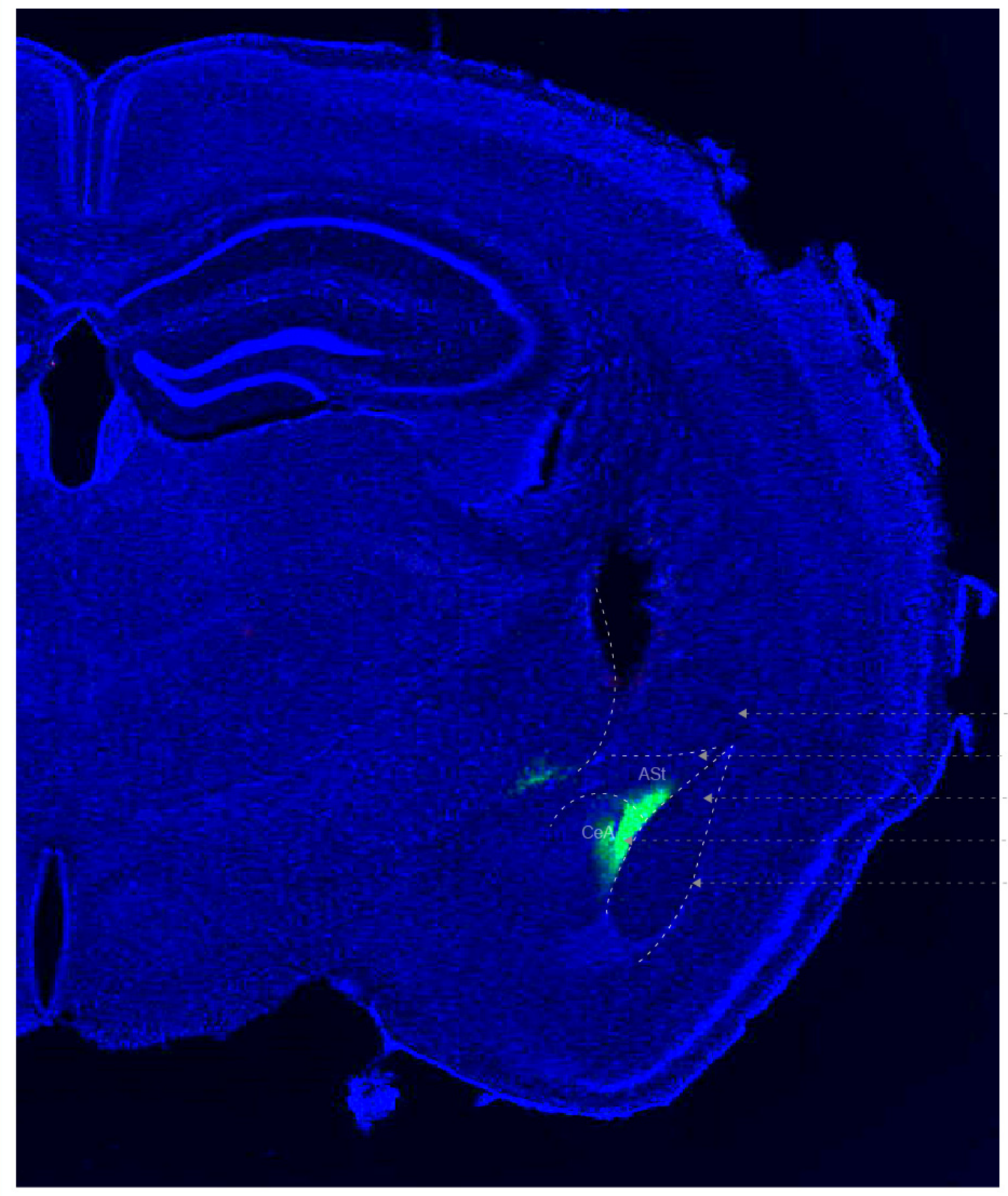

Data from this mouse were not included in the study

- TS: Expression ✓
- ASt: Some expression ✓
- LA: No expression ✓
- CeA: Expression ✗
- BLA: No expression ✓

Drd2+ ChR2 Cohort "Mouse ID: D2AJ075R"

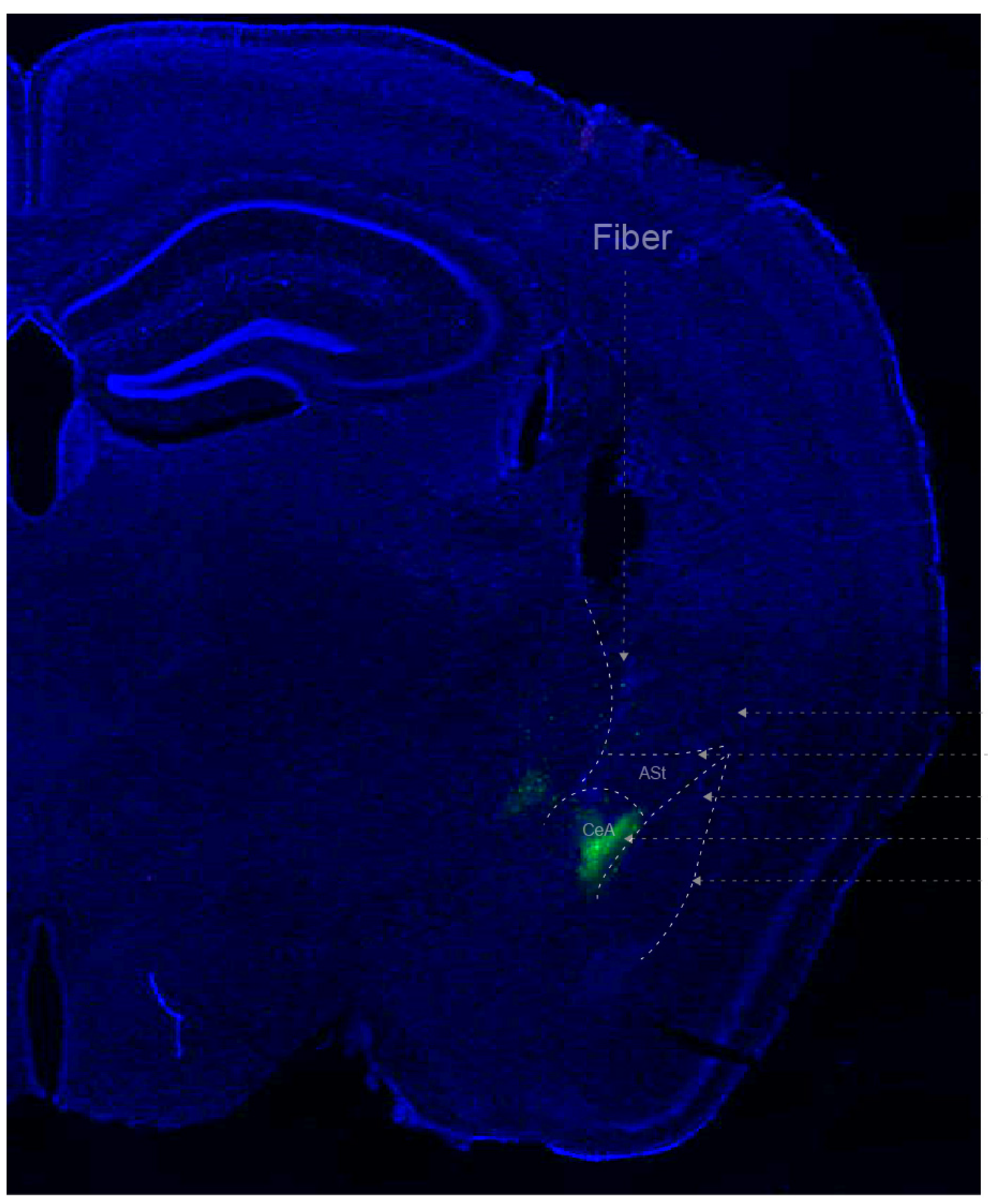

Data from this mouse were not included in the study

- TS: Expression ✓
- ASt: Some expression ✓
- LA: No expression ✓
- CeA: Expression ✗
- BLA: No expression ✓

Drd2+ ChR2 Cohort "Mouse ID: D2AJ054A"

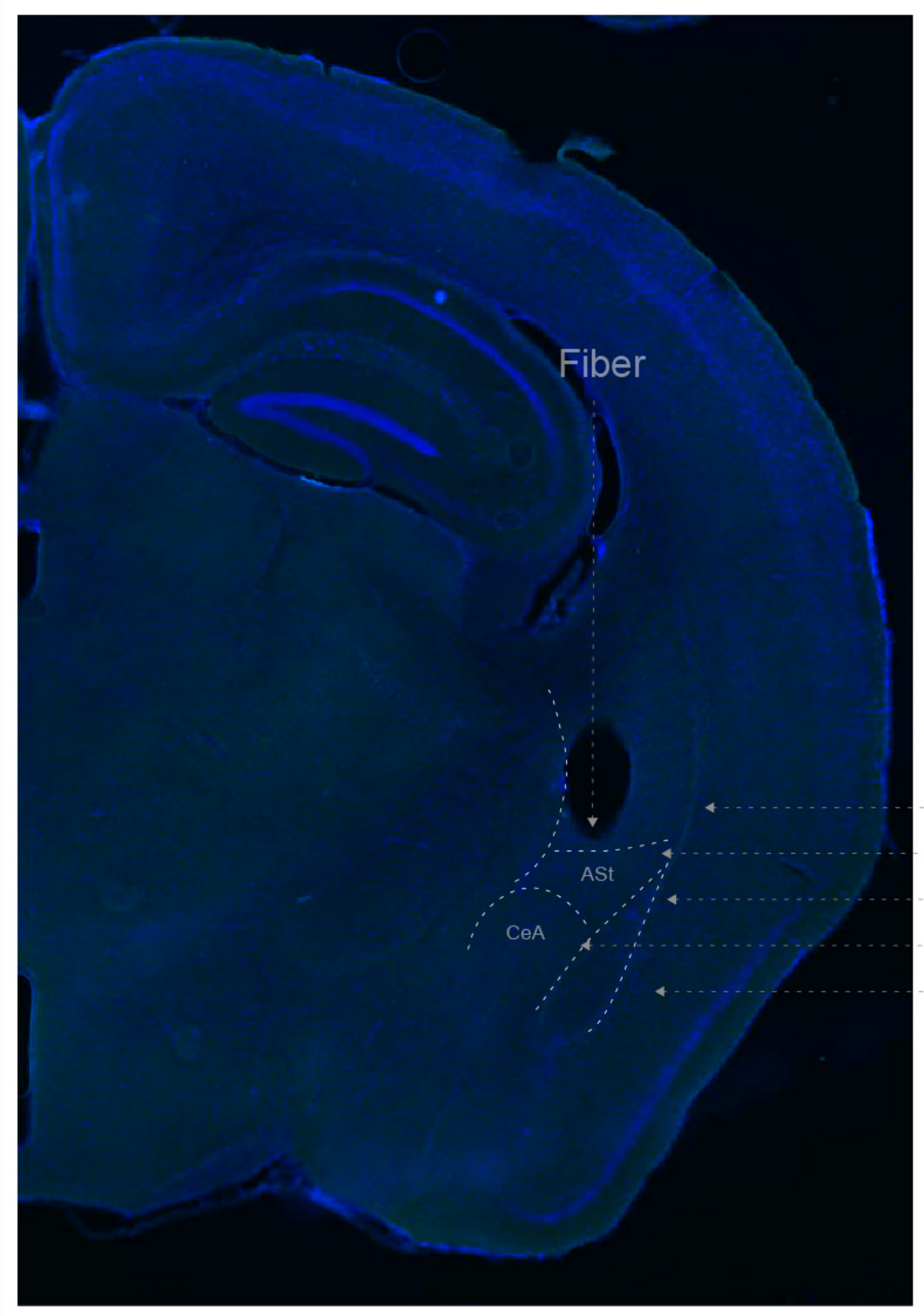

Data from this mouse were not included in the study

- TS: No expression ✓
- ASt: No expression ✗
- LA: No expression ✓
- CeA: No expression ✓
- BLA: No expression ✓

Drd2+ ChR2 Cohort "Mouse ID: D2AJ051B"

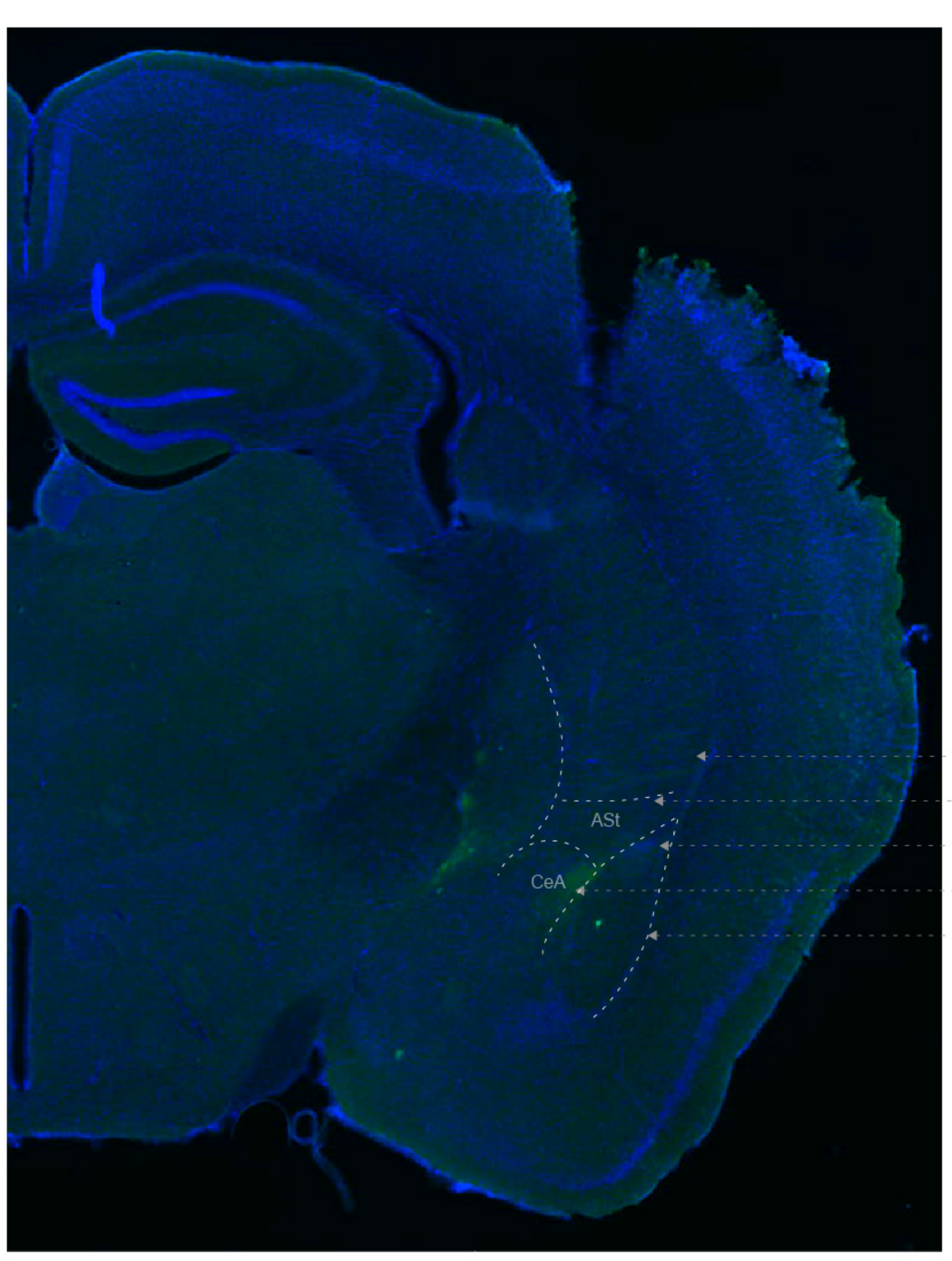

Data from this mouse were not included in the study

- TS: Expression ✓
- ASt: Some expression ✓
- LA: No expression ✓
- CeA: Expression ✗
- BLA: No expression ✓

Drd2+ ChR2 Cohort "Mouse ID: D2AJ054B"

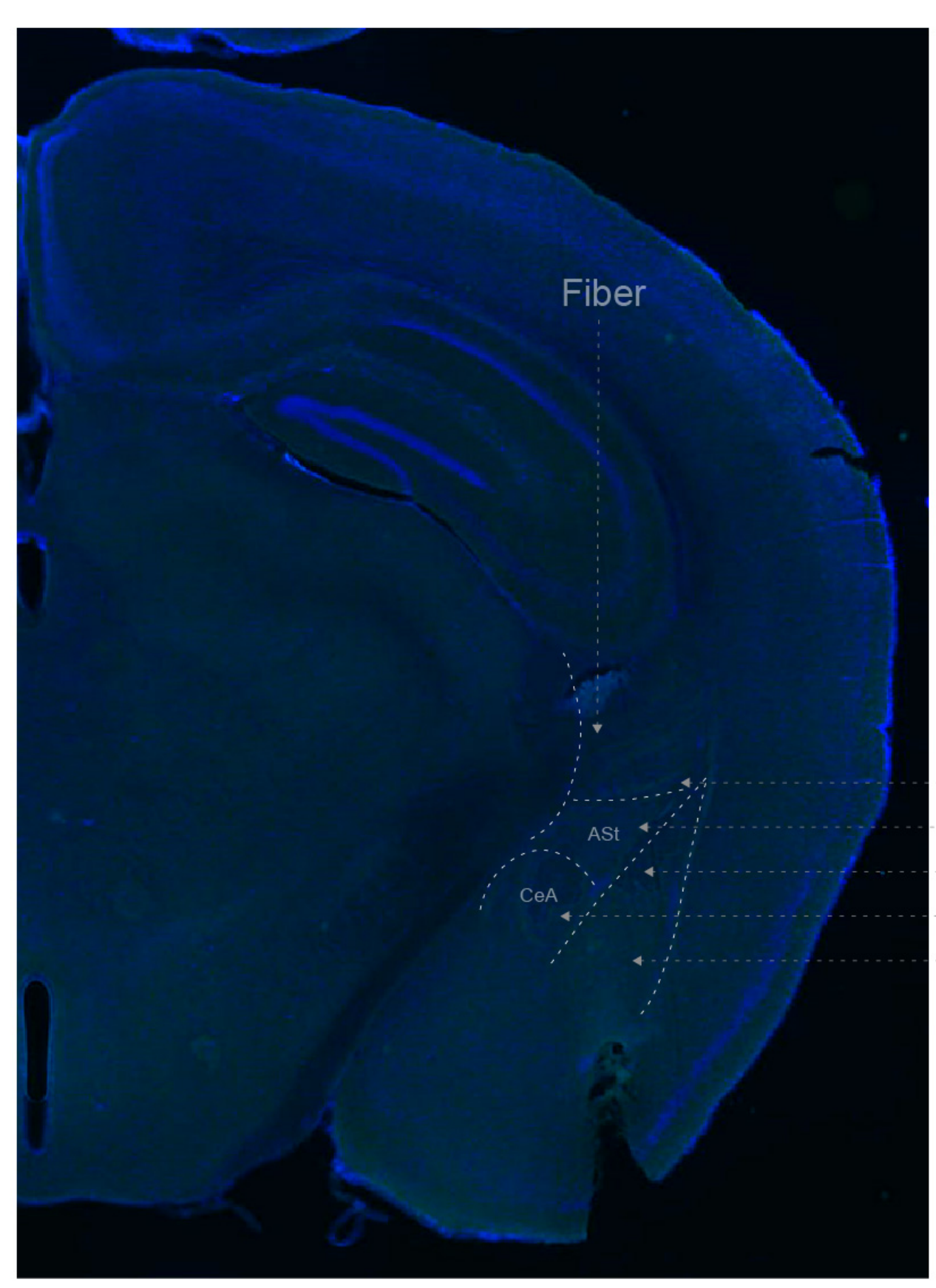

Data from this mouse were not included in the study

- TS: No expression ✓
- ASt: No expression ✗
- LA: No expression ✓
- CeA: No expression ✓
- BLA: No expression ✓

Appendix C - Histology - *Drd2-Cre* mice, ChR2 experiments:

Drd2+ ChR2 Cohort

"Mouse ID: D2AJ997A"

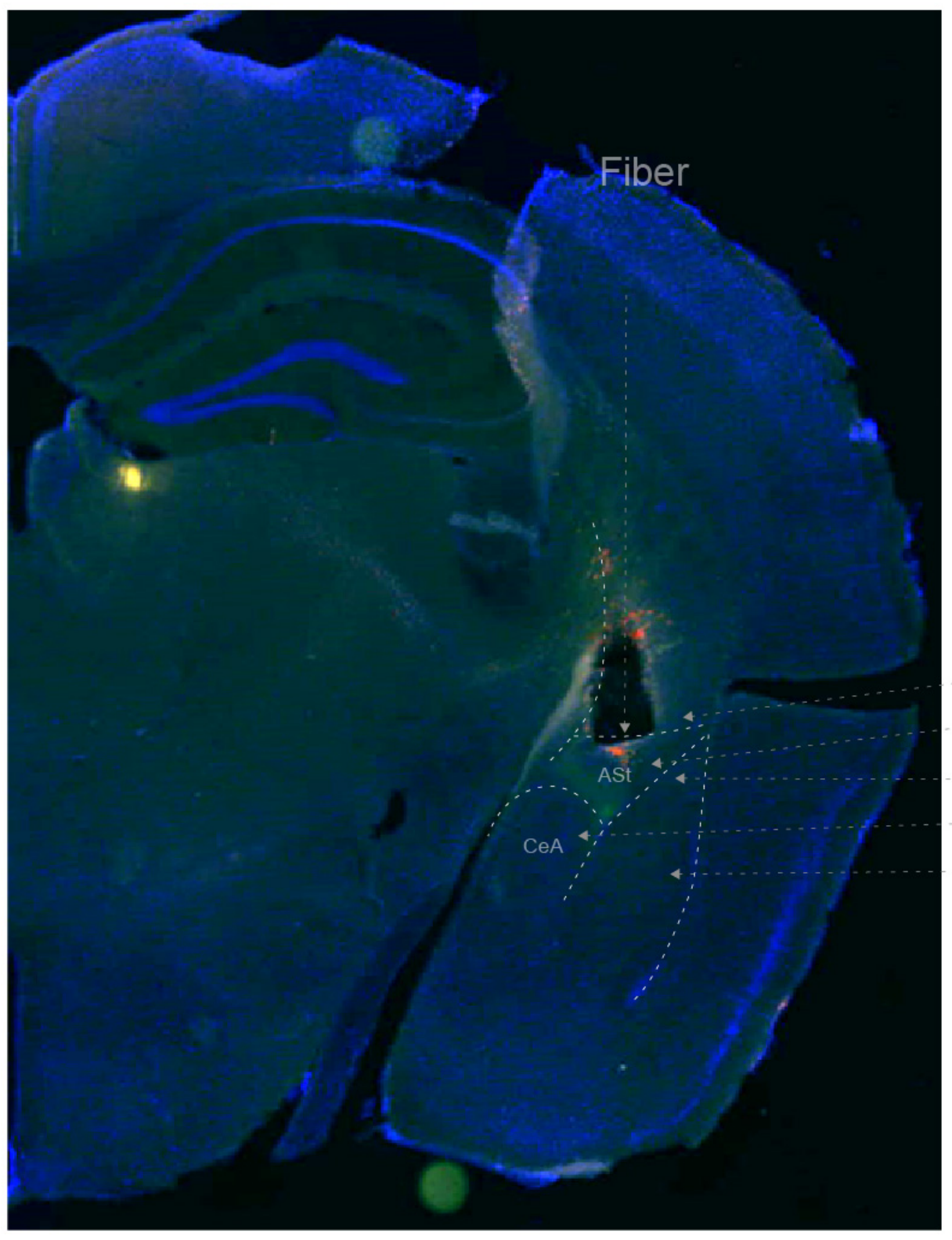

Data from this mouse were not included in the study

- TS: Some expression ✓
- ASt: Ok expression ✓
- LA: Low expression ✓
- CeA: Expression ✗
- BLA: No expression ✓

Drd2+ ChR2 Cohort

"Mouse ID: D2AJ997B"

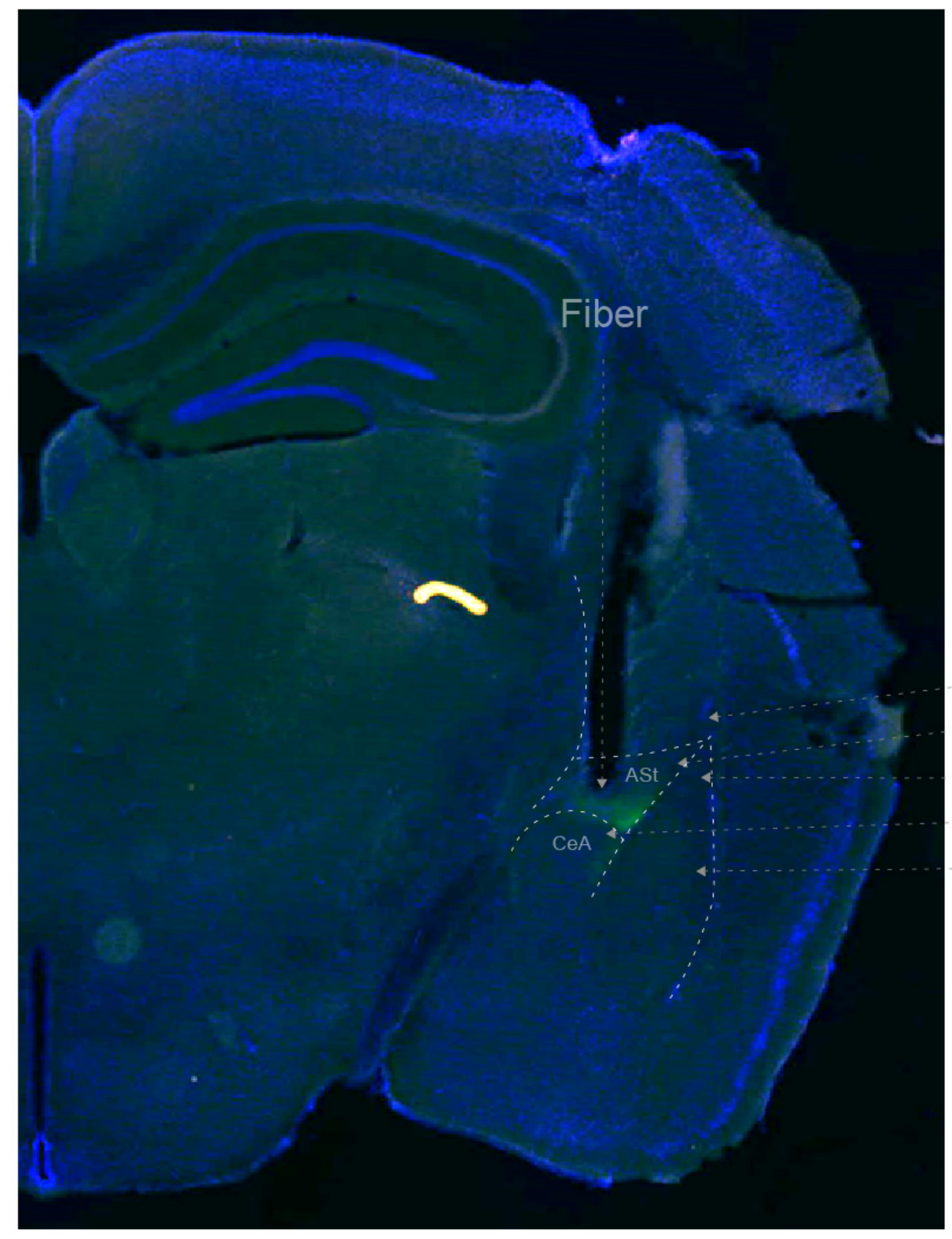

Data from this mouse were not included in the study

- TS: No expression ✓
- ASt: Ok expression ✓
- LA: Low expression ✓
- CeA: Expression ✗
- BLA: No expression ✓

Drd2+ ChR2 Cohort

"Mouse ID: D2AJ046B"

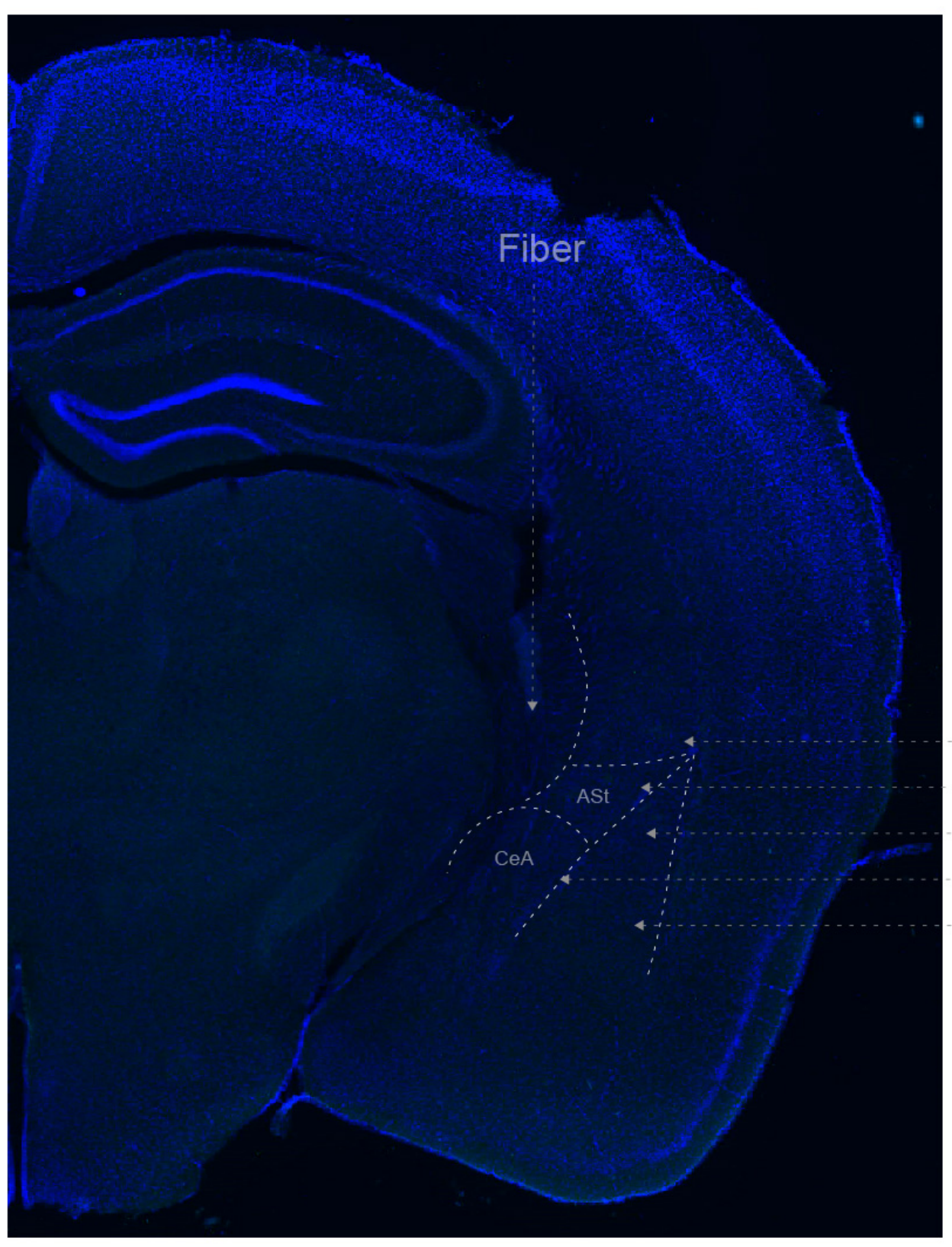

Data from this mouse were not included in the study

- TS: No expression ✓
- ASt: No expression ✗
- LA: No expression ✓
- CeA: No expression ✓
- BLA: No expression ✓

Appendix C - Histology - *Drd2-Cre* mice, ChR2 experiments:

Drd2+ ChR2 Cohort

"Mouse ID: D2BJ095L"

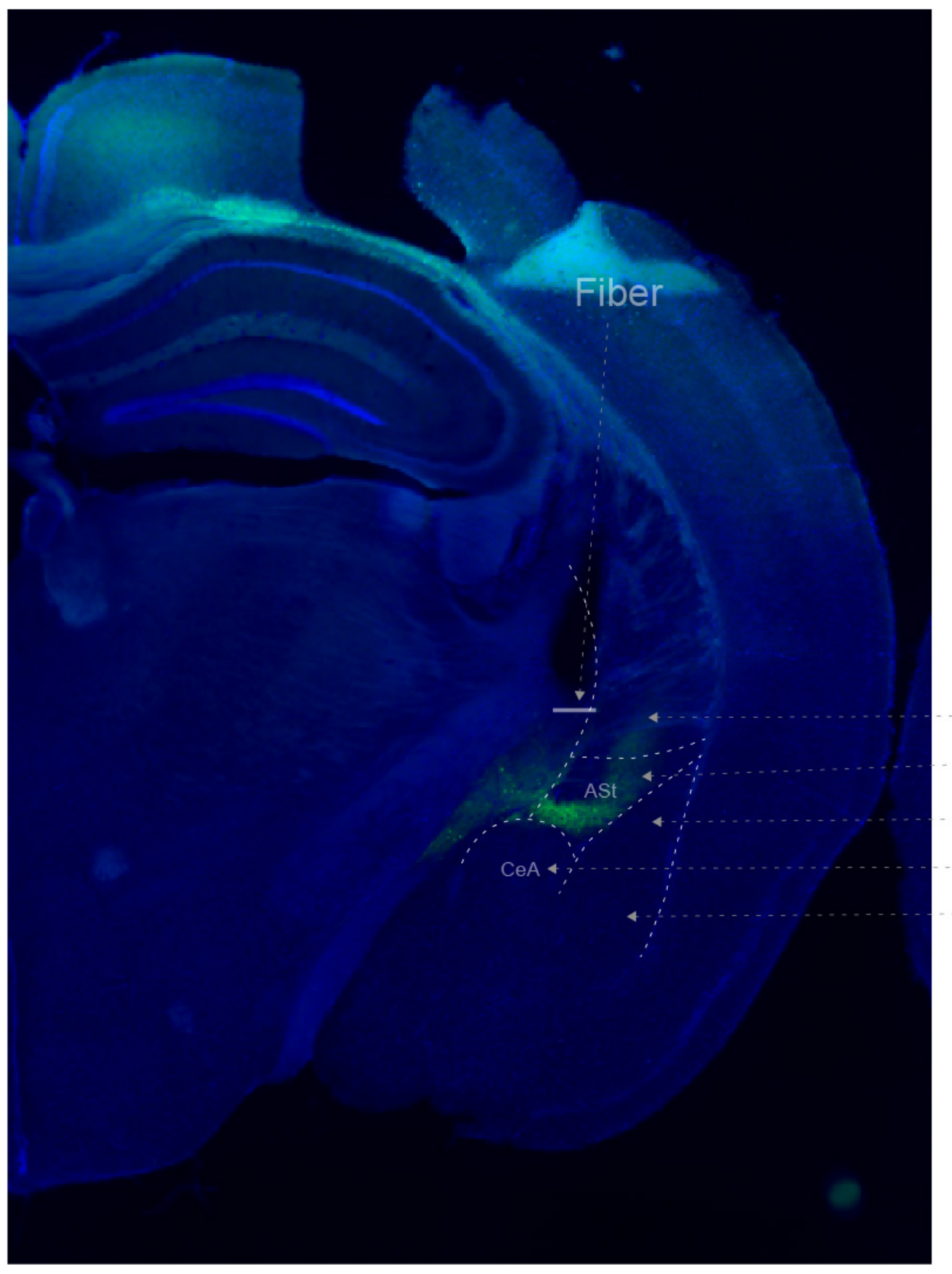

Histology okay, data included

- TS: No expression ✓
- ASt: Good expression ✓
- LA: No expression ✓
- CeA: No expression ✓
- BLA: No expression ✓

Drd2+ ChR2 Cohort

"Mouse ID: D2BJ060R"

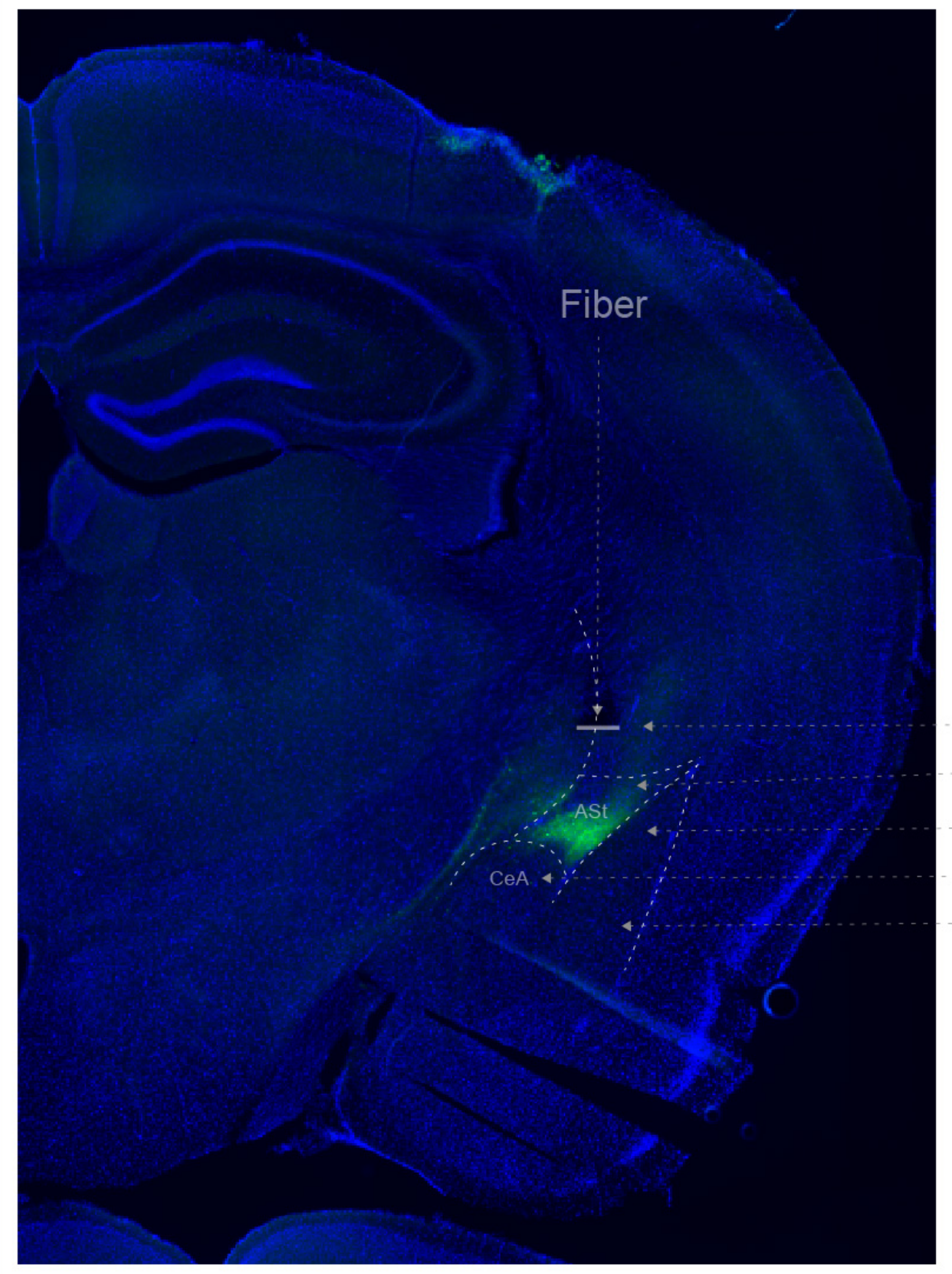

Histology okay, data included

- TS: Very little expression ✓
- ASt: Good expression ✓
- LA: No expression ✓
- CeA: No expression ✓
- BLA: No expression ✓

Drd2+ ChR2 Cohort

"Mouse ID: D2BJ061L"

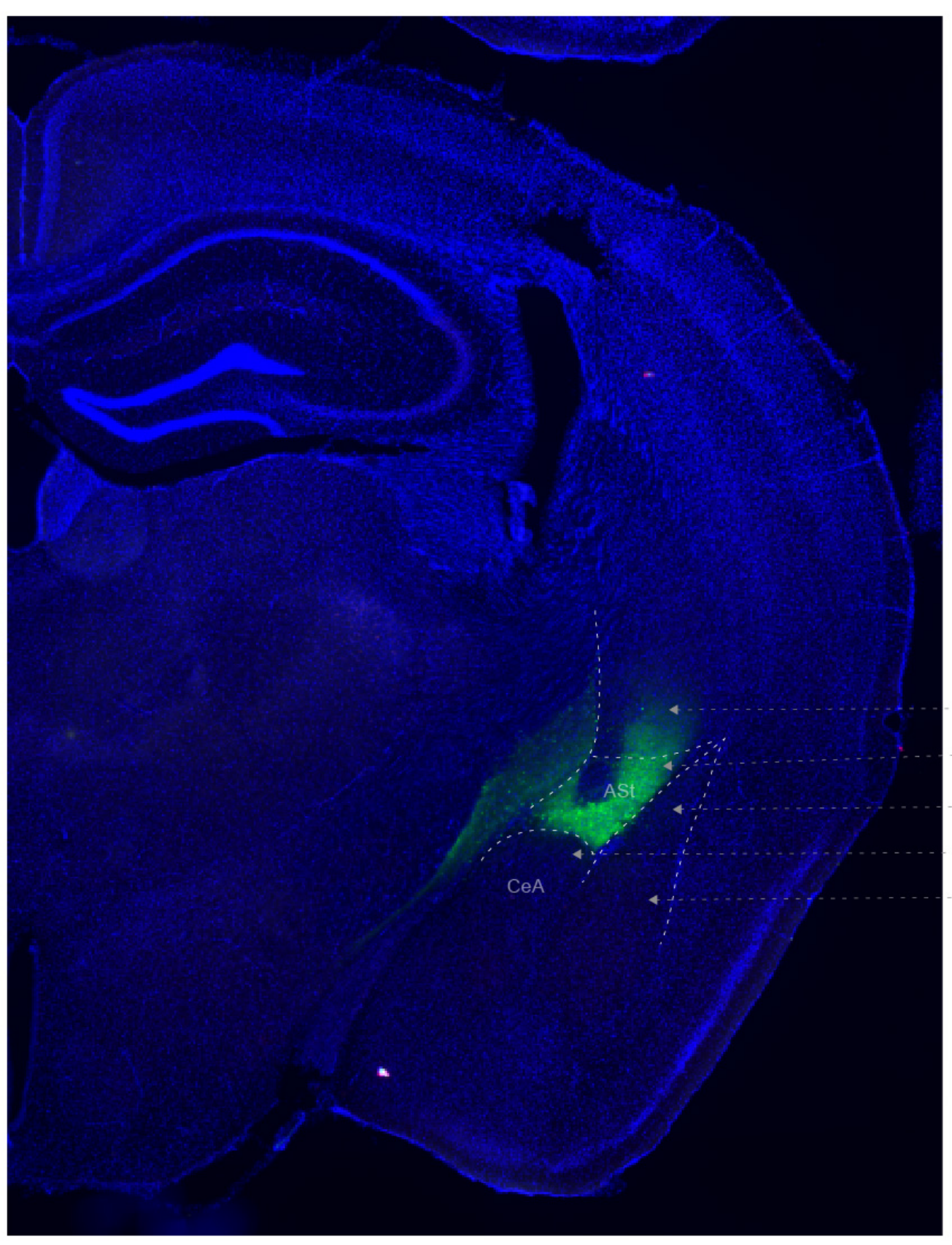

Histology okay, data included

- TS: Very little expression ✓
- ASt: Good expression ✓
- LA: No expression ✓
- CeA: No expression ✓
- BLA: No expression ✓

Drd2+ ChR2 Cohort

"Mouse ID: D2BJ152L"

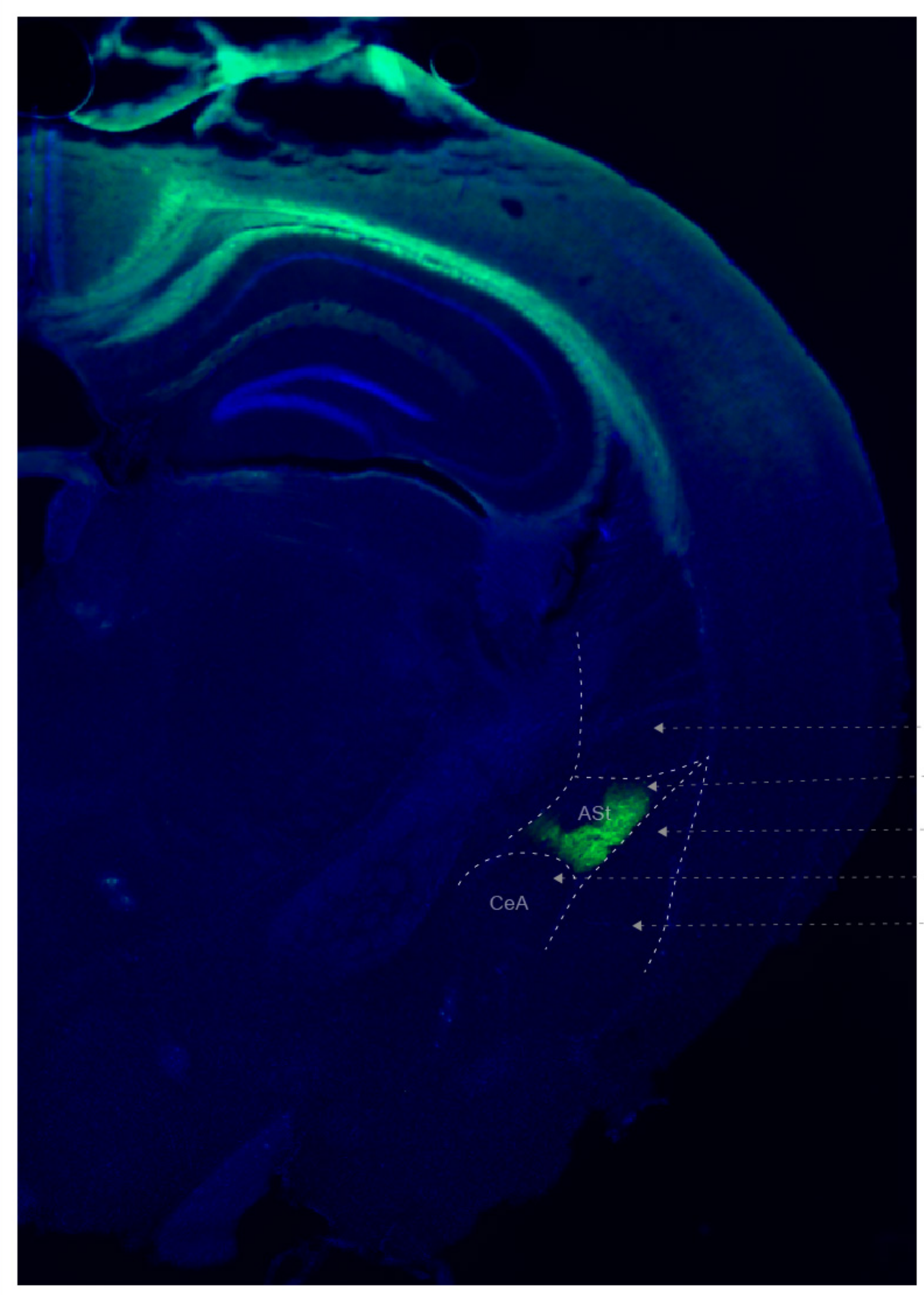

Histology okay, data included

- TS: No expression ✓
- ASt: Good expression ✓
- LA: No expression ✓
- CeA: No expression ✓
- BLA: No expression ✓

Drd2+ ChR2 Cohort

"Mouse ID: D2BJ152R"

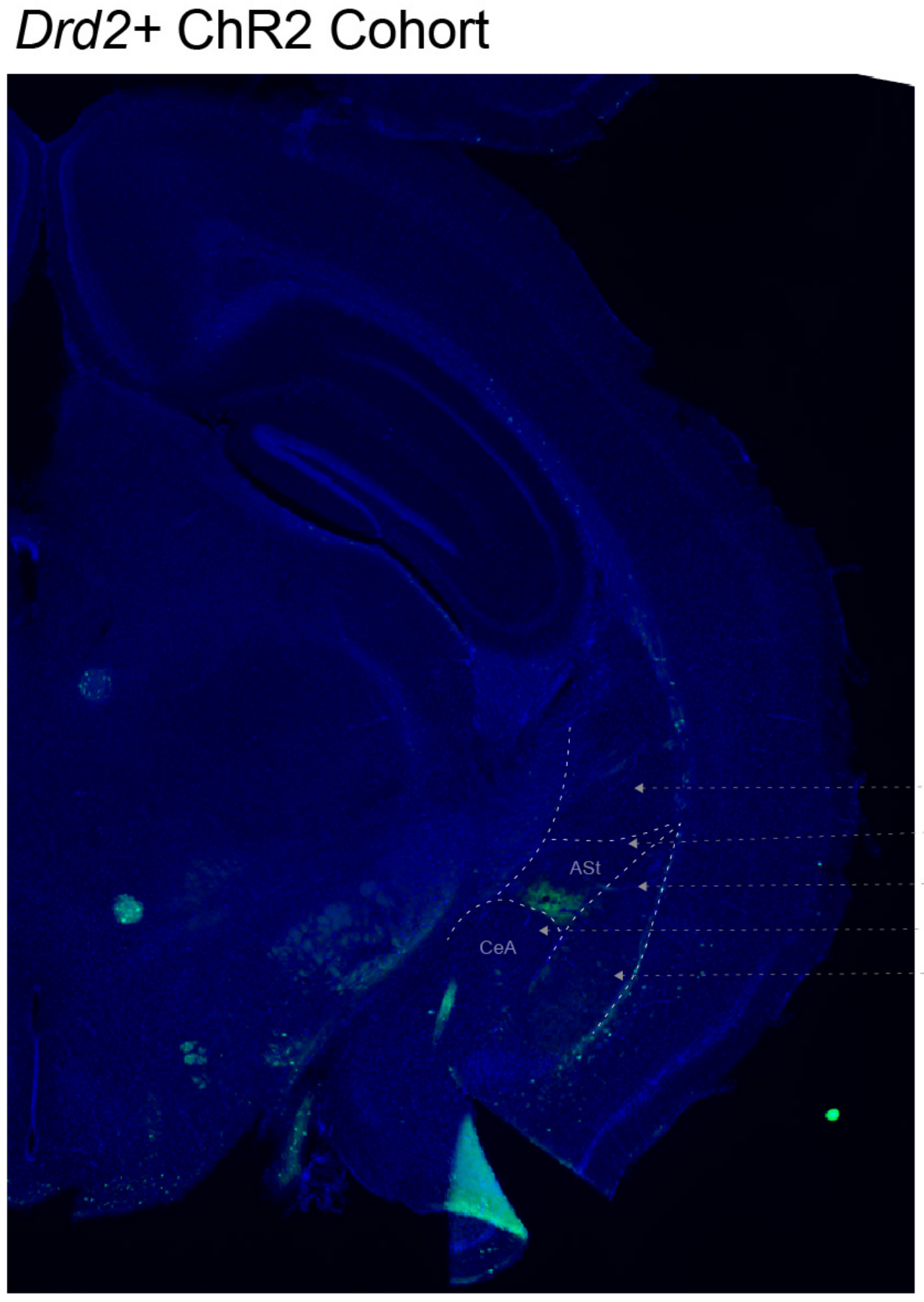

Histology okay, data included

- TS: No expression ✓
- ASt: Good expression ✓
- LA: No expression ✓
- CeA: No expression ✓
- BLA: No expression ✓

Drd2+ ChR2 Cohort

"Mouse ID: D2AJ962B"

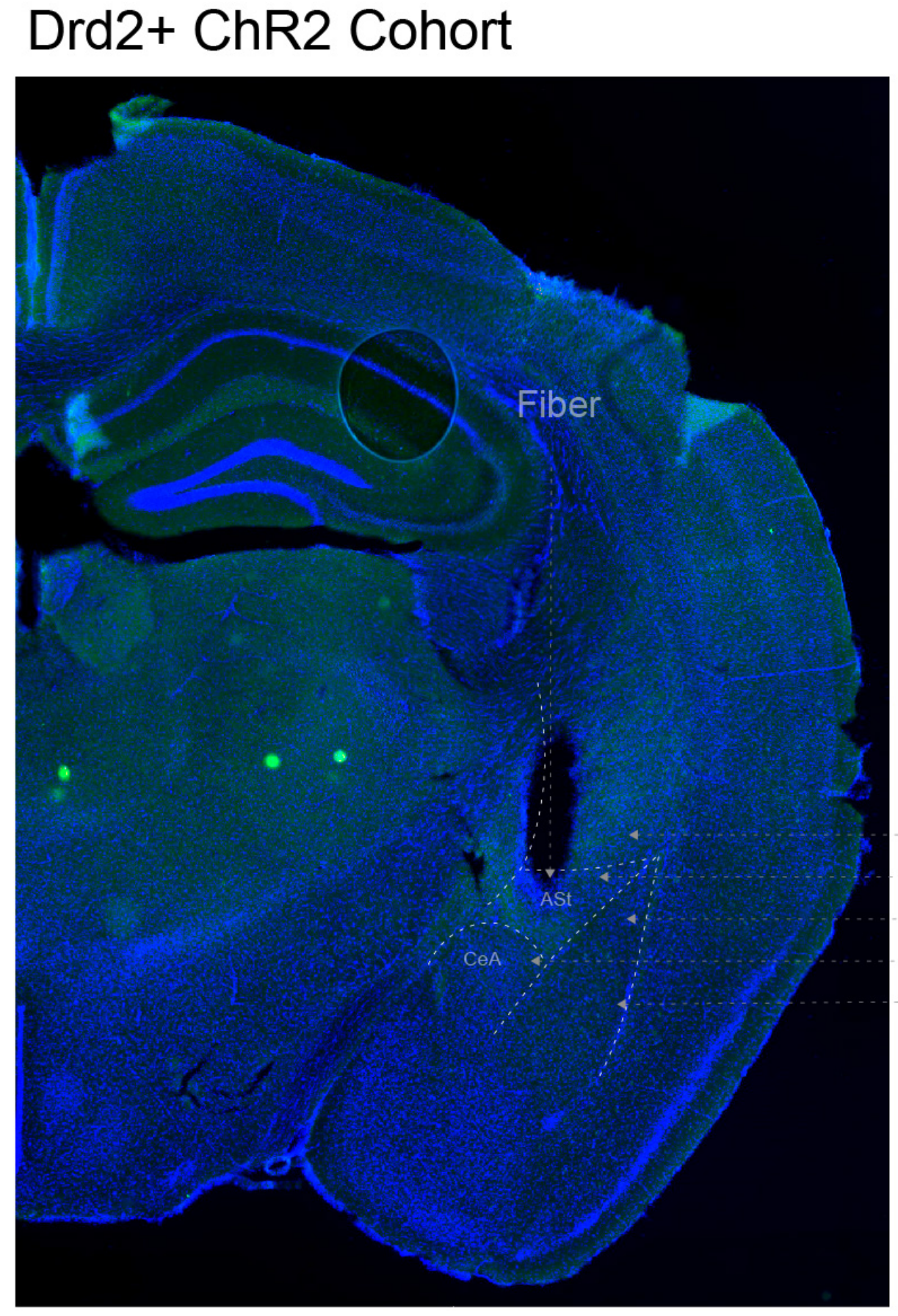

Histology okay, data included

- TS: Almost no expression ✓
- ASt: Low but ok expression ✓
- LA: No expression ✓
- CeA: No expression ✓
- BLA: No expression ✓

Appendix C - Histology - *Drd2-Cre* mice, ChR2 experiments:

Drd2+ ChR2 Cohort

"Mouse ID: D2AJ962A"

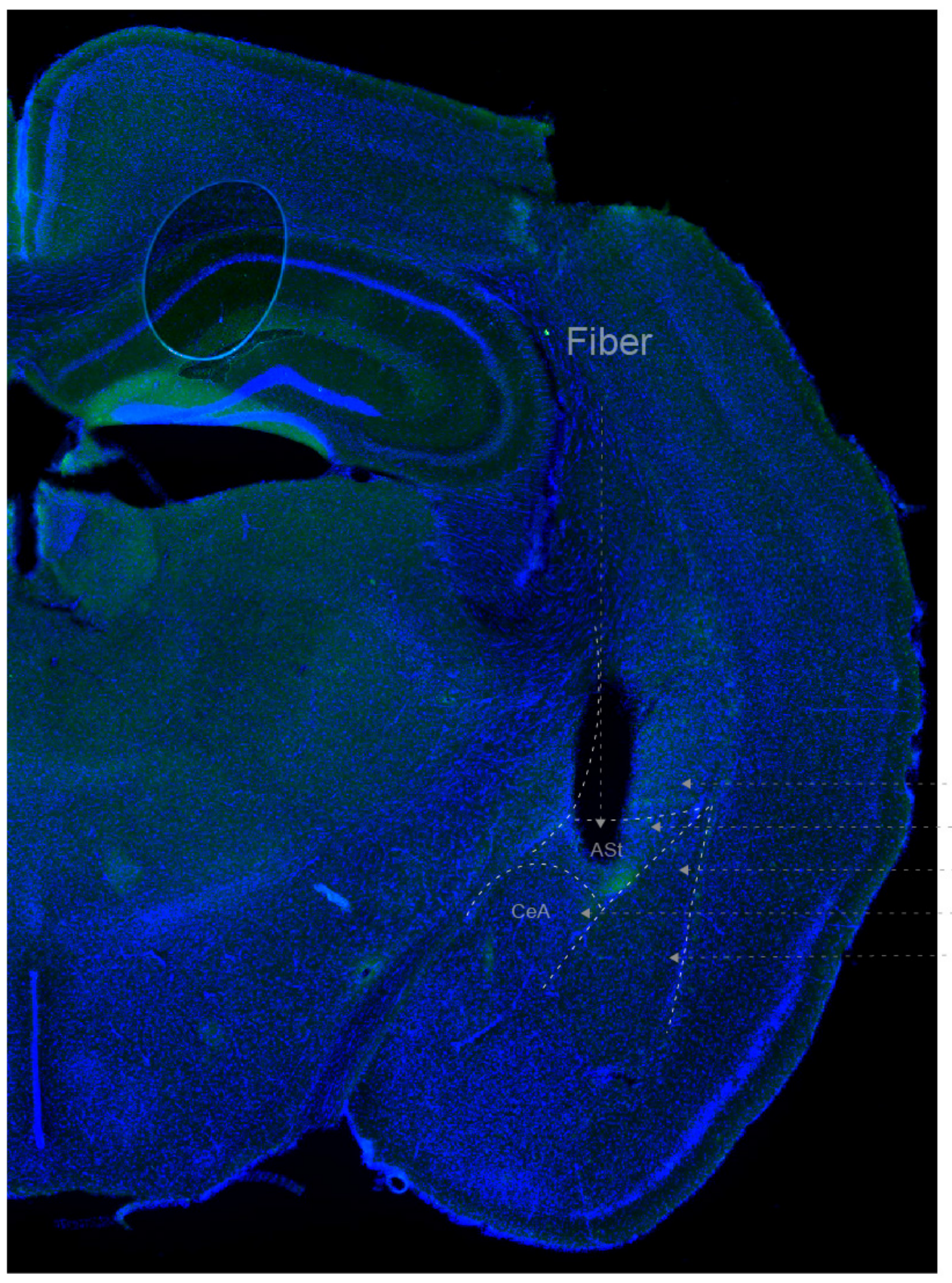

Histology okay, data included

TS: Almost no expression ✓
ASt: Ok expression ✓
LA: No expression ✓
CeA: No expression ✓
BLA: No expression ✓

Drd2+ ChR2 Cohort

"Mouse ID: D2AJ033B"

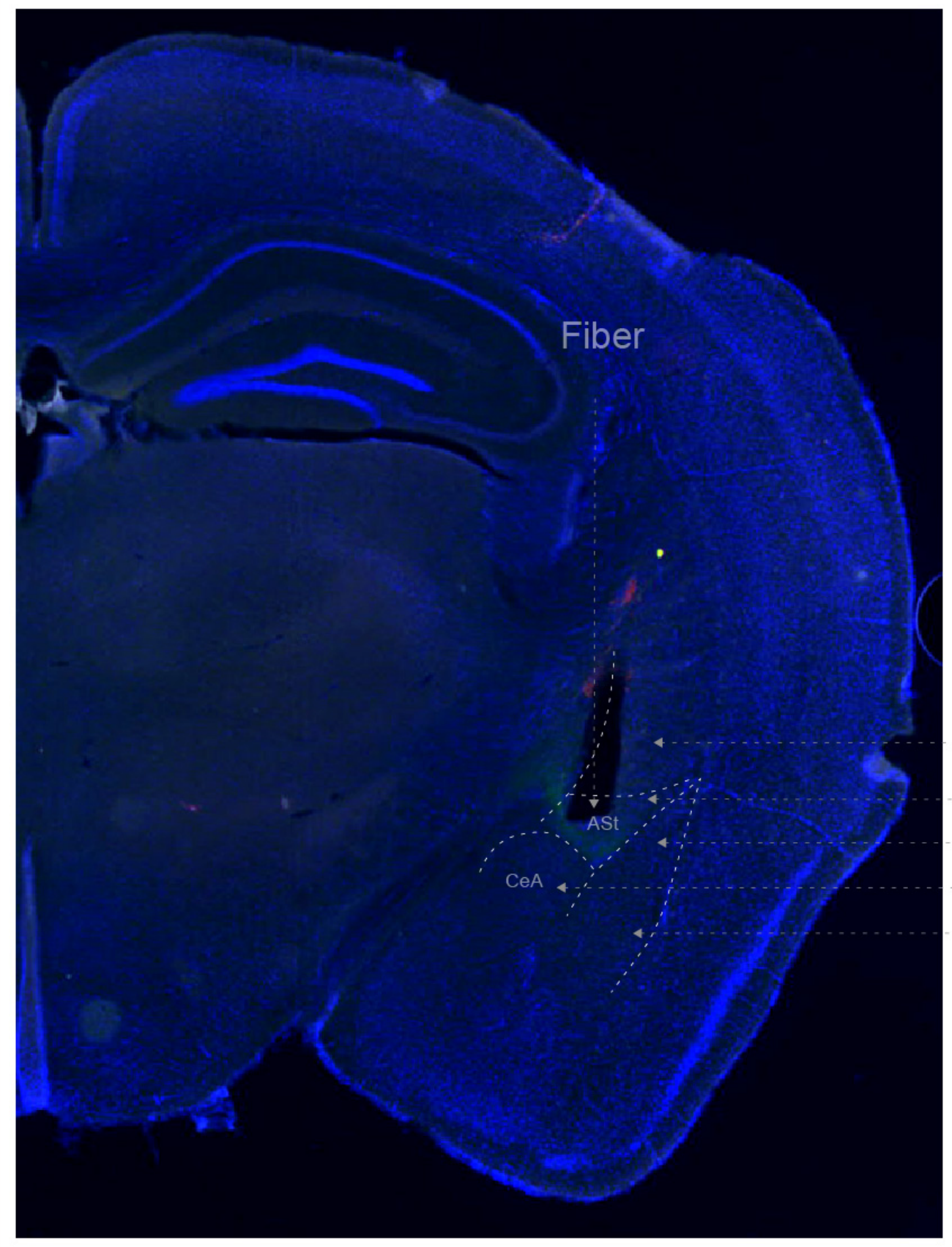

Histology okay, data included

TS: No expression ✓
ASt: Good expression ✓
LA: No expression ✓
CeA: No expression ✓
BLA: No expression ✓

Appendix C - Histology - *D1:Cre* mice, ChR2 experiments:

Drd1a+ ChR2 Cohort

"Mouse ID: D1K115R"

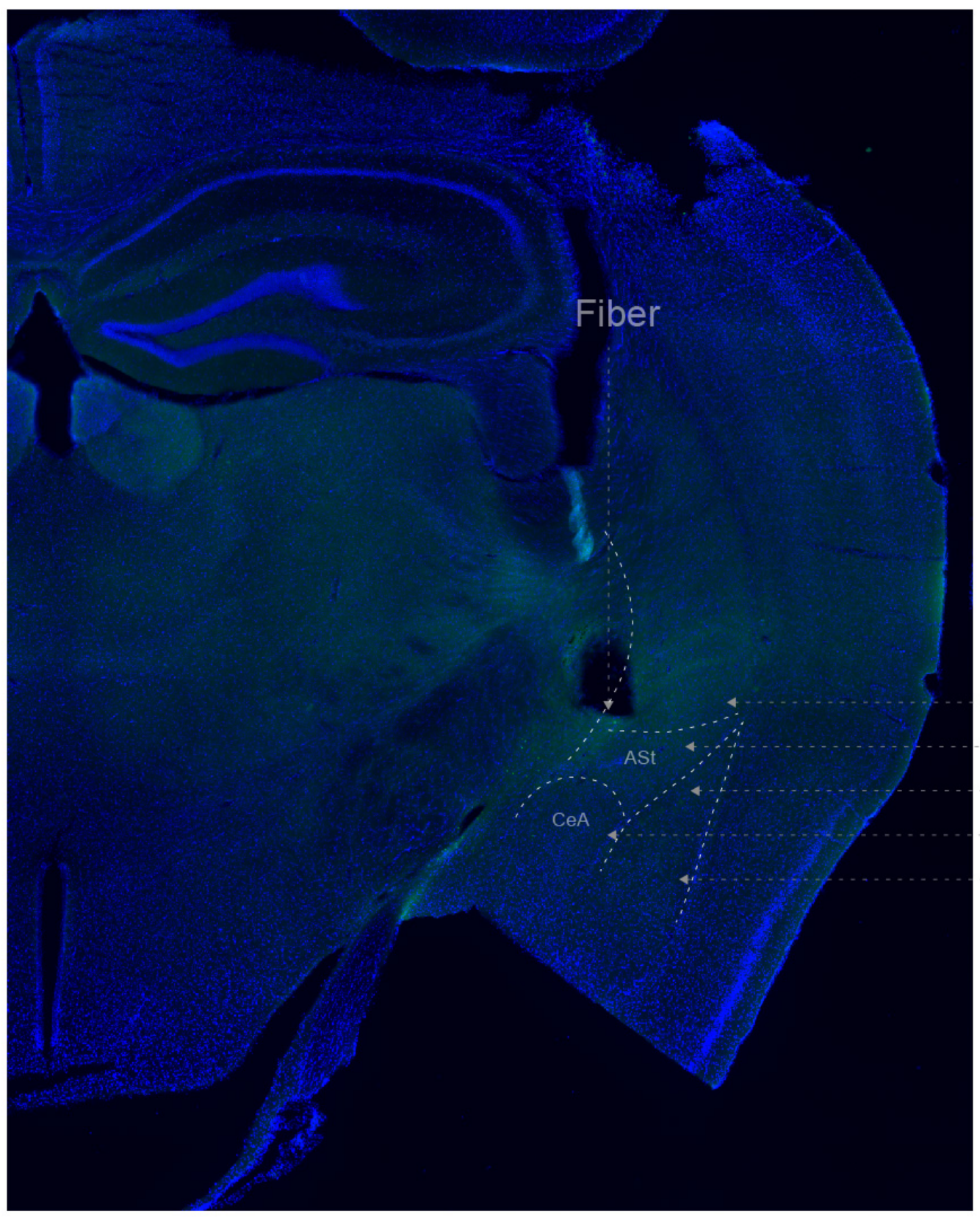

Data from this mouse were not included in the study

TS: No expression ✓
ASt: No expression ✗
LA: No expression ✓
CeA: No expression ✓
BLA: No expression ✓

Drd1a+ ChR2 Cohort

"Mouse ID: D1K115L"

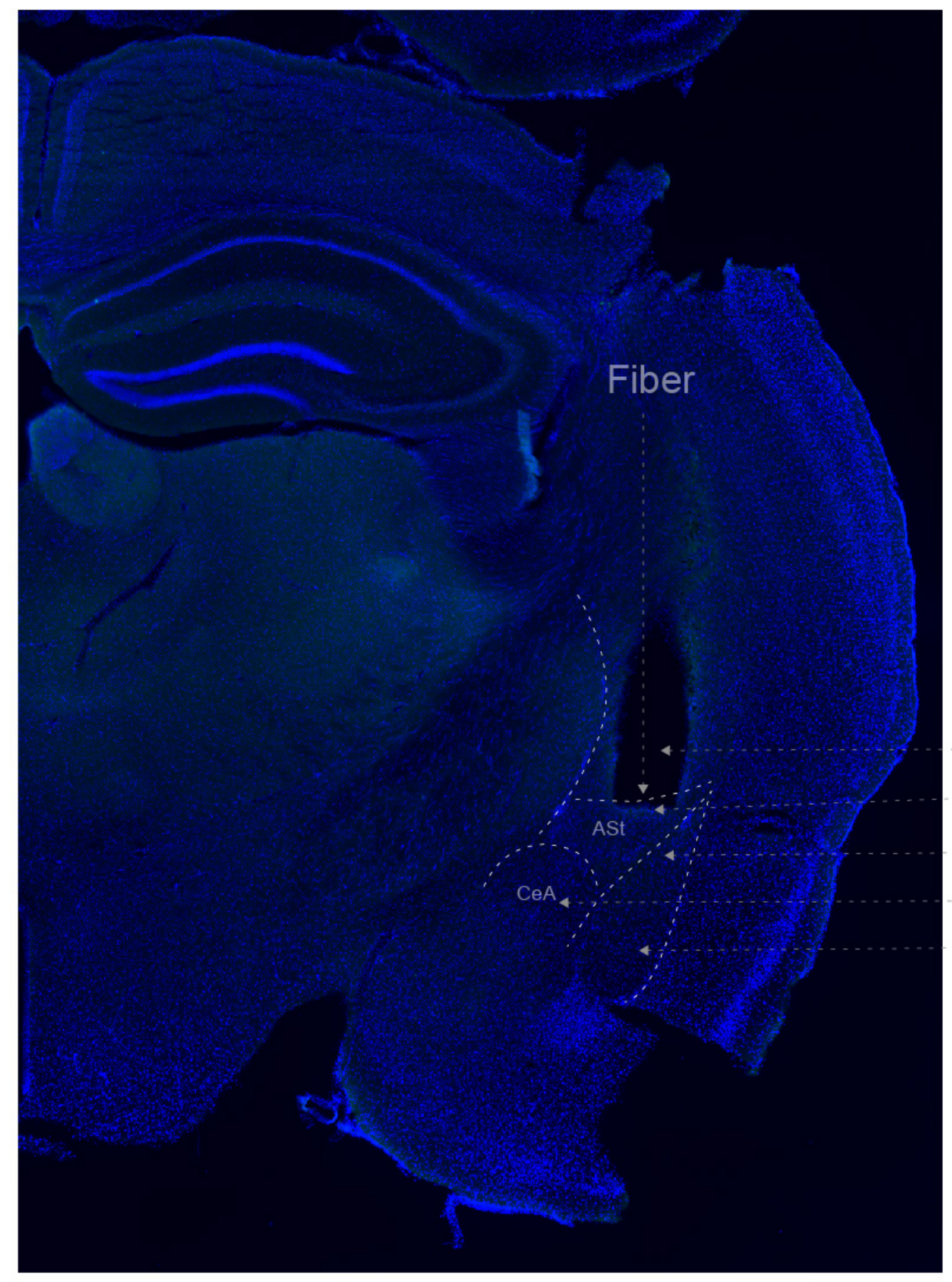

Data from this mouse were not included in the study

TS: No expression ✓
ASt: No expression ✗
LA: No expression ✓
CeA: No expression ✓
BLA: No expression ✓

Drd1a+ ChR2 Cohort

"Mouse ID: D1K159R"

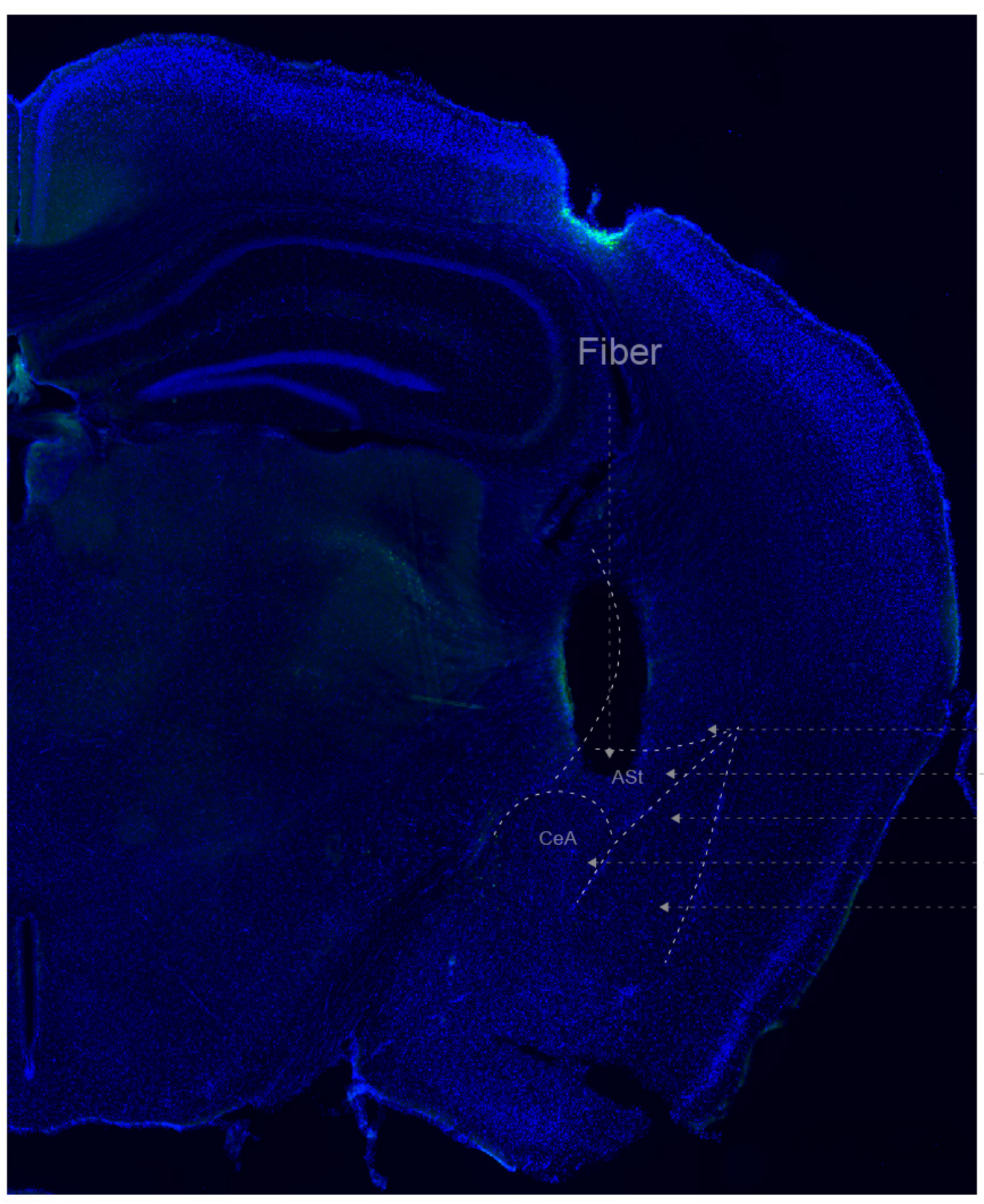

Data from this mouse were not included in the study

TS: No expression ✓
ASt: No expression ✗
LA: No expression ✓
CeA: No expression ✓
BLA: No expression ✓

Drd1a+ ChR2 Cohort

"Mouse ID: D1K107R"

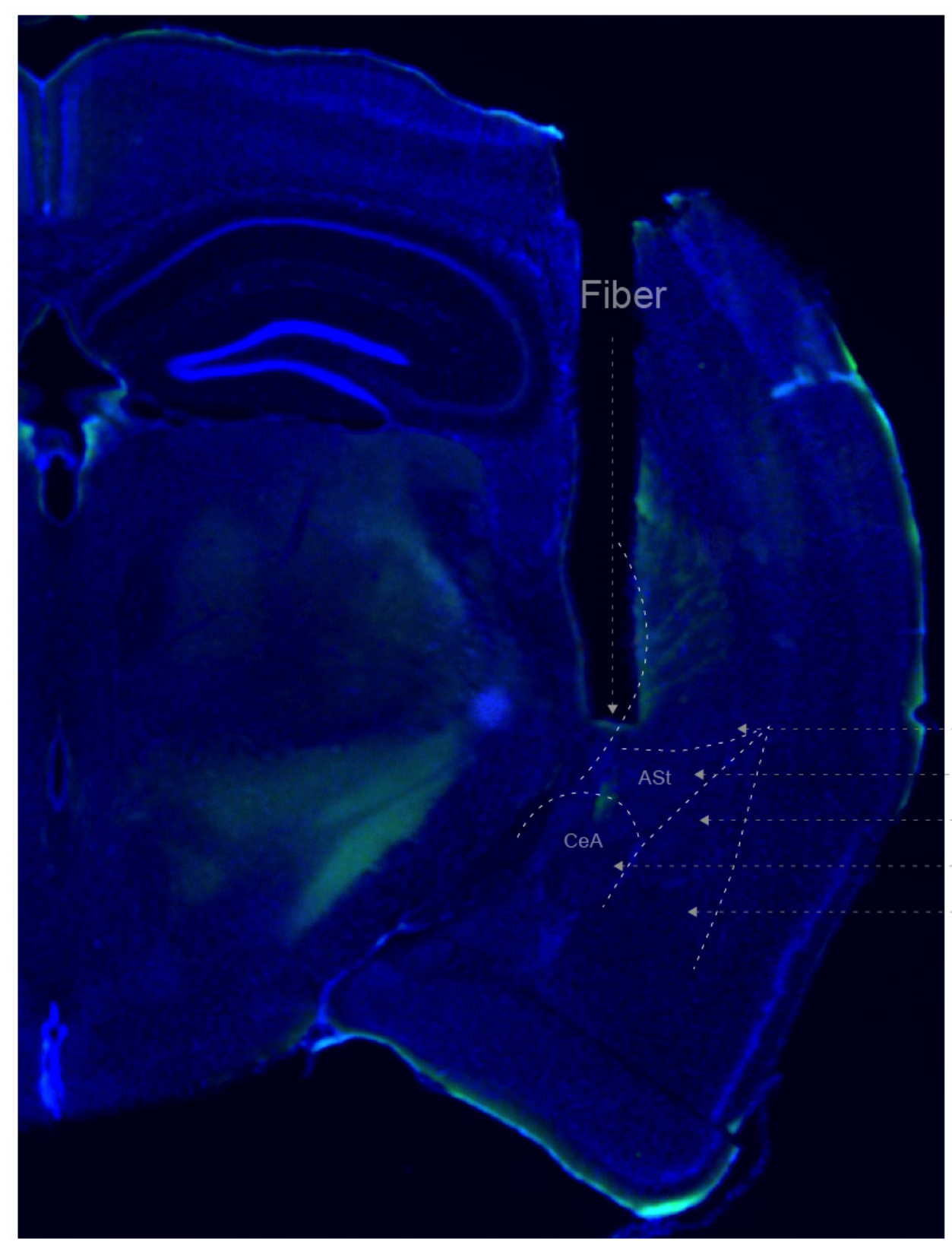

Data from this mouse were not included in the study

TS: No expression ✓
ASt: No expression ✗
LA: No expression ✓
CeA: No expression ✓
BLA: No expression ✓

Drd1a+ ChR2 Cohort

"Mouse ID: D1K157L"

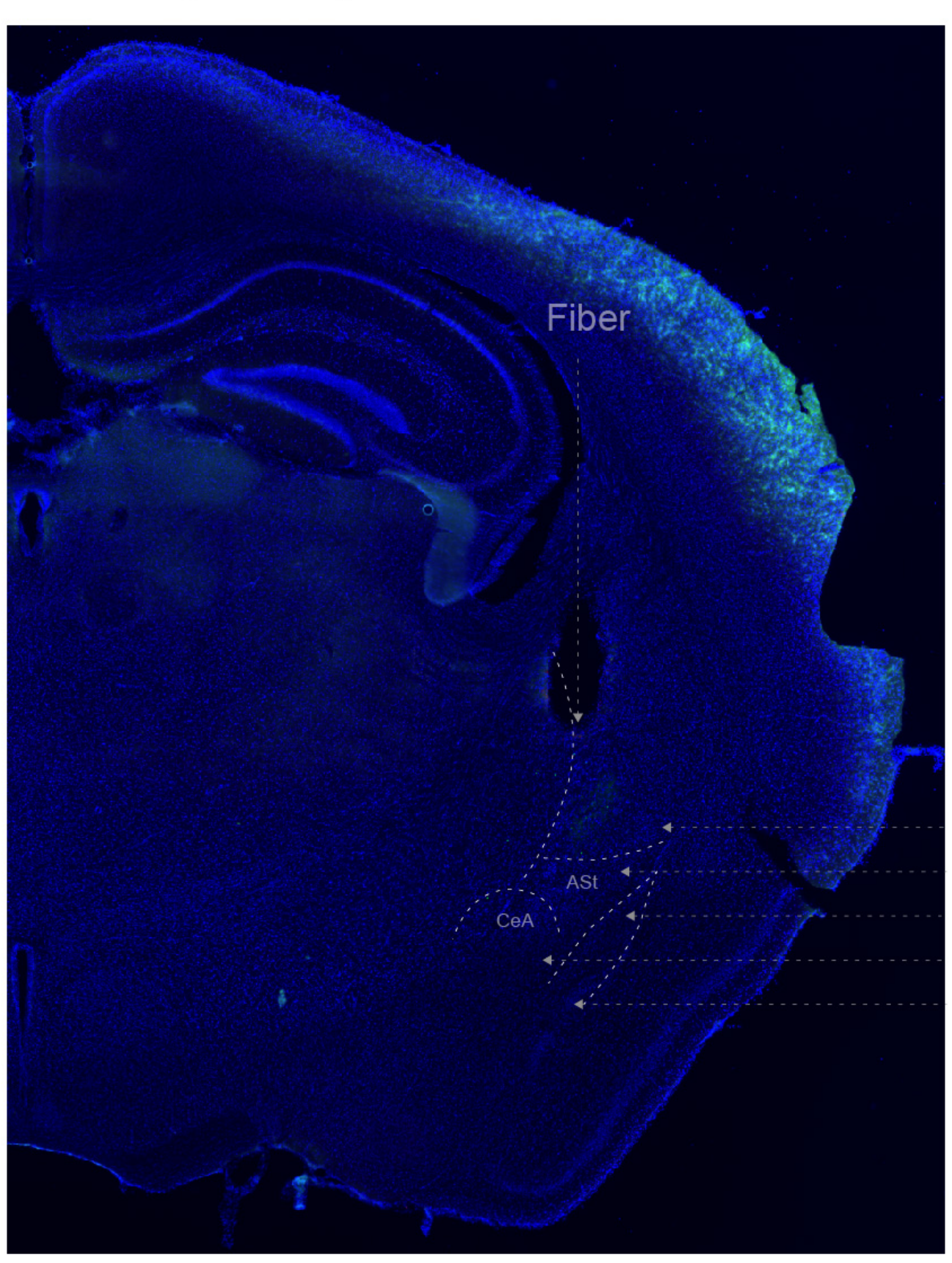

Data from this mouse were not included in the study

TS: No expression ✓
ASt: No expression ✗
LA: No expression ✓
CeA: No expression ✓
BLA: No expression ✓

Drd1a+ ChR2 Cohort

"Mouse ID: D1K133L"

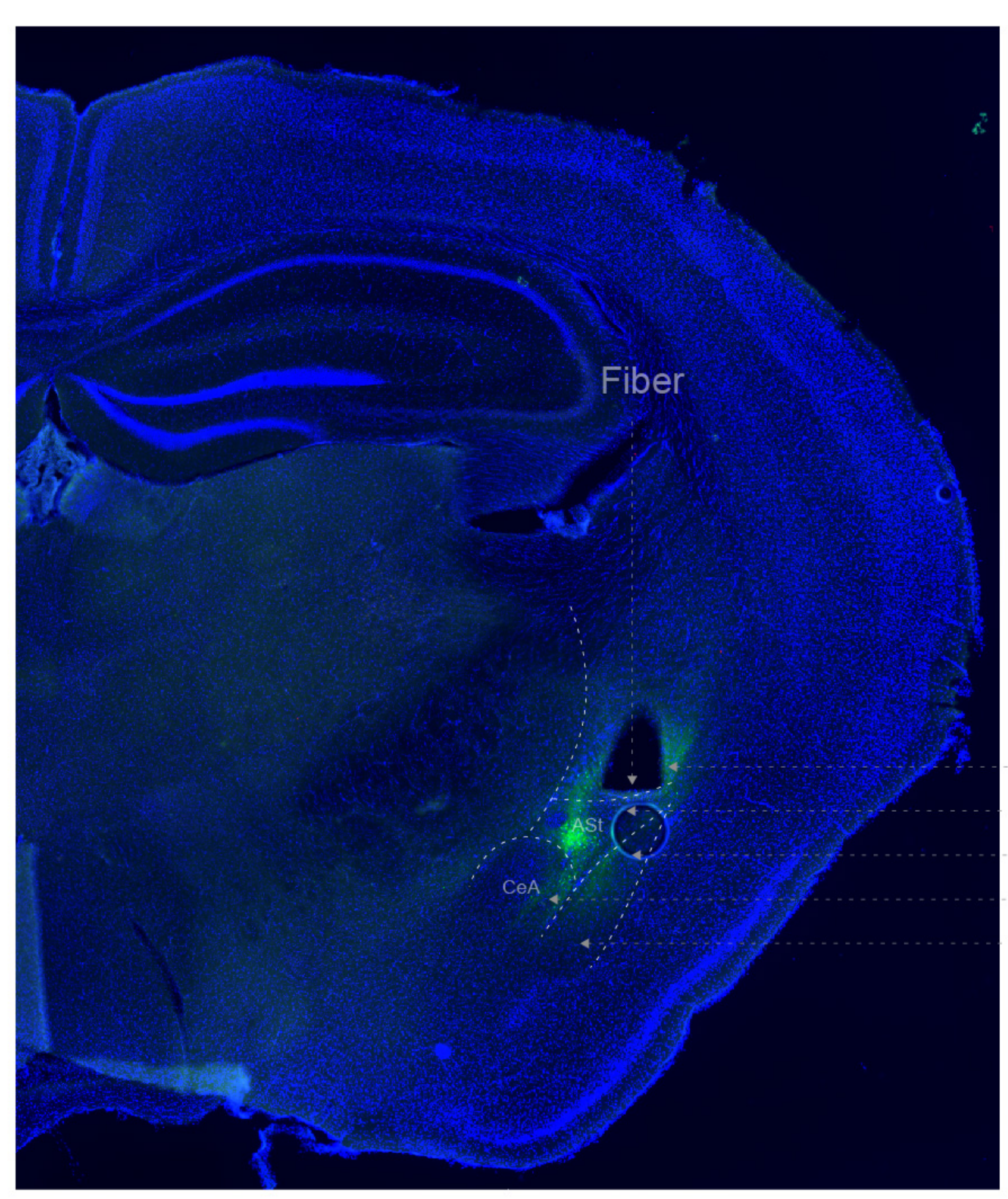

Data from this mouse were not included in the study

TS: Some expression ✓
ASt: Ok expression ✓
LA: No expression ✓
CeA: Expression ✗
BLA: No expression ✓

Appendix C - Histology - *D1:Cre* mice, ChR2 experiments:

Drd1a+ ChR2 Cohort

"Mouse ID: D1K140R"

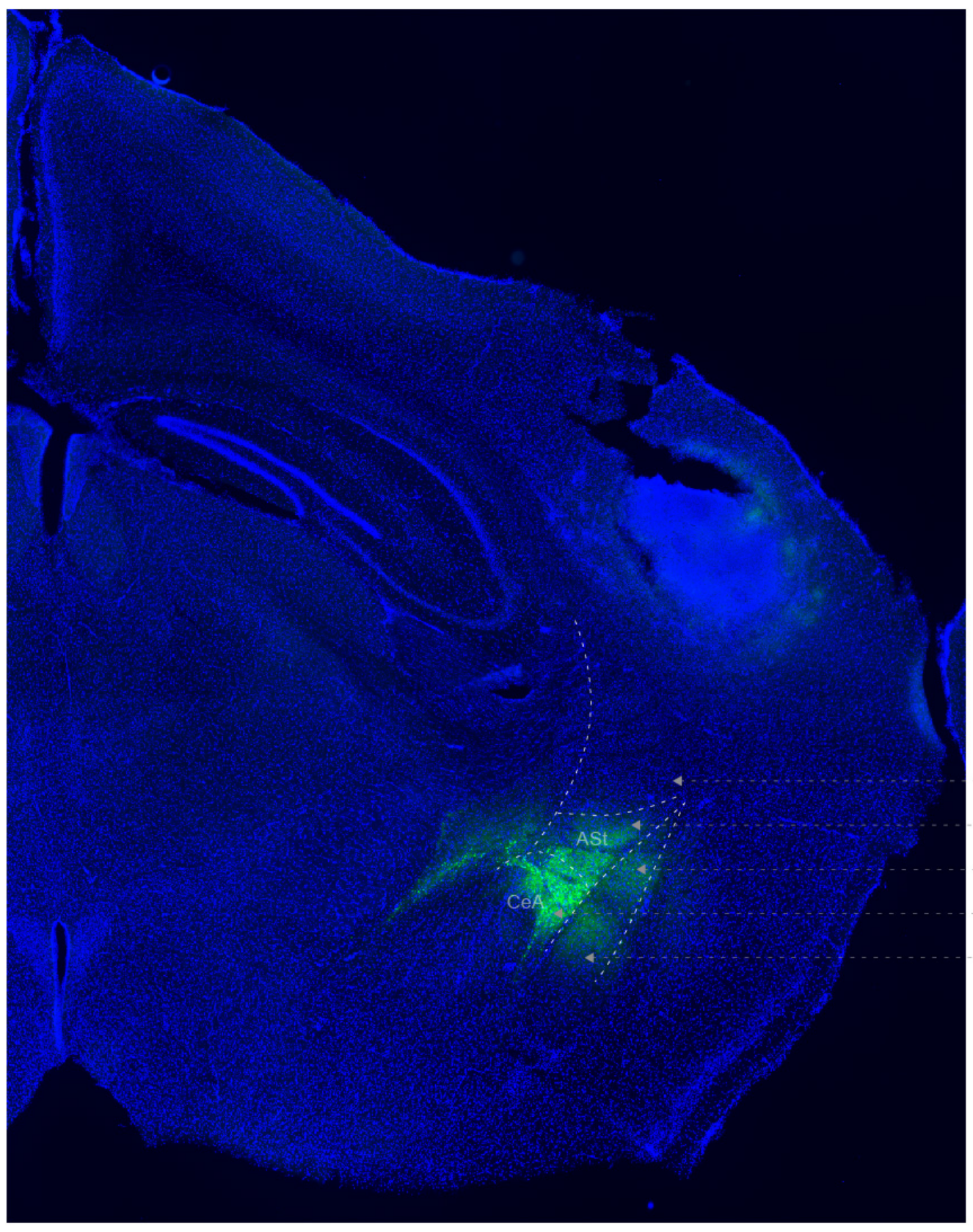

Data from this mouse were not included in the study

TS: Some expression ✓
ASt: Ok expression ✓
LA: No expression ✓
CeA: Expression ✗
BLA: No expression ✓

Drd1a+ ChR2 Cohort

"Mouse ID: D1K140L"

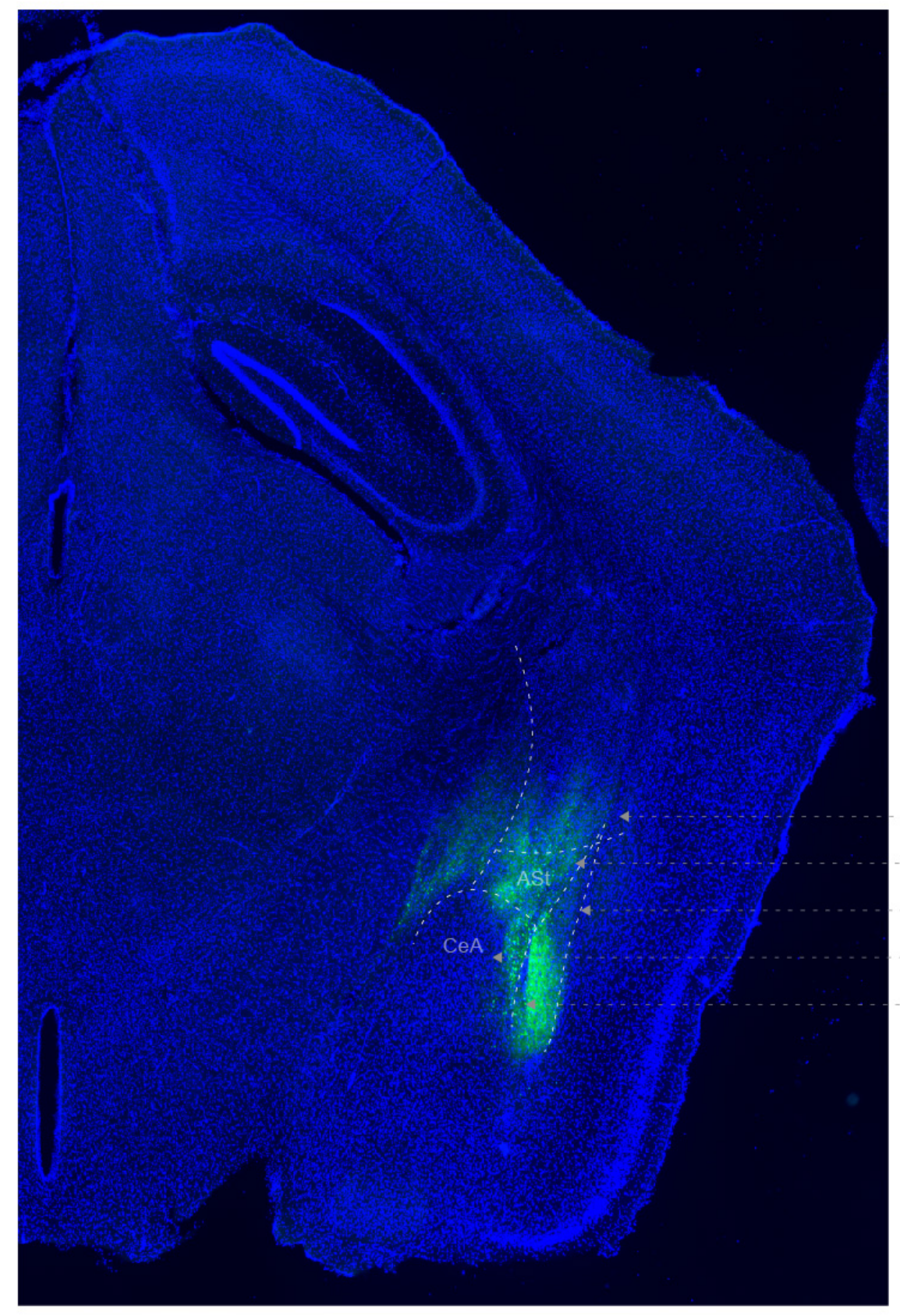

Data from this mouse were not included in the study

TS: Some expression ✓
ASt: Ok expression ✓
LA: No expression ✓
CeA: Expression ✗
BLA: No expression ✓

Appendix C - Histology - *VGAT:Cre* mice, ChR2 experiments:

Drd1a+ ChR2 Cohort

"Mouse ID: D1K087L"

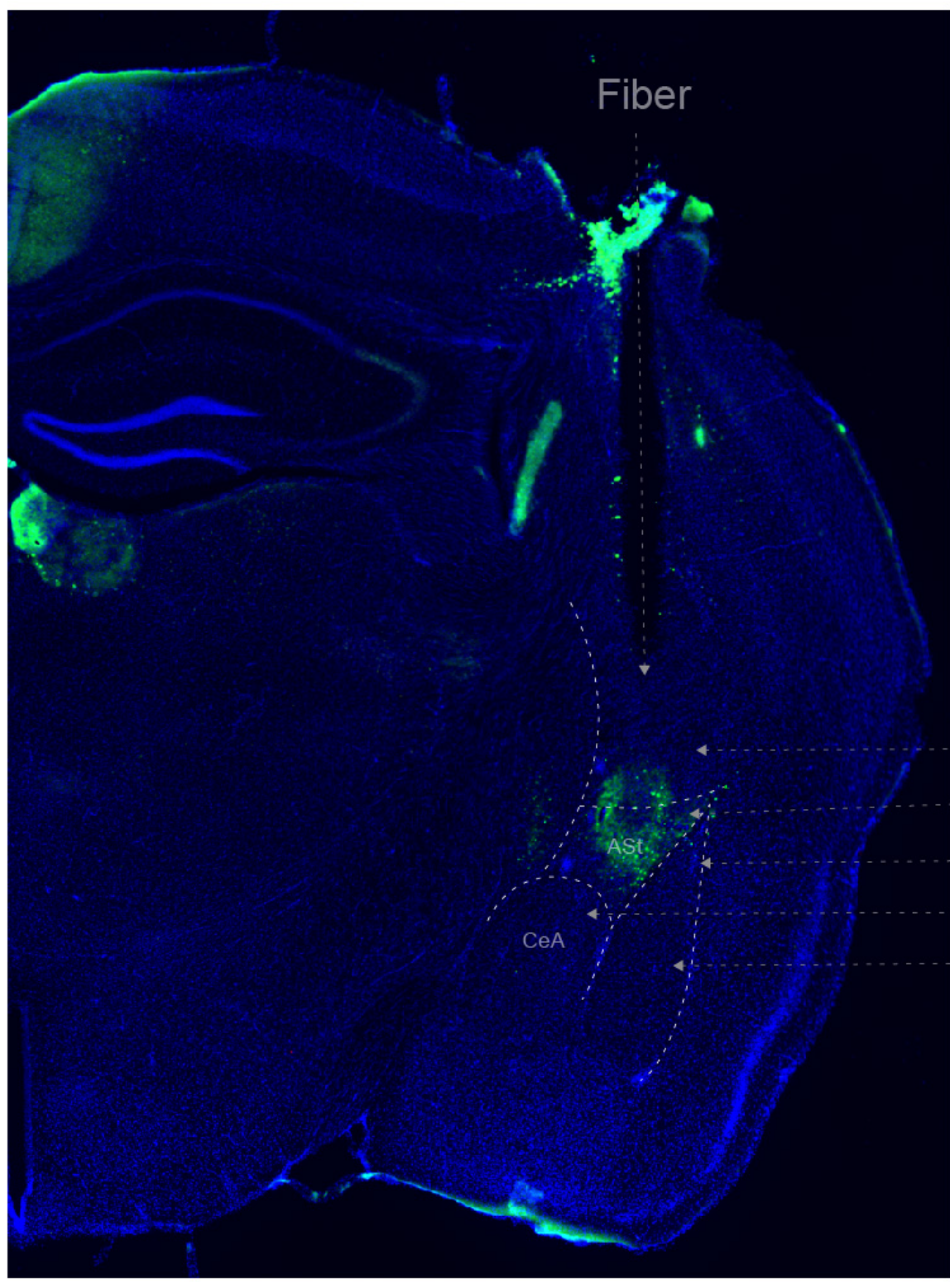

Histology okay, data included

TS: some expression ✓
ASt: Good expression ✓
LA: No expression ✓
CeA: No expression ✓
BLA: No expression ✓

Drd1a+ ChR2 Cohort

"Mouse ID: D1K087R"

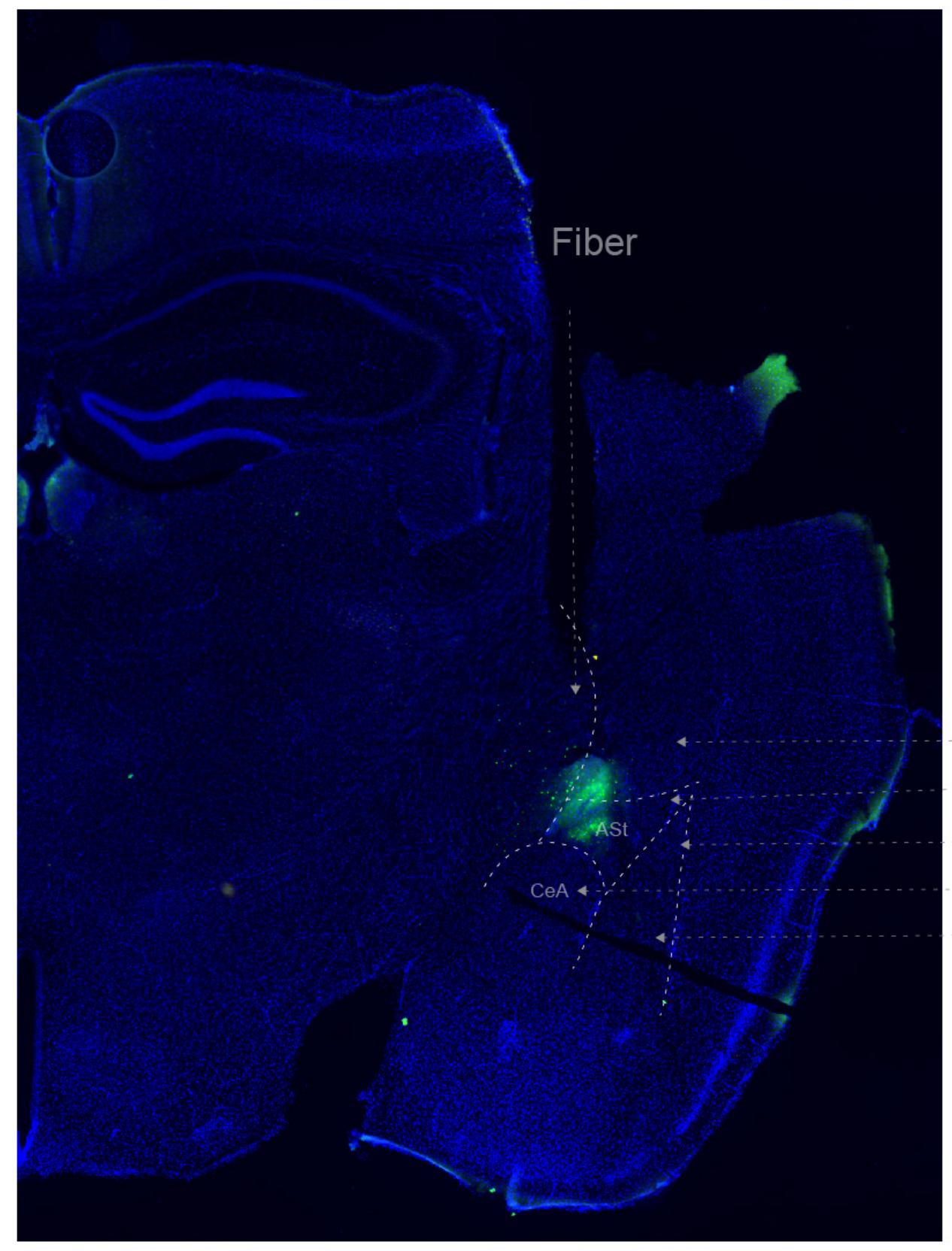

Histology okay, data included

TS: some expression ✓
ASt: Ok expression ✓
LA: No expression ✓
CeA: No expression ✓
BLA: No expression ✓

Drd1a+ ChR2 Cohort

"Mouse ID: D1K107R"

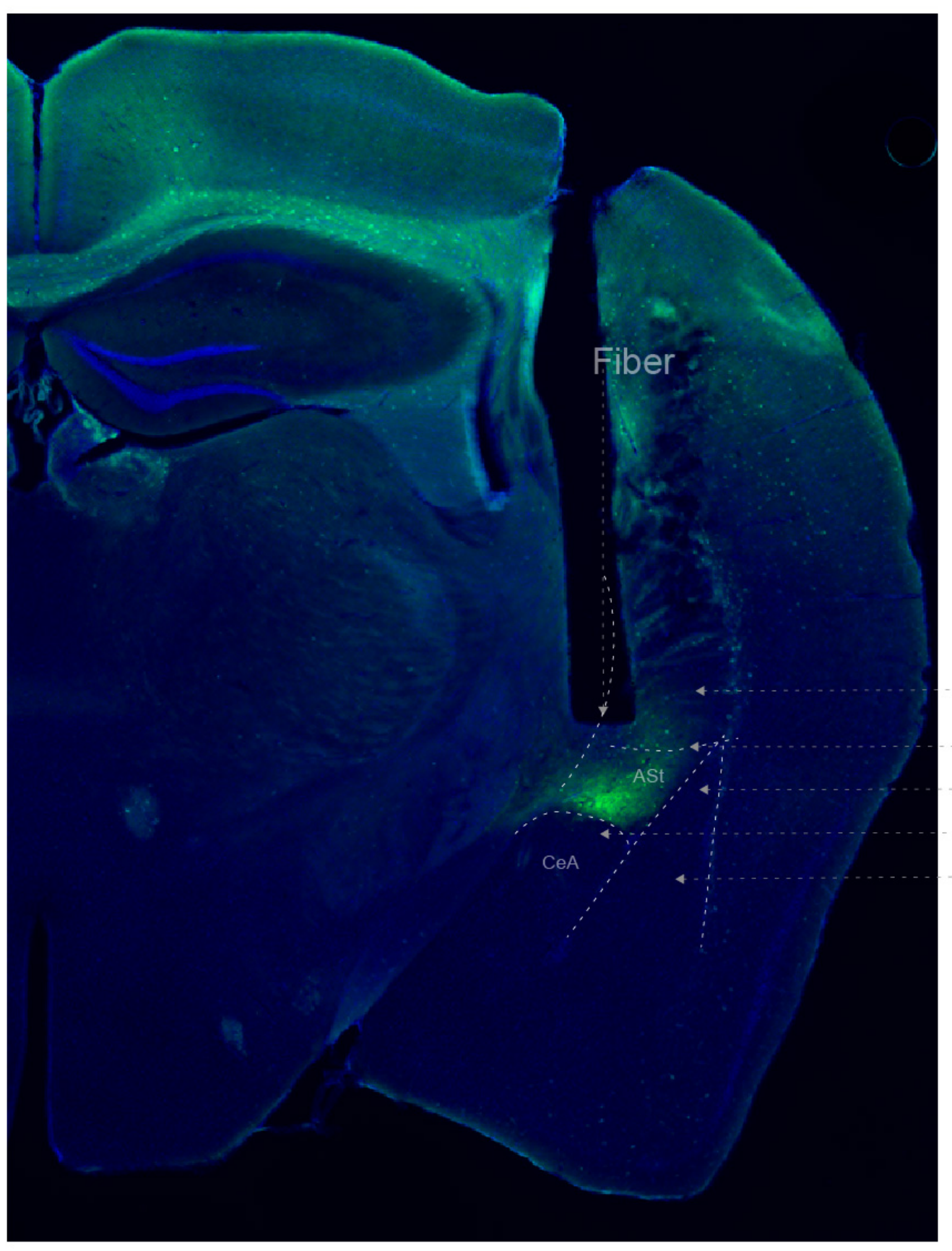

Histology okay, data included

TS: Low expression ✓
ASt: Good expression ✓
LA: No expression ✓
CeA: No expression ✓
BLA: No expression ✓

"Mouse ID: D1K157R"

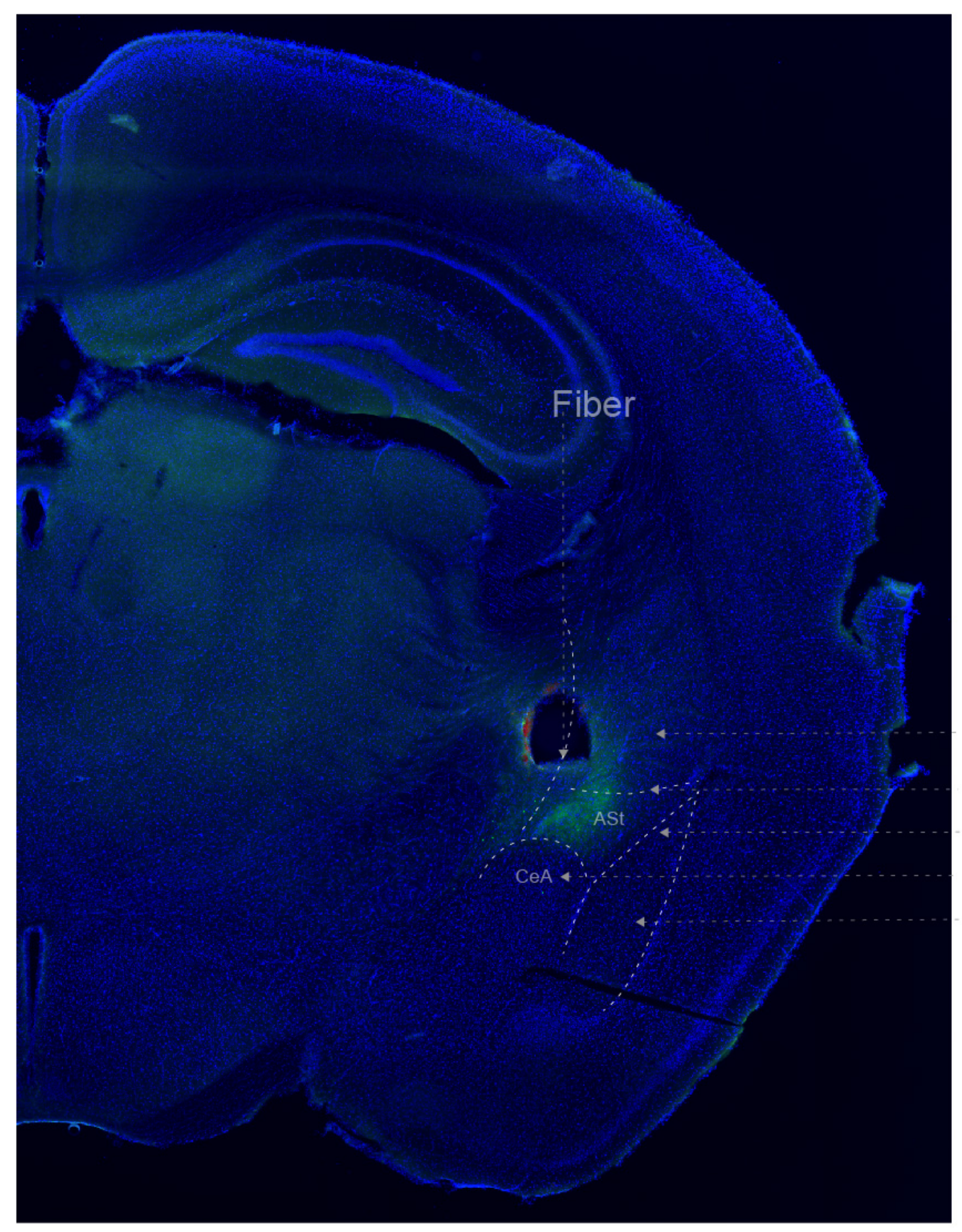

Histology okay, data included

TS: Low expression ✓
ASt: Ok expression ✓
LA: No expression ✓
CeA: No expression ✓
BLA: No expression ✓

Drd1a+ ChR2 Cohort

"Mouse ID: D1K159L"

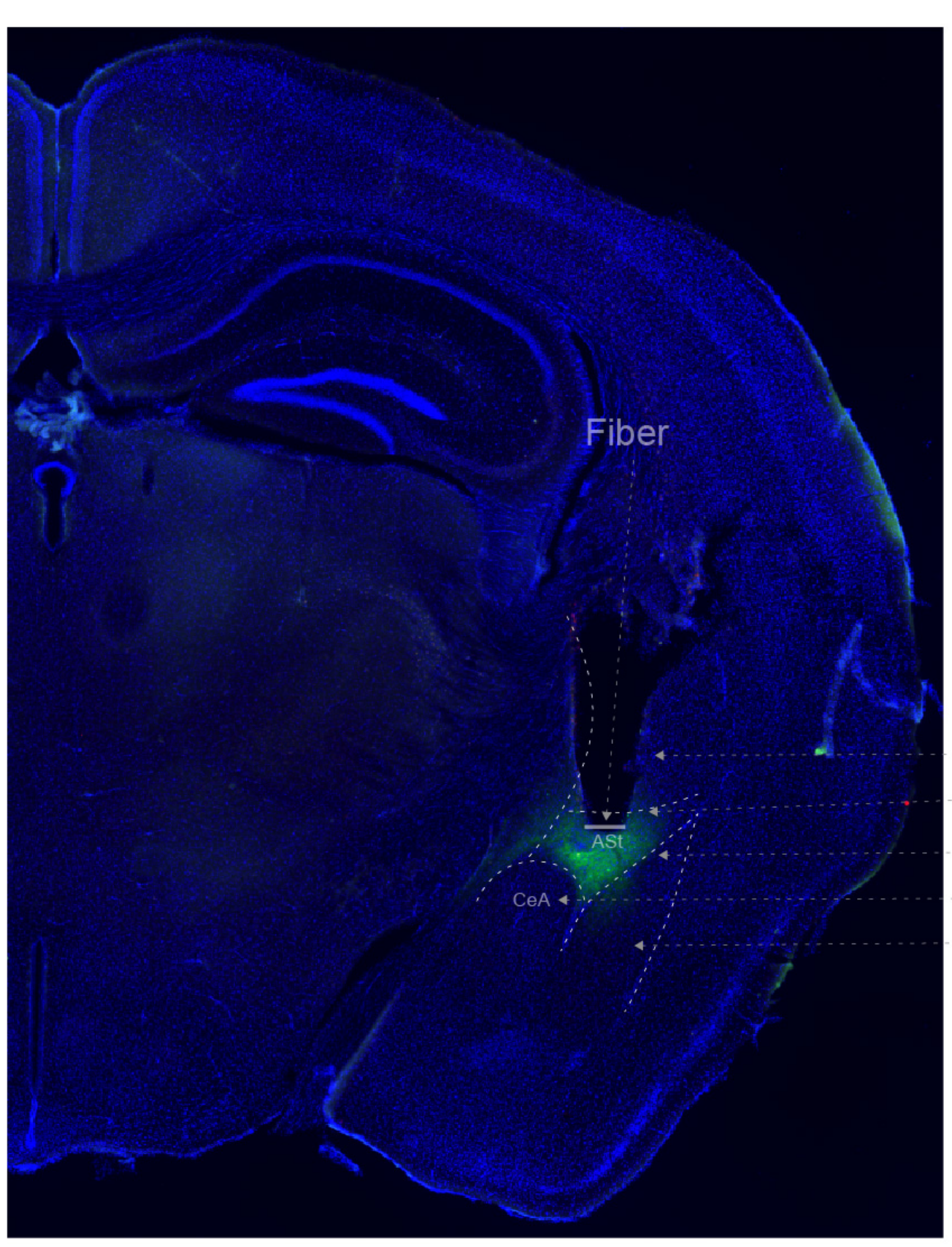

Histology okay, data included

TS: No expression ✓
ASt: Good expression ✓
LA: No expression ✓
CeA: No expression ✓
BLA: No expression ✓

Drd1a+ ChR2 Cohort

"Mouse ID: D1K133R"

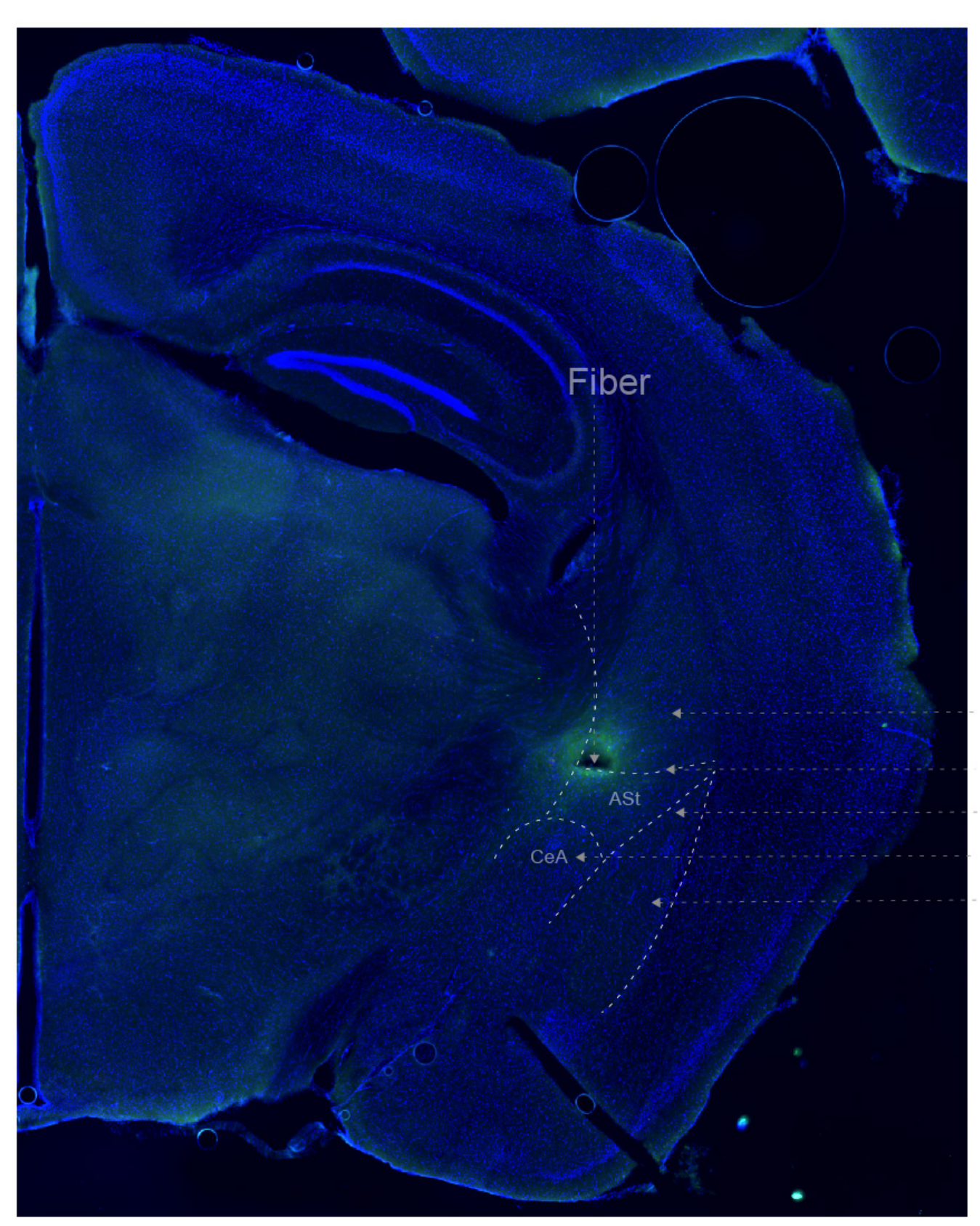

Histology okay, data included

TS: Low expression ✓
ASt: Low expression ✓
LA: No expression ✓
CeA: No expression ✓
BLA: No expression ✓

Mills et al. 2026

Data S1 - Appendix D
Histology for *Drd1a*-Cre and *Drd2*-Cre
Calcium imaging mice

Appendix D - Histology (Calcium Imaging)

Drd1a-Cre Paired Mouse ID: CACO3L_yowie

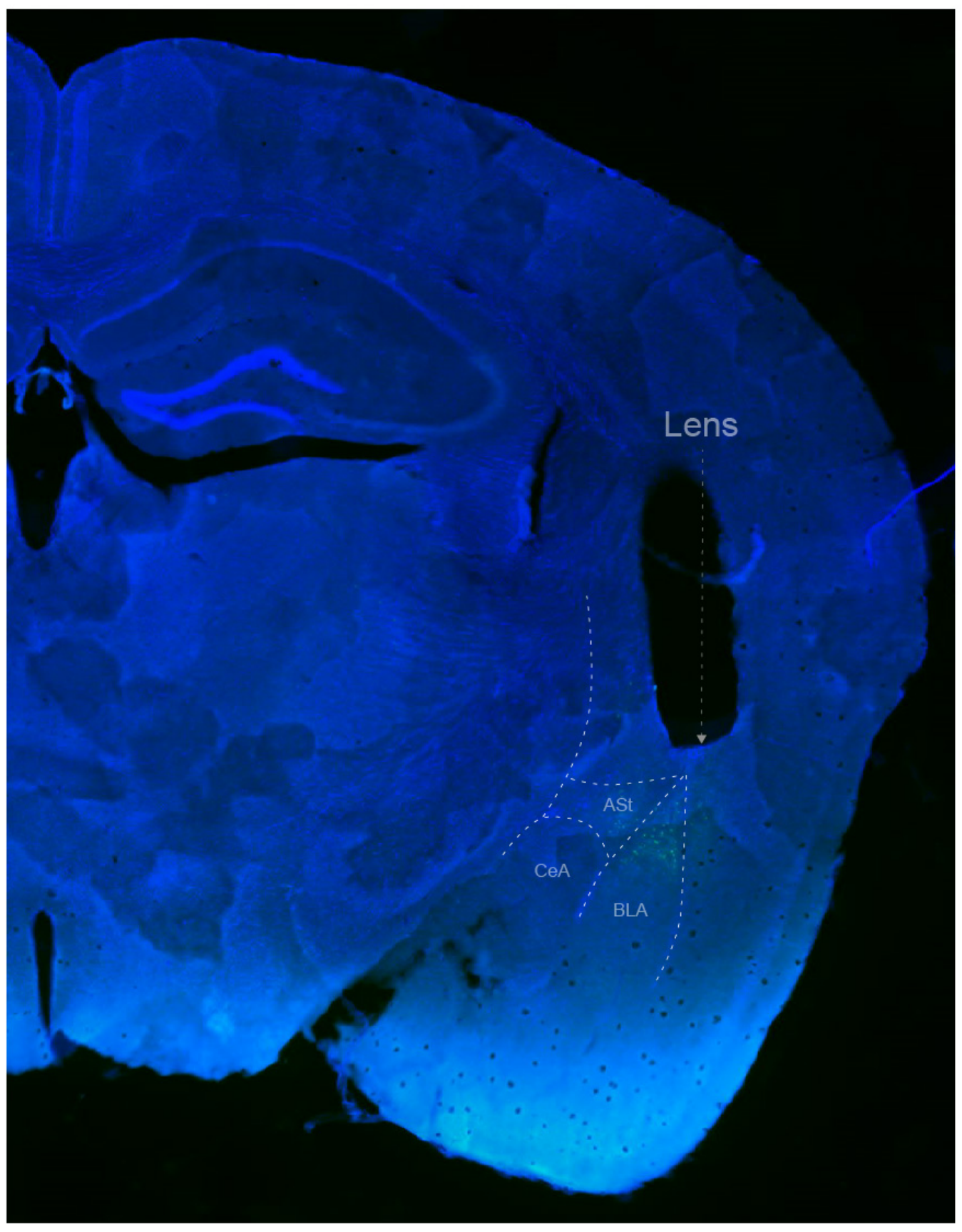

Data from this mouse were not included in the study

Lens too lateral, cells in field of view are in cortex ✘

Green = GCaMP7f
Blue = DAPI

Drd1a-Cre Paired Mouse ID: CACO3_0000_notag

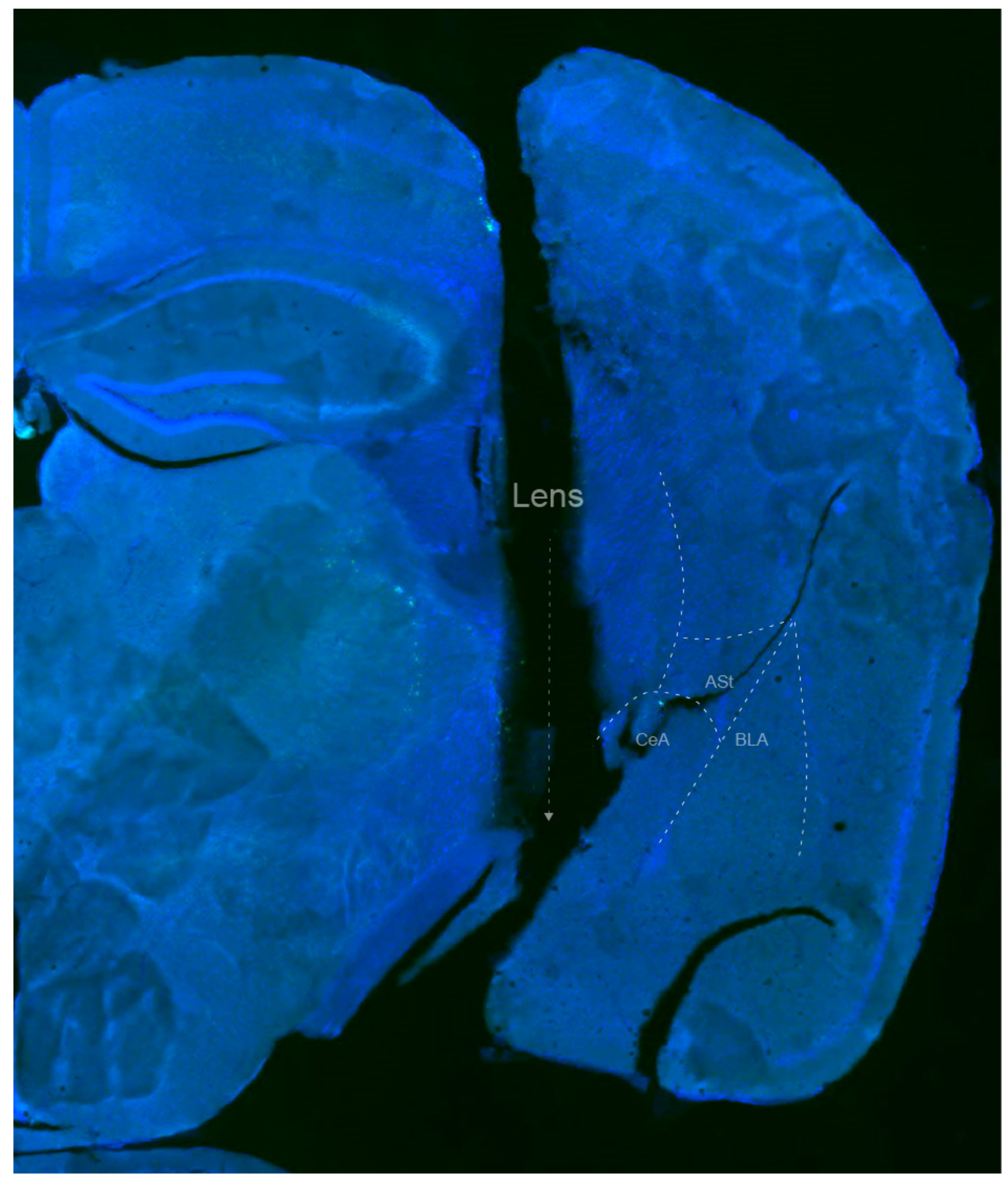

Data from this mouse were not included in the study

Lens too medial, field of view far from ASt ✘

Green = GCaMP7f
Blue = DAPI

Drd1a-Cre Paired Mouse ID: CACO5_K012

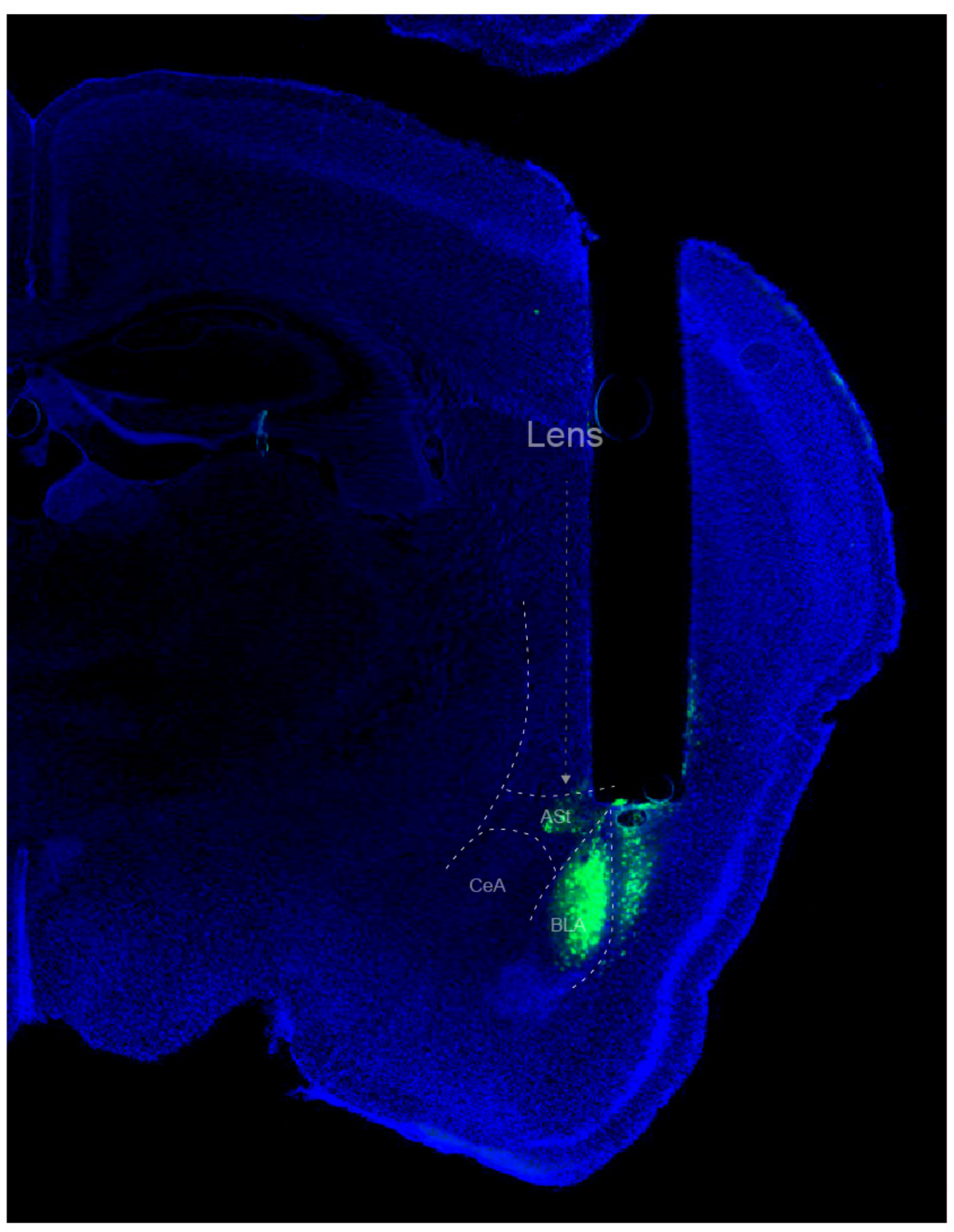

Data from this mouse were not included in the study

Lens too lateral, cells imaged are in cortex ✘

Green = GCaMP7f
Blue = DAPI

Drd1a-Cre Unpaired Mouse ID: CACO3_6862_L wendigo

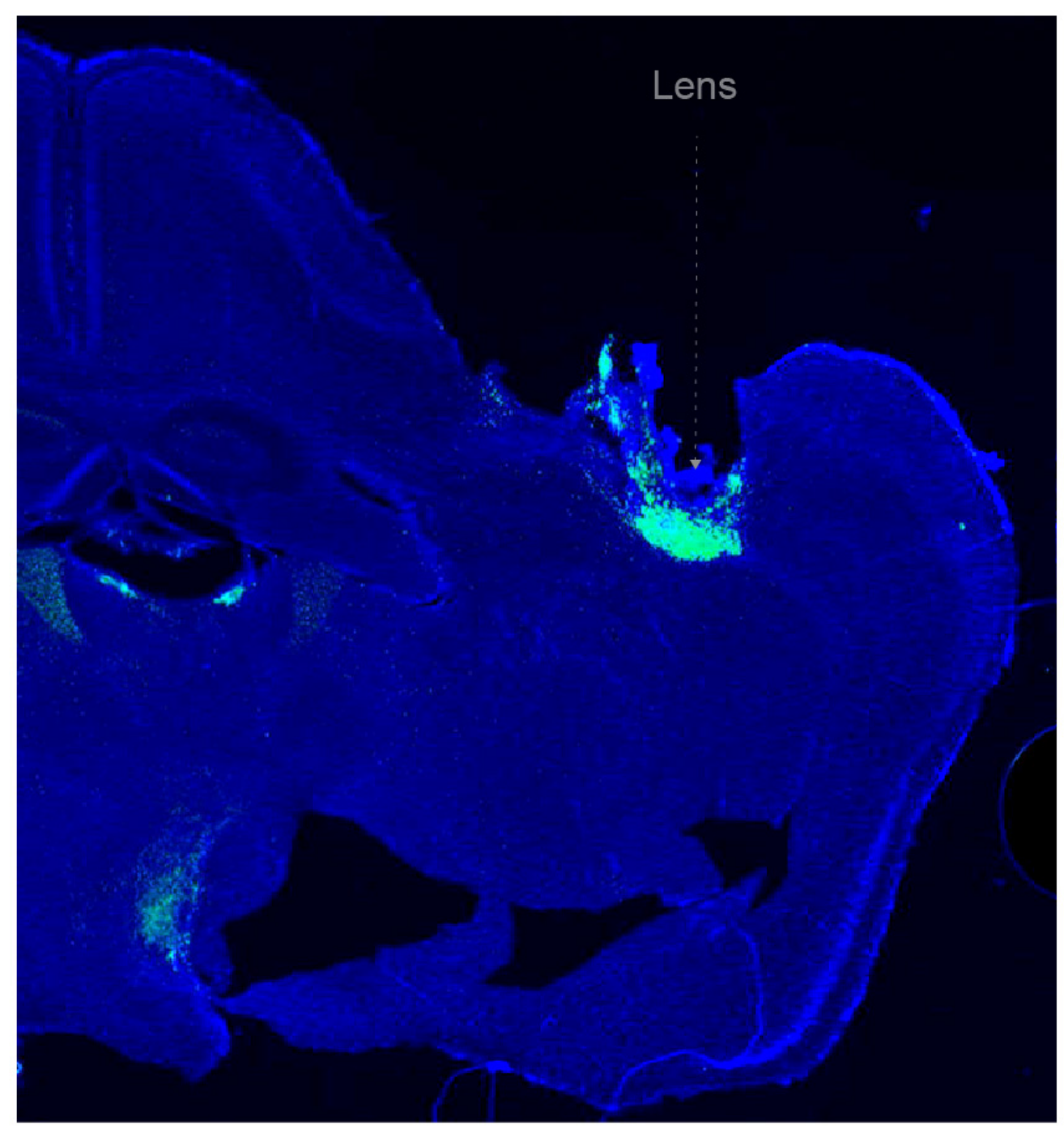

Data from this mouse were not included in the study

Brain skull deforma-tion distorted brain, lens and virus far lateral of ASt ✘

Green = GCaMP7f
Blue = DAPI

Drd1a-Cre Unpaired Mouse ID: CACO3_6887_R_phil

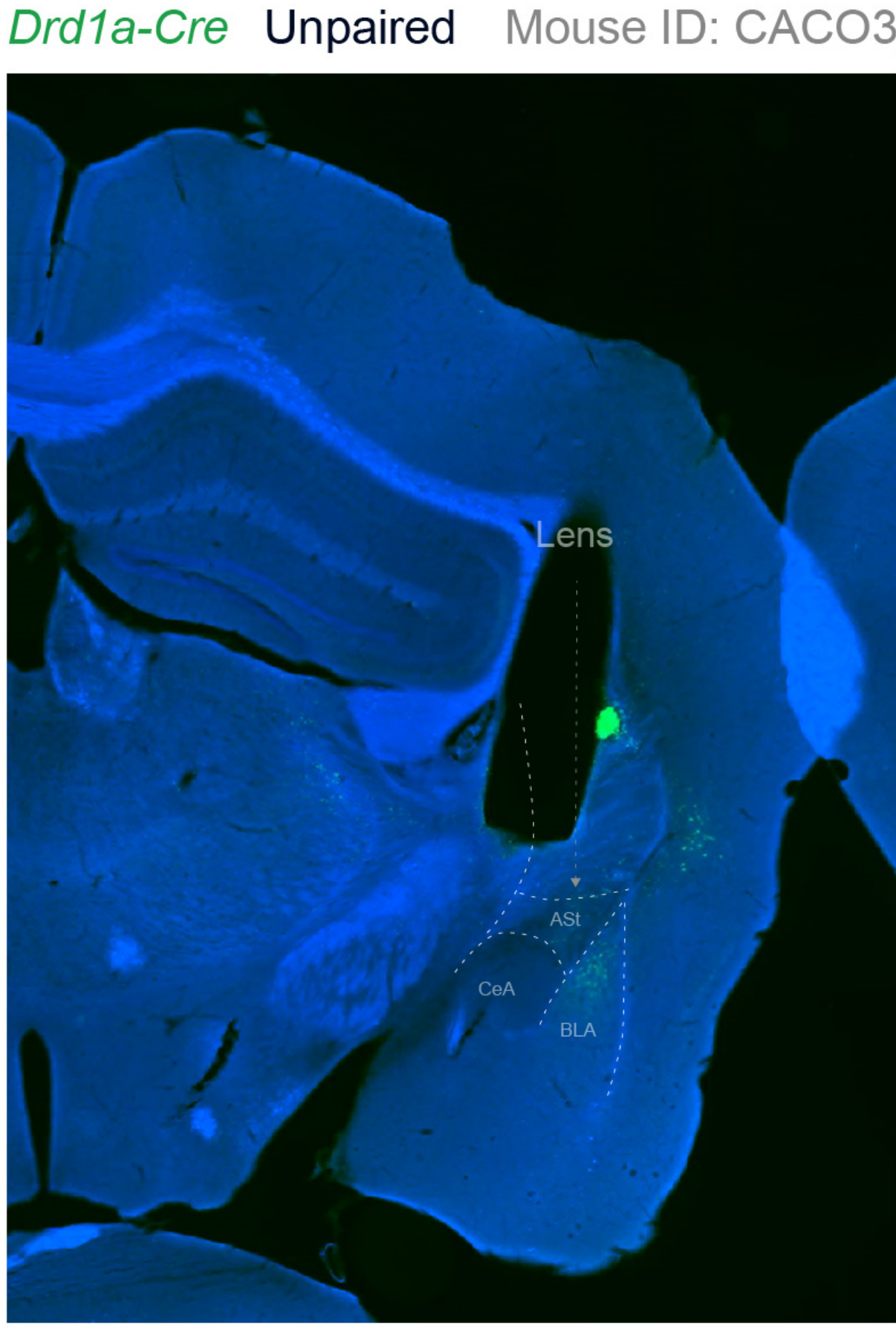

Data from this mouse were not included in the study

Lens too medial, angle of lens indicates any cells imaged are in GPe ✘

Green = GCaMP7f
Blue = DAPI

Drd1a-Cre Unpaired Mouse ID: CACO3_6861_R_guy

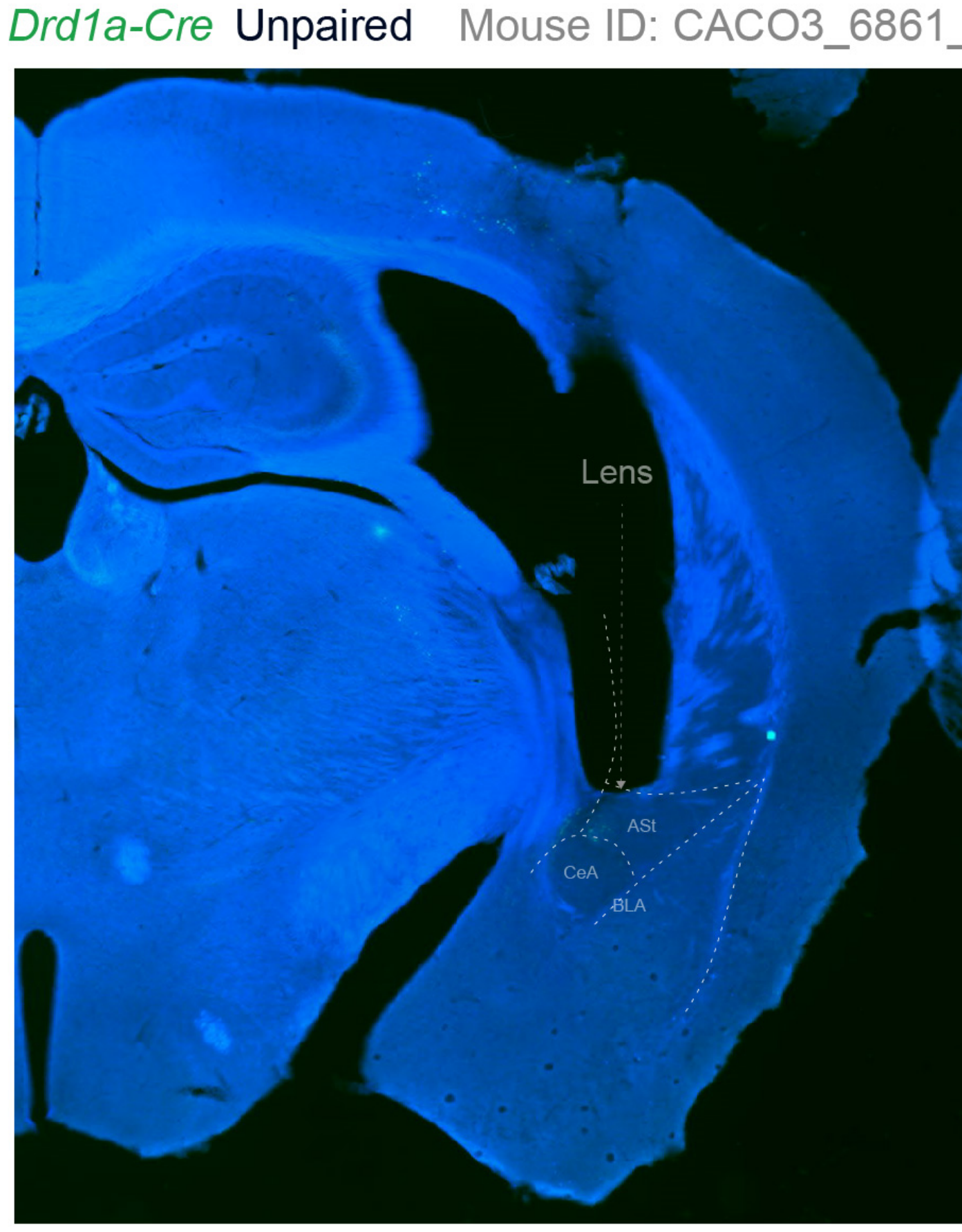

Data from this mouse were not included in the study

Lens close to CeA ✘

Green = GCaMP7f
Blue = DAPI

Appendix D - Histology (Calcium Imaging)

Drd1a-Cre Paired Mouse ID: CACO3_6868_L_quetzal Data included

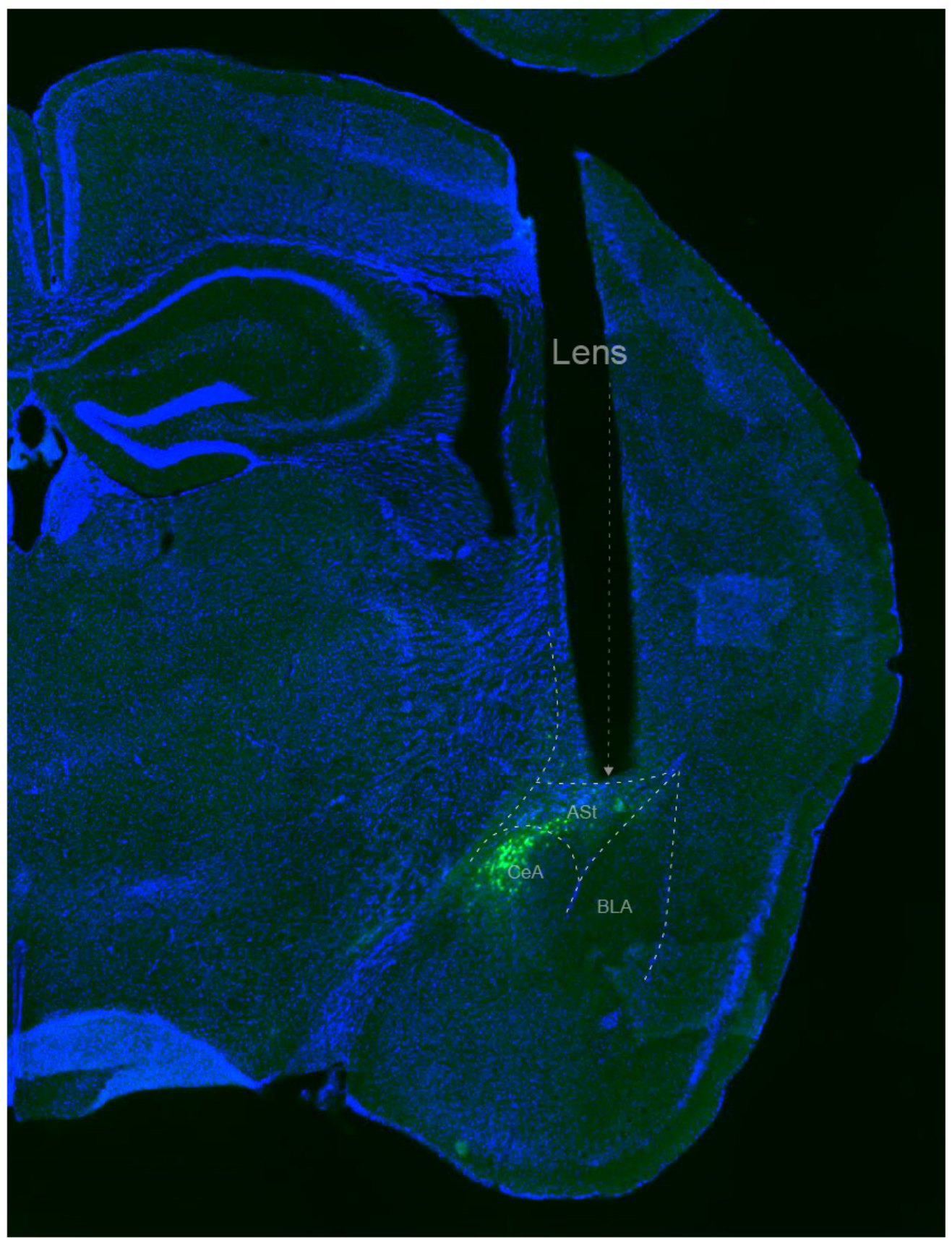

Great lens placement + some viral spread to CeA but well outside of imaging field ✓

Green = GCaMP7f
Blue = DAPI

Drd1a-Cre Paired Mouse ID: CACO3_6933_R_chupacabra Data included

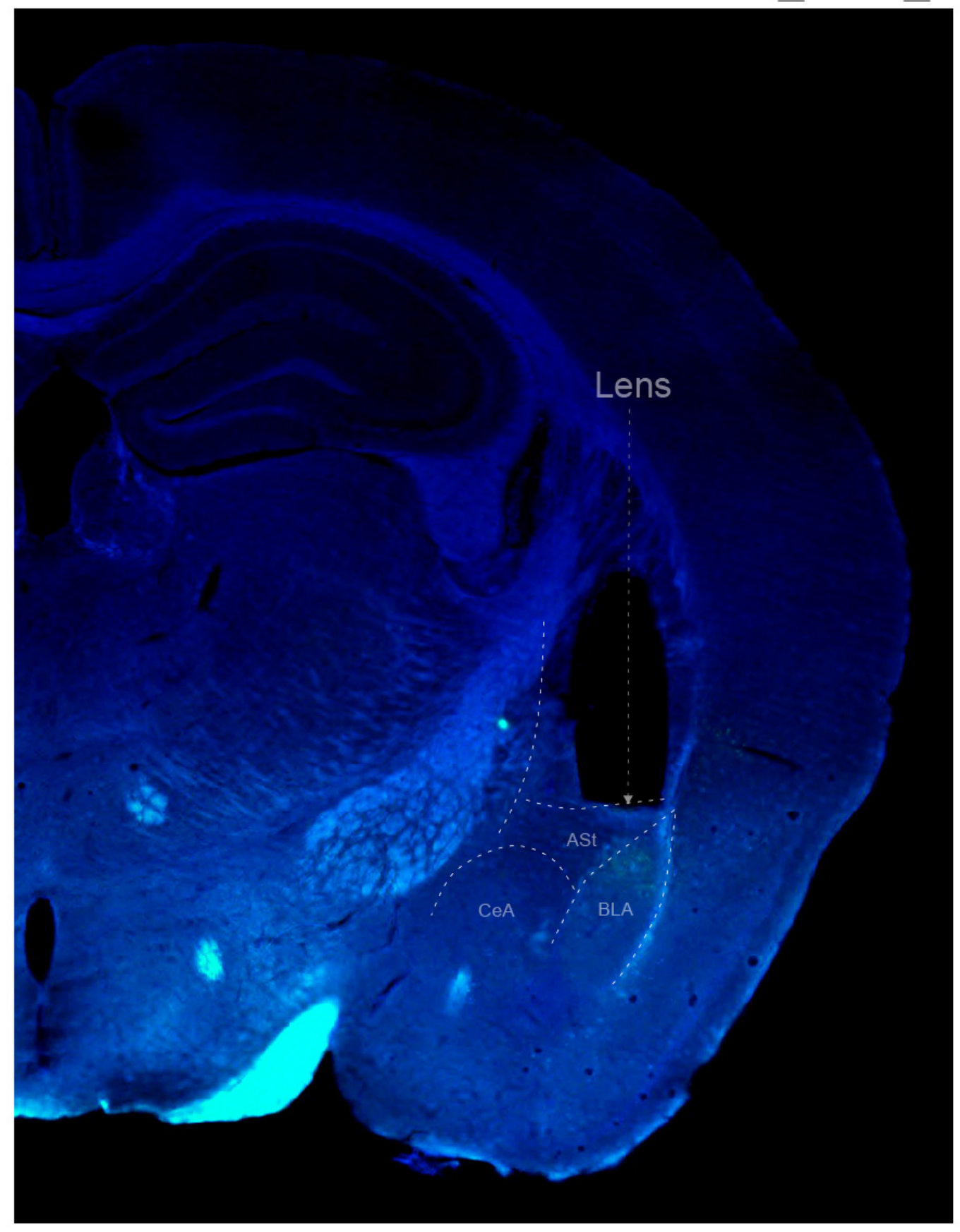

Good lens targeting to ASt + viral expression ✓

Green = GCaMP7f
Blue = DAPI

Drd1a-Cre Paired Mouse ID: CACO3_6882_L_kevin Data included

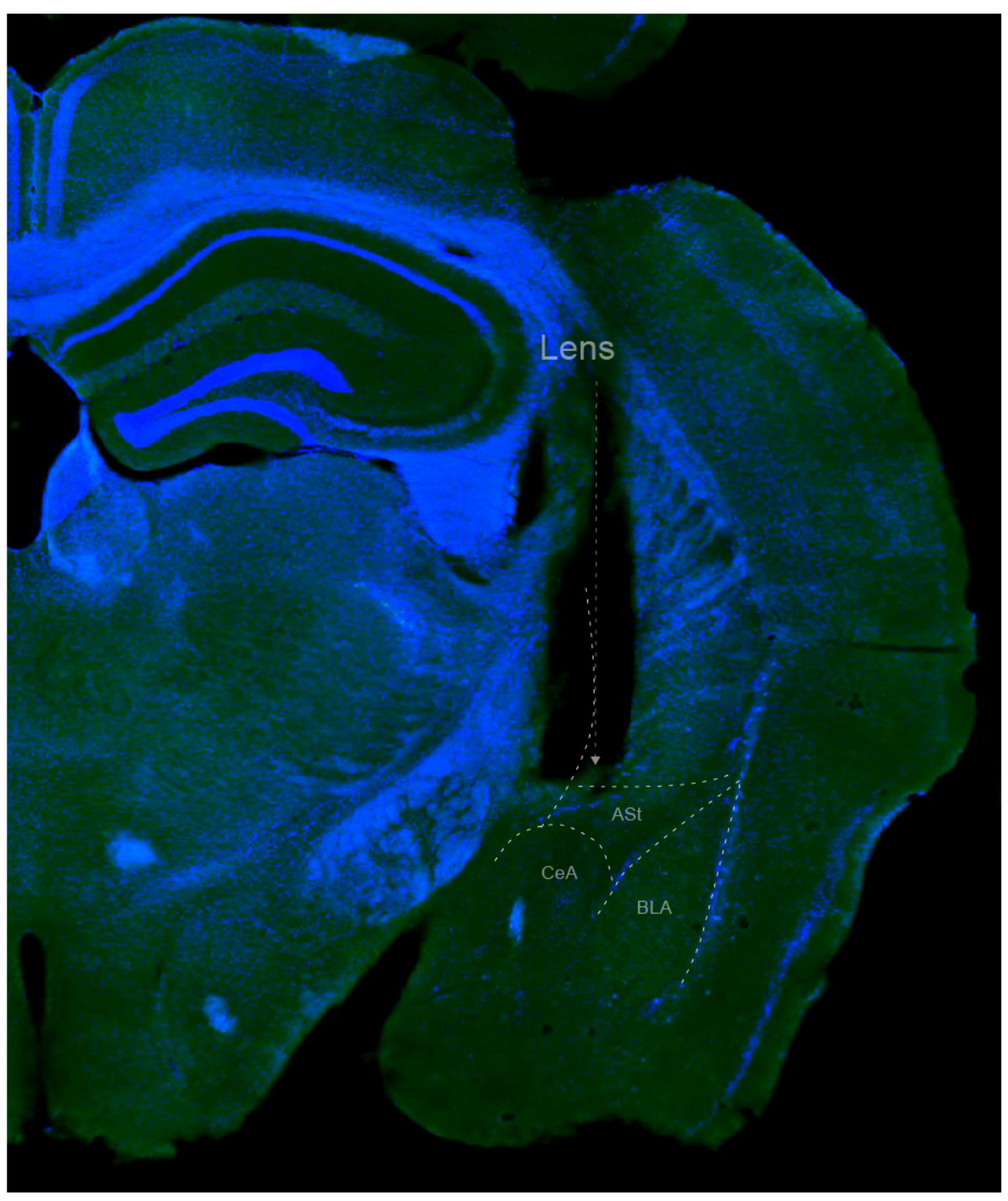

Slightly medial, low virus expression, but imaging field of view should be in ASt ✓

Green = GCaMP7f
Blue = DAPI

Drd1a-Cre Paired Mouse ID: CACO3_6921_R_nessie Data included

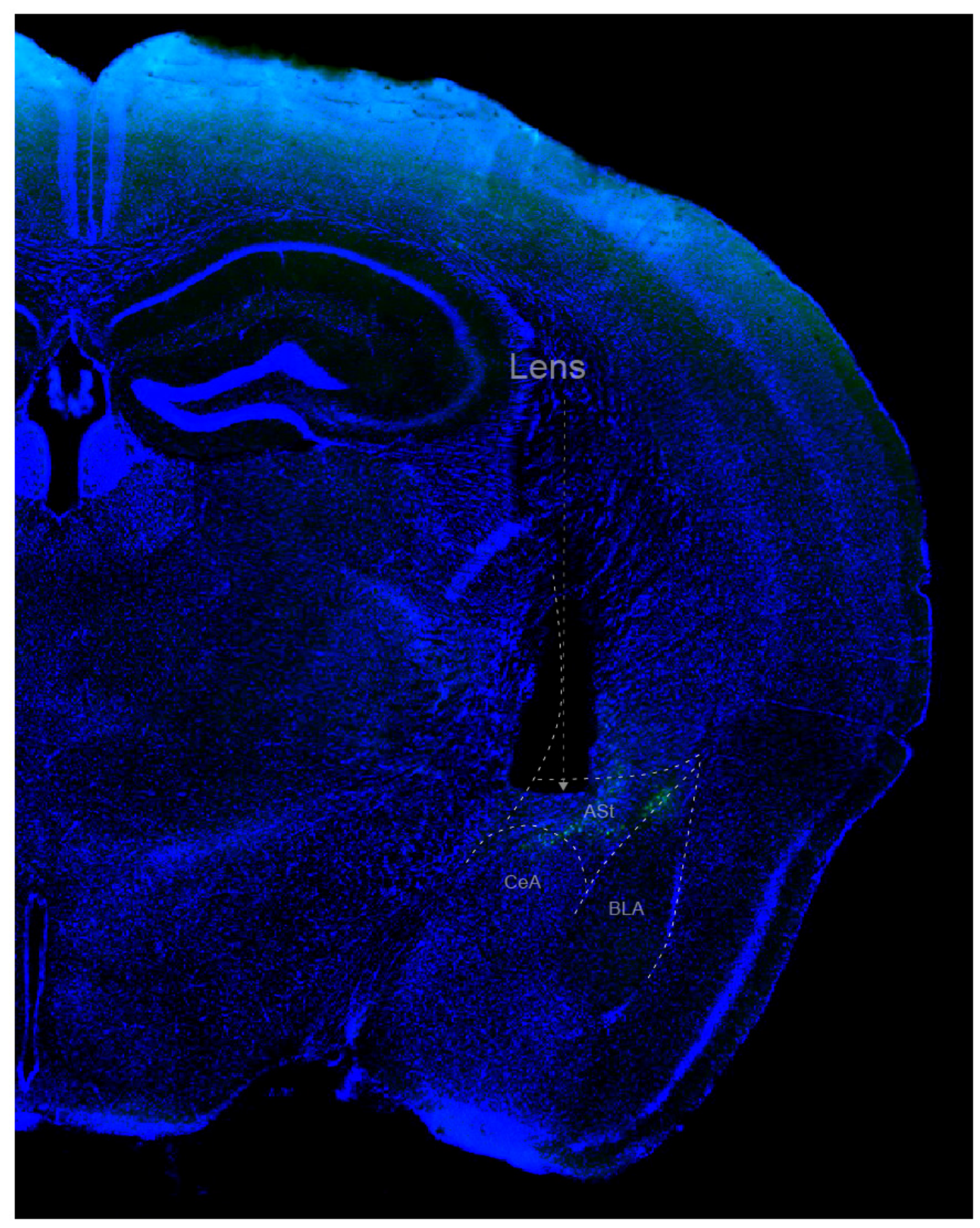

Slightly medial lens, expression in ASt ✓

Green = GCaMP7f
Blue = DAPI

Drd1a-Cre Paired Mouse ID: CACO3_6865_R_frogman Data included

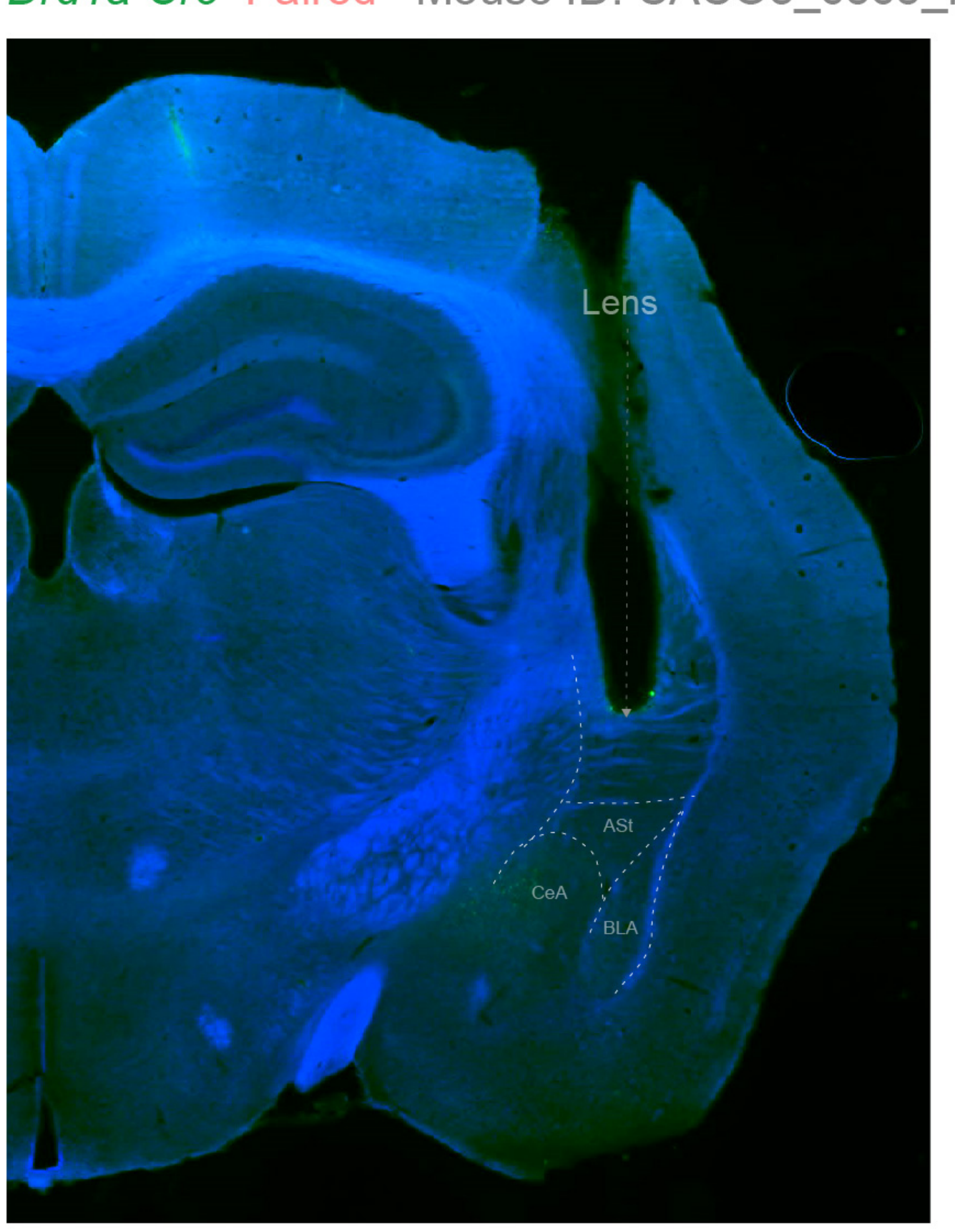

Lens placement a bit dorsal, borderline ✓

Green = GCaMP7f
Blue = DAPI

Drd1a-Cre Paired Mouse ID: CACO3_6915_R_mothman Data included

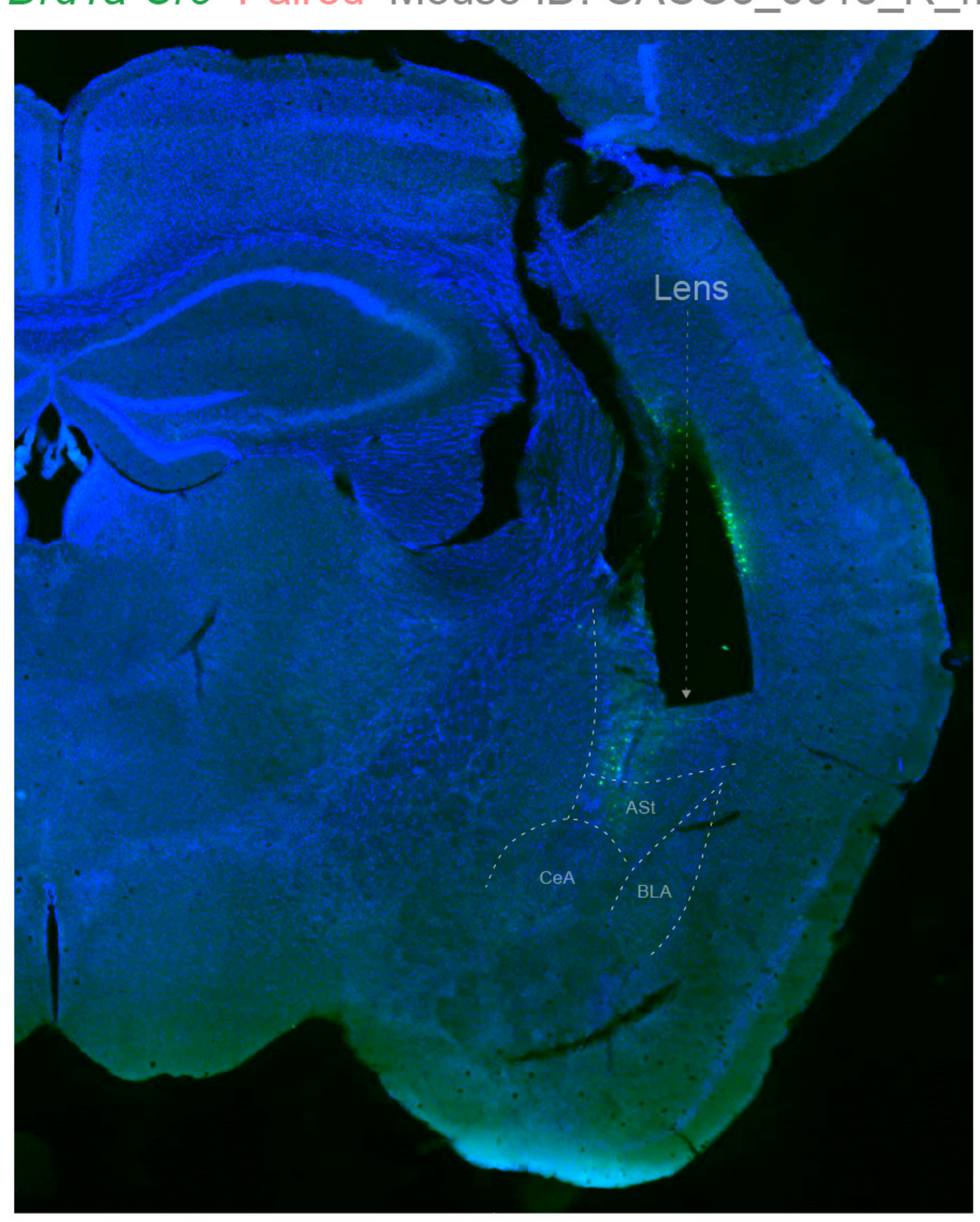

Lens dorsal to ASt, ok expression in ASt ✓

Green = GCaMP7f
Blue = DAPI

Appendix D - Histology (Calcium Imaging)

Drd1a-Cre Paired Mouse ID: CACO5_K033_L Data included

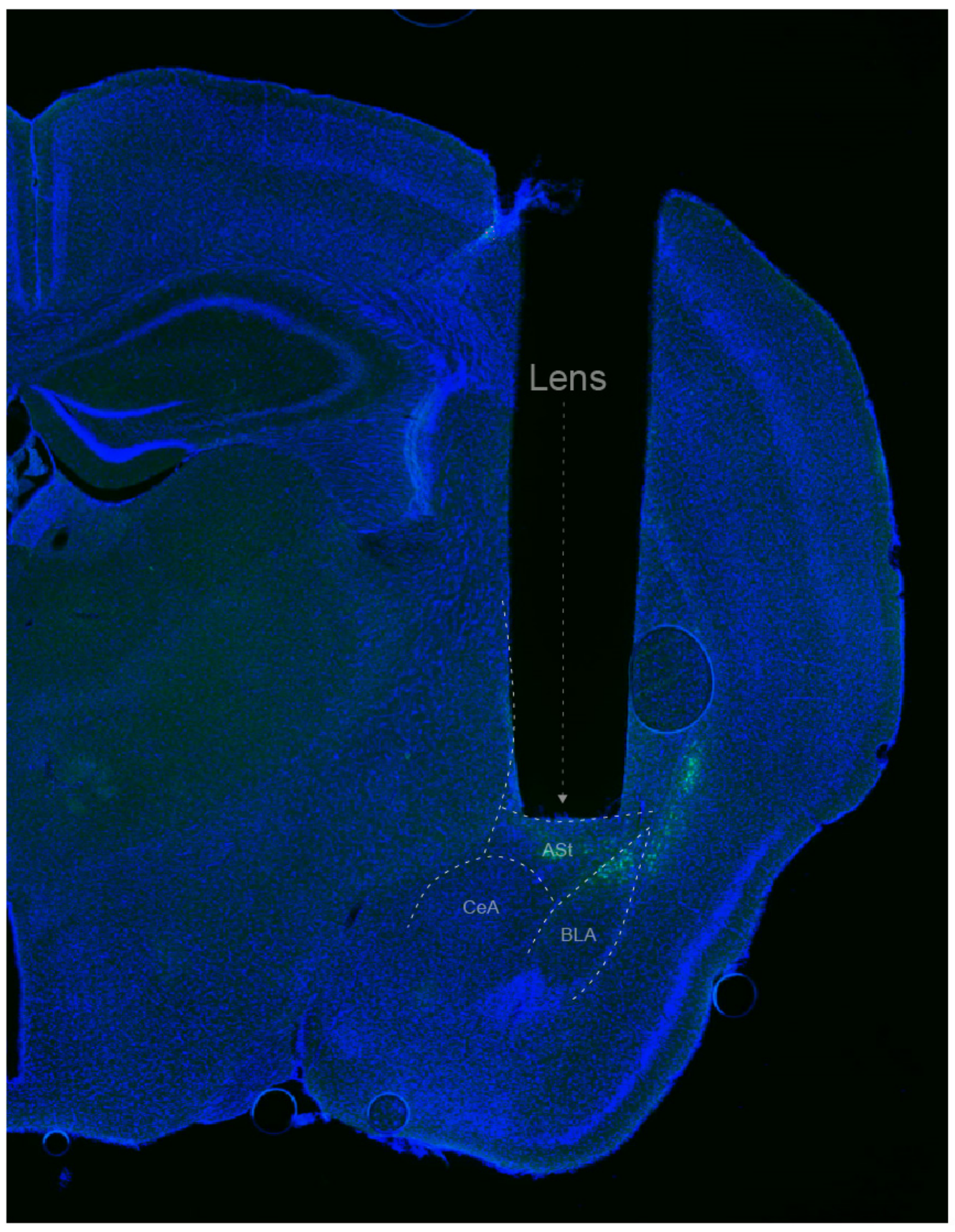

Great lens placement over ASt and virus expression in ASt. Some viral spread but outside of imaging area under lens. ✓

Green = GCaMP7f
Blue = DAPI

Drd1a-Cre Paired Mouse ID: CACO5_K035 Data included

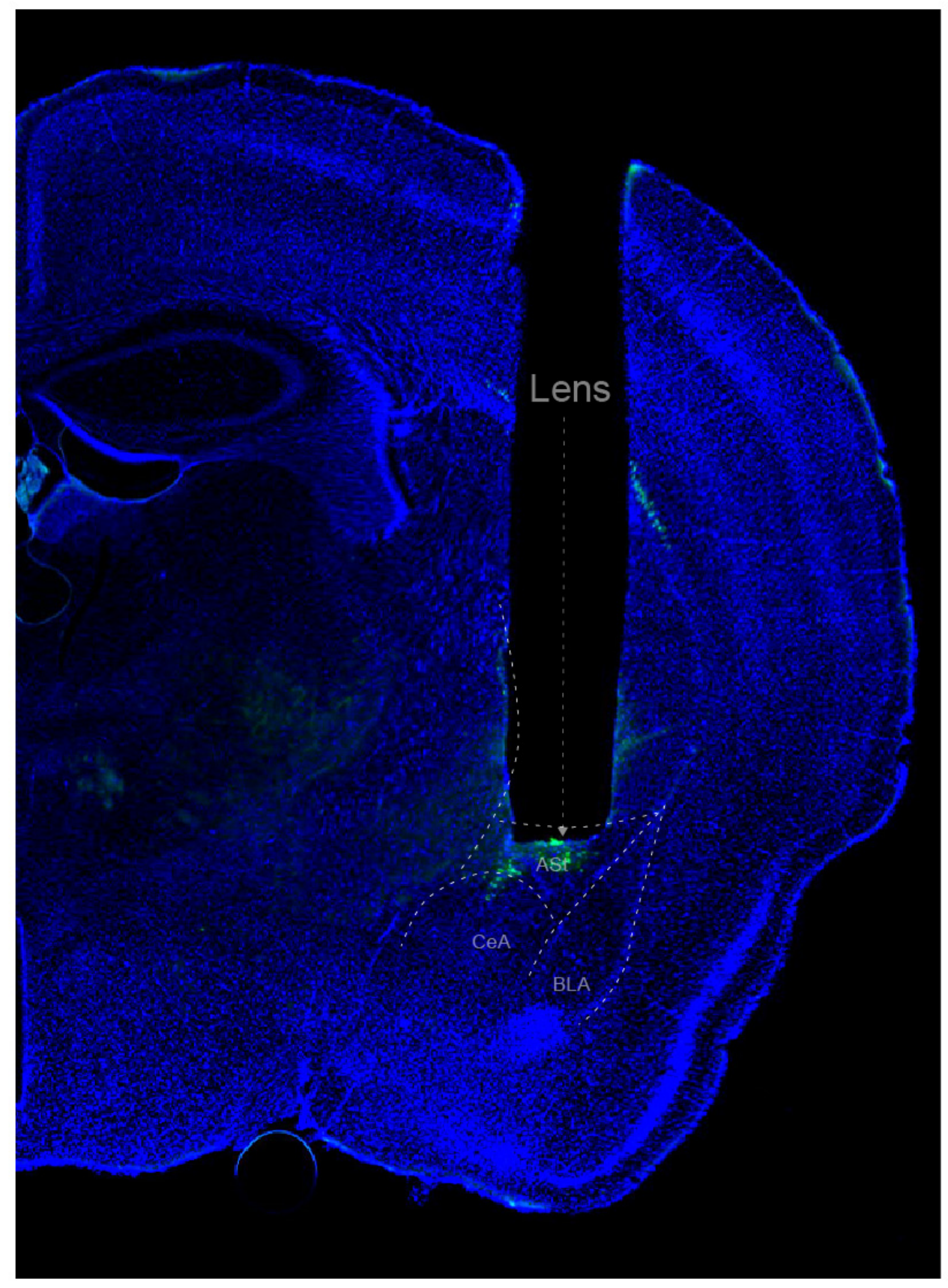

Great lens placement over ASt and virus expression in ASt ✓

Green = GCaMP7f
Blue = DAPI

Drd1a-Cre Paired Mouse ID: CACO5_K047 Data included

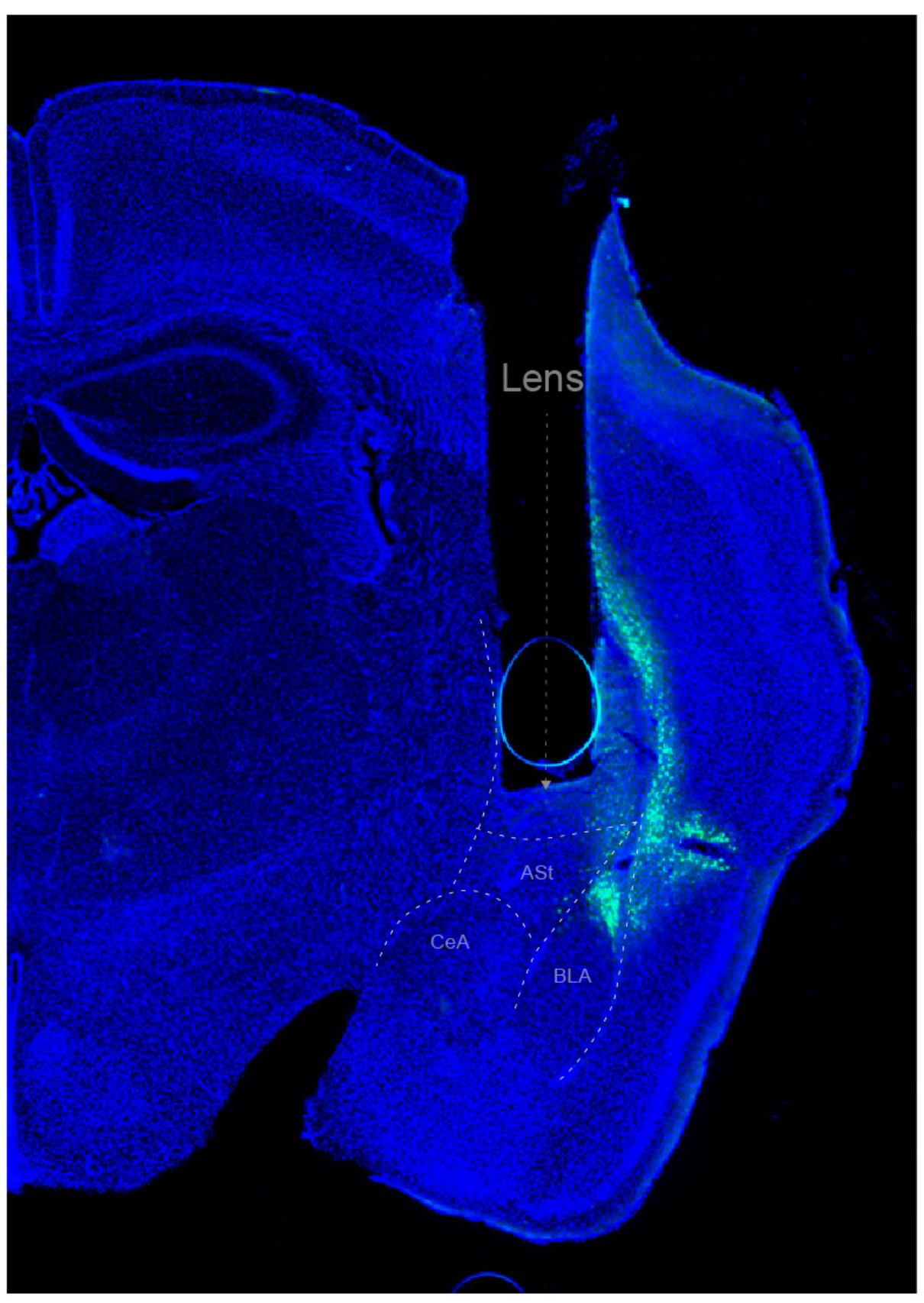

Lens placement slightly dorsal, but cells still in ASt. Some viral spread but outside of imaging area. ✓

Green = GCaMP7f
Blue = DAPI

Drd1a-Cre Paired Mouse ID: CACO5+_K008 Data included

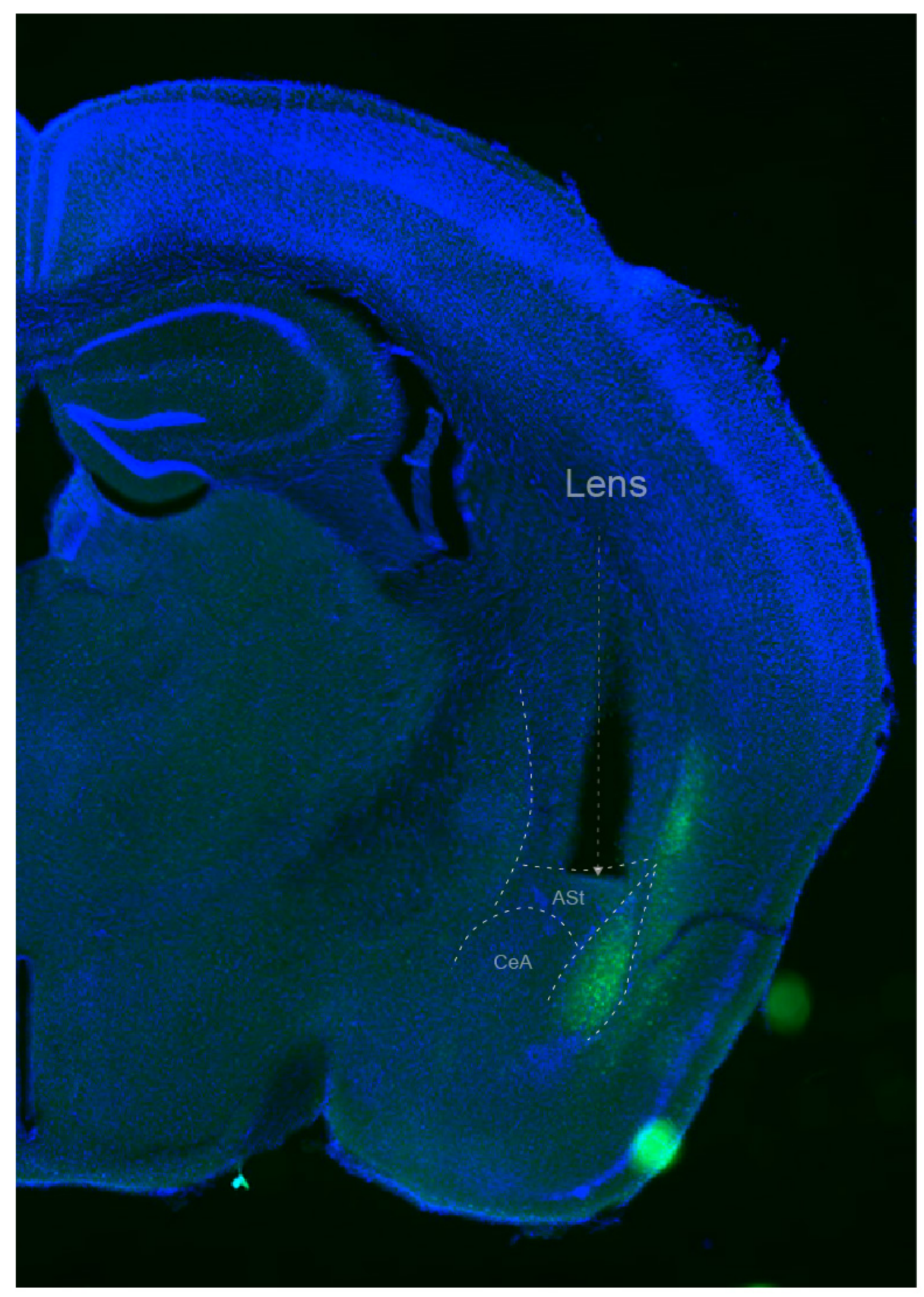

Good lens placement, virus spread to BLA but lens location restricts imaging to AST ✓

Green = GCaMP7f
Blue = DAPI

Appendix D - Histology (Calcium Imaging)

Drd1a-Cre Unpaired Mouse ID: CACO3_6886_L_jackalope Data included

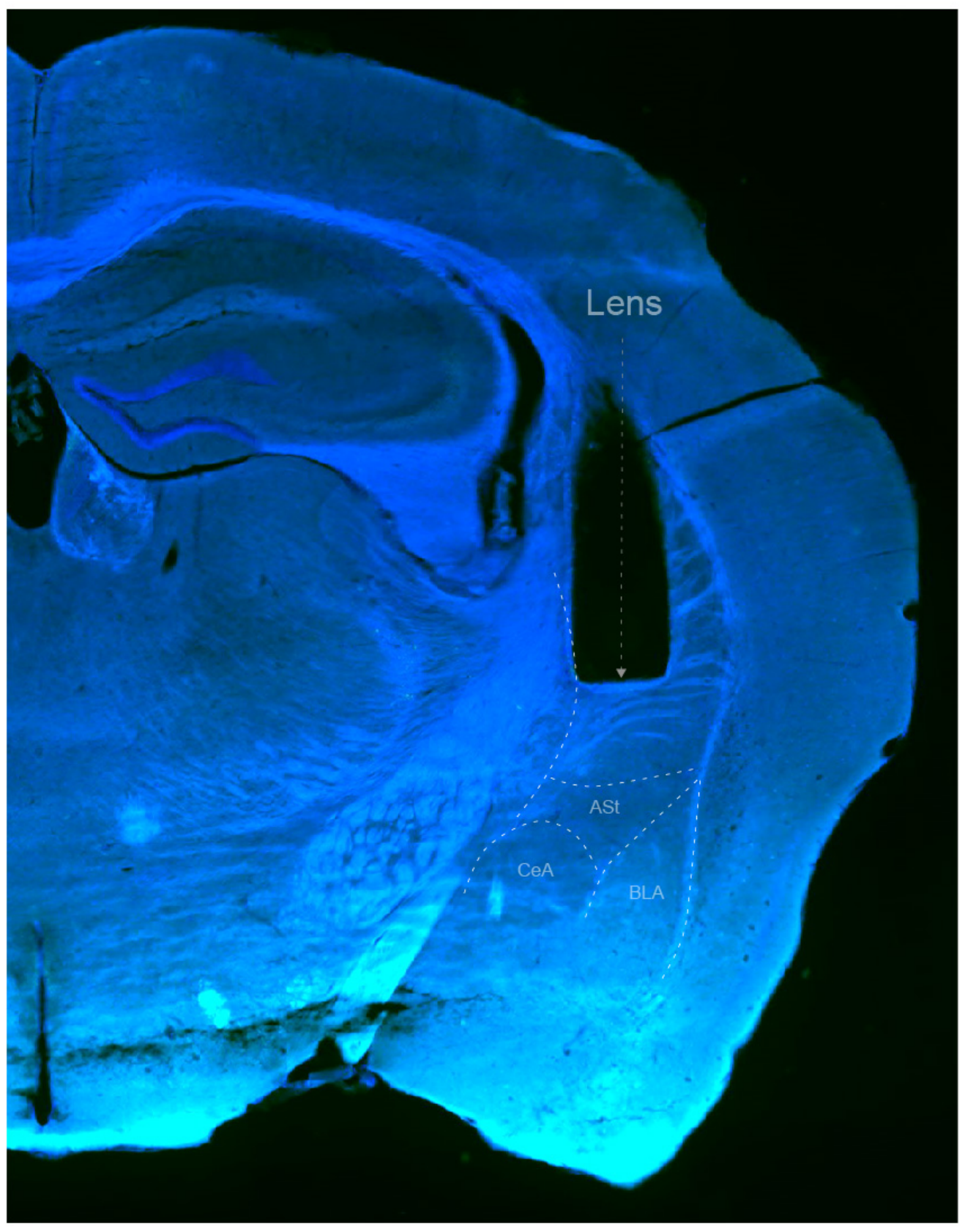

Lens slightly dorsal, field of view towards ASt ✓

Green = GCaMP7f
Blue = DAPI

Drd1a-Cre Unpaired Mouse ID: CACO3_6885_R_cyclops Data included

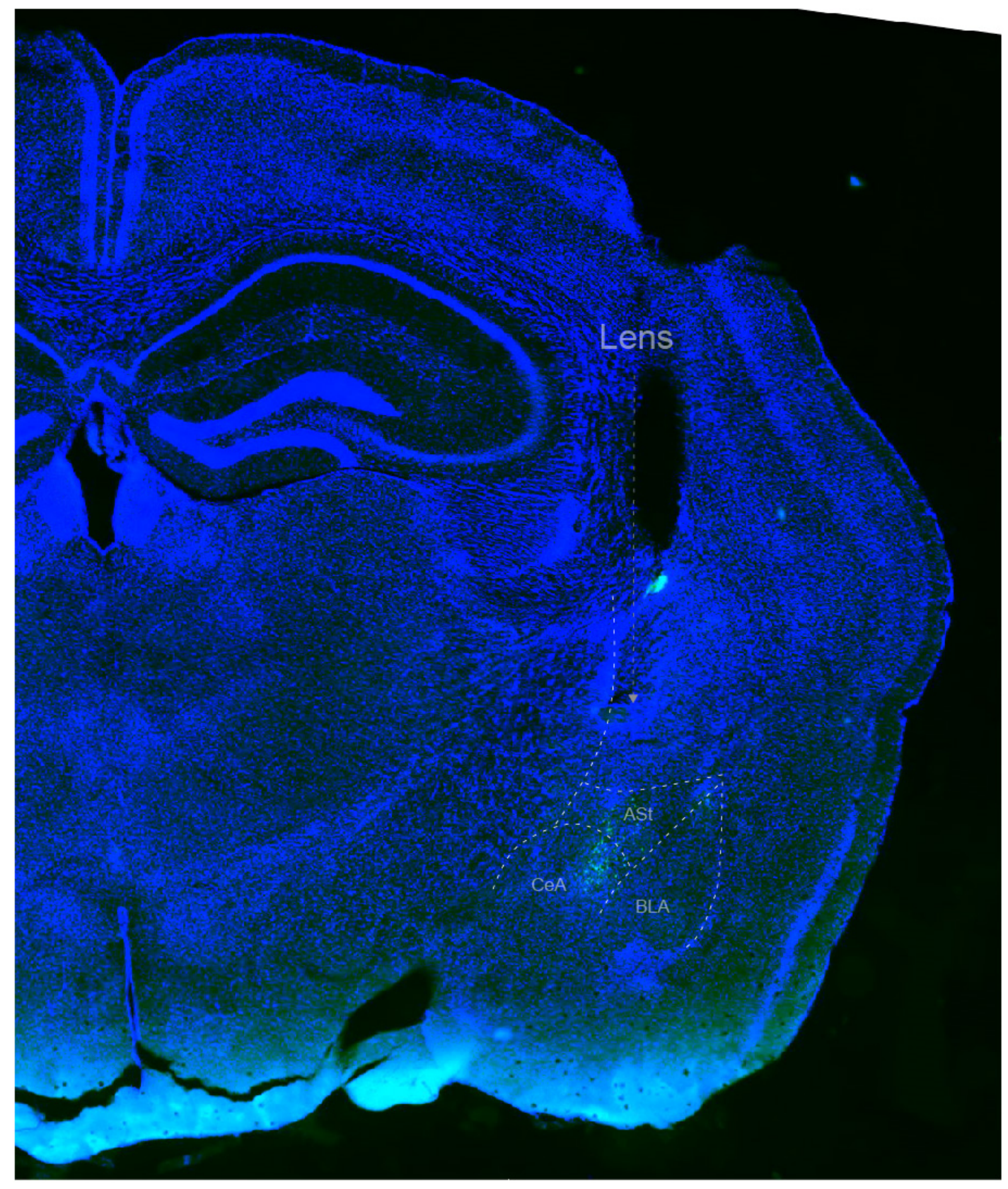

Lens placement dorsal, some viral spread but ASt cells directly under lens ✓

Green = GCaMP7f
Blue = DAPI

Drd1a-Cre Unpaired Mouse ID: CACO3_6852_L_yeti Data included

No good cells, so no data in final analysis

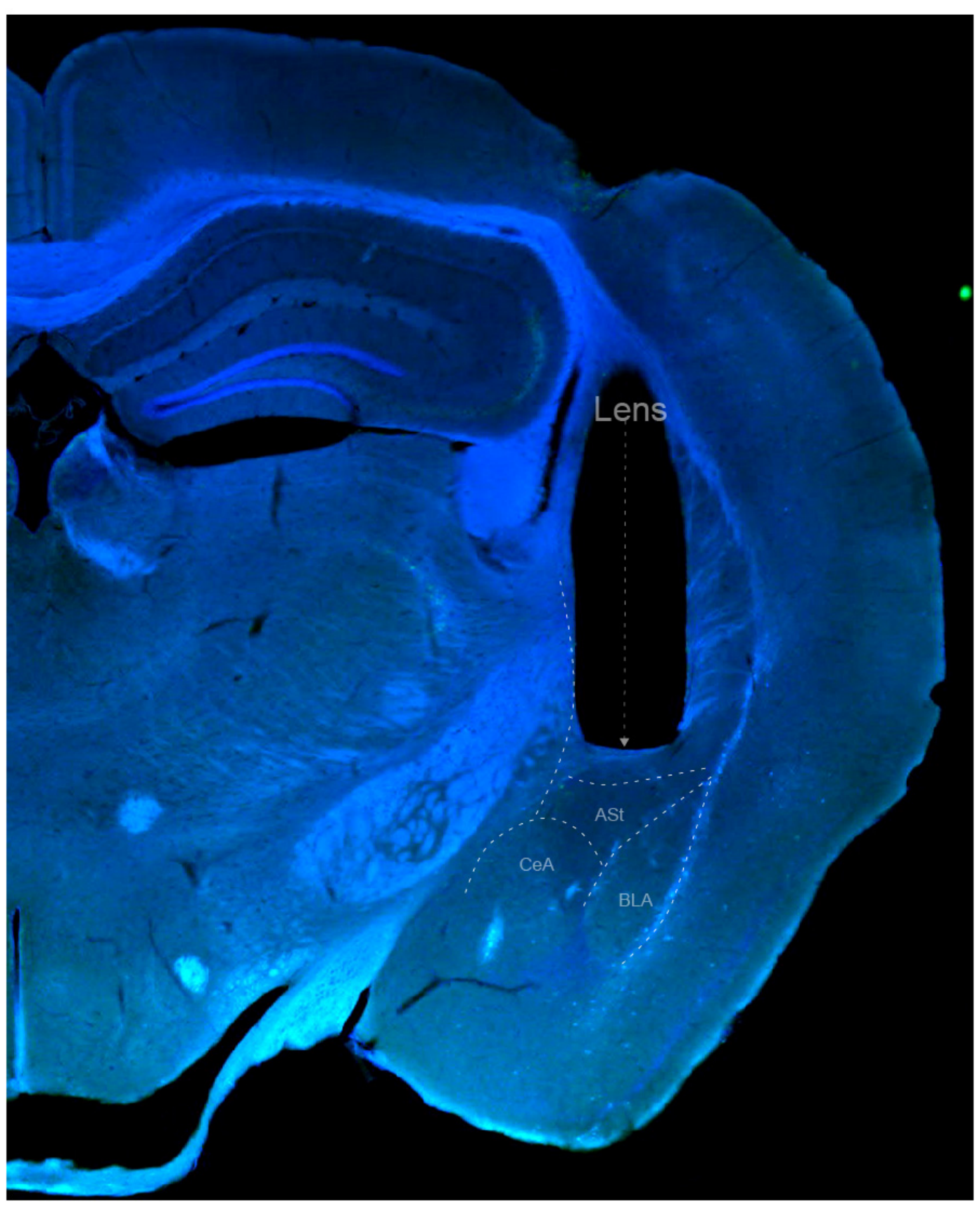

Great lens placement over ASt, expression sparse but present ✓

Green = GCaMP7f
Blue = DAPI

Drd1a-Cre Unpaired Mouse ID: CACO3_6860_L_carl Data included

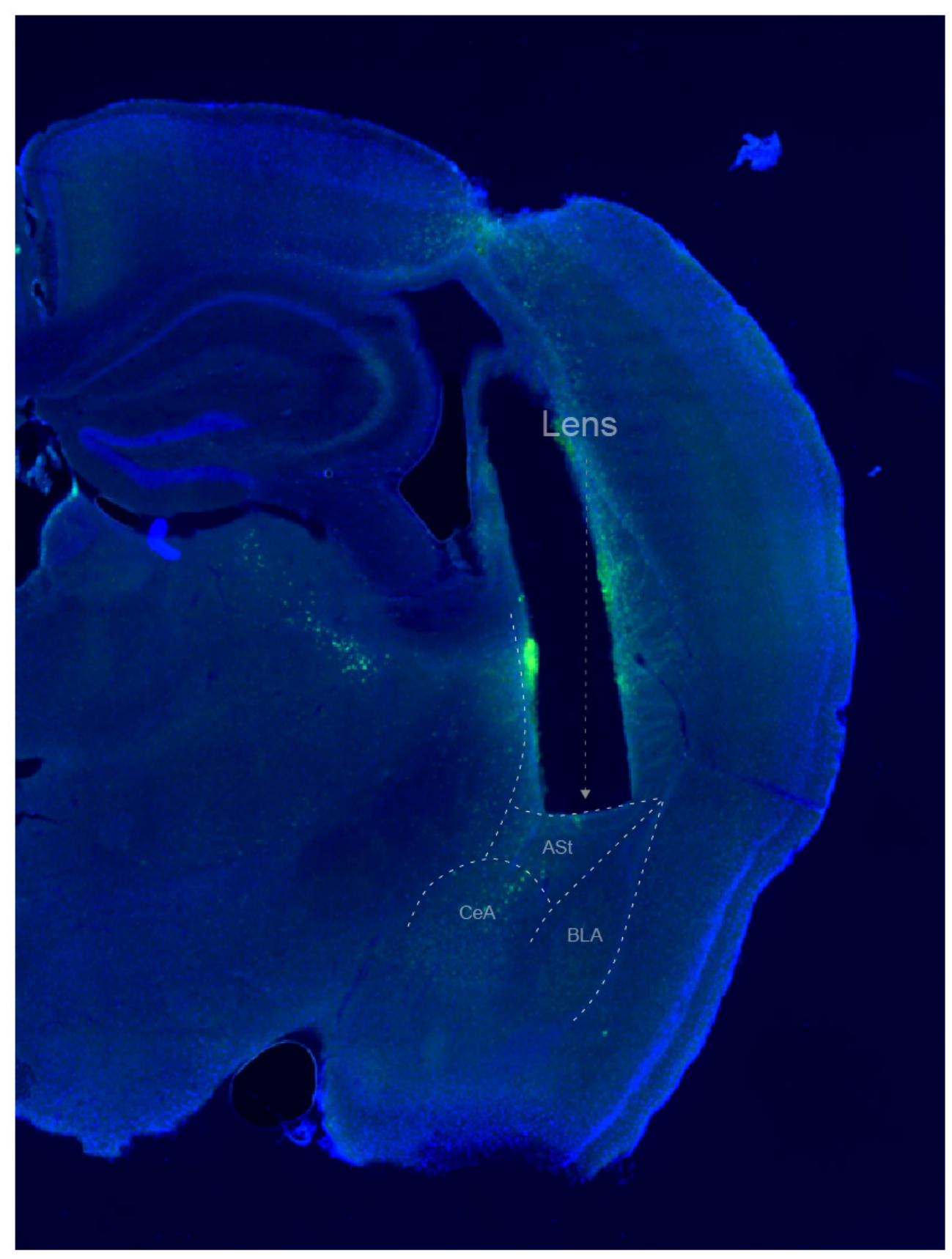

Great lens over ASt ✓

Green = GCaMP7f
Blue = DAPI

Drd1a-Cre Unpaired Mouse ID: CACO3_6891_R_mooneye Data included

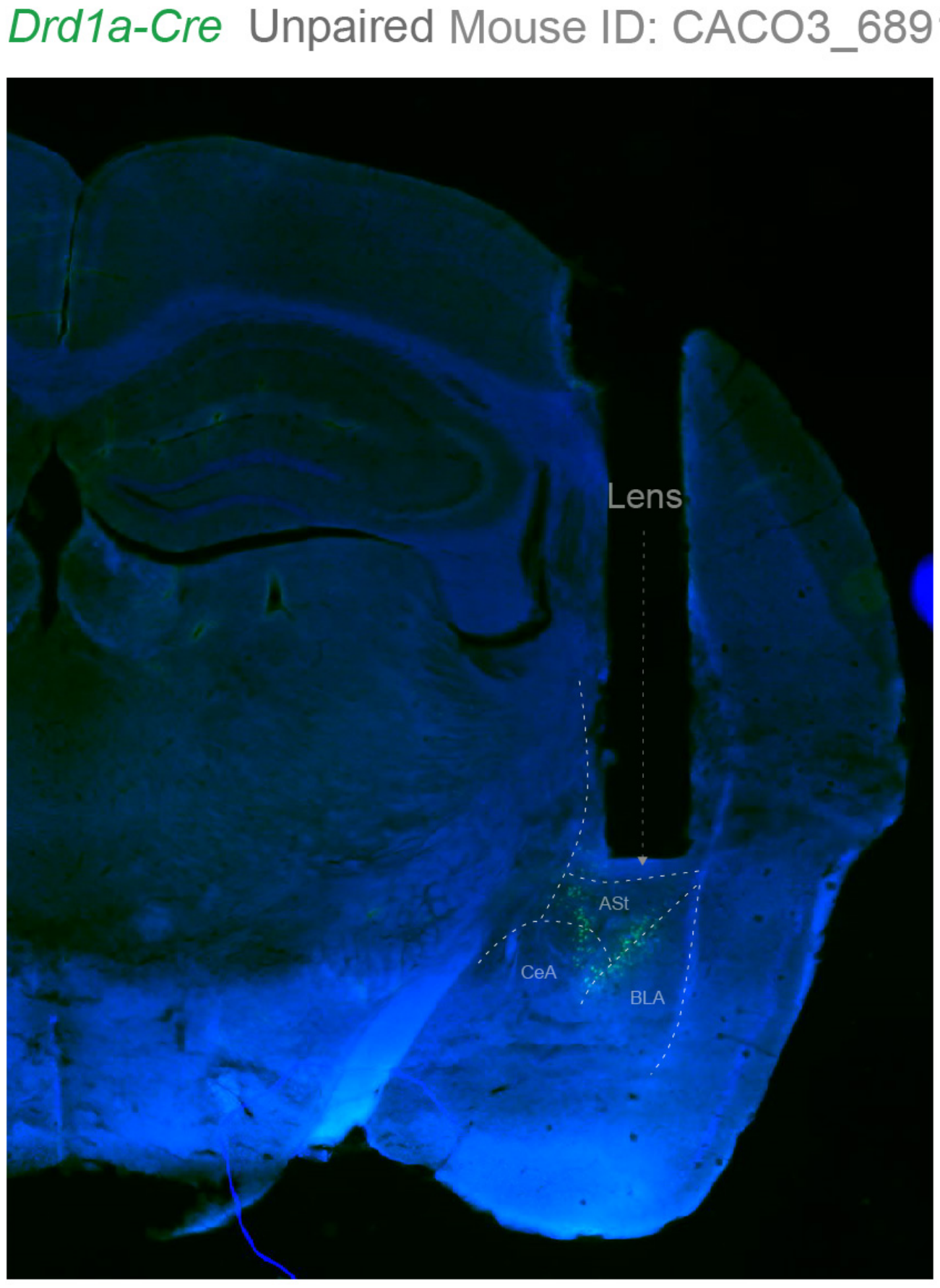

Great lens placement over ASt, expression present ✓

Green = GCaMP7f
Blue = DAPI

Drd1a-Cre Unpaired Mouse ID: CACO5_K036 Data included

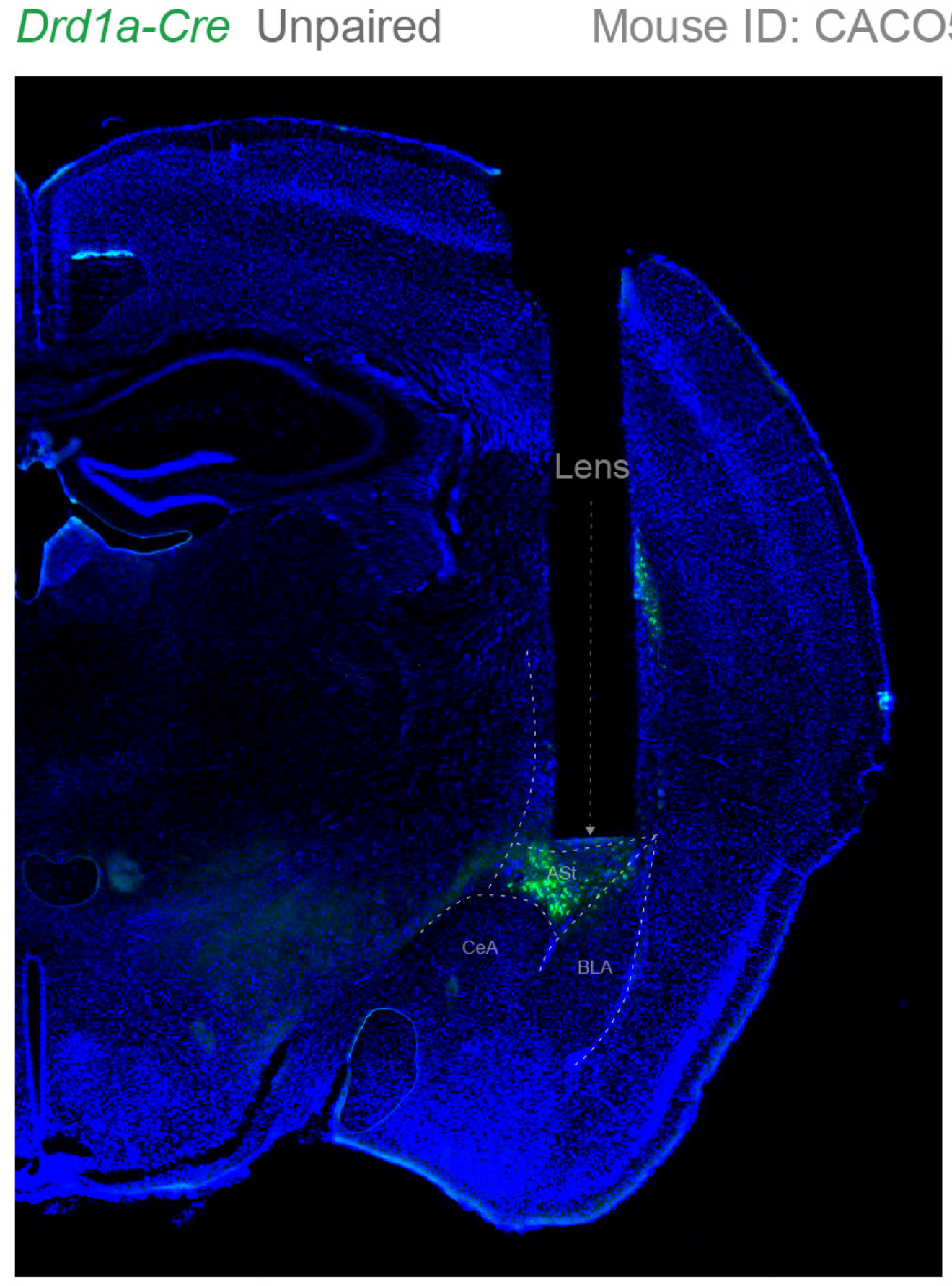

Great lens over ASt and viral expression ✓

Green = GCaMP7f
Blue = DAPI

Appendix D - Histology (Calcium Imaging)

Drd1a-Cre Unpaired group Mouse ID: CACO5_K054 Data included

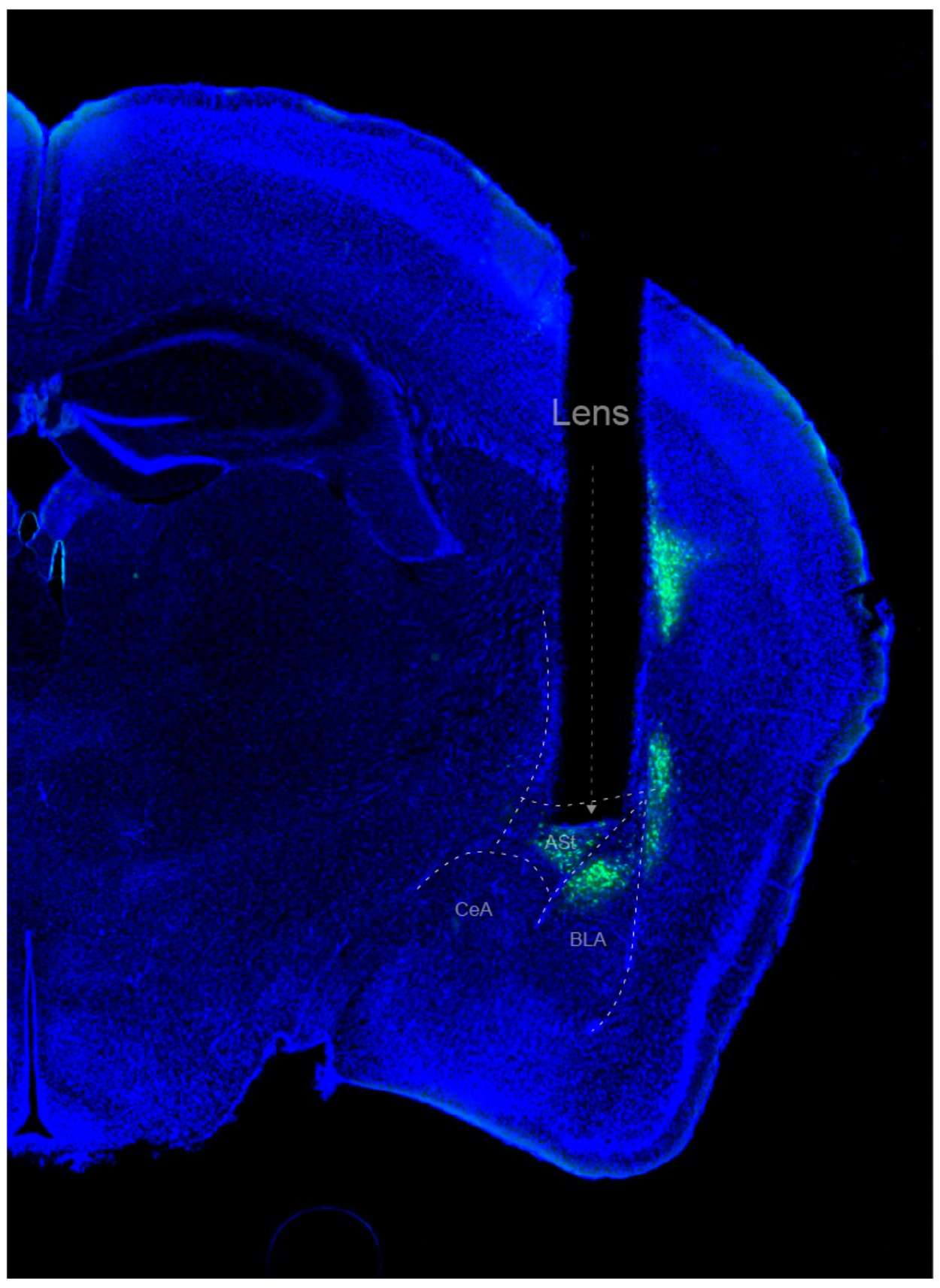

Great lens over ASt and viral expression ✓

Green = GCaMP7f
Blue = DAPI

Drd1a-Cre Unpaired group Mouse ID: CACO5_K010 Data included

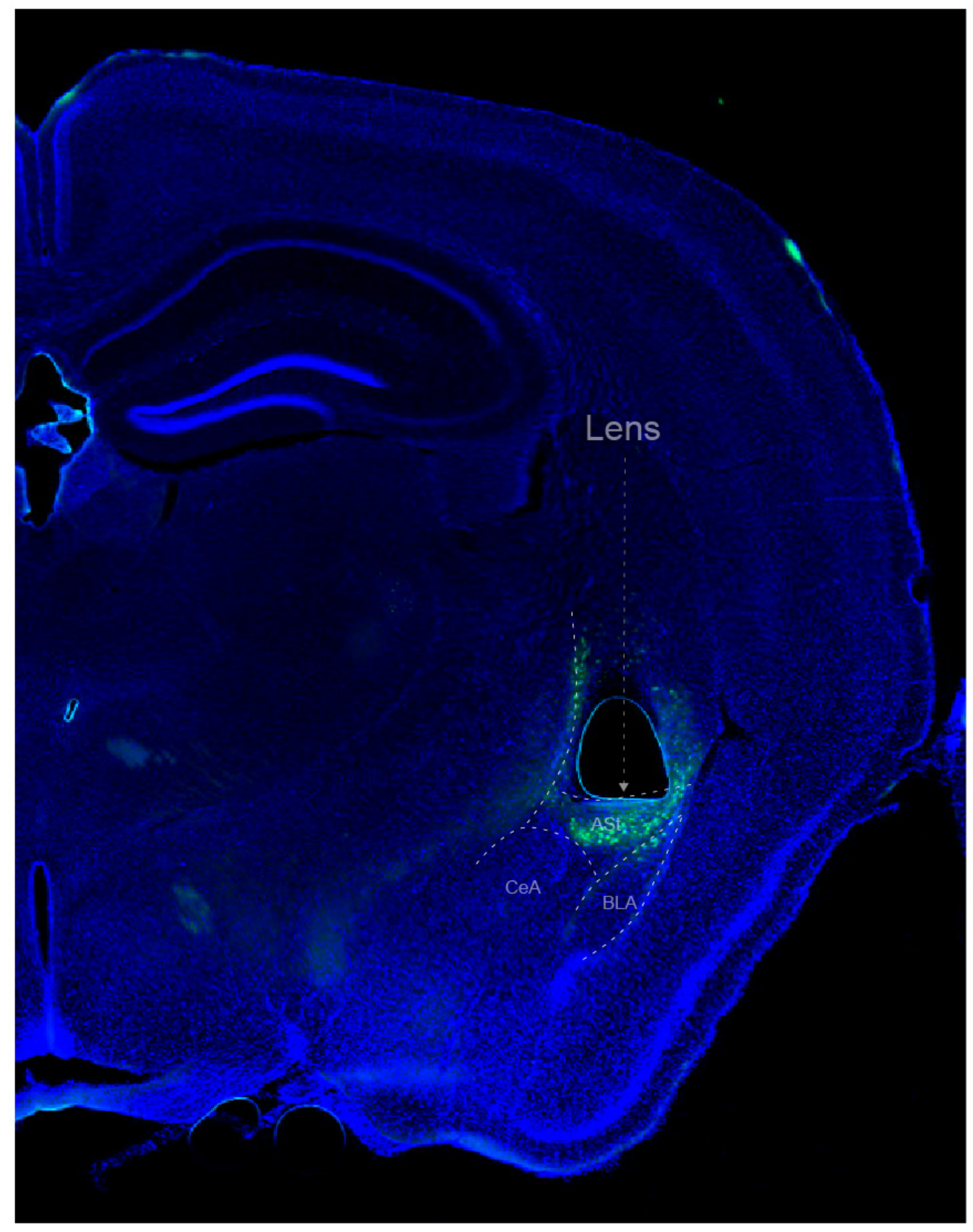

Great lens over ASt and viral expression ✓

Green = GCaMP7f
Blue = DAPI

Drd1a-Cre Unpaired group Mouse ID: CACO5_K029 Data included

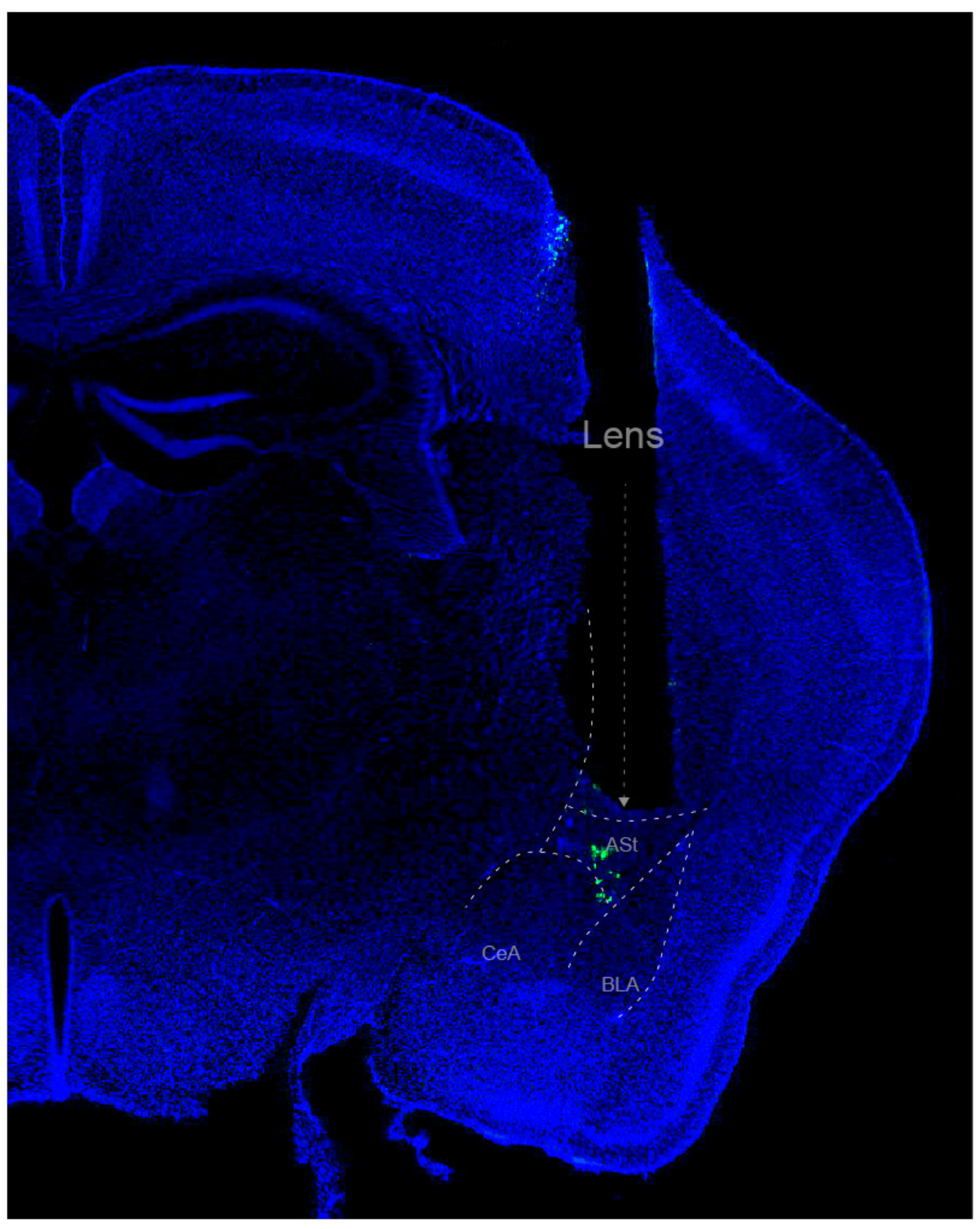

Good lens over ASt ✓

Green = GCaMP7f
Blue = DAPI

Appendix D - Histology (Calcium Imaging)

Drd2-Cre Paired group Mouse ID: CACO2J640

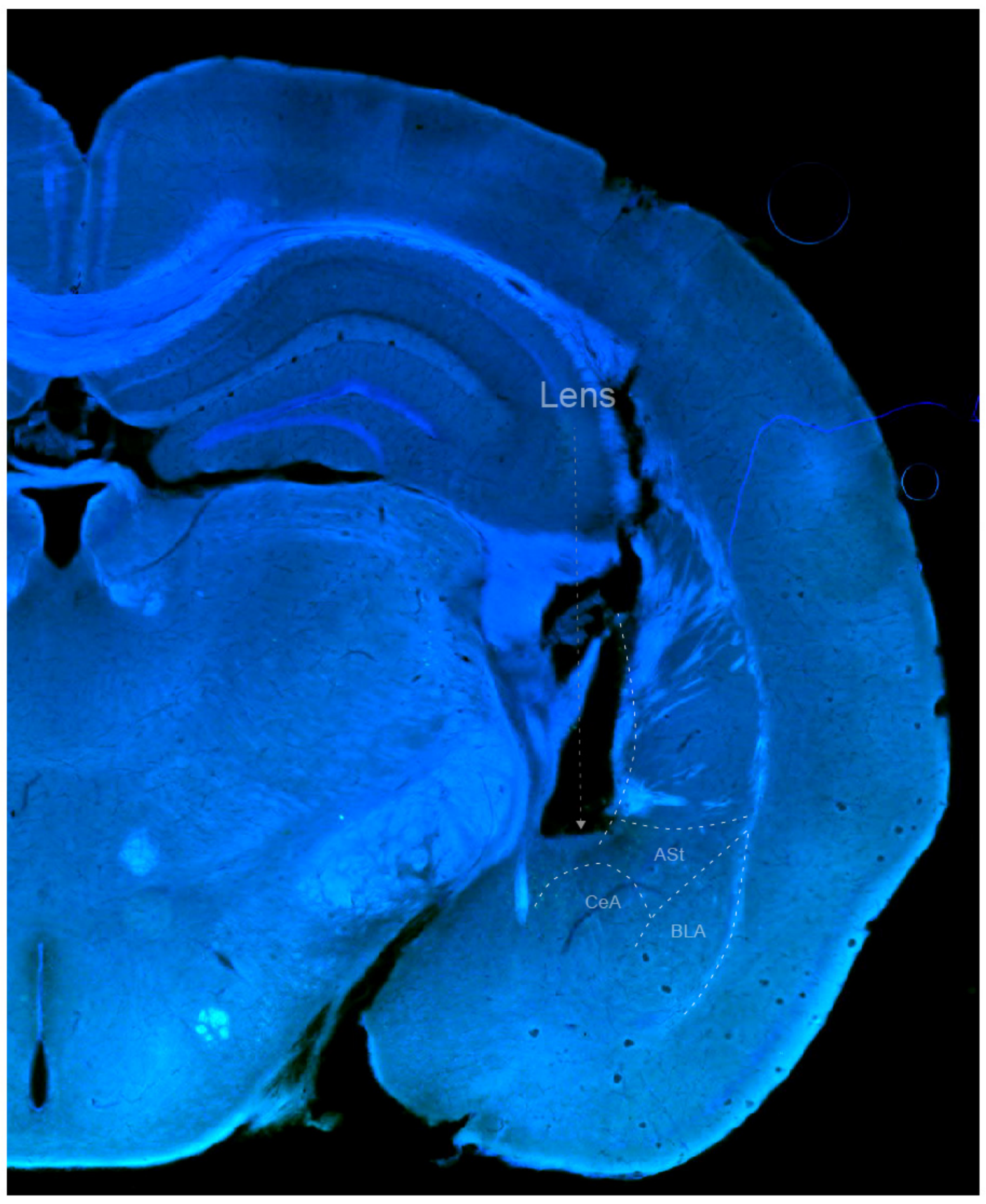

Data from this mouse were not included in the study

Lens too medial, field of view not towards ASt ✘

Green = GCaMP7f
Blue = DAPI

Drd2-Cre Paired group Mouse ID: CACO6J918

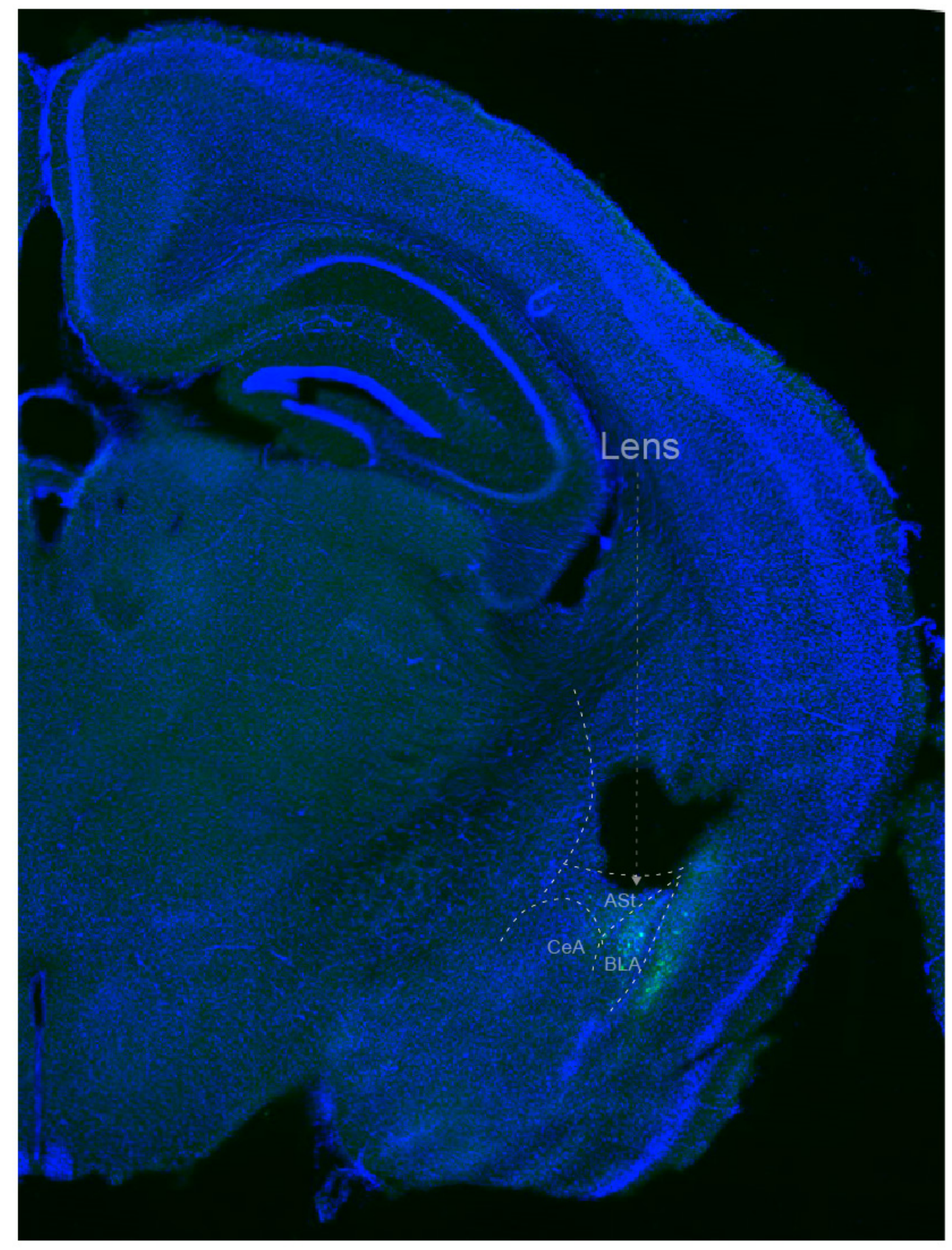

Data from this mouse were not included in the study

Anterior lens placement, LA directly in imaging path

✘

Green = GCaMP7f
Blue = DAPI

Drd2-Cre Unpaired group Mouse ID: CACO5J673

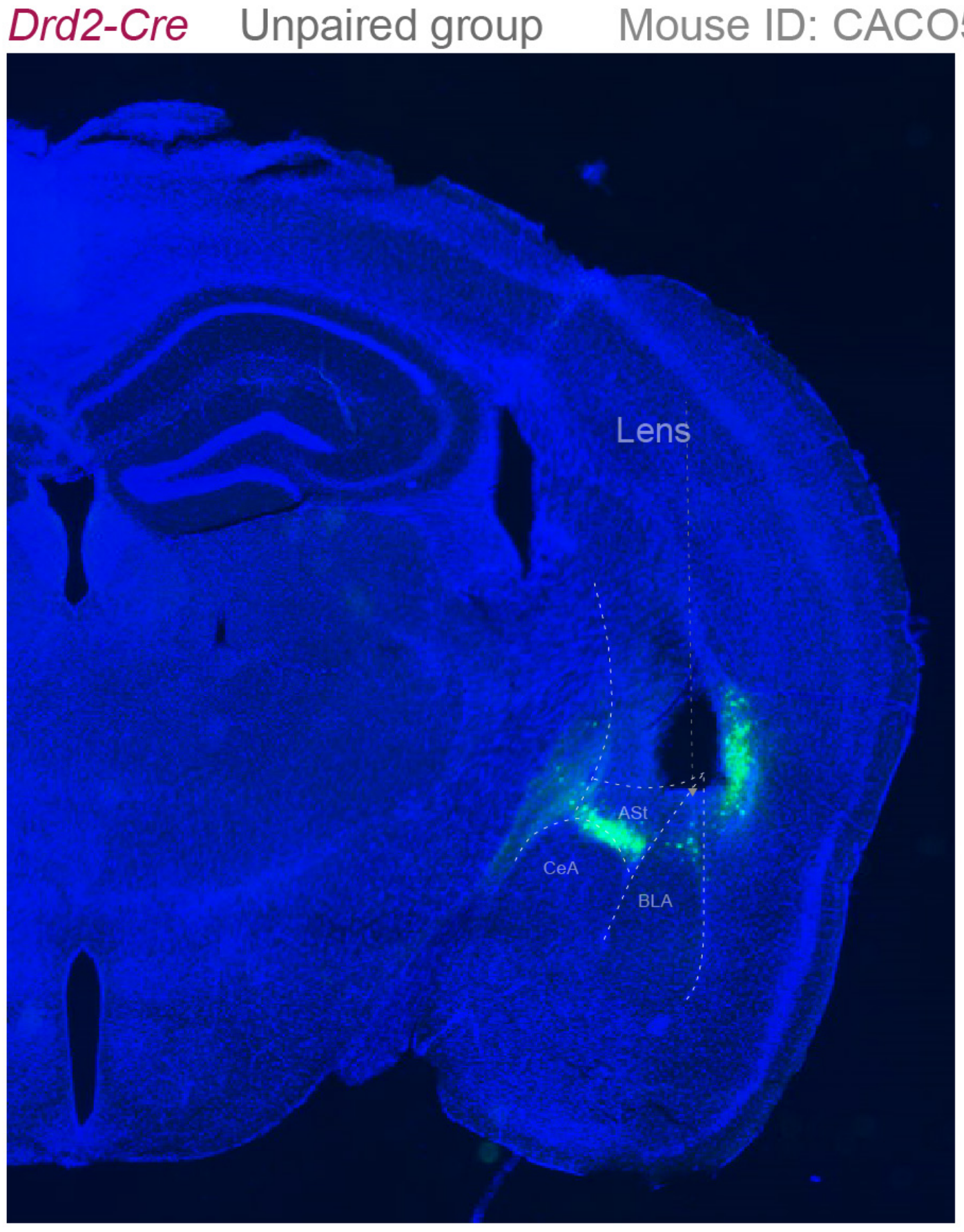

Data from this mouse were not included in the study

Lens too far into cortex and BLA

✘

Green = GCaMP7f
Blue = DAPI

Drd2-Cre Unpaired group Mouse ID: CACO5J930

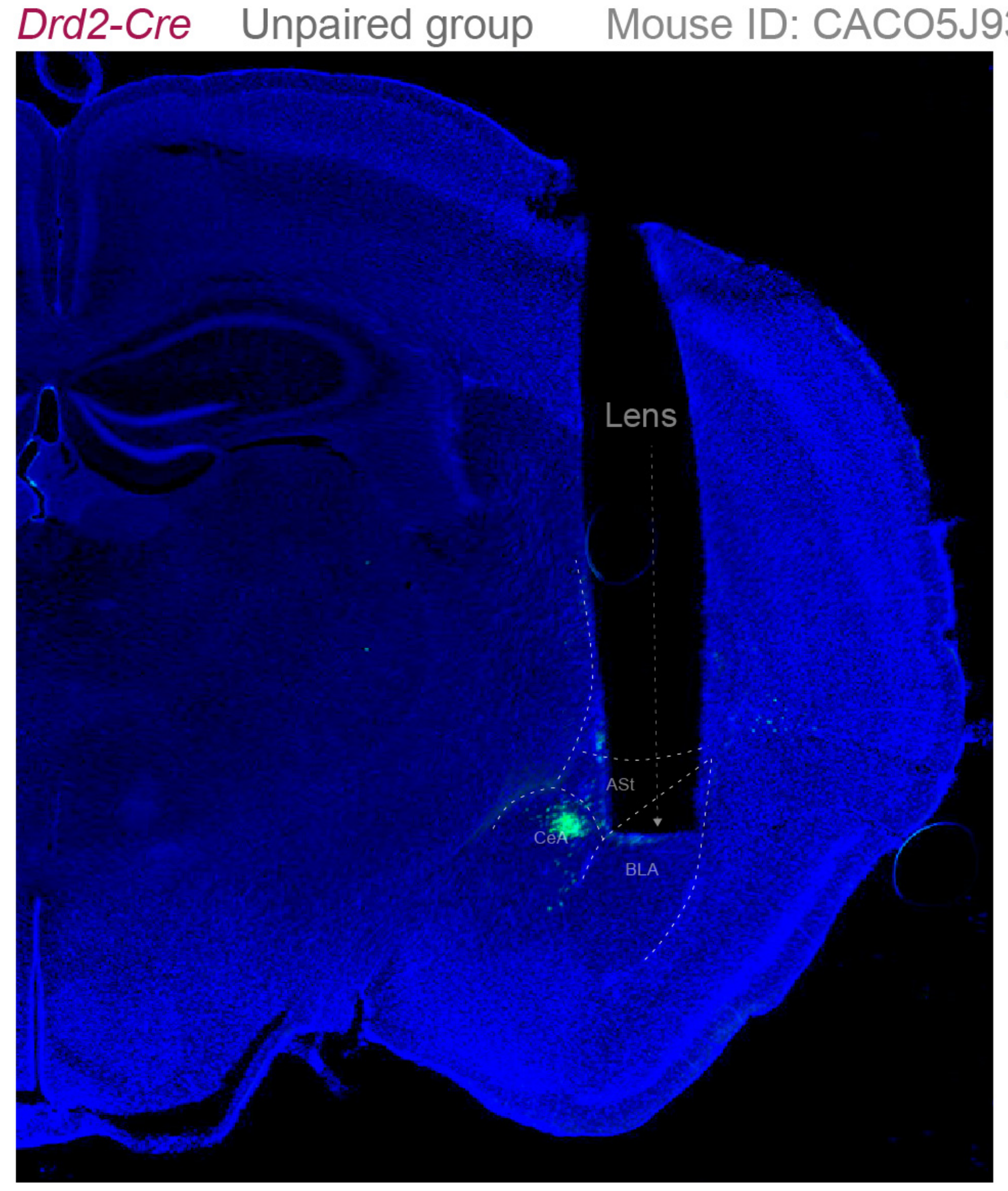

Data from this mouse were not included in the study

Lens too ventral, in BLA

✘

Green = GCaMP7f
Blue = DAPI

Appendix D - Histology (Calcium Imaging)

Drd2-Cre Paired group Mouse ID: CACO2_J642 Data included

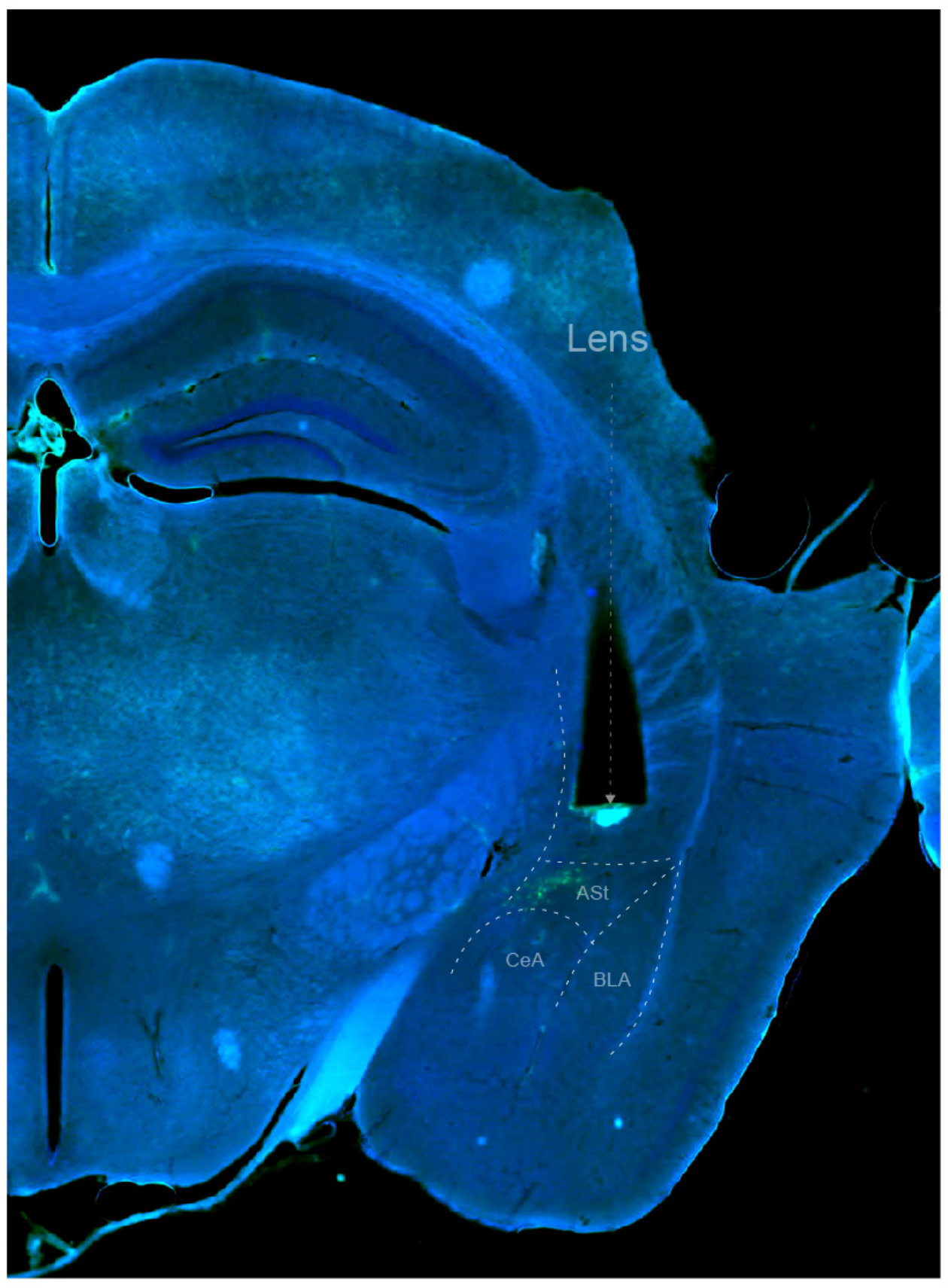

Good lens placement
+ virus in ASt ✓

Green = GCaMP7f
Blue = DAPI

Drd2-Cre Paired group Mouse ID: CACO2_J588 Data included

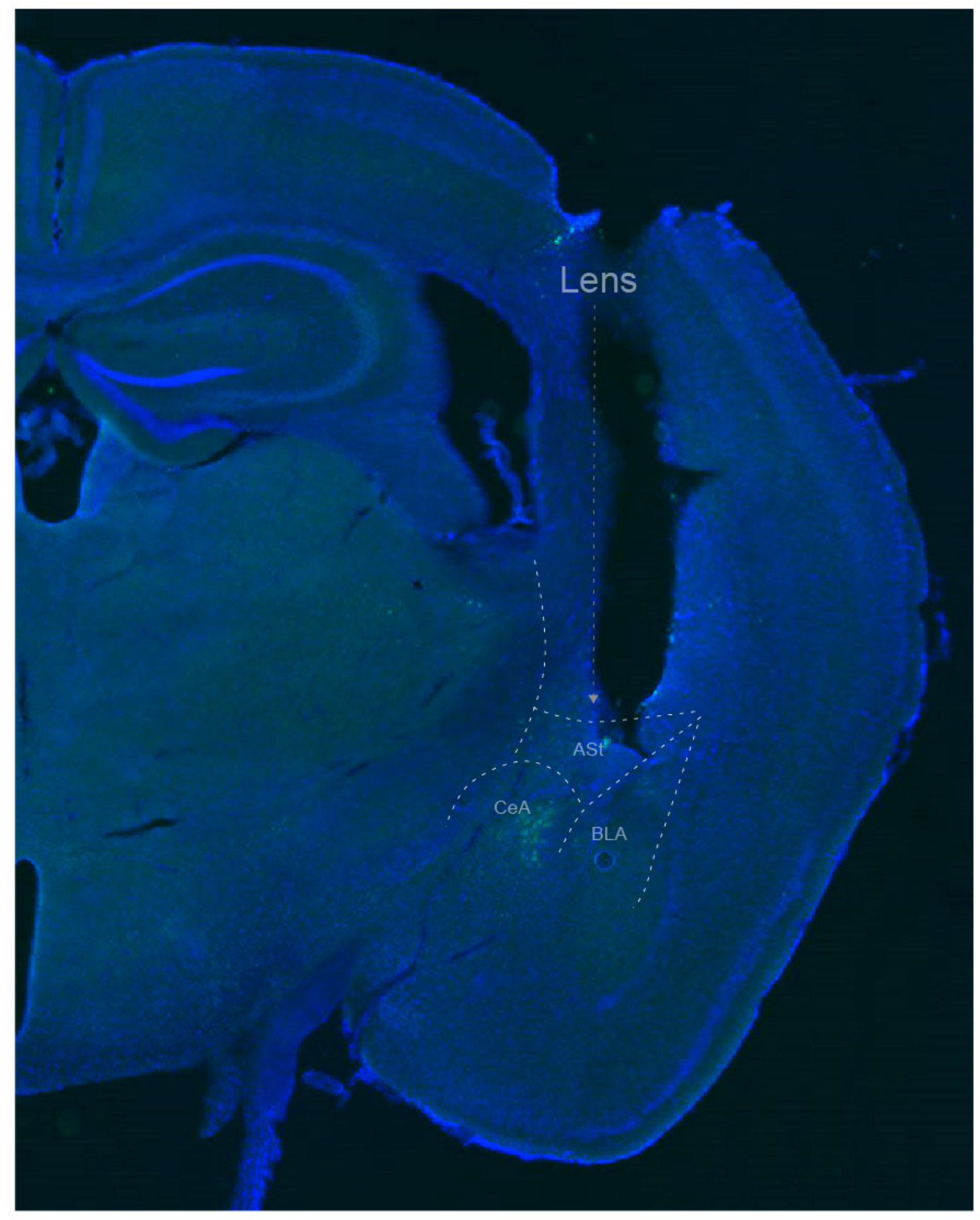

Good lens placement
to ASt ✓

Green = GCaMP7f
Blue = DAPI

Drd2-Cre Paired group Mouse ID: CACO2_J650 Data included

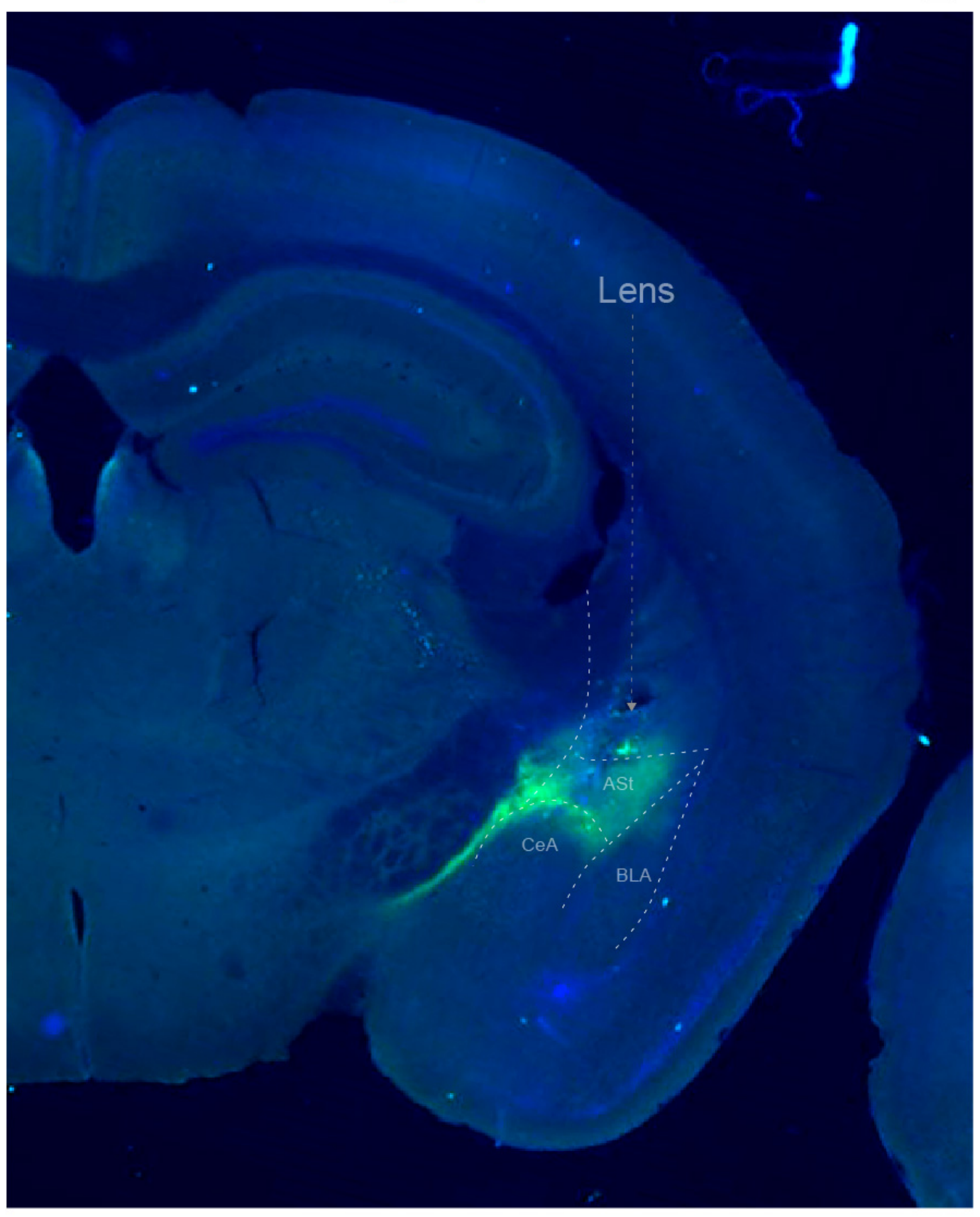

Good lens placement
to ASt , slightly dorsal ✓

Green = GCaMP7f
Blue = DAPI

Drd2-Cre Paired group Mouse ID: CACO5_J895 Data included

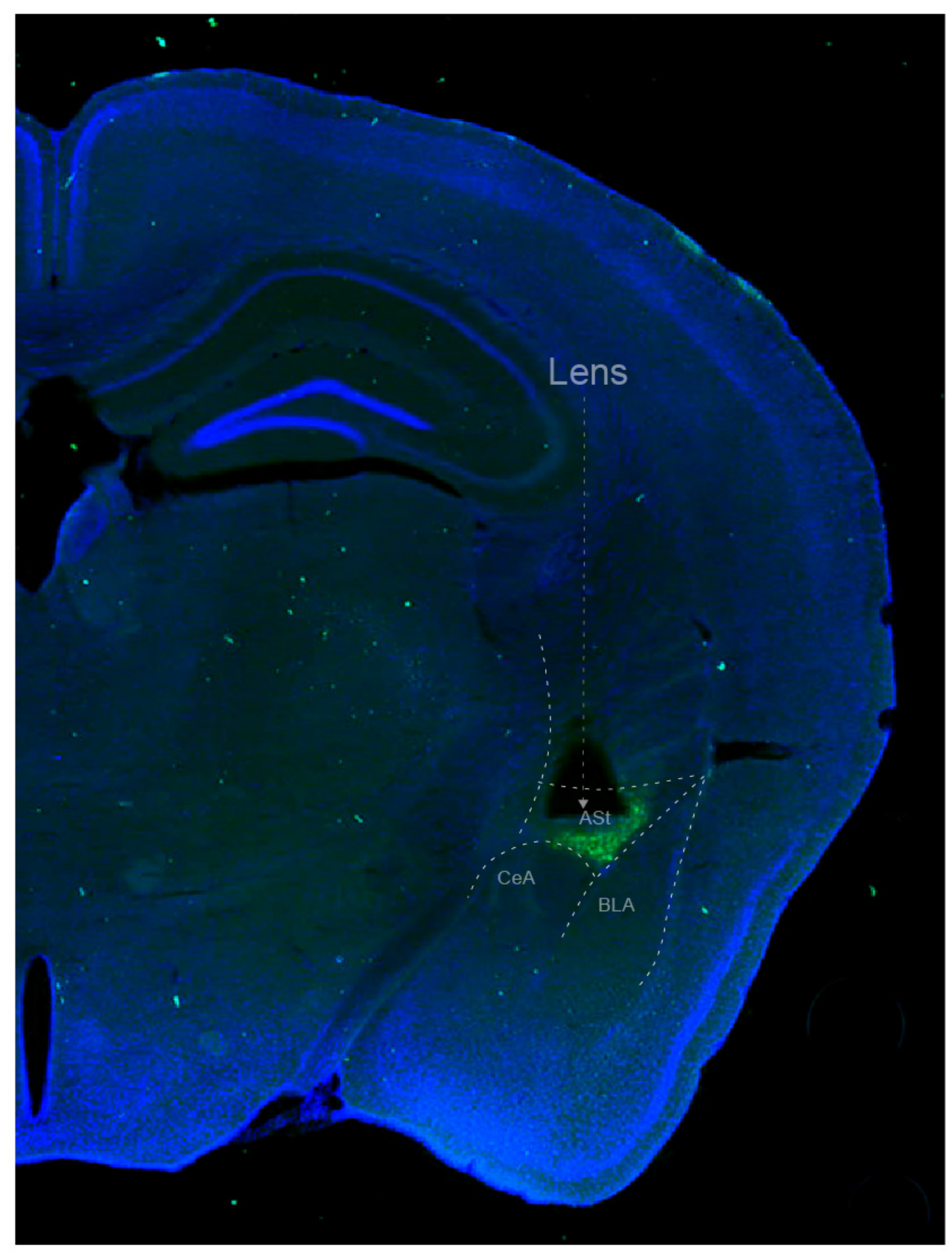

Great lens placement
+ virus in ASt ✓

Green = GCaMP7f
Blue = DAPI

Drd2-Cre Paired group Mouse ID: CACO5_J904 Data included

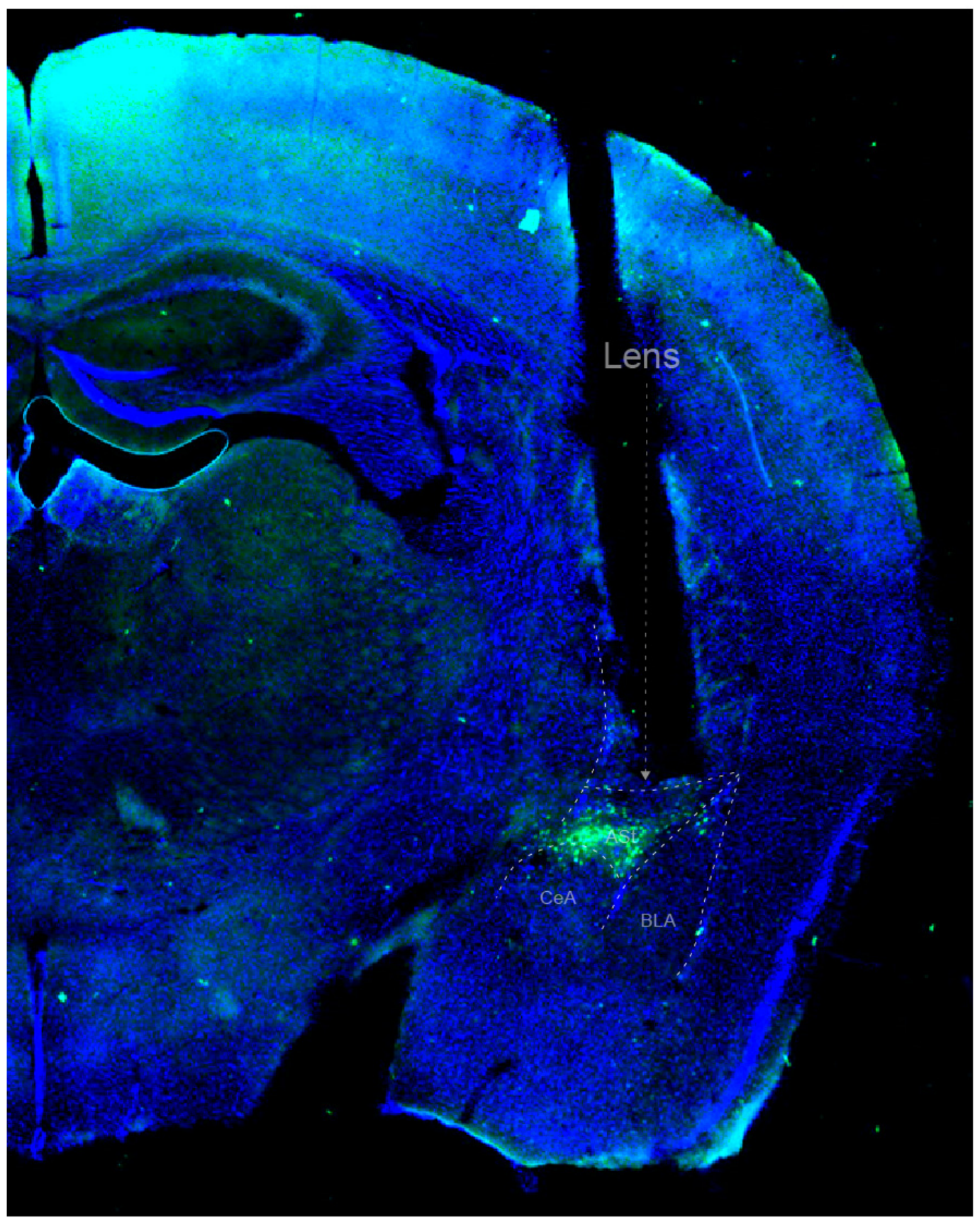

<- Note: diffuse green
labelling due to minor
histology processing
error, not virus

Good lens placement
+ virus in ASt ✓

Green = GCaMP7f
Blue = DAPI

Drd2-Cre Paired group Mouse ID: CACO5_J908 Data included

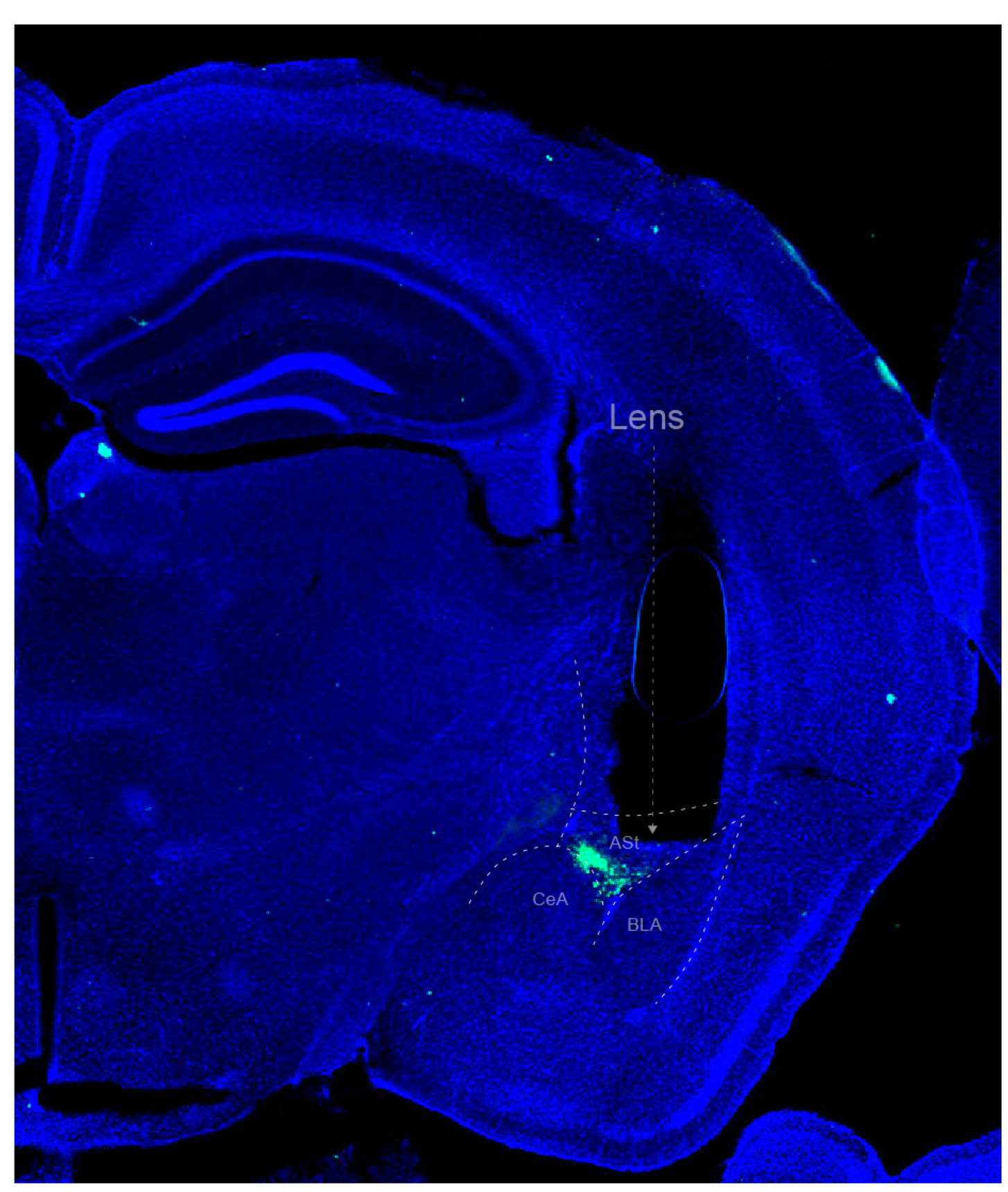

Good lens placement
+ virus in ASt
✓

Green = GCaMP7f
Blue = DAPI

Appendix D - Histology (Calcium Imaging)

Drd2-Cre Paired group Mouse ID: CACO5J907 Data included

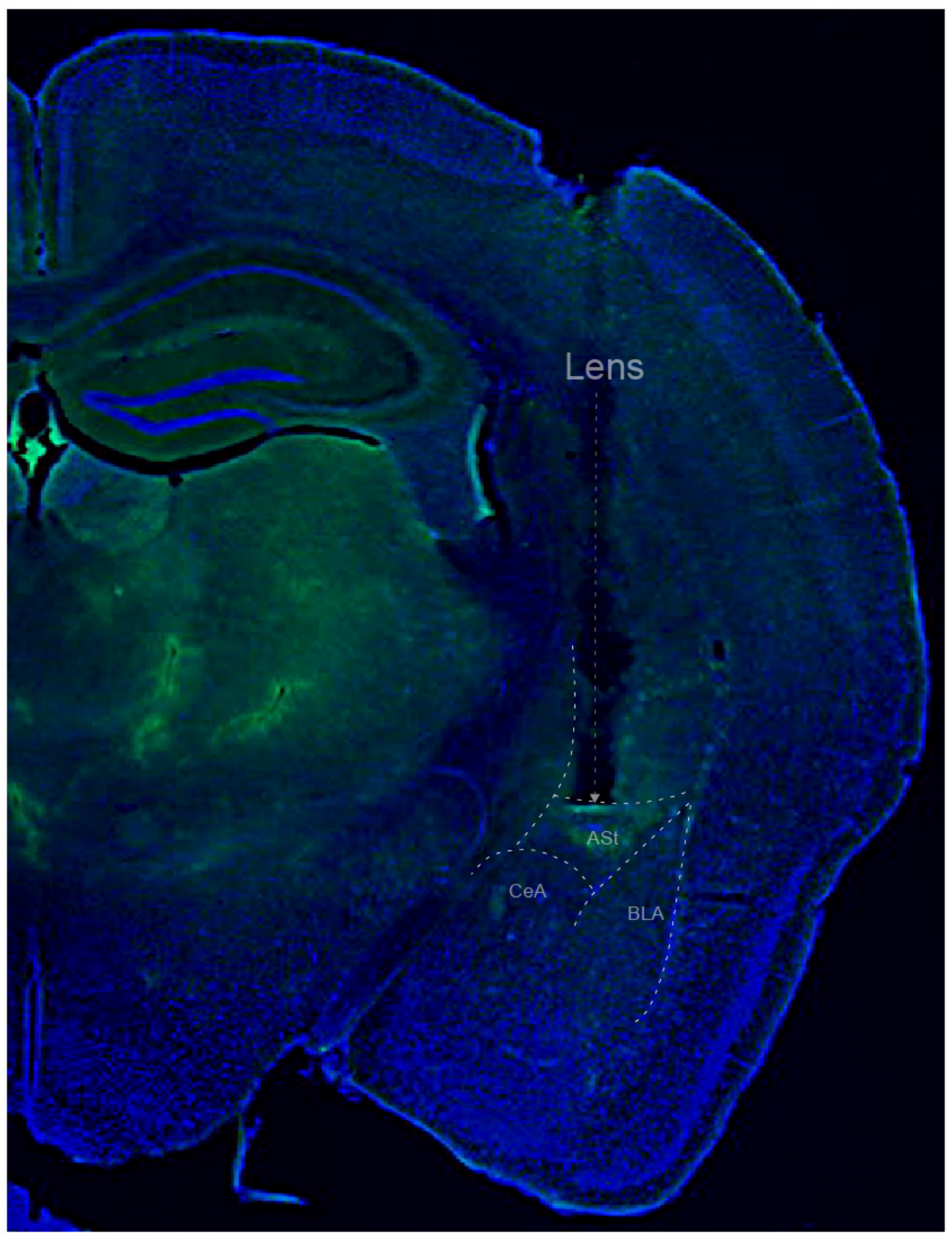

Good lens placement
+ virus in ASt
✓

Green = GCaMP7f
Blue = DAPI

Drd2-Cre Paired group Mouse ID: CACO6J932 Data included

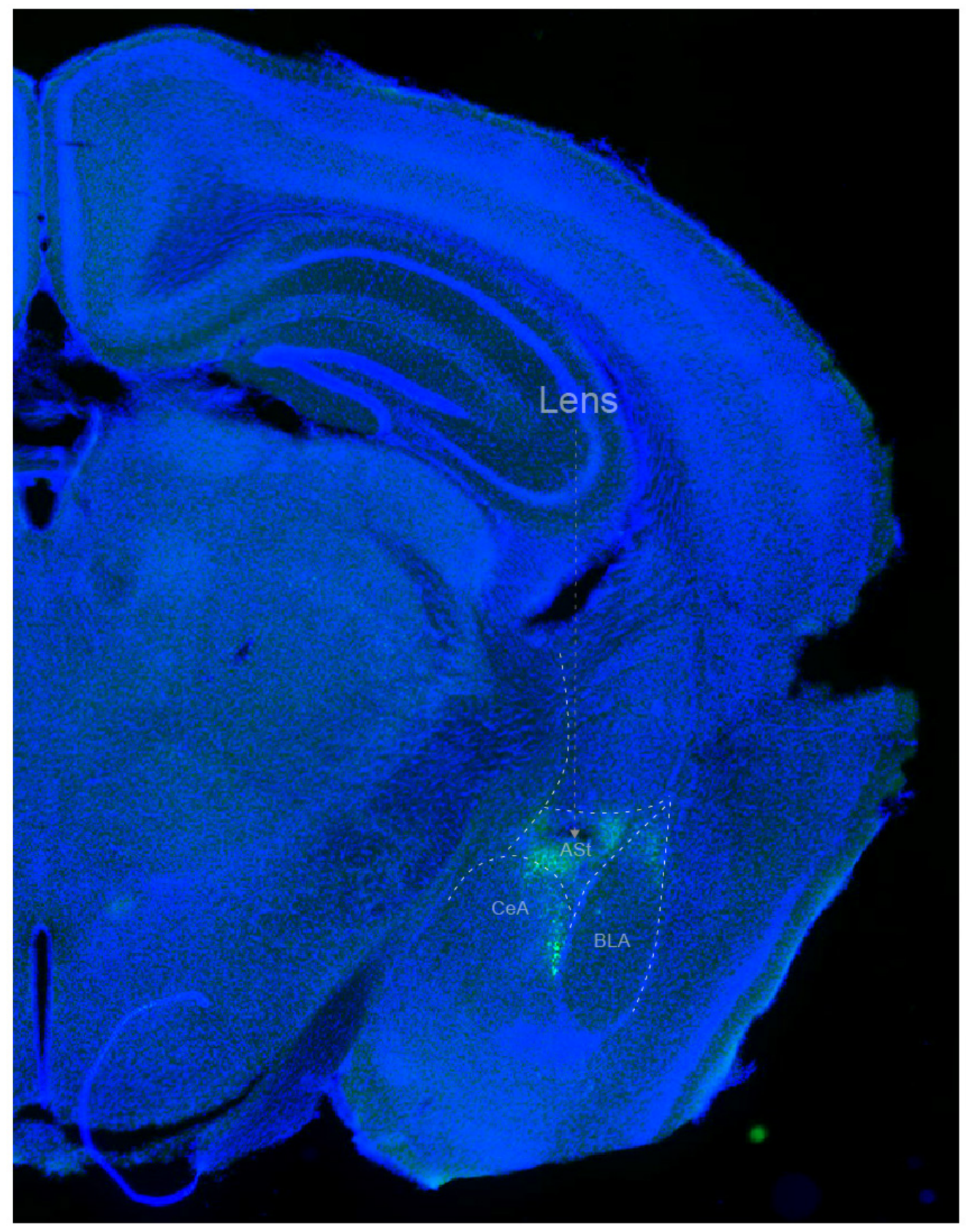

Good lens placement
+ virus in ASt
✓

Green = GCaMP7f
Blue = DAPI

Drd2-Cre Paired group Mouse ID: CACO6J933 Data included

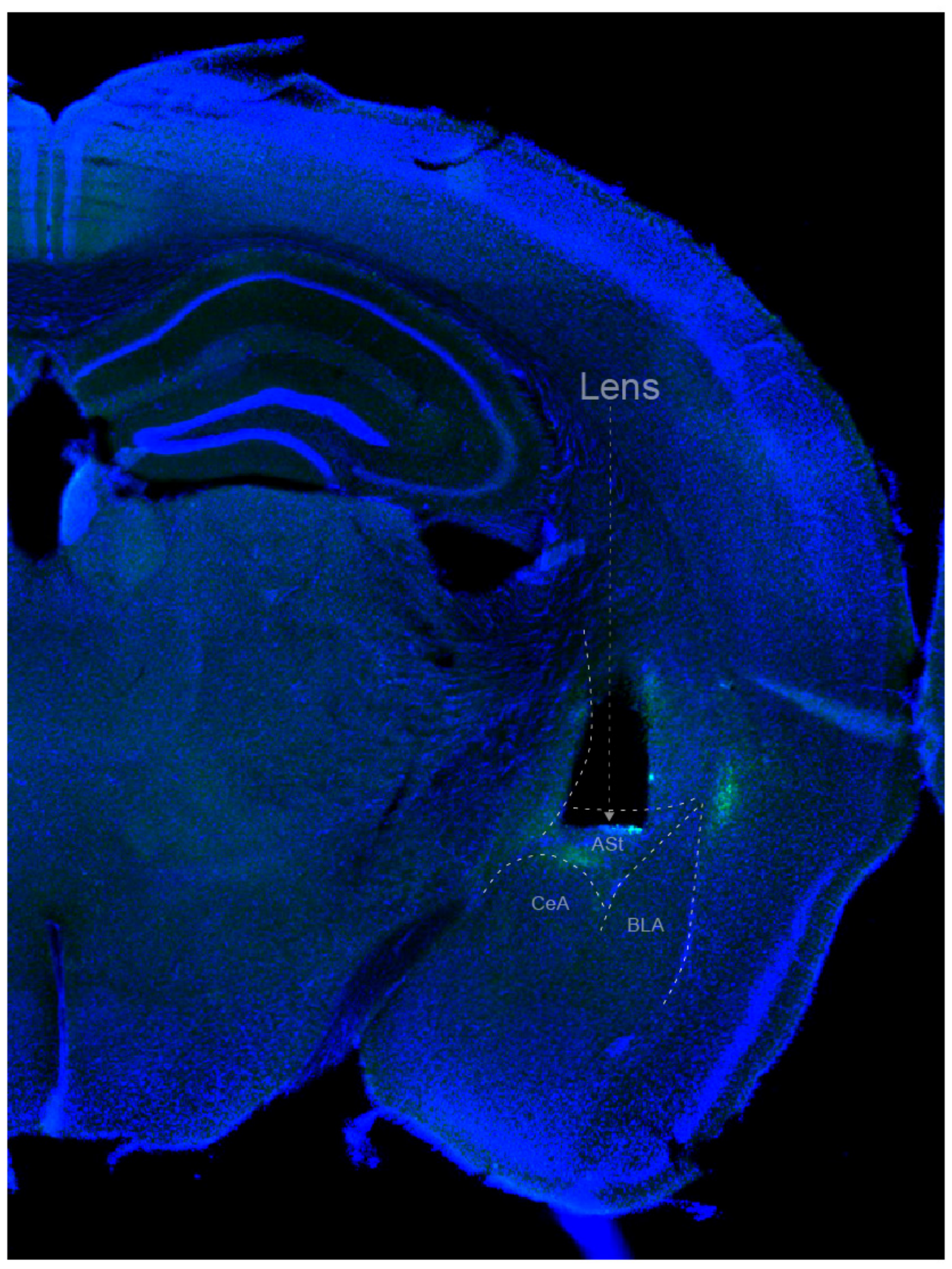

Good lens placement
+ virus in ASt
✓

Green = GCaMP7f
Blue = DAPI

Appendix D - Histology (Calcium Imaging)

Drd2-Cre Unpaired group Mouse ID: CACO2_J670 Data included

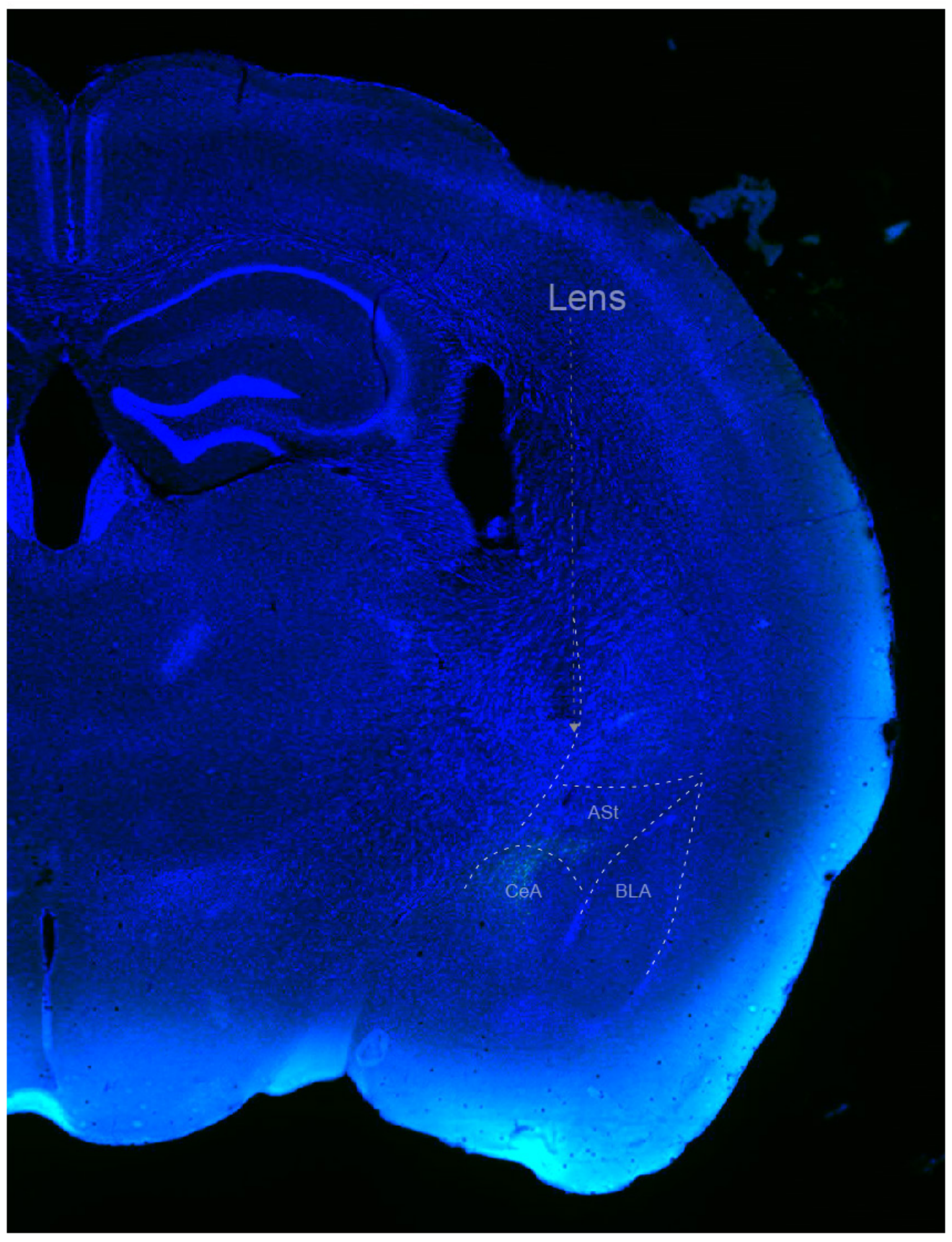

Lens slightly dorsal, but field of view towards ASt, virus present ✓

Green = GCaMP7f
Blue = DAPI

Drd2-Cre Unpaired group Mouse ID: CACO2_J633 Data included

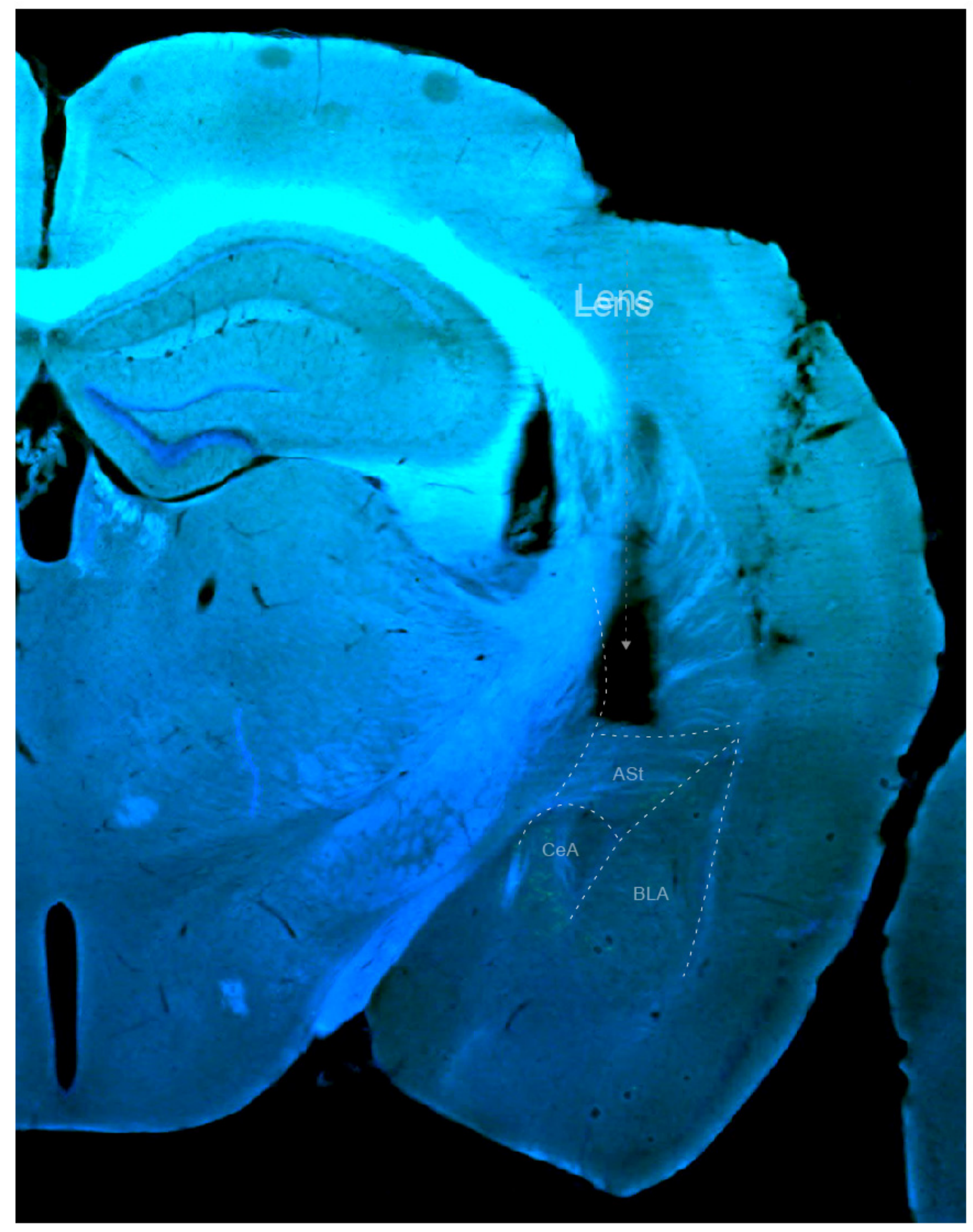

Good lens placement, virus in ASt ✓

Green = GCaMP7f
Blue = DAPI

Drd2-Cre Unpaired group Mouse ID: CACO2_J634 Data included

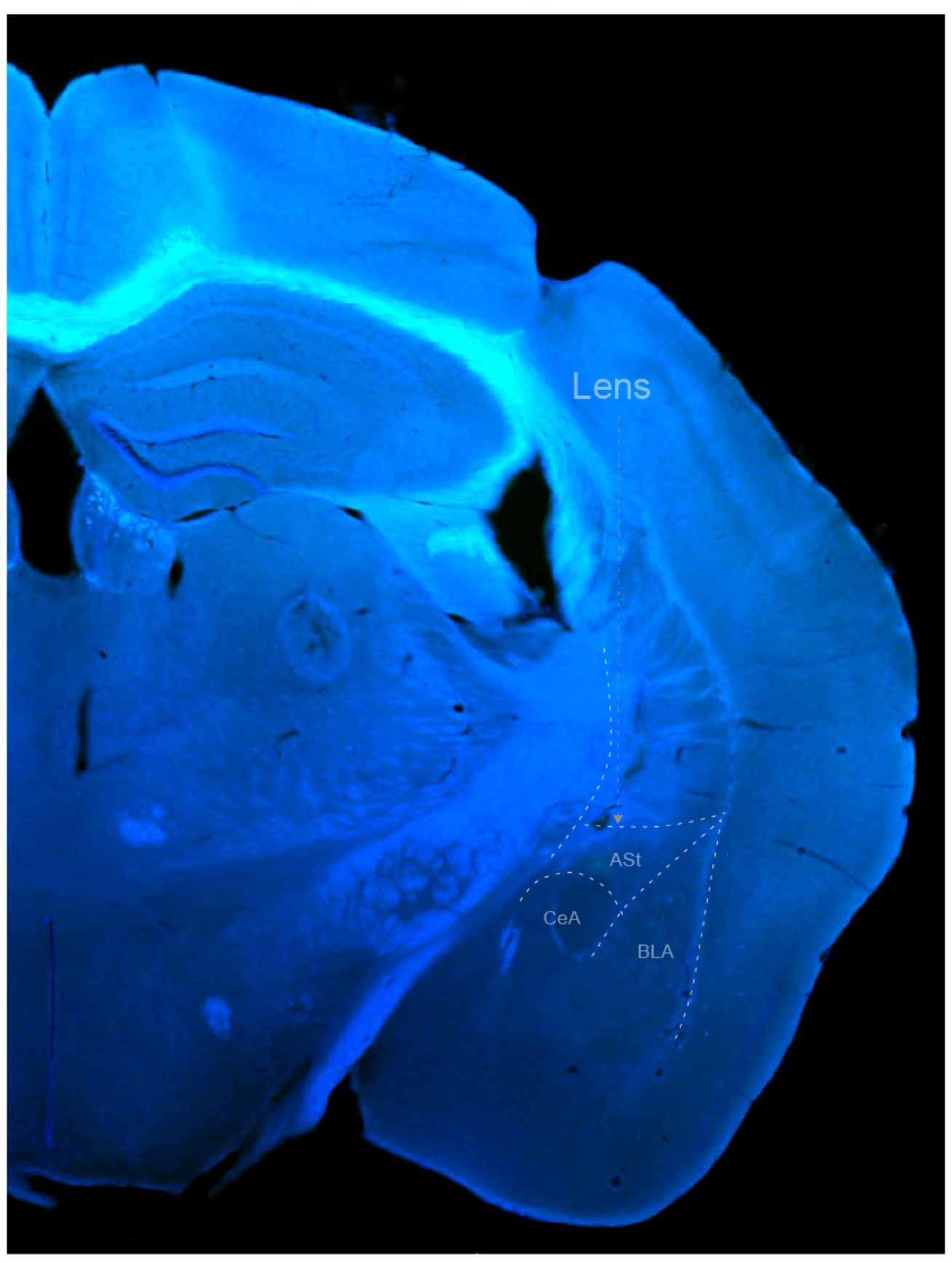

Good lens placement and virus in ASt ✓

Green = GCaMP7f
Blue = DAPI

Drd2-Cre Unpaired group Mouse ID: CACO5_J915 Data included

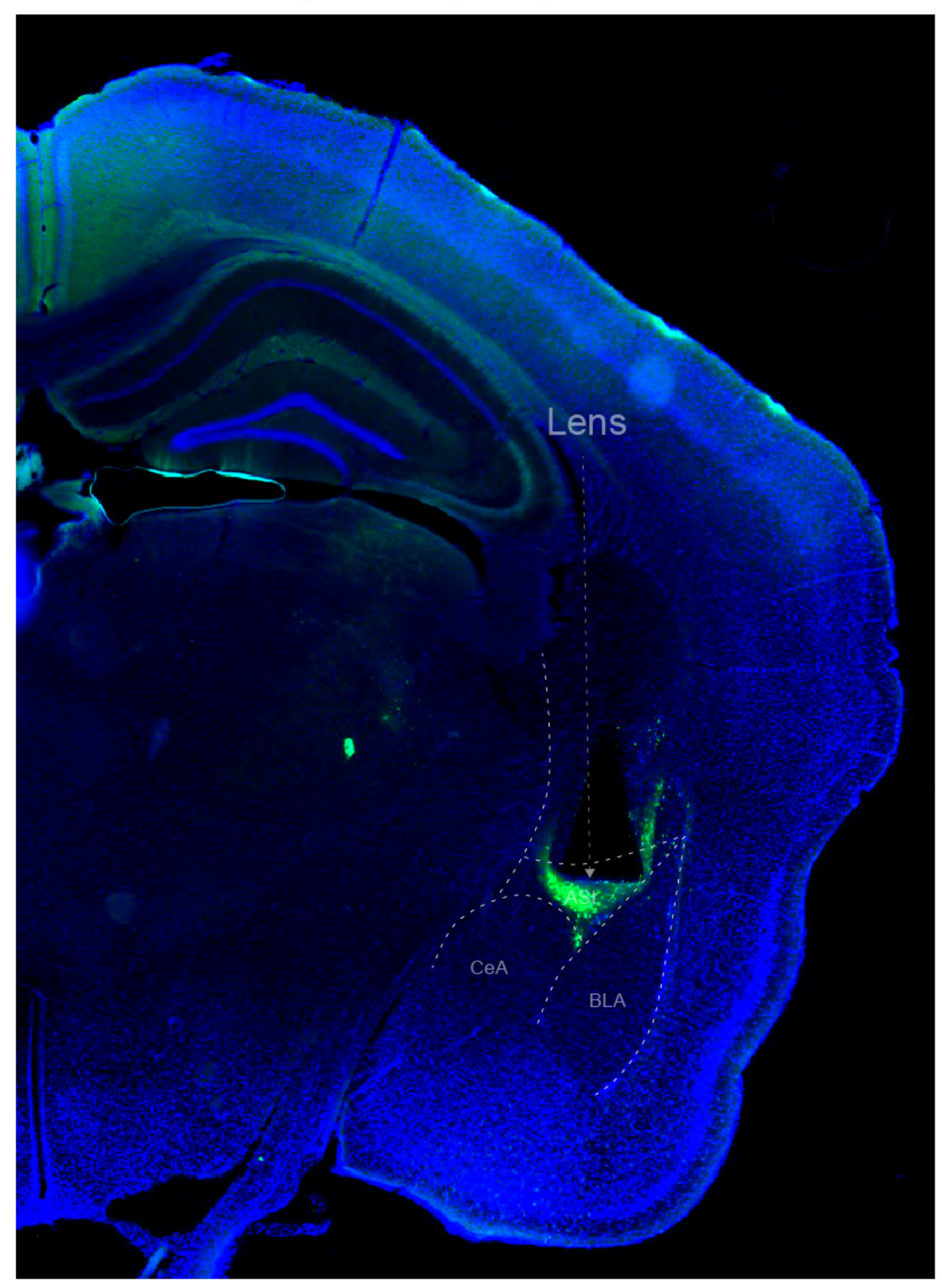

Great lens placement + virus in ASt ✓

Green = GCaMP7f
Blue = DAPI

Drd2-Cre Unpaired group Mouse ID: CACO5_J917 Data included

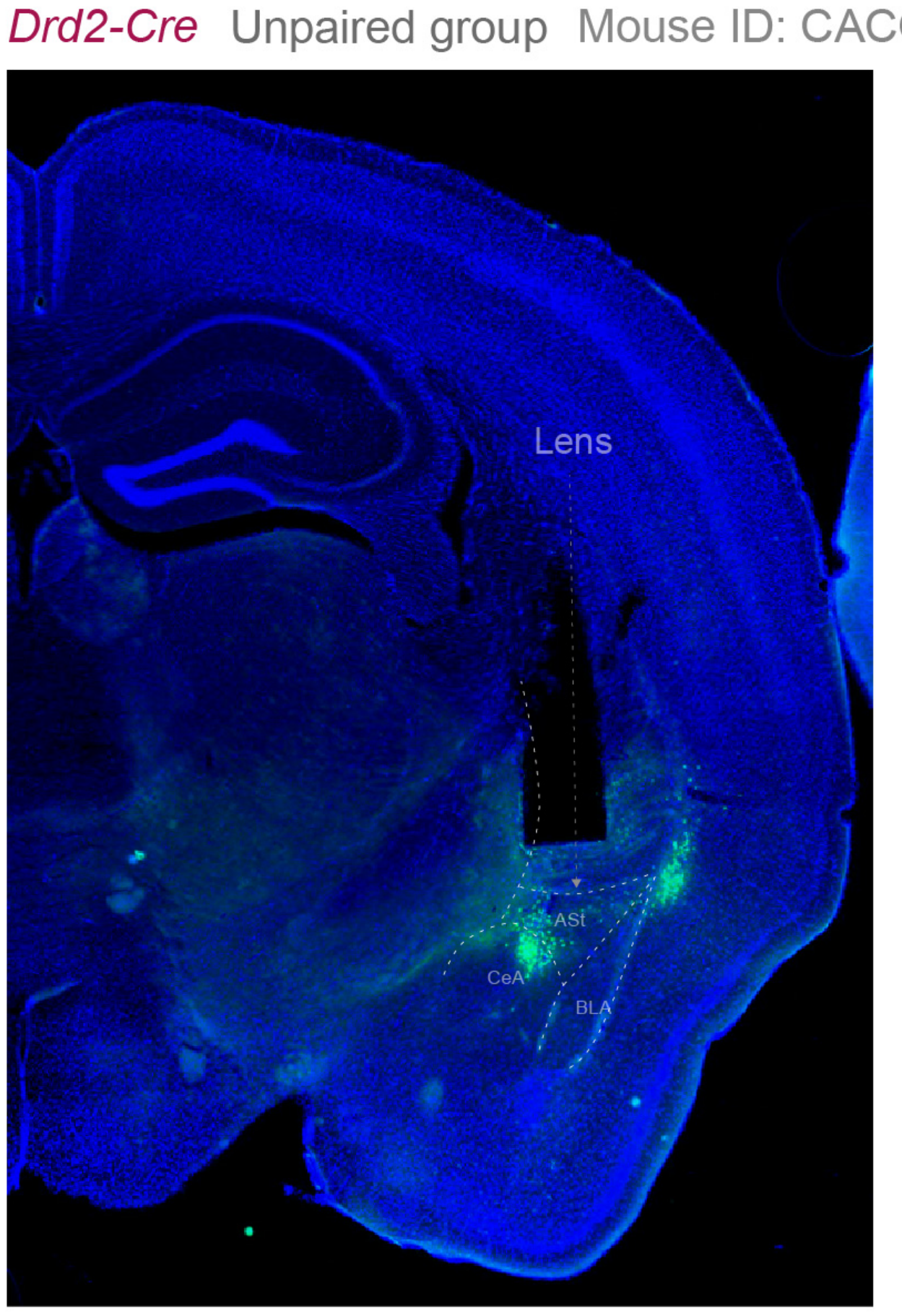

Lens placement slightly dorsal, but field of view still towards ASt ✓

Green = GCaMP7f
Blue = DAPI

Drd2-Cre Unpaired group Mouse ID: CACO5_J919 Data included

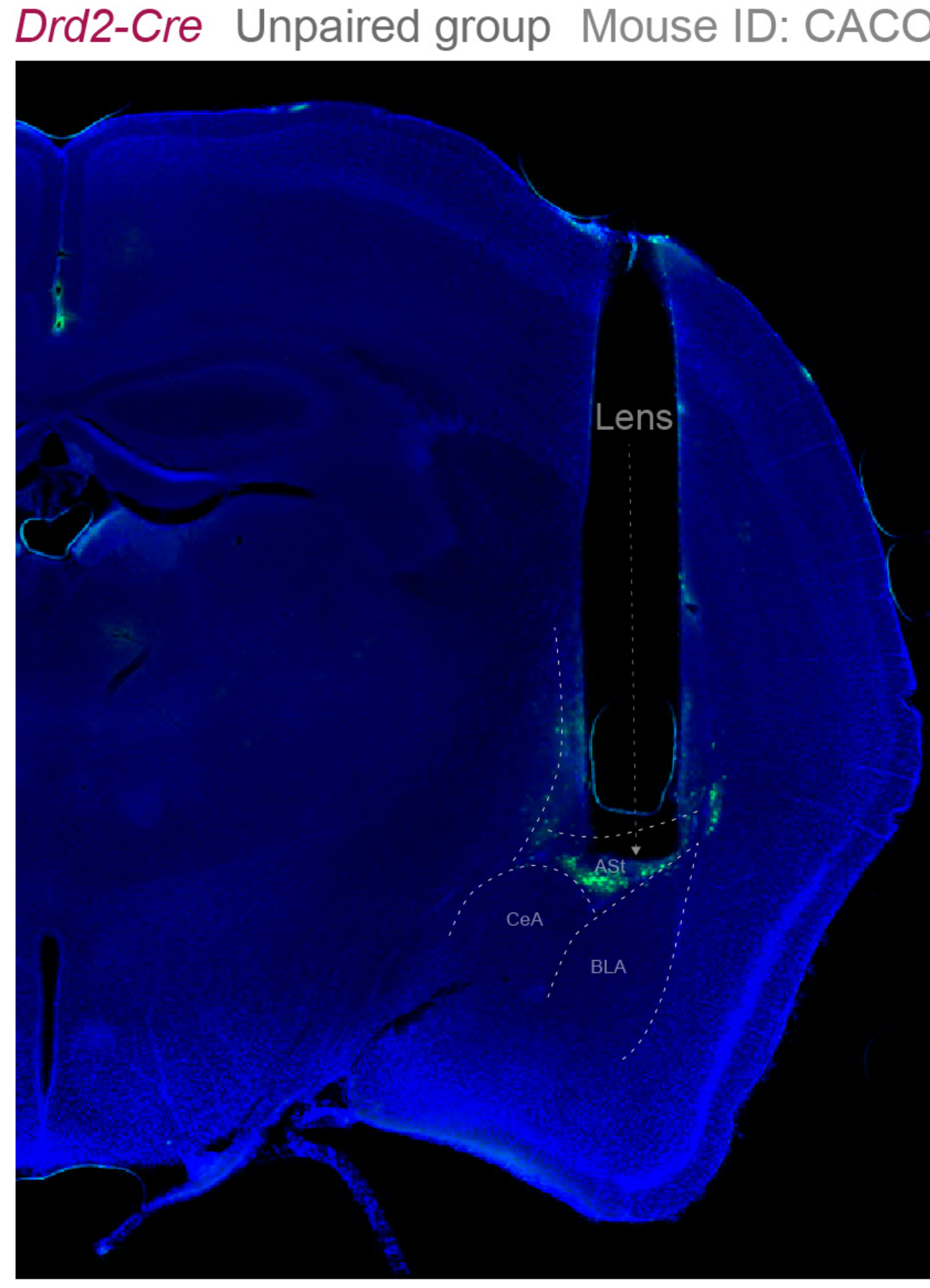

Great lens placement + virus in ASt ✓

Green = GCaMP7f
Blue = DAPI

Mills et al. 2026

Data S1 - Appendix E

Histology for *Drd1a*-Cre and *Drd2*-Cre NpHR mice

Appendix E - Histology - *Drd2:Cre* mice, NpHR experiments :

Drd2:Cre NpHR Mouse ID: C03J376

Data from this mouse were not included in the study

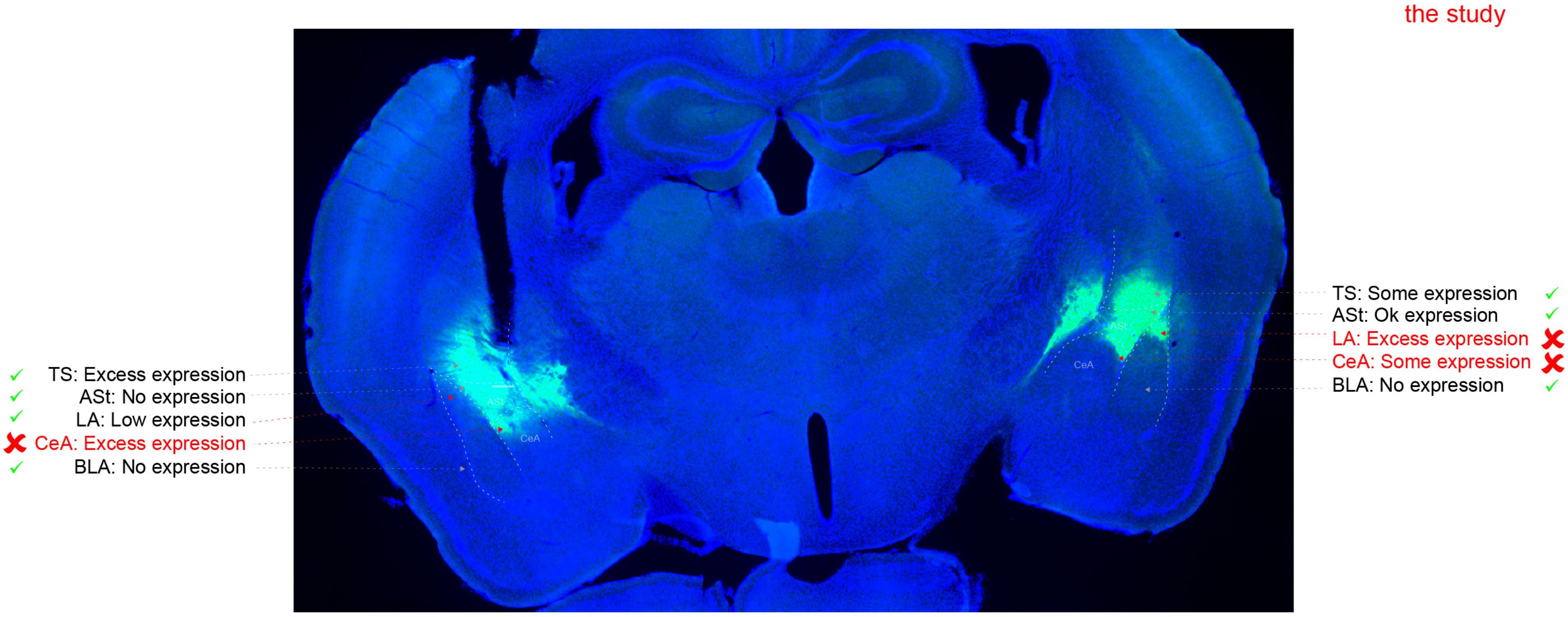

Drd2:Cre NpHR Mouse ID: C03J416

Data from this mouse were not included in the study

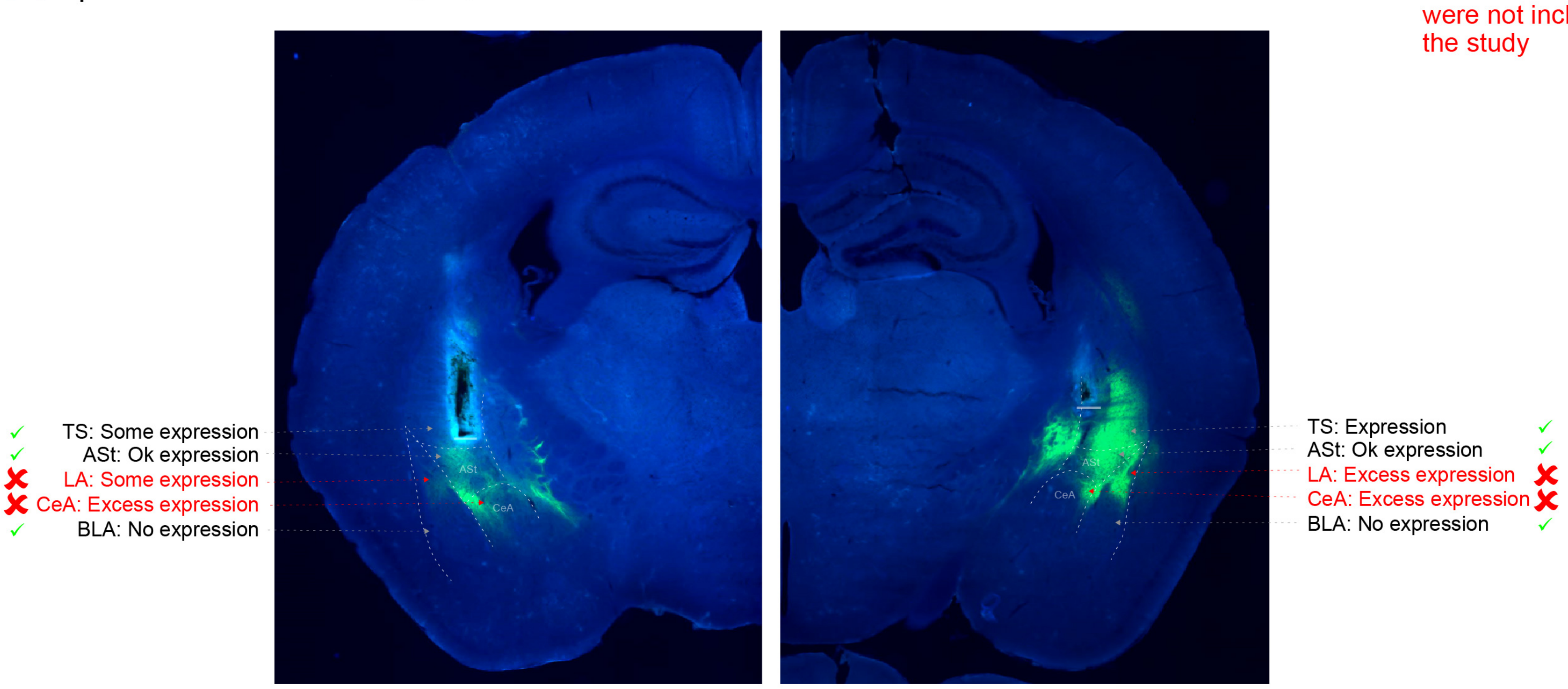

Drd2:Cre NpHR Mouse ID: C04J559

Data from this mouse were not included in the study

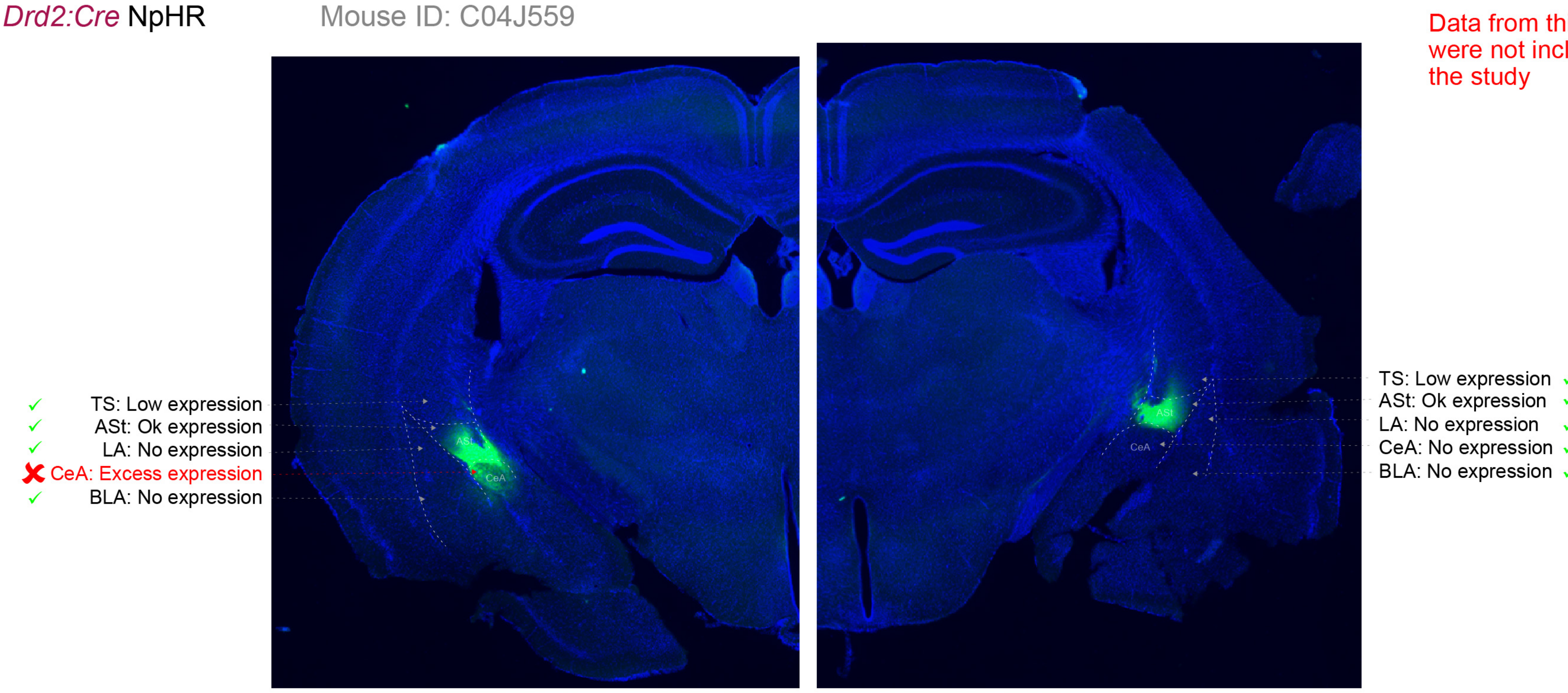

Appendix E - Histology - *Drd2:Cre* mice, NpHR experiments:

Drd2:Cre NpHR

Mouse ID: C025815

Data from this mouse were not included in the study

✓ TS: Low expression
✗ ASt: No expression
✓ LA: No expression
✓ CeA: No expression
✓ BLA: No expression

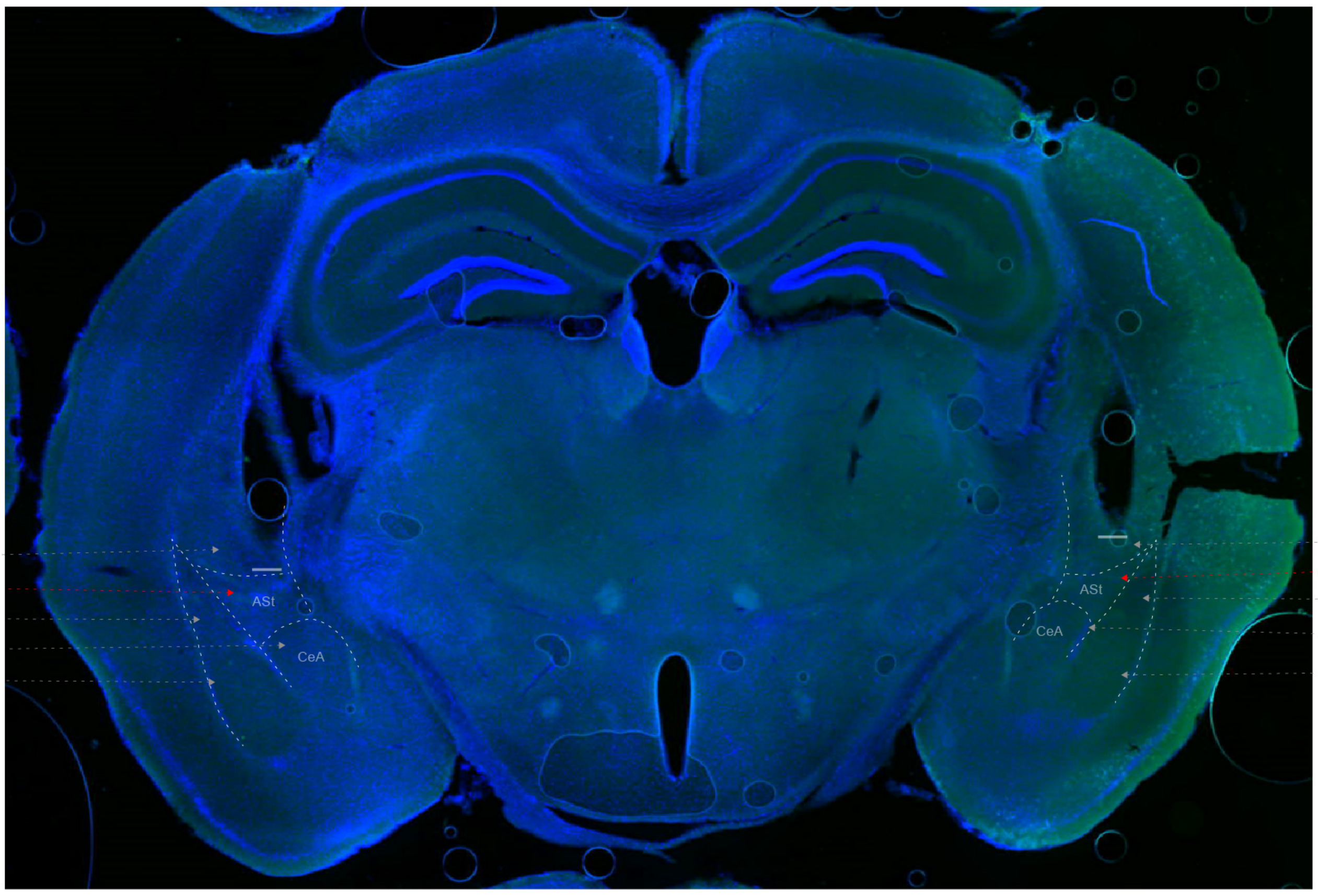

TS: Low expression ✓
ASt: No expression ✗
LA: No expression ✓
CeA: No expression ✓
BLA: No expression ✓

Drd2:Cre NpHR

Mouse ID: C025816

Data from this mouse were not included in the study

✓ TS: Excess expression
✓ ASt: No expression
✗ LA: Excess expression
✓ CeA: No expression
✓ BLA: No expression

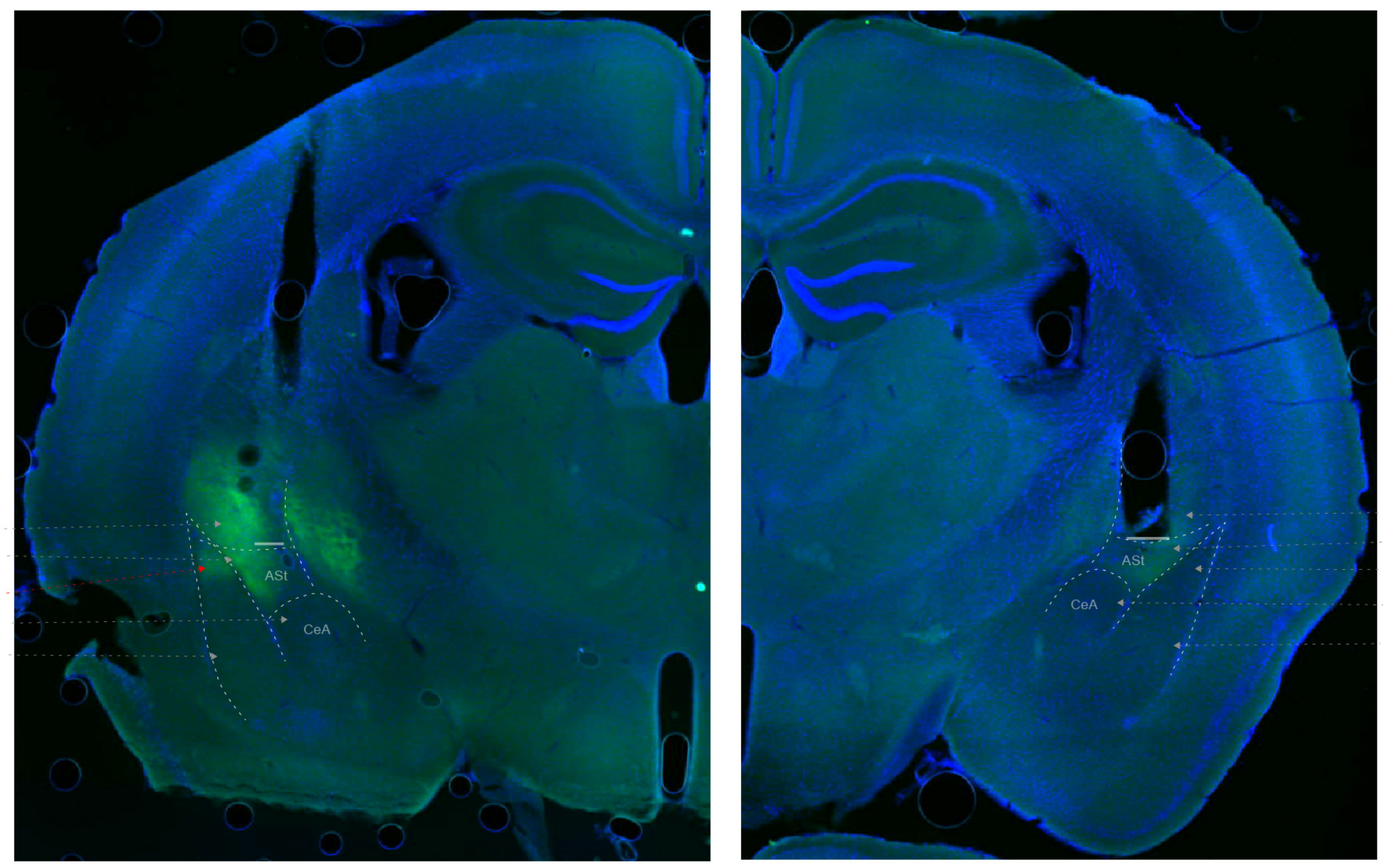

TS: Low expression ✓
ASt: Ok expression ✓
LA: No expression ✓
CeA: No expression ✓
BLA: No expression ✓

Drd2:Cre NpHR

Mouse ID: C03J350

Data from this mouse were not included in the study

✓ TS: Low expression
✓ ASt: Ok expression
✗ LA: Excess expression
✗ CeA: No expression
✓ BLA: Low expression

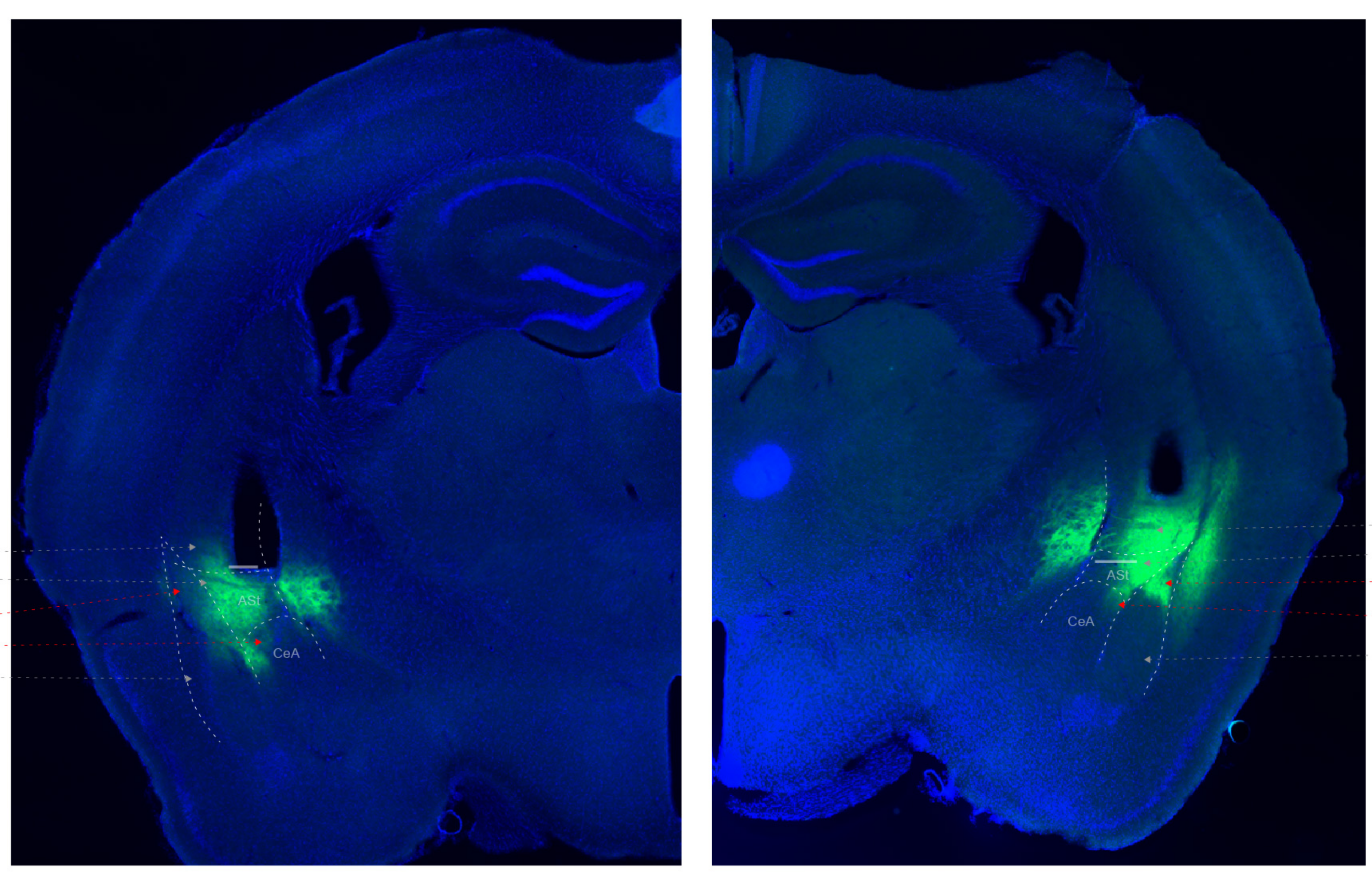

TS: Expression ✓
ASt: Ok expression ✓
LA: Excess expression ✗
CeA: Some expression ✗
BLA: No expression ✓

Appendix E - Histology - *Drd2:Cre* mice, NpHR experiments:

Drd2:Cre NpHR   Mouse ID: C04J564

Data from this mouse were not included in the study

Left:
- ✓ TS: Low expression
- ✗ ASt: Low expression
- ✓ LA: No expression
- ✓ CeA: No expression
- ✓ BLA: No expression

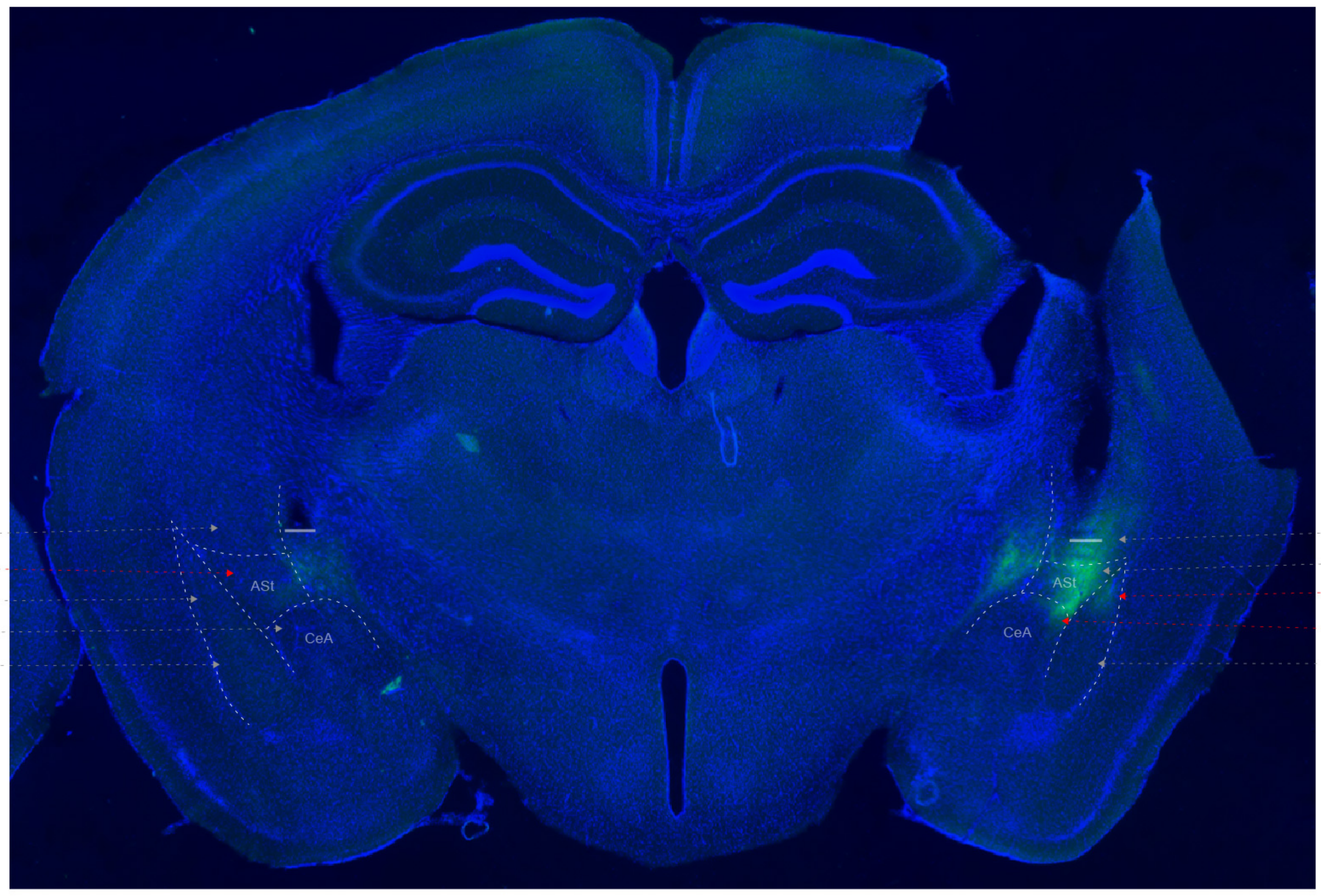

Right:
- TS: Low expression ✓
- ASt: Ok expression ✓
- LA: Some expression ✗
- CeA: Some expression ✗
- BLA: No expression ✓

Drd2:Cre NpHR   Mouse ID: C04J574

Data from this mouse were not included in the study

Left:
- ✓ TS: Low expression
- ✓ ASt: Ok expression
- ✓ LA: No expression
- ✗ CeA: Excess expression
- ✓ BLA: No expression

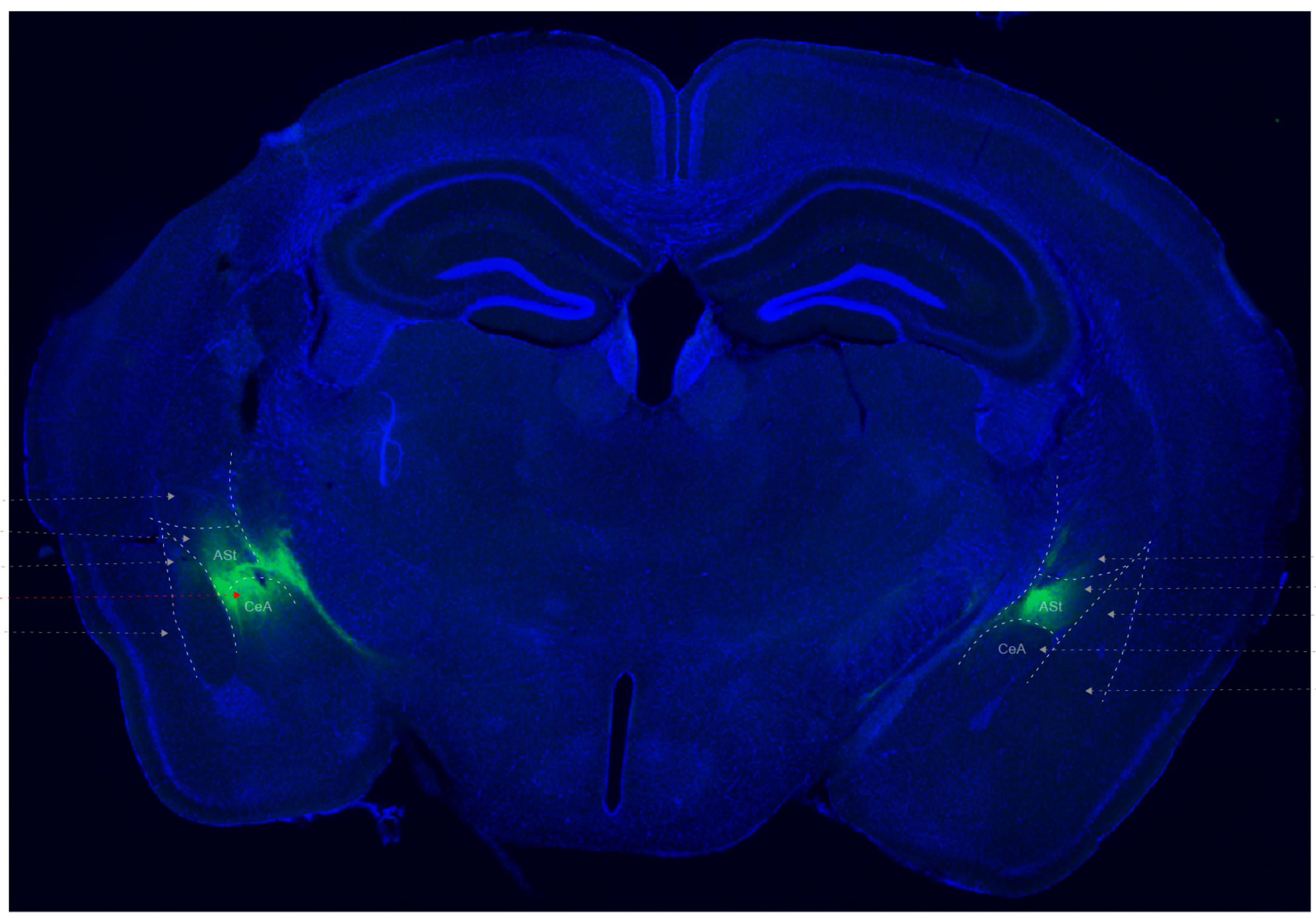

Right:
- TS: Low expression ✓
- ASt: Ok expression ✓
- LA: No expression ✓
- CeA: No expression ✓
- BLA: No expression ✓

Drd2:Cre NpHR   Mouse ID: C04J556

Data from this mouse were not included in the study

Left:
- ✓ TS: Low expression
- ✓ ASt: Ok expression
- ✓ LA: No expression
- ✗ CeA: Some expression
- ✓ BLA: No expression

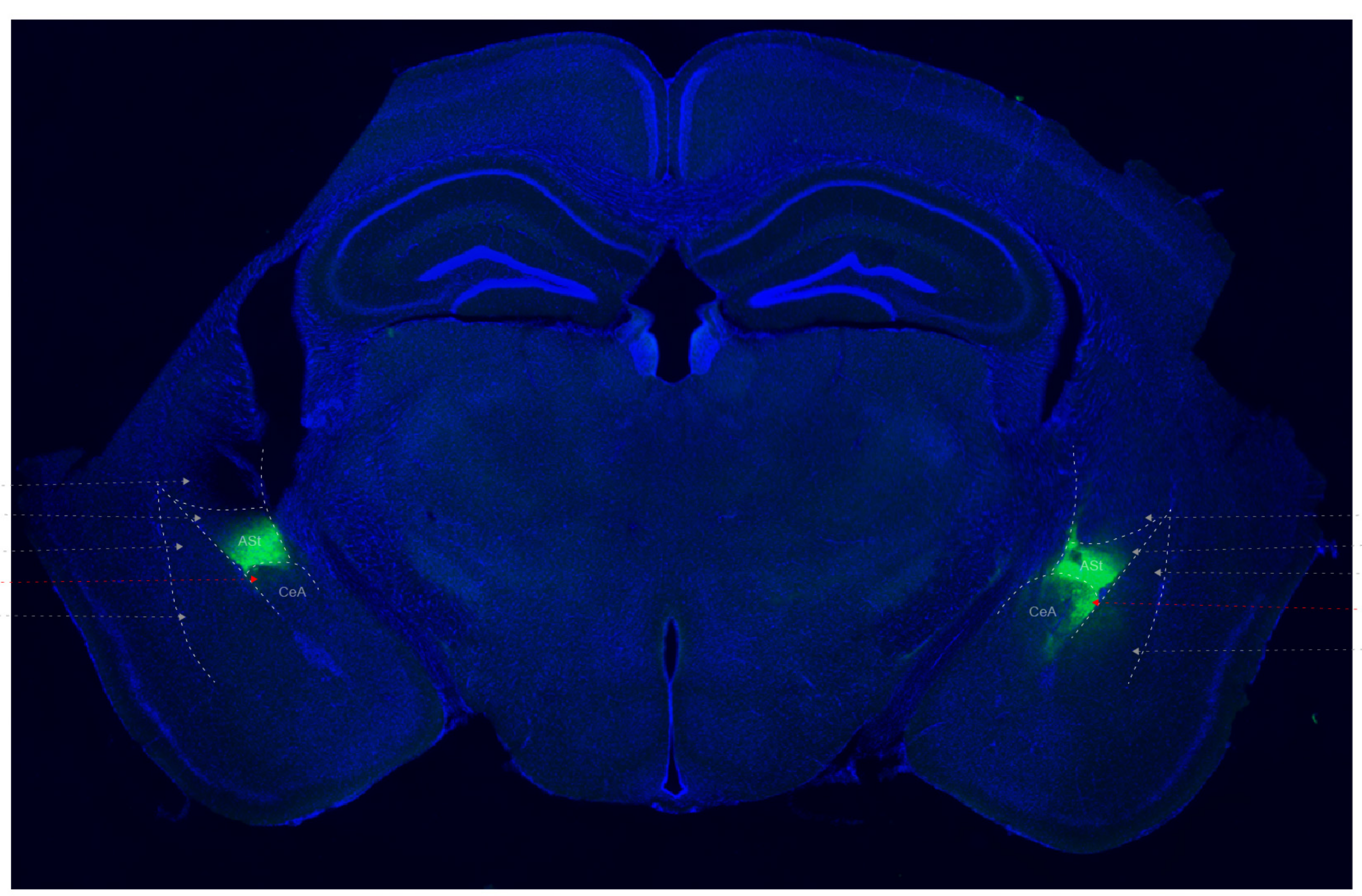

Right:
- TS: Low expression ✓
- ASt: No expression ✓
- LA: No expression ✓
- CeA: Excess expression ✗
- BLA: No expression ✓

Appendix E - Histology - *Drd2:Cre* mice, NpHR experiments:

Drd2:Cre NpHR Mouse ID: C01I2

Data from this mouse were not included in the study

✓ TS: Low expression
✗ ASt: No expression
✓ LA: No expression
✓ CeA: No expression
✓ BLA: No expression

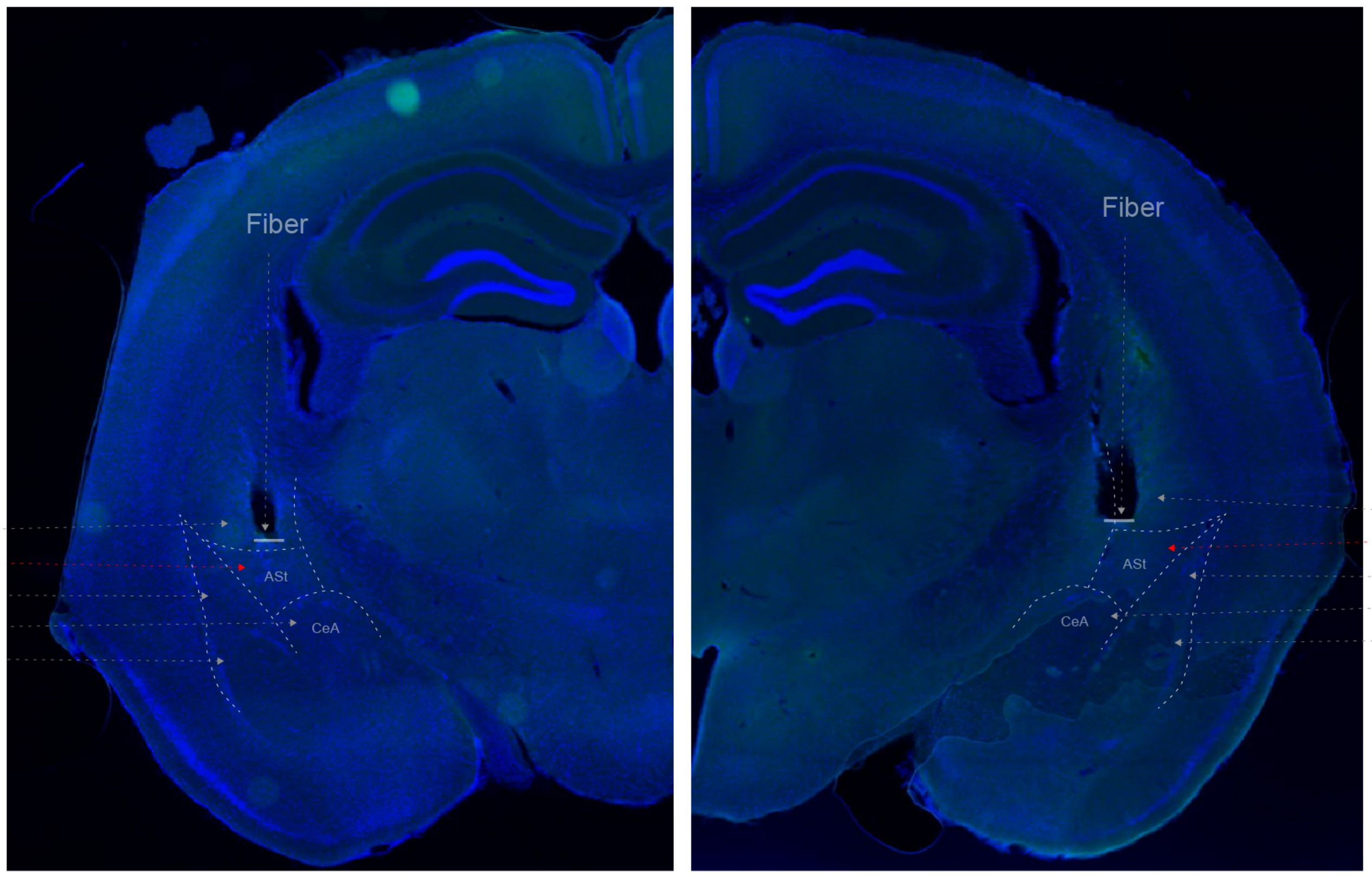

TS: Low expression ✓
ASt: No expression ✗
LA: No expression ✓
CeA: No expression ✓
BLA: No expression ✓

Drd2:Cre NpHR Mouse ID: C025807

Data from this mouse were not included in the study

✓ TS: Low expression
✓ ASt: Ok expression
✓ LA: No expression
✓ CeA: No expression
✓ BLA: No expression

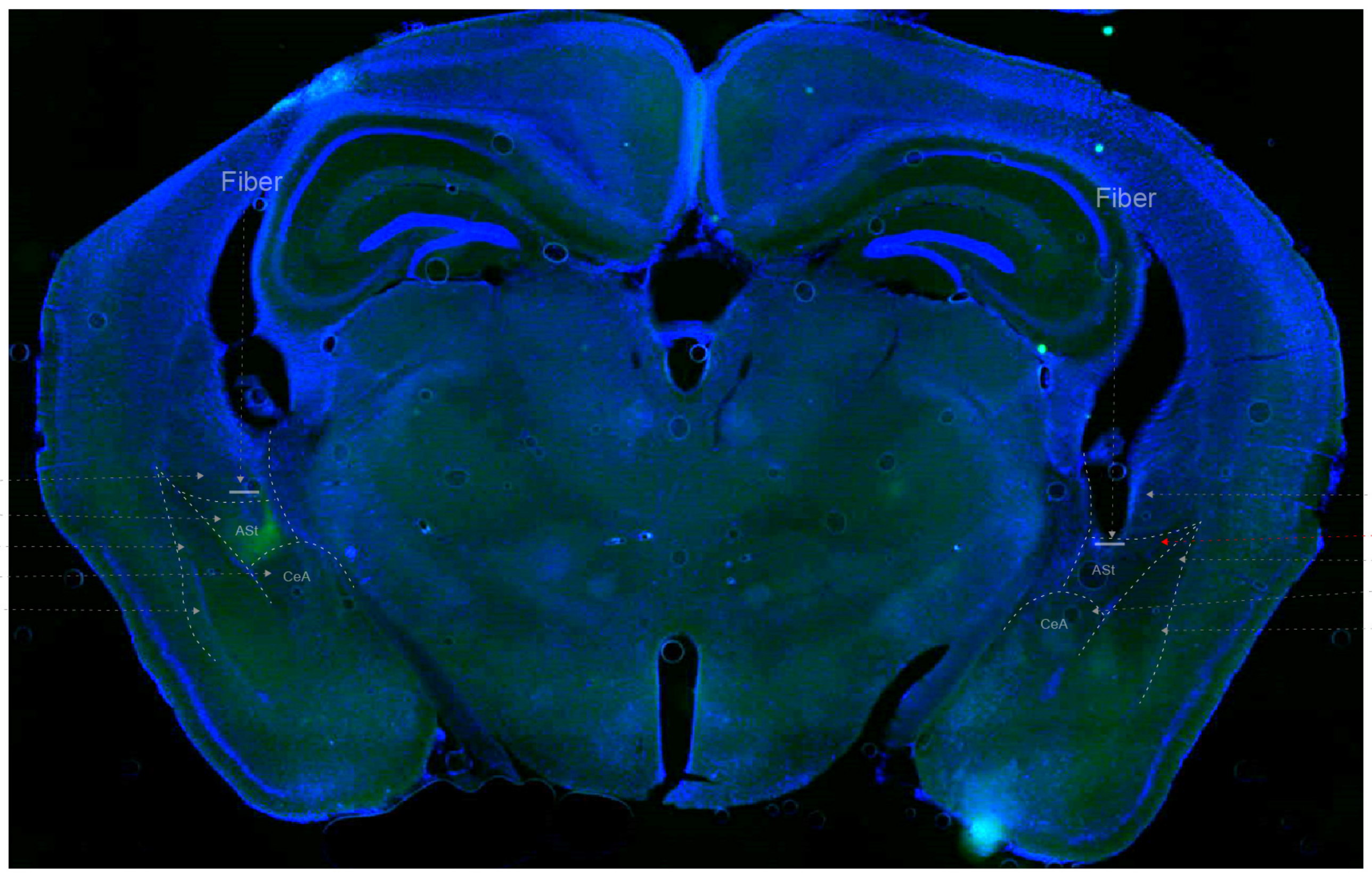

TS: Low expression ✓
ASt: No expression ✗
LA: No expression ✓
CeA: No expression ✓
BLA: No expression ✓

Drd2:Cre NpHR Mouse ID: C025808

Data from this mouse were not included in the study

✓ TS: Low expression
✗ ASt: No expression
✓ LA: No expression
✓ CeA: No expression
✓ BLA: No expression

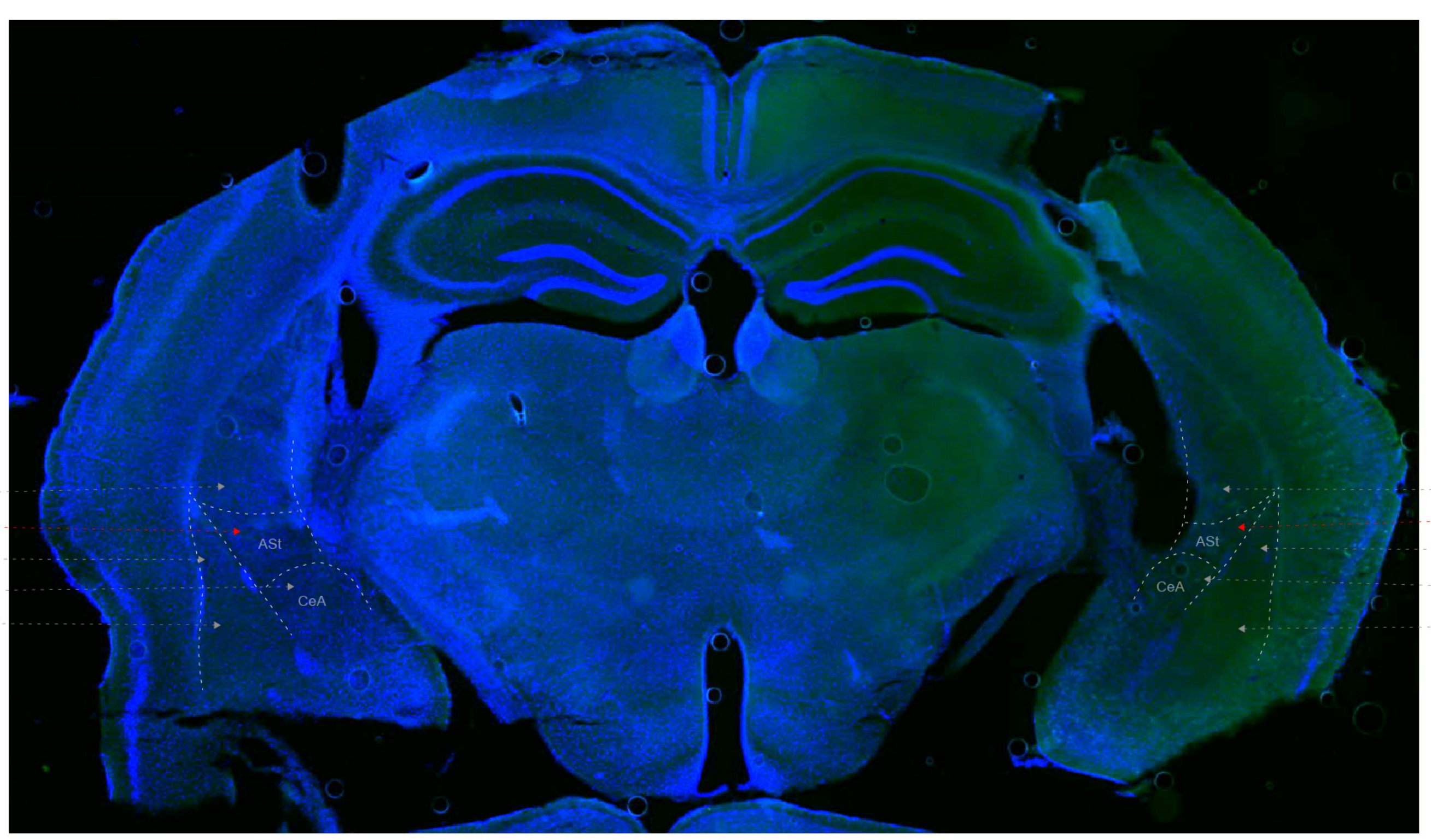

TS: Low expression ✓
ASt: No expression ✗
LA: No expression ✓
CeA: No expression ✓
BLA: No expression ✓

Appendix E - Histology - *Drd2:Cre* mice, NpHR experiments:

Drd2:Cre NpHR Mouse ID: C025809

Histology okay, Mouse included

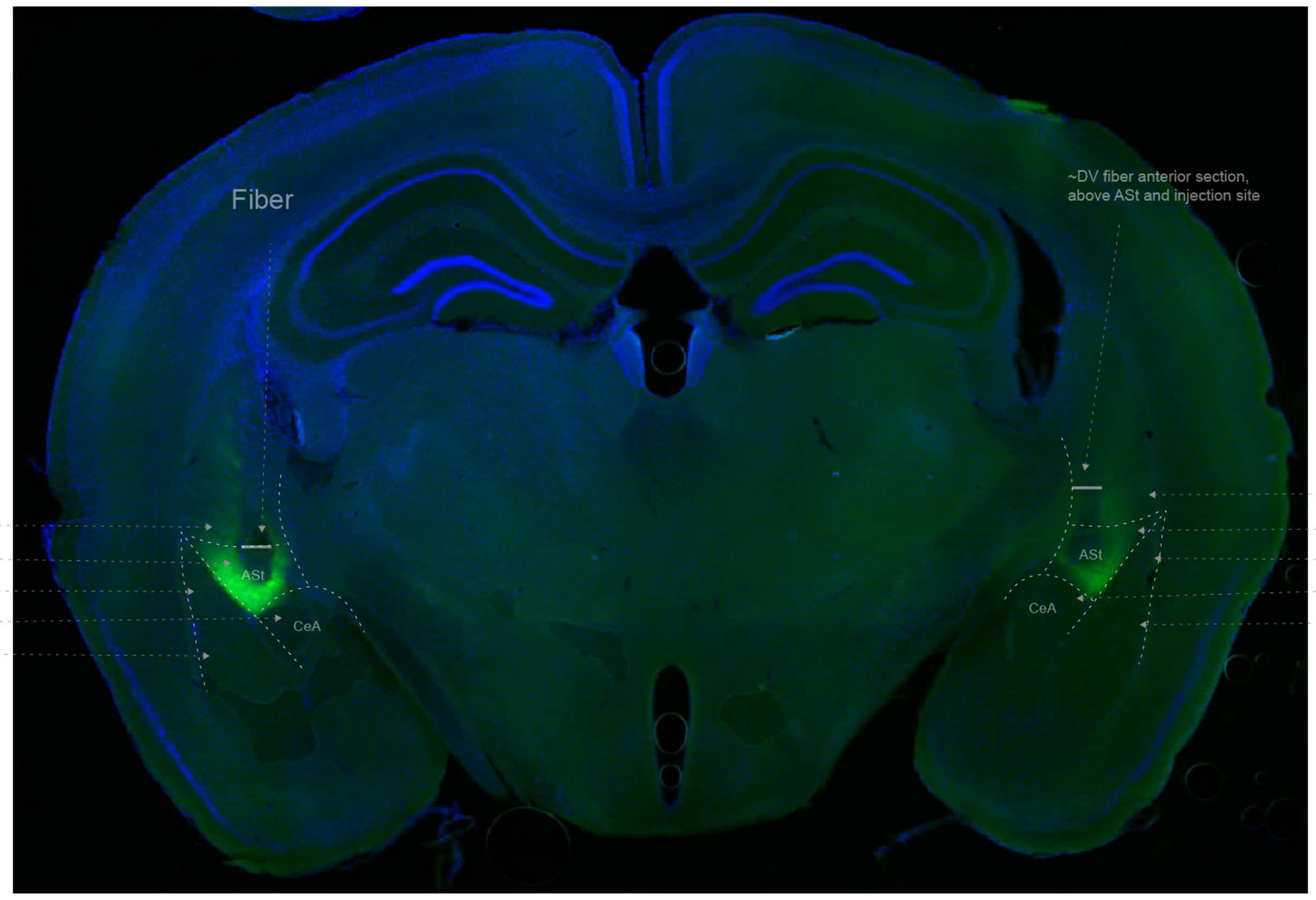

Left:
- ✓ TS: Low expression
- ✓ ASt: Good expression
- ✓ LA: No expression
- ✓ CeA: No expression
- ✓ BLA: No expression

Right:
- TS: Minimal expression ✓
- ASt: Good expression ✓
- LA: No expression ✓
- CeA: No expression ✓
- BLA: No expression ✓

Drd2:Cre NpHR Mouse ID: C025817

Histology okay, Mouse included

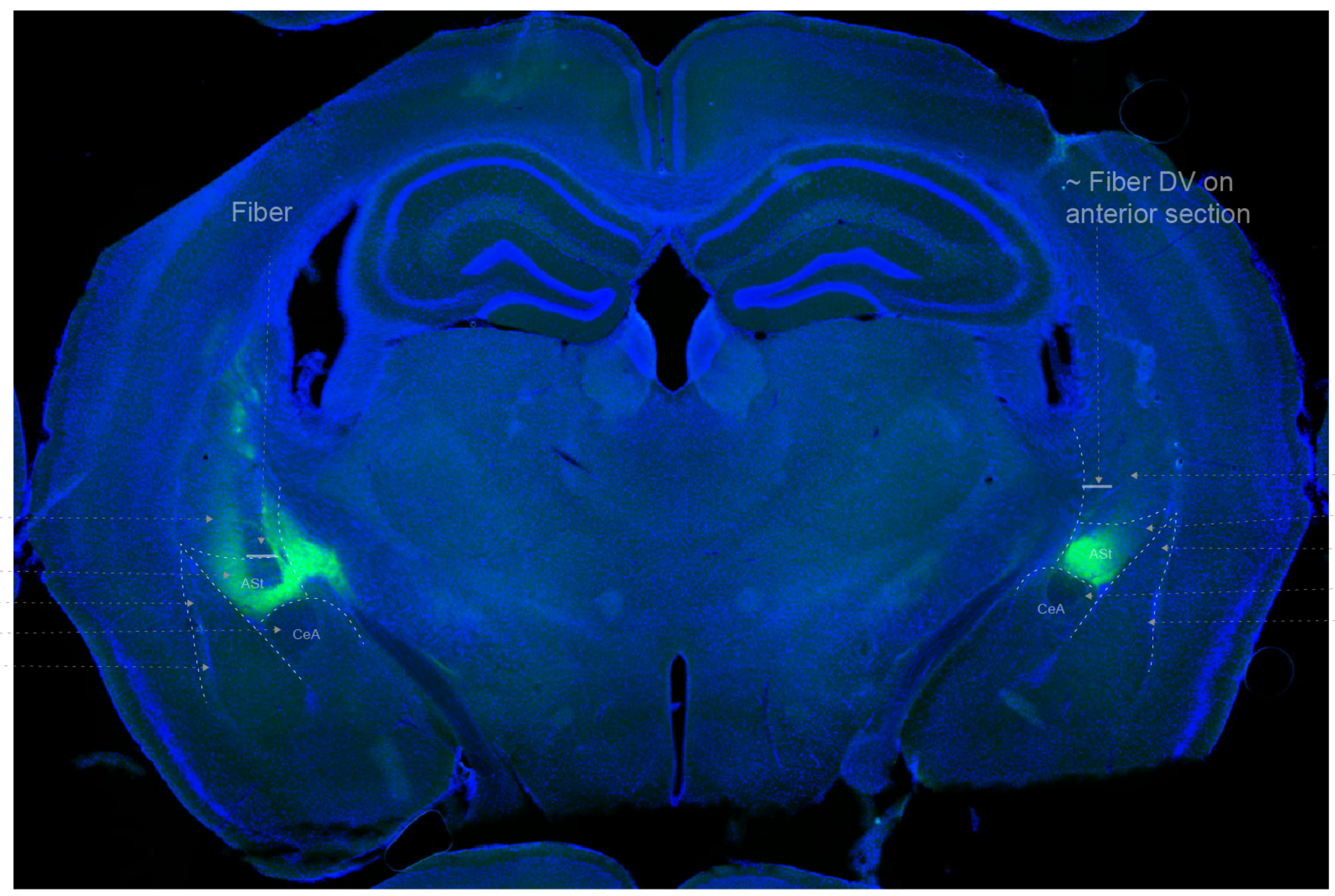

Left:
- ✓ TS: Some expression
- ✓ ASt: Good expression
- ✓ LA: No expression
- ✓ CeA: No expression
- ✓ BLA: No expression

Right:
- TS: Minimal expression ✓
- ASt: Good expression ✓
- LA: No expression ✓
- CeA: No expression ✓
- BLA: No expression ✓

Drd2:Cre NpHR Mouse ID: C025810

Histology okay, Mouse included

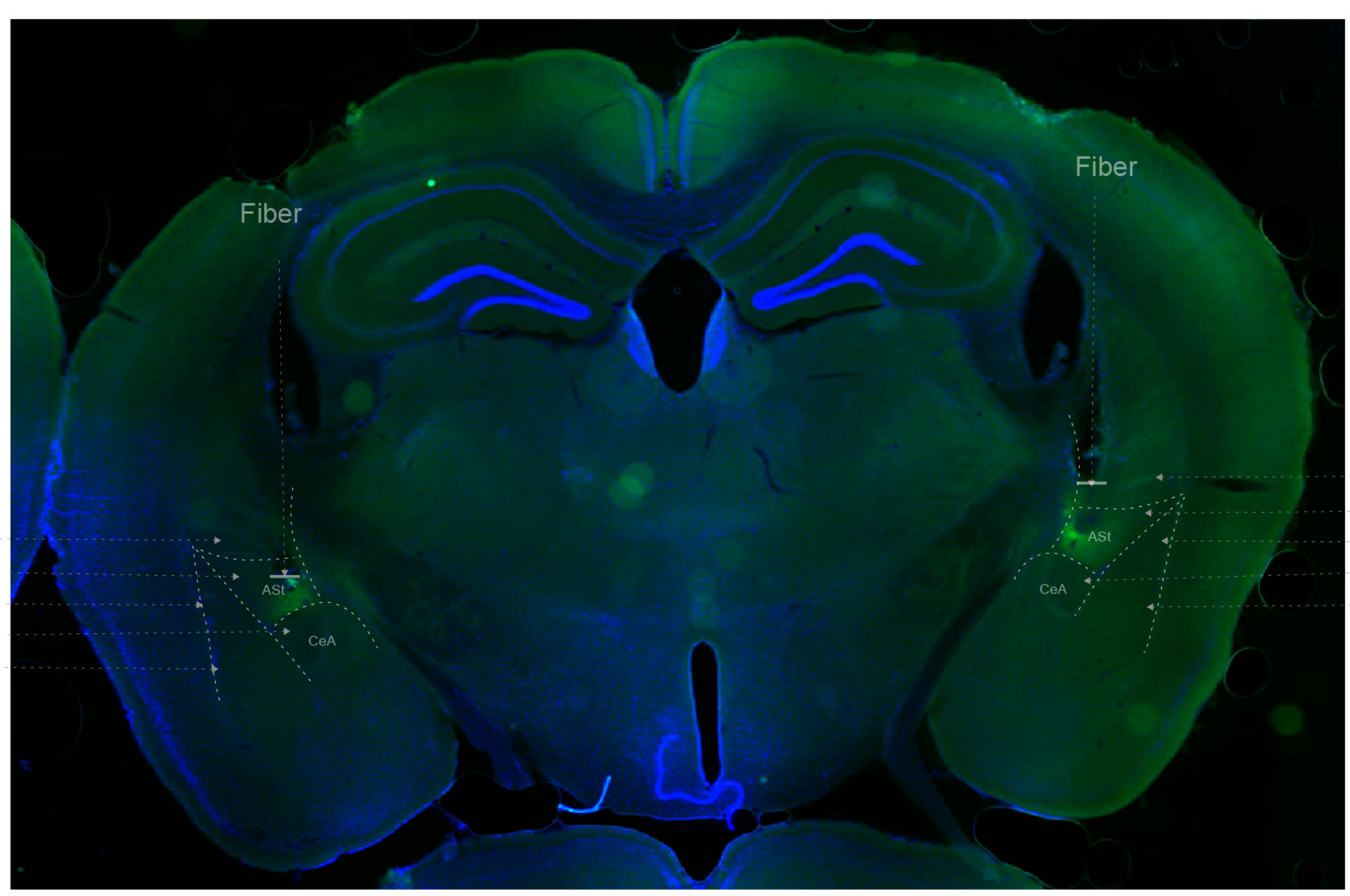

Left:
- ✓ TS: Low expression
- ✓ ASt: Good expression
- ✓ LA: No expression
- ✓ CeA: No expression
- ✓ BLA: No expression

Right:
- TS: Minimal expression ✓
- ASt: Good expression ✓
- LA: No expression ✓
- CeA: No expression ✓
- BLA: No expression ✓

Appendix E - *Drd2:Cre* mice, NpHR experiments:

Drd2:Cre NpHR

Mouse ID: C04J561

Histology okay, Mouse included

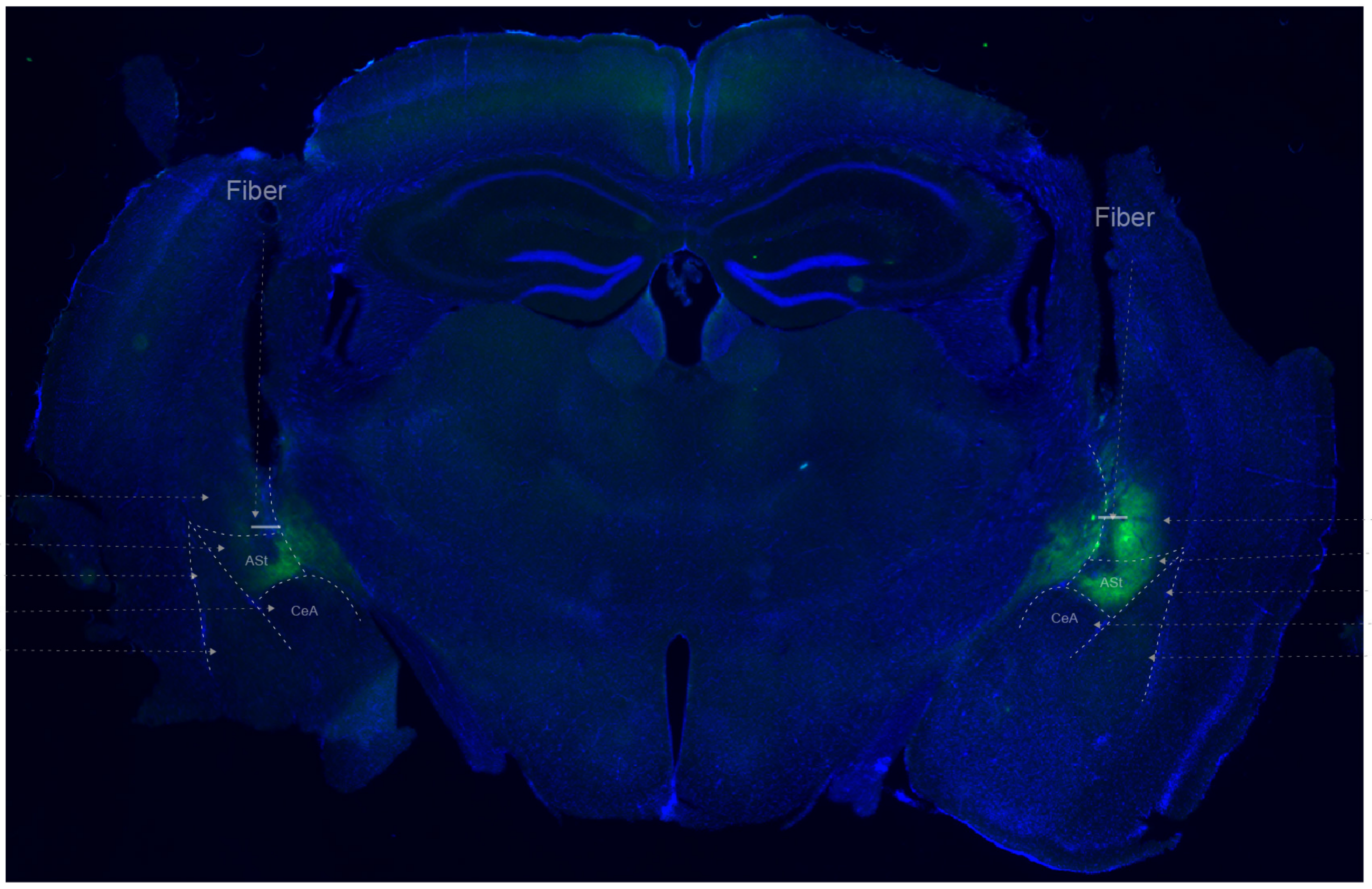

✓ TS: Low expression
✓ ASt: Ok expression
✓ LA: No expression
✓ CeA: No expression
✓ BLA: No expression

TS: Some ventral expression ✓
ASt: Ok expression ✓
LA: No expression ✓
CeA: No expression ✓
BLA: No expression ✓

Drd2:Cre NpHR

Mouse ID: C04J571

Histology okay, Mouse included

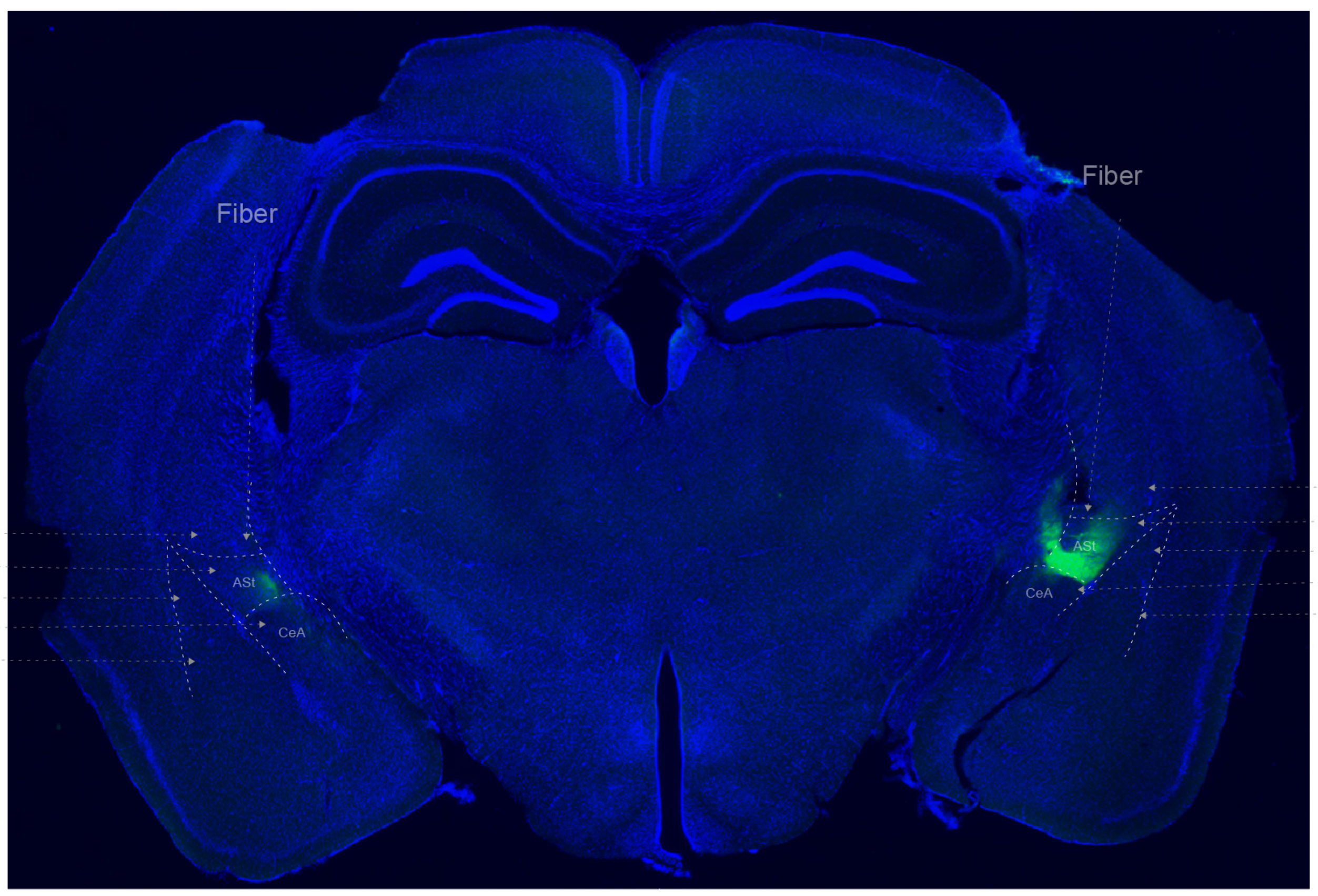

✓ TS: Low expression
✓ ASt: Good expression
✓ LA: No expression
✓ CeA: No expression
✓ BLA: No expression

TS: Minimal expression ✓
ASt: Good expression ✓
LA: No expression ✓
CeA: No expression ✓
BLA: No expression ✓

Drd2:Cre NpHR

Mouse ID: C01I4

Histology okay, Mouse included

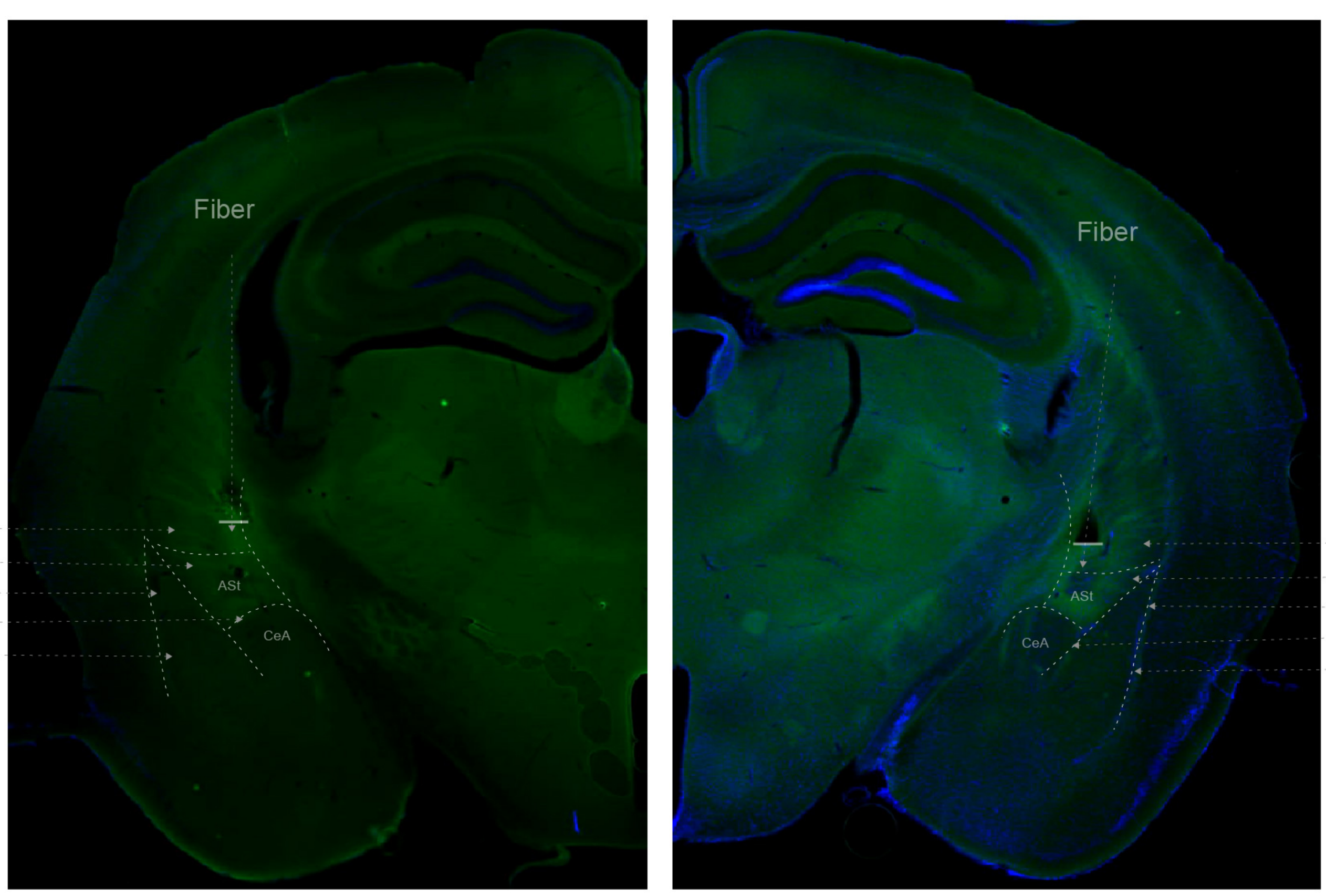

✓ TS: Some expression
✓ ASt: Ok expression
✓ LA: No expression
✓ CeA: No expression
✓ BLA: No expression

TS: Some expression ✓
ASt: Good expression ✓
LA: No expression ✓
CeA: No expression ✓
BLA: No expression ✓

Appendix E - Histology - *Drd2:Cre* mice, NpHR experiments:

Drd2:Cre NpHR

Mouse ID: C01I1

Histology okay, Mouse included

✓ TS: No expression
✓ ASt: Okay expression
✓ LA: No expression
✓ CeA: No expression
✓ BLA: No expression

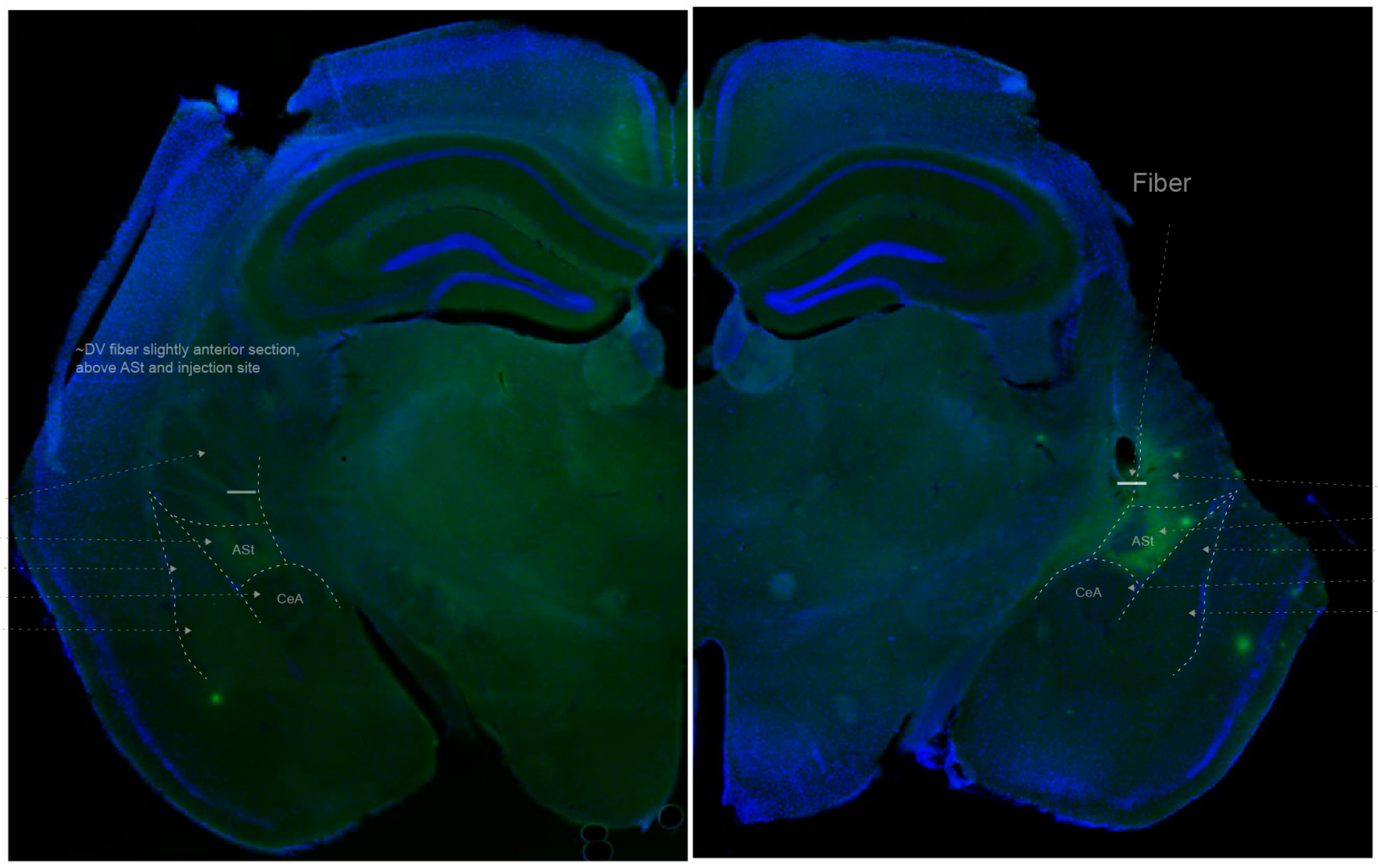

TS: Some expression ✓
ASt: Great expression ✓
LA: No expression ✓
CeA: No expression ✓
BLA: No expression ✓

Drd2:Cre NpHR

Mouse ID: C01I3

Histology okay, Mouse included

✓ TS: Low expression
✓ ASt: Good expression
✓ LA: No expression
✓ CeA: No expression
✓ BLA: No expression

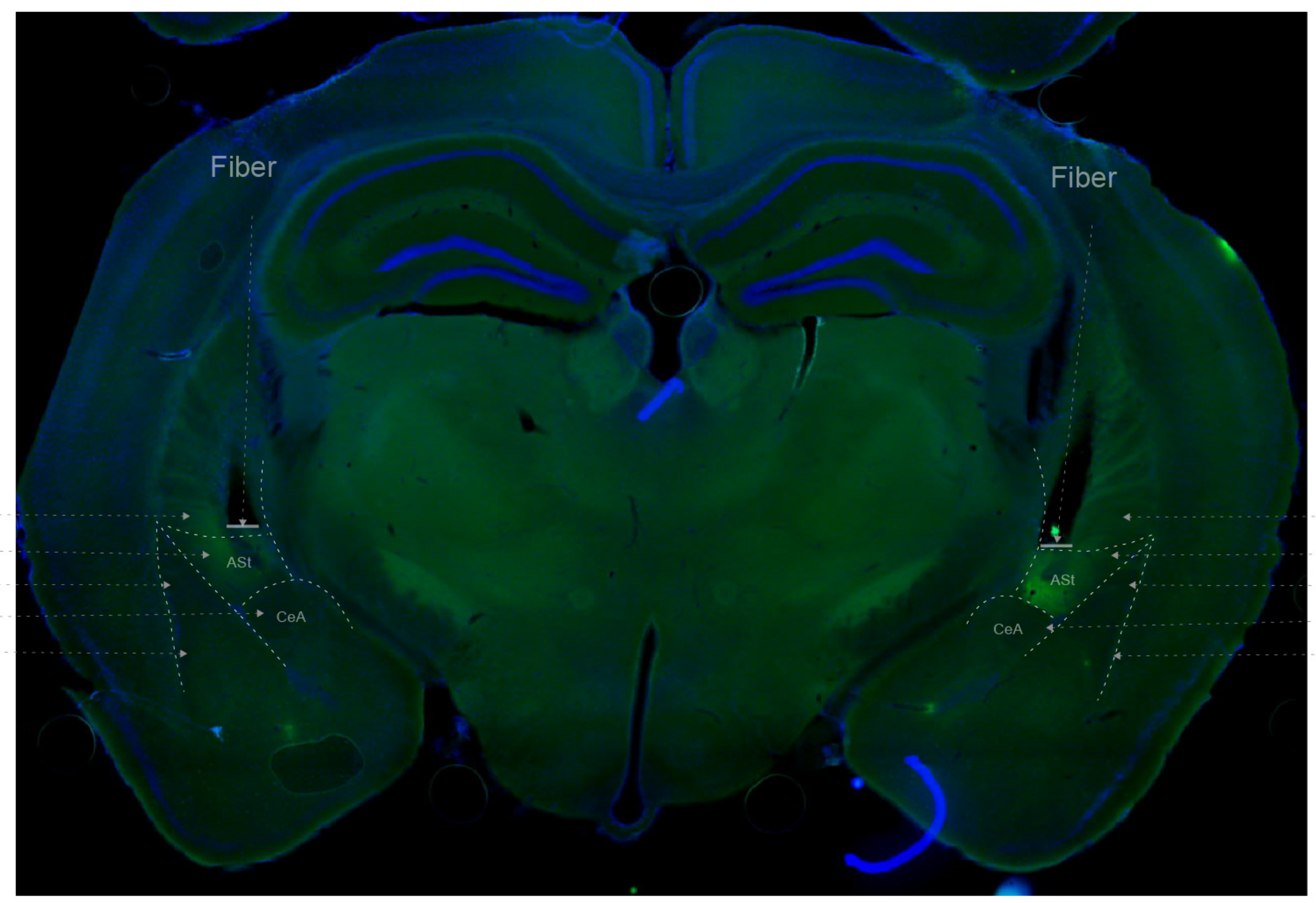

TS: Minimal expression ✓
ASt: Good expression ✓
LA: No expression ✓
CeA: No expression ✓
BLA: No expression ✓

Appendix E - Histology - *Drd1a:Cre* mice, NpHR experiments:

Drd1a:Cre NpHR

Mouse ID: C05K178

Data from this mouse were not included in the study

TS: No expression
ASt: Good expression
LA: No expression
✘ CeA: Expression
BLA: No expression

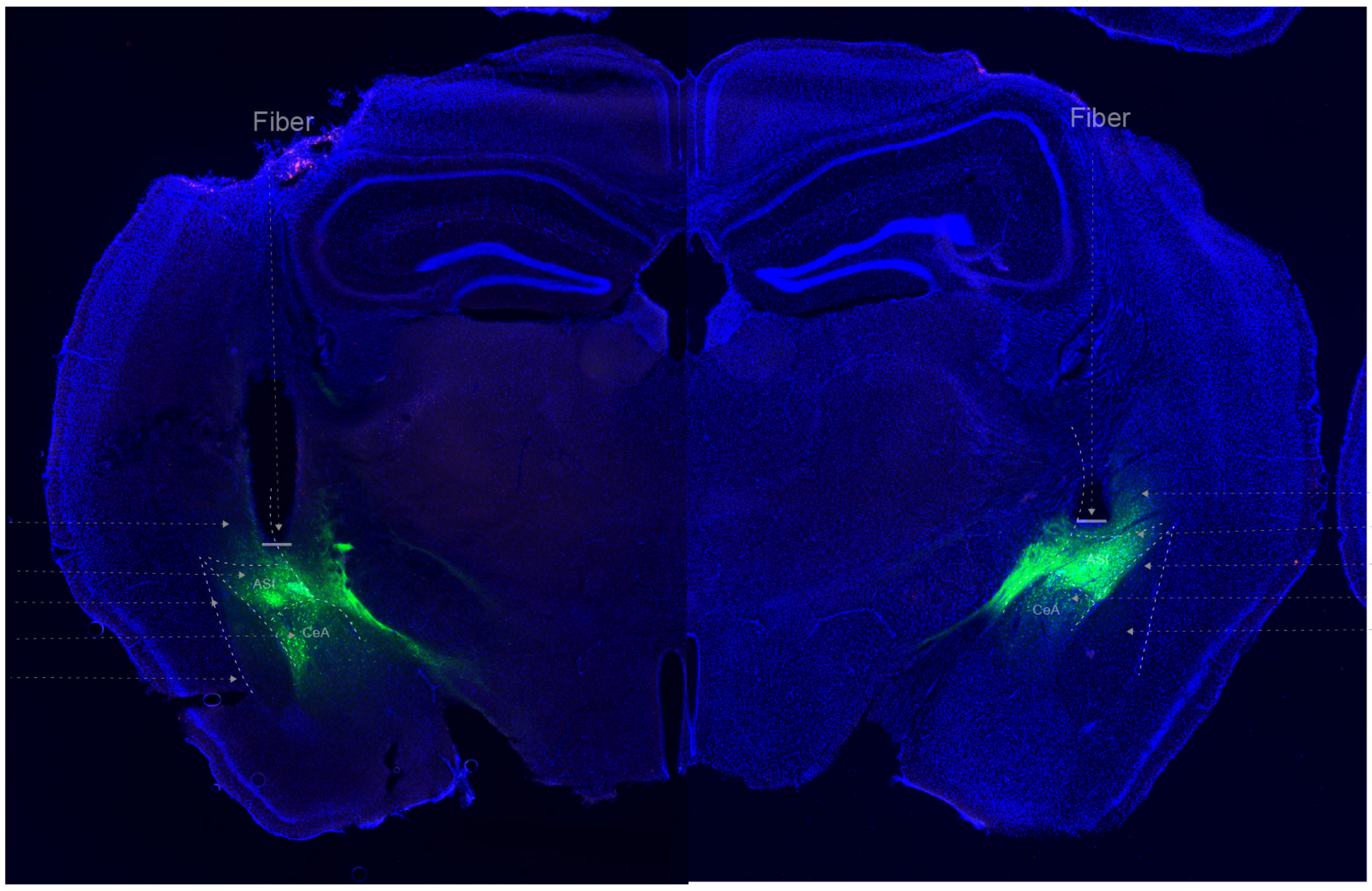

TS: Low expression
ASt: Good expression
LA: No expression
CeA: Expression ✘
BLA: No expression

Drd1a:Cre NpHR

Mouse ID: C01I7

Data from this mouse were not included in the study

Expression slightly too dorsal

✓ TS: Low expression
✓ ASt: Low expression
✓ LA: No expression
✓ CeA: No expression
✓ BLA: No expression

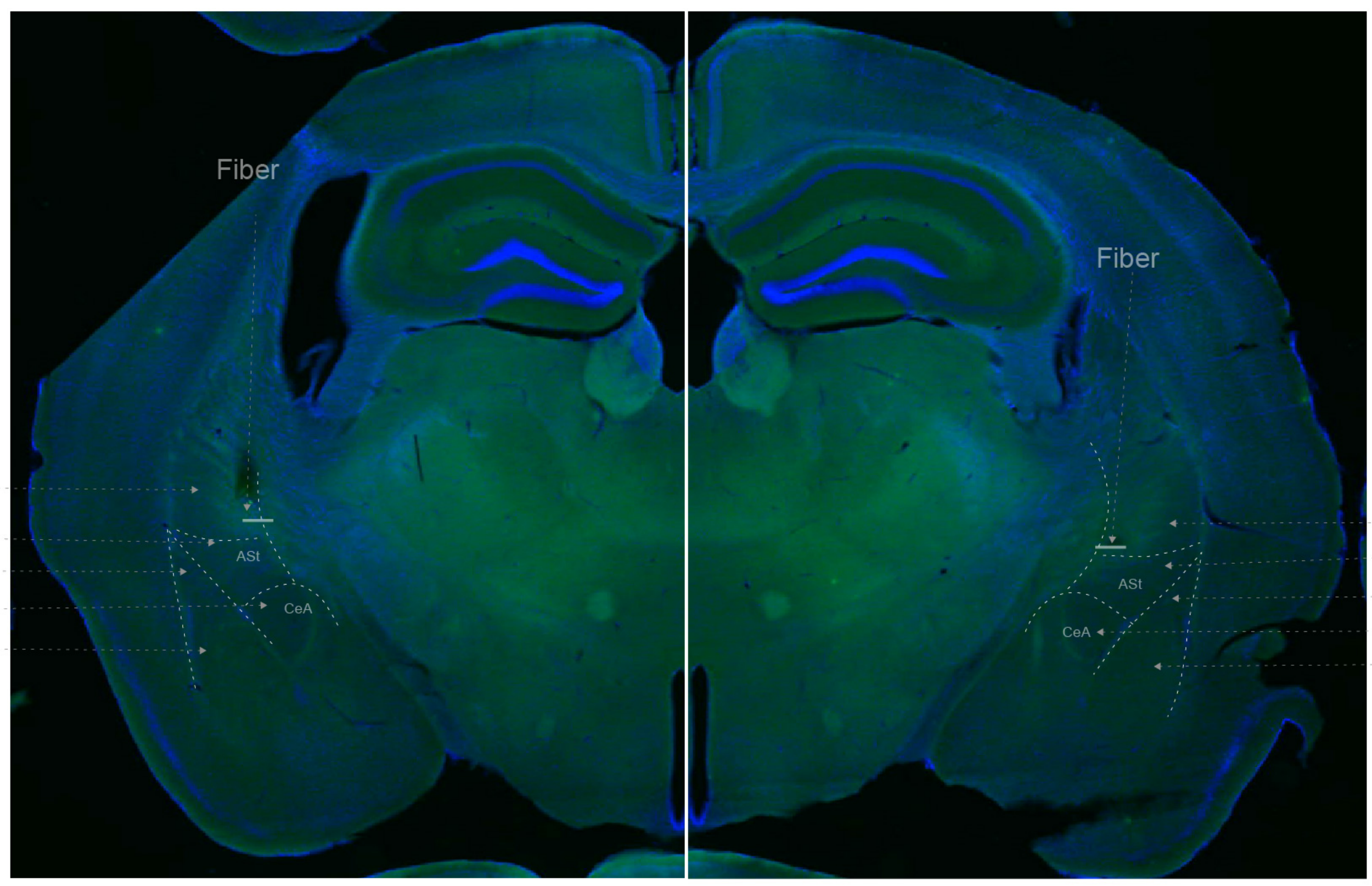

Expression slightly too dorsal

TS: Some lateral expression ✓
ASt: low expression ✓
LA: No expression ✓
CeA: No expression ✓
BLA: No expression ✓

Drd1a:Cre NpHR

Mouse ID: C036672

Data from this mouse were not included in the study

✓ TS: No expression
✘ ASt: No expression
✓ LA: No expression
✓ CeA: No expression
✓ BLA: No expression

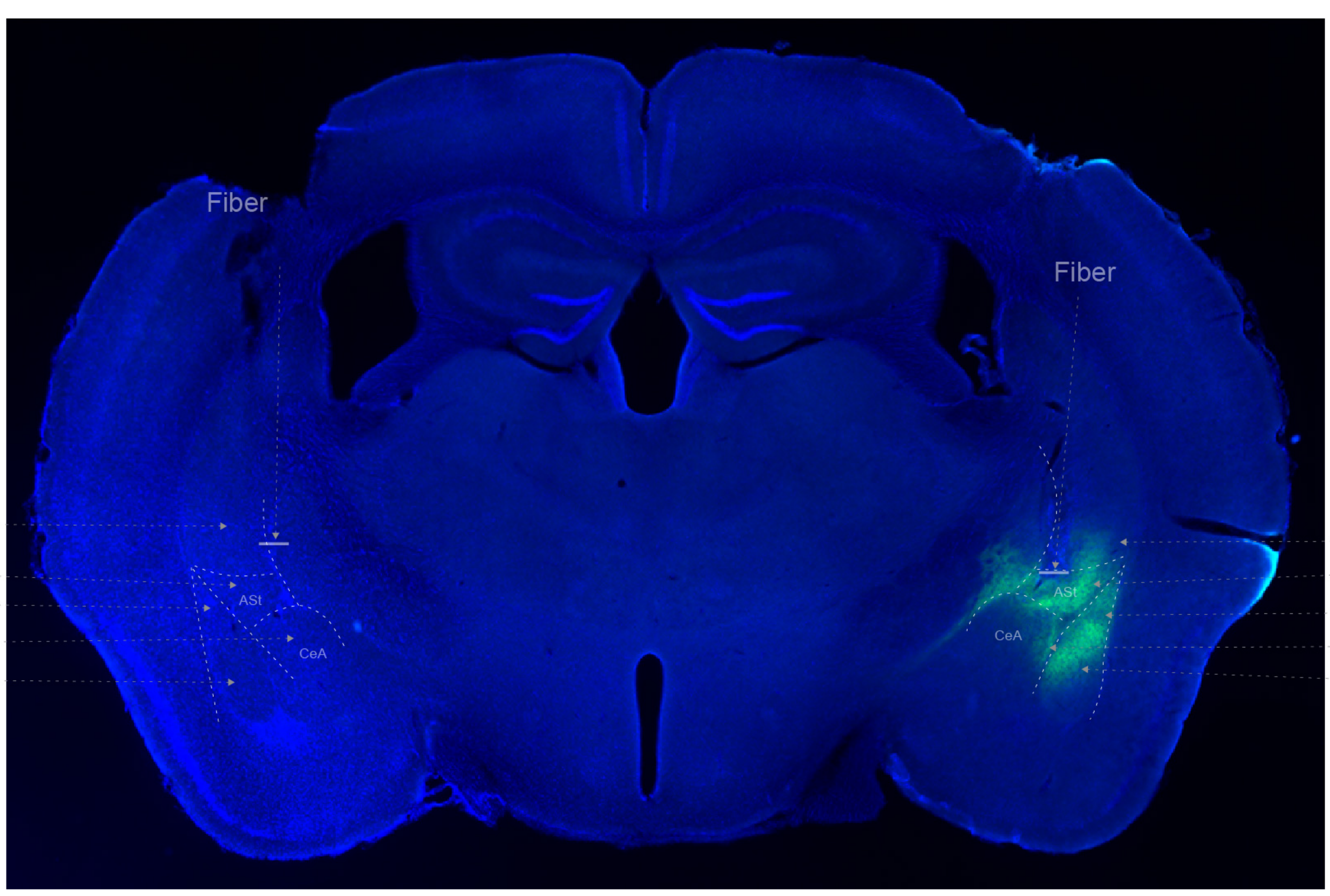

TS: Some expression ✓
ASt: Good expression ✓
LA: No expression ✓
CeA: Expression ✘
BLA: Expression ✓

Appendix E - Histology - *Drd1a:Cre* mice, NpHR experiments:

Drd1a:Cre NpHR

Mouse ID: C05K167

Data from this mouse were not included in the study

TS: No expression
✗ ASt: No expression
LA: No expression
CeA: No expression
BLA: No expression

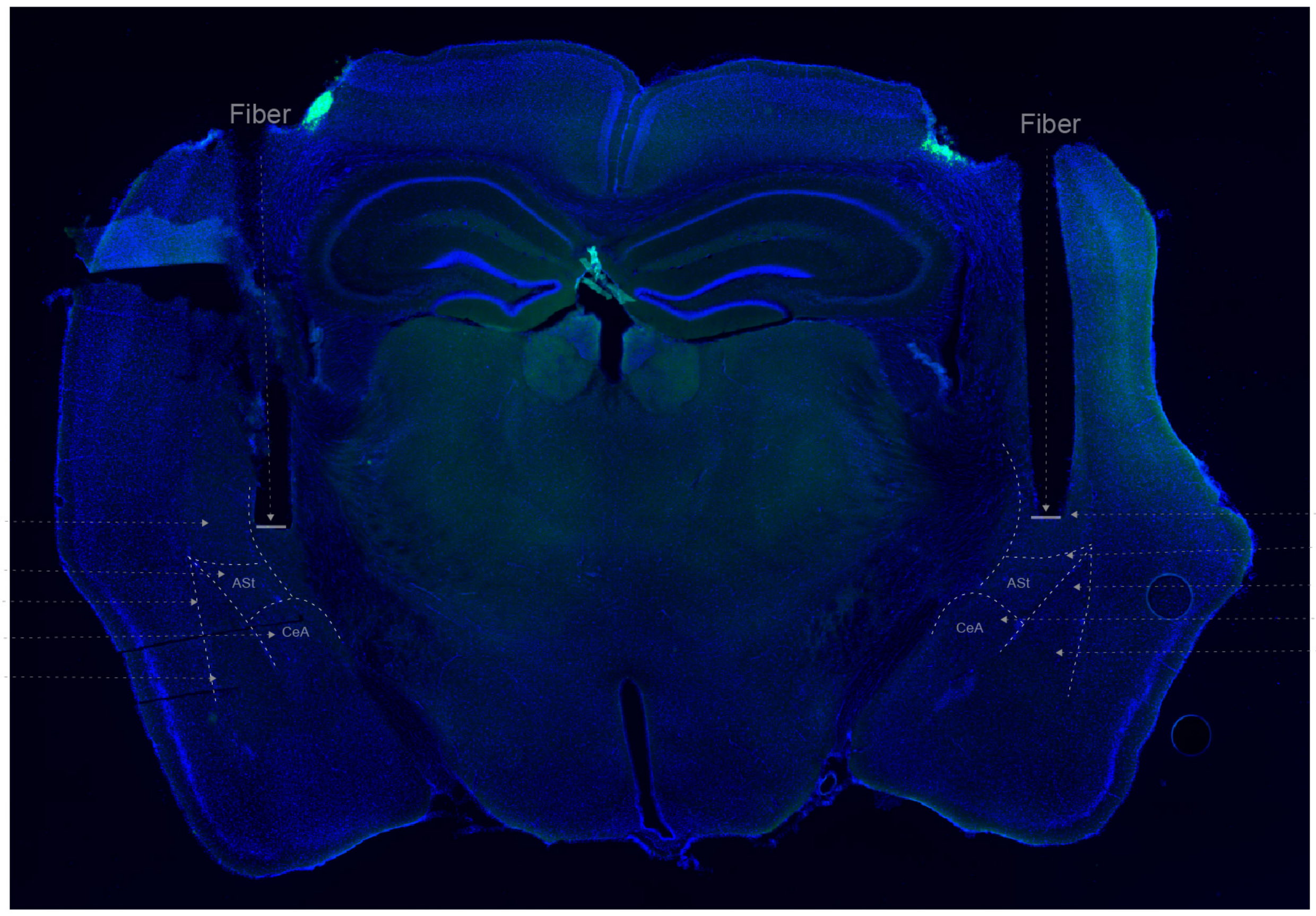

TS: No expression
ASt: No expression ✗
LA: No expression
CeA: No expression
BLA: No expression

Drd1a:Cre NpHR

Mouse ID: C05K151

Data from this mouse were not included in the study

TS: No expression
✗ ASt: No expression
LA: No expression
CeA: No expression
BLA: No expression

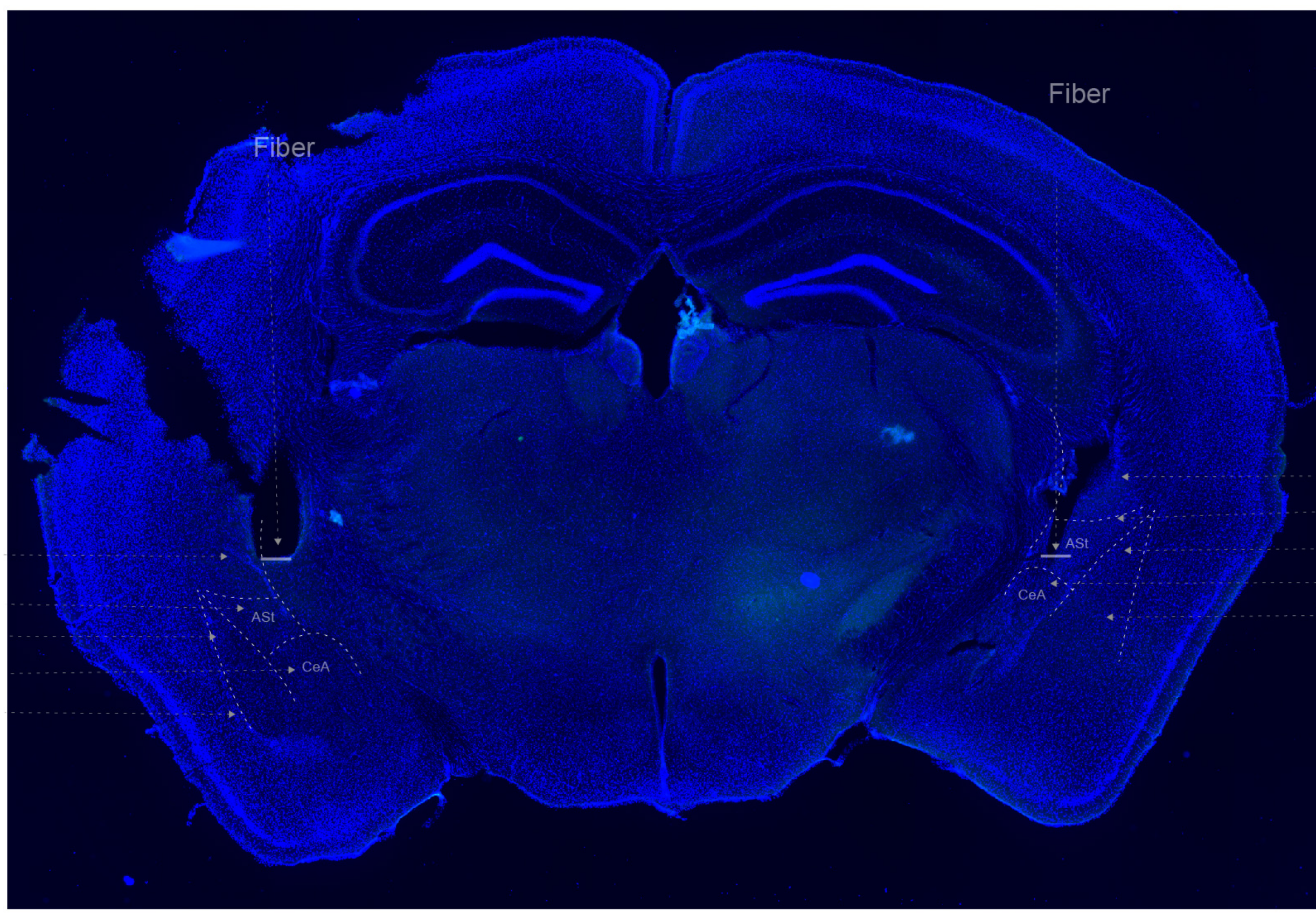

TS: No expression
ASt: No expression ✗
LA: No expression
CeA: Expression
BLA: No expression

Drd1a:Cre NpHR

Mouse ID: C01I6

Data from this mouse were not included in the study

✓ TS: Low expression
✓ ASt: Low expression
✓ LA: No expression
✓ CeA: No expression
✓ BLA: No expression

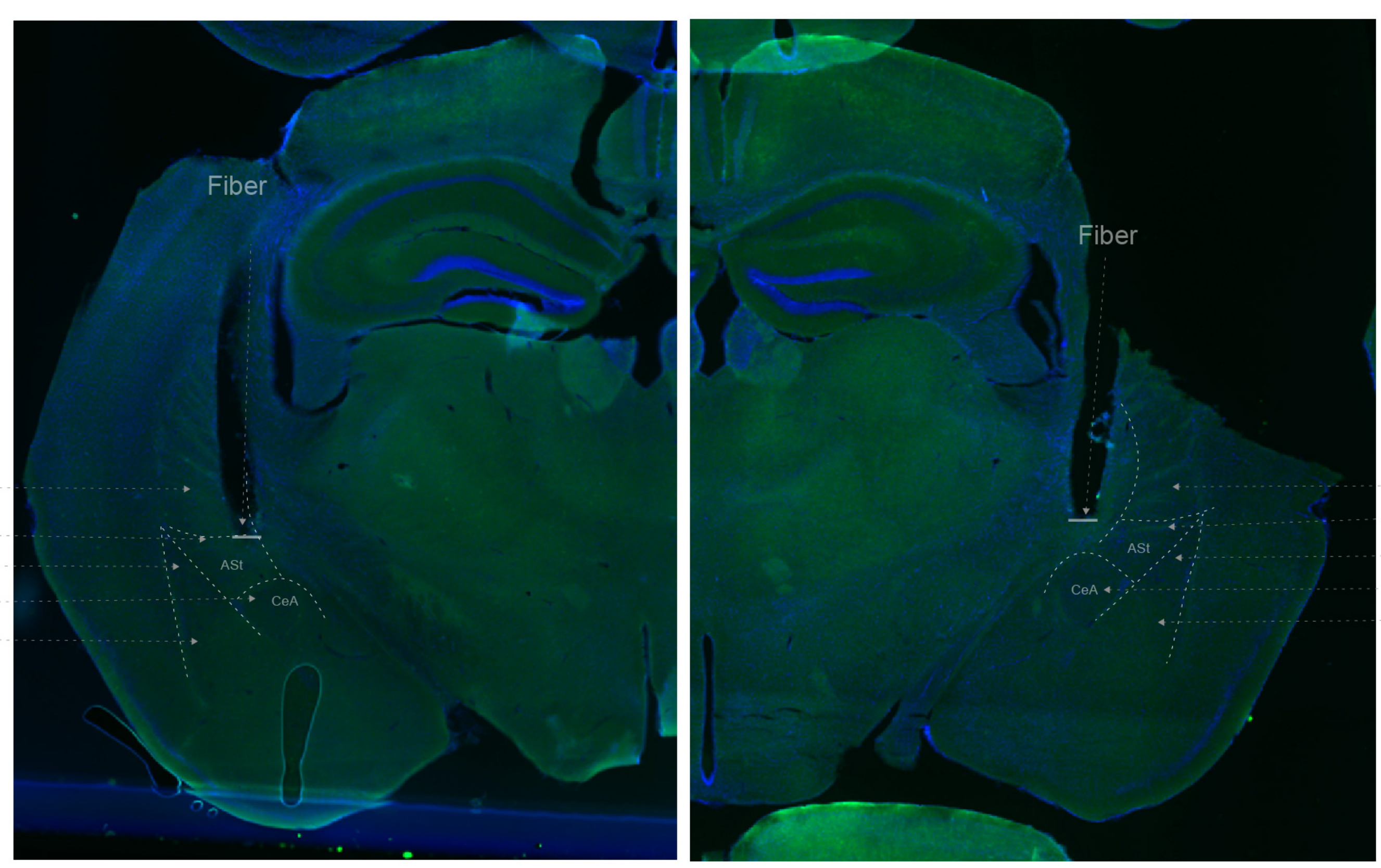

TS: Some lateral expression ✓
ASt: No expression ✗ ✓
LA: No expression ✓
CeA: No expression ✓
BLA: No expression ✓

Appendix E - Histology - *Drd1a:Cre* mice, NpHR experiments:

Drd1a:Cre NpHR

Mouse ID: C05K180

Data from this mouse were not included in the study

TS: Low expression
ASt: Good expression
LA: Expression
CeA: Expression
BLA: Expression

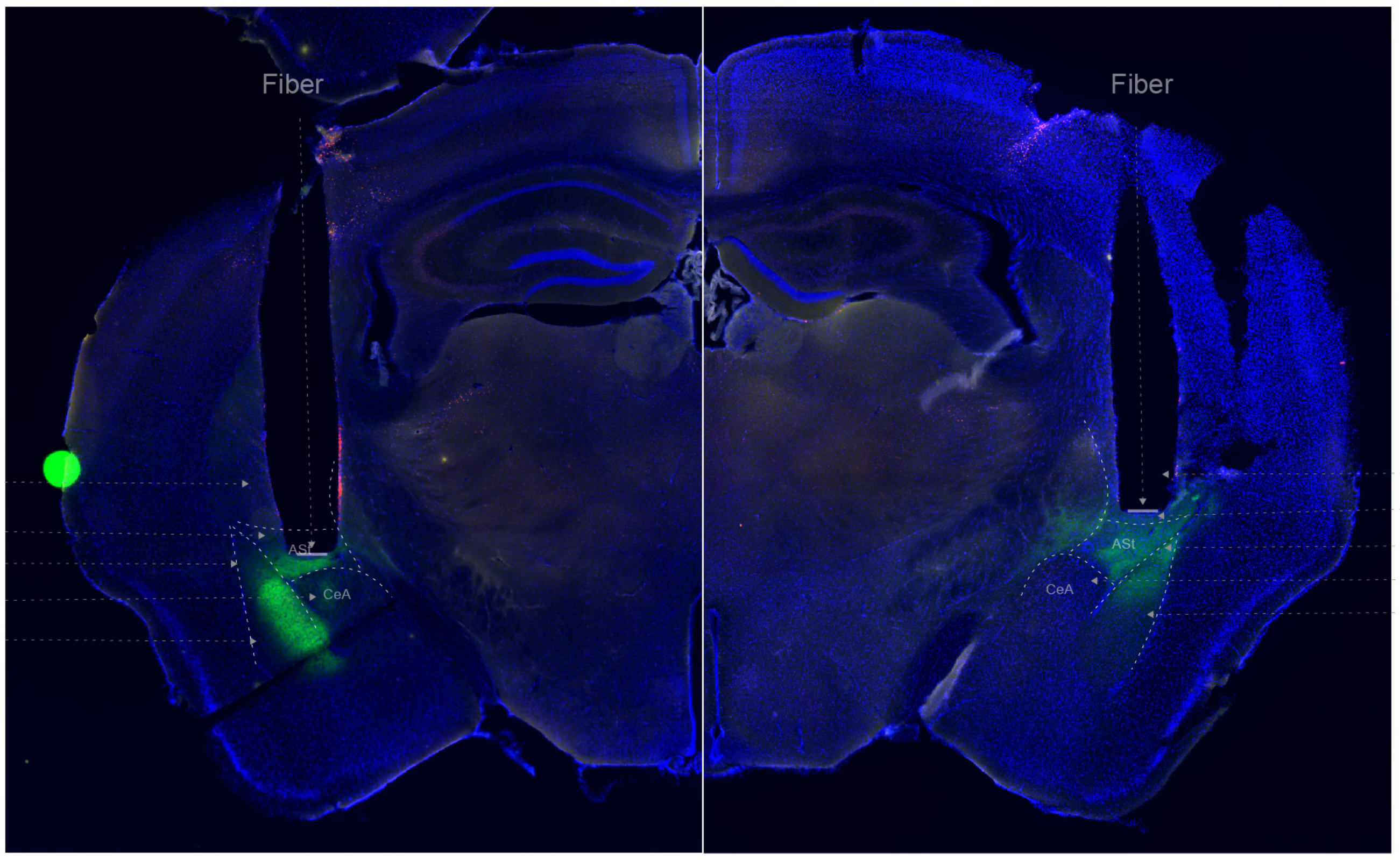

TS: Low expression
ASt: Good expression
LA: Expression
CeA: No expression
BLA: Expression

Drd1a:Cre NpHR

Mouse ID: C05K190

Data from this mouse were not included in the study

TS: Low expression
ASt: Good expression
LA: Expression
CeA: Low Expression
BLA: Expression

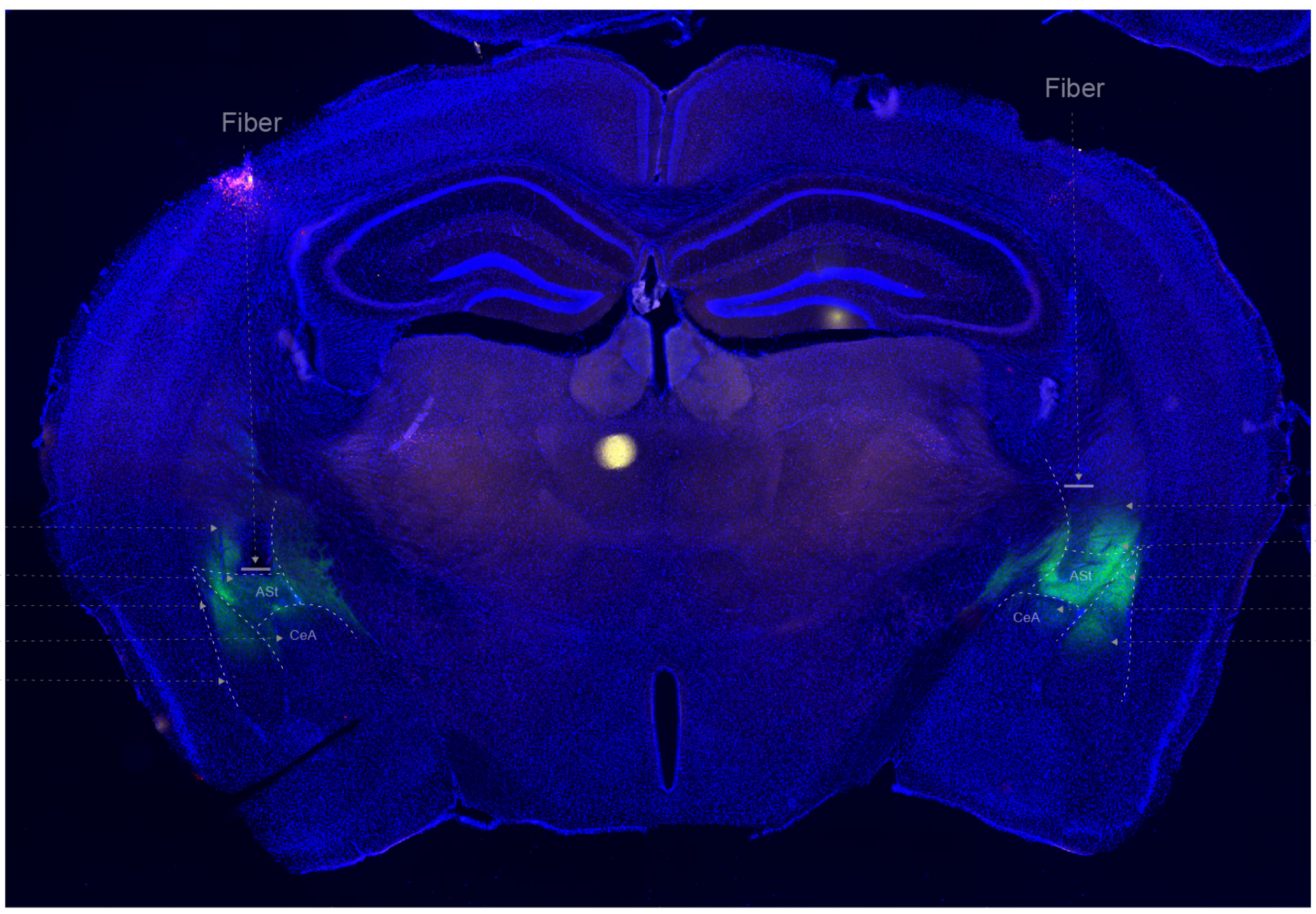

TS: Low expression
ASt: Good expression
LA: Expression
CeA: Low expression
BLA: Expression

Drd1a:Cre NpHR

Mouse ID: C05K171

Data from this mouse were not included in the study

TS: Low expression
ASt: Good expression
LA: Expression
CeA: Expression
BLA: Low Expression

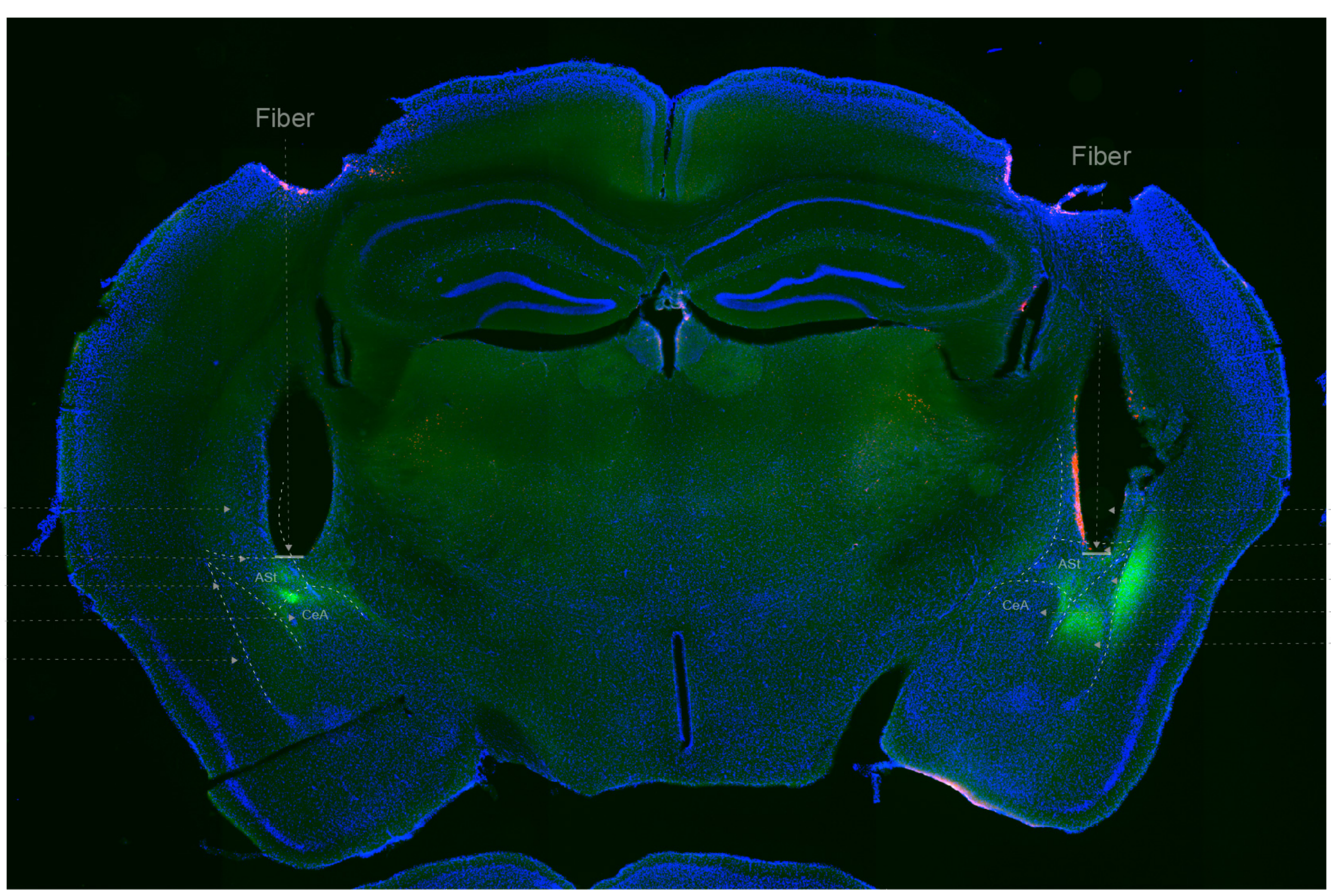

TS: Low expression
ASt: Good expression
LA: Expression
CeA: Low expression
BLA: Expression

Appendix E - Histology - *Drd1a:Cre* mice, NpHR experiments:

Drd1a:Cre NpHR

Mouse ID: C05K162

Data from this mouse were not included in the study

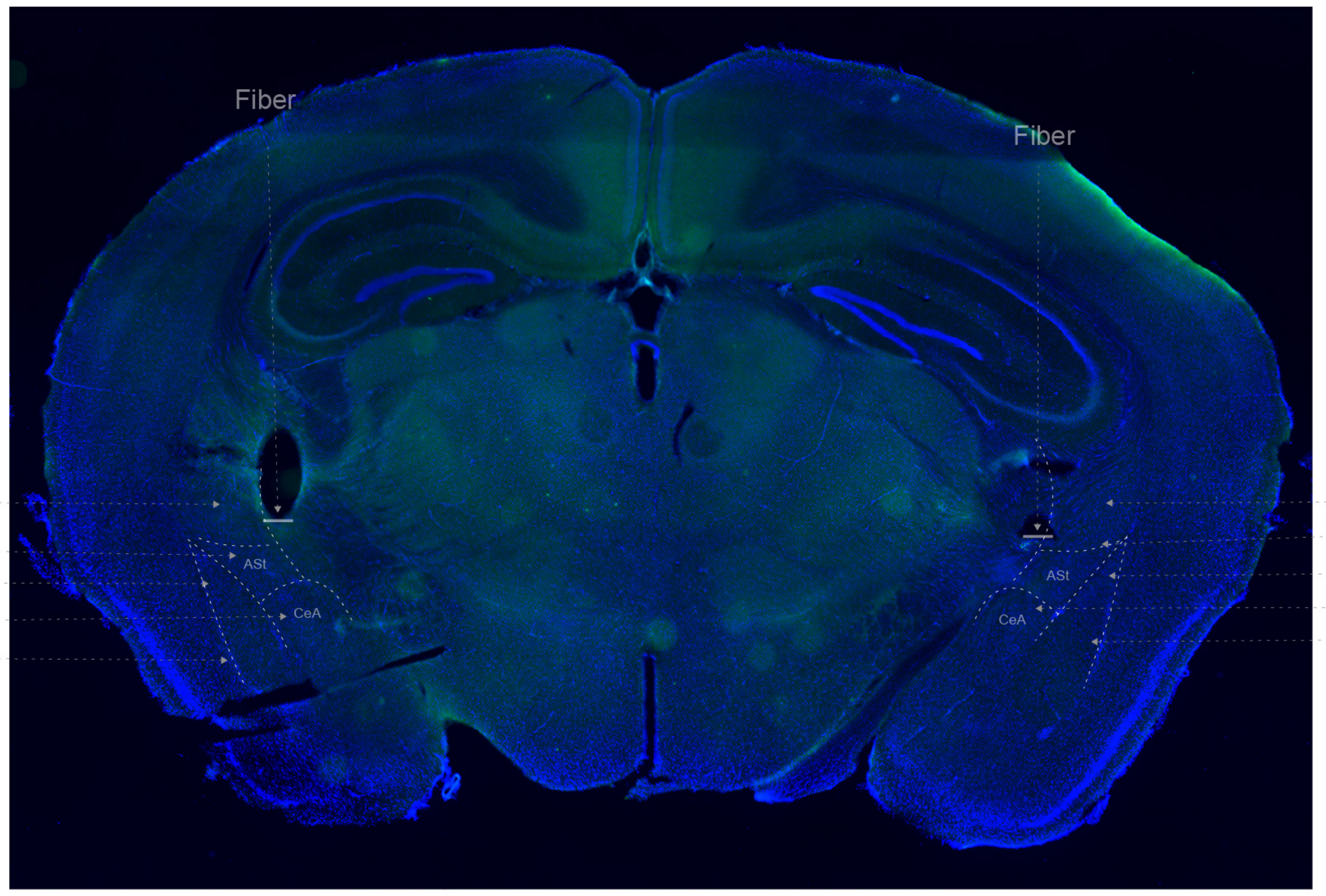

Drd1a:Cre NpHR

Mouse ID: C05K163

Data from this mouse were not included in the study

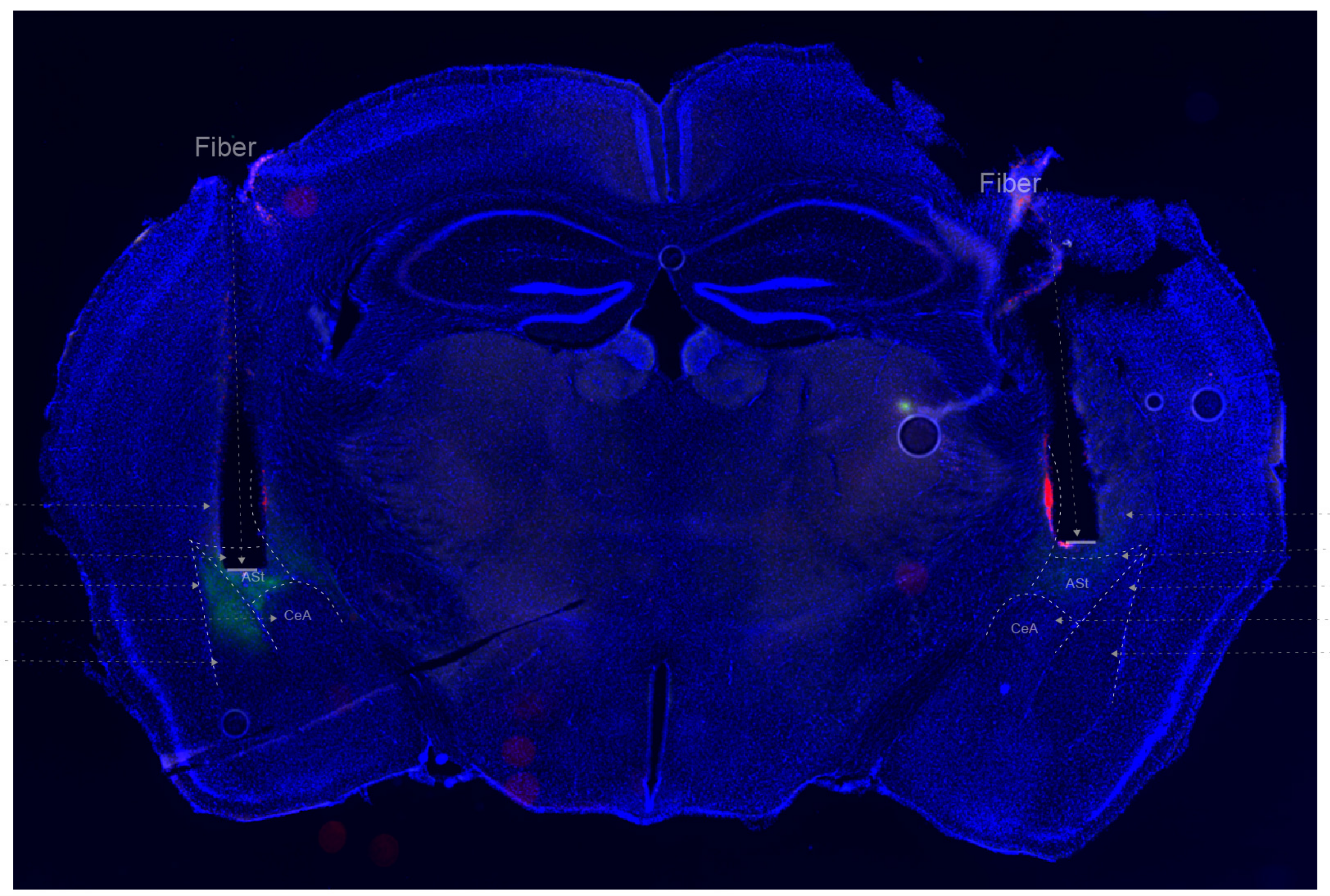

Appendix E - Histology - *Drd1a:Cre* mice, NpHR experiments:

Drd1a:Cre NpHR — Mouse ID: C01I5

Histology okay, Mouse included

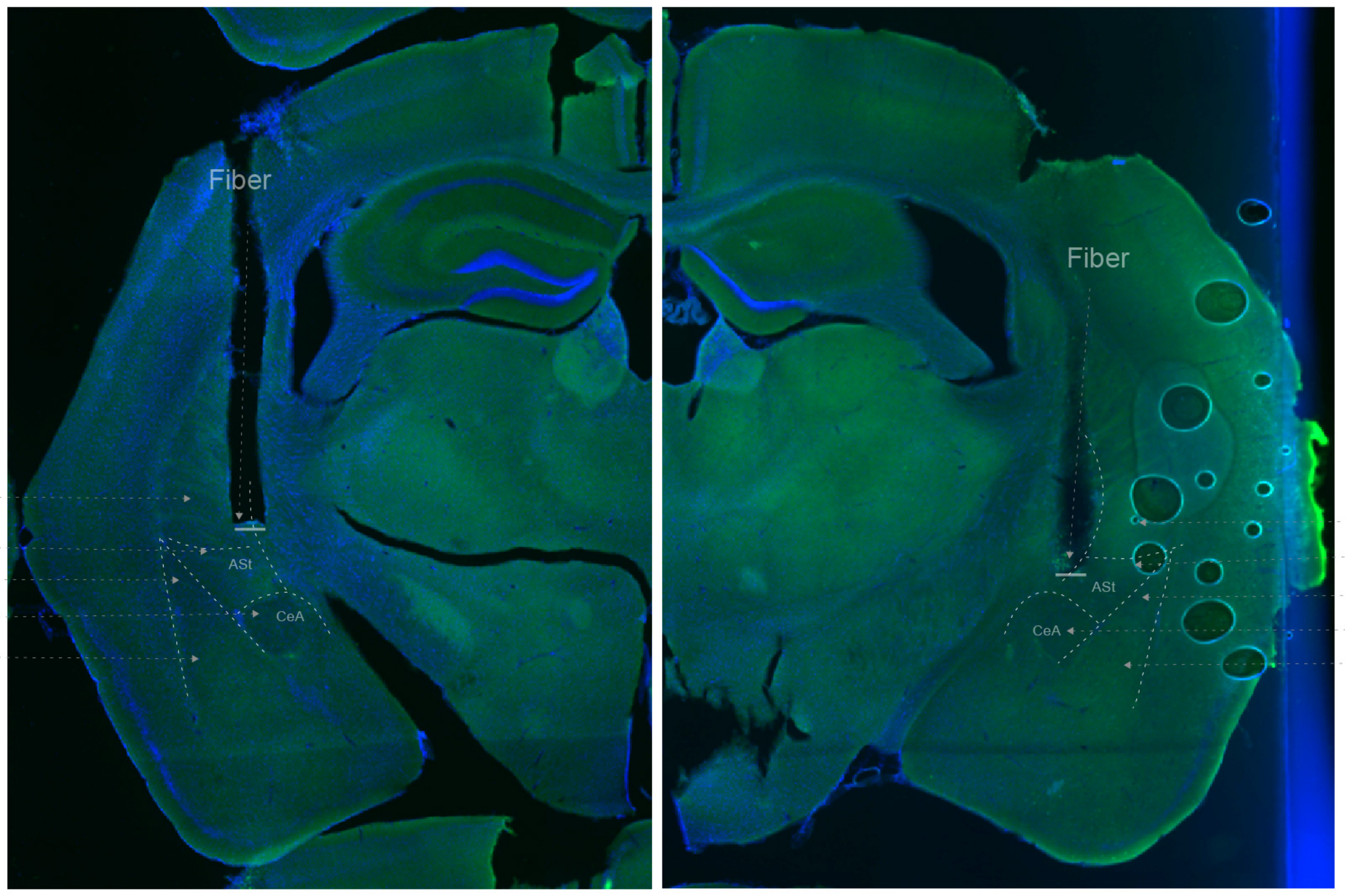

✓ TS: Low expression
✓ ASt: Good expression
✓ LA: No expression
✓ CeA: No expression
✓ BLA: No expression

TS: Some lateral expression ✓
ASt: Weak expression ✓
LA: No expression ✓
CeA: No expression ✓
BLA: No expression ✓

Drd1a:Cre NpHR — Mouse ID: C01I8

Histology okay, Mouse included

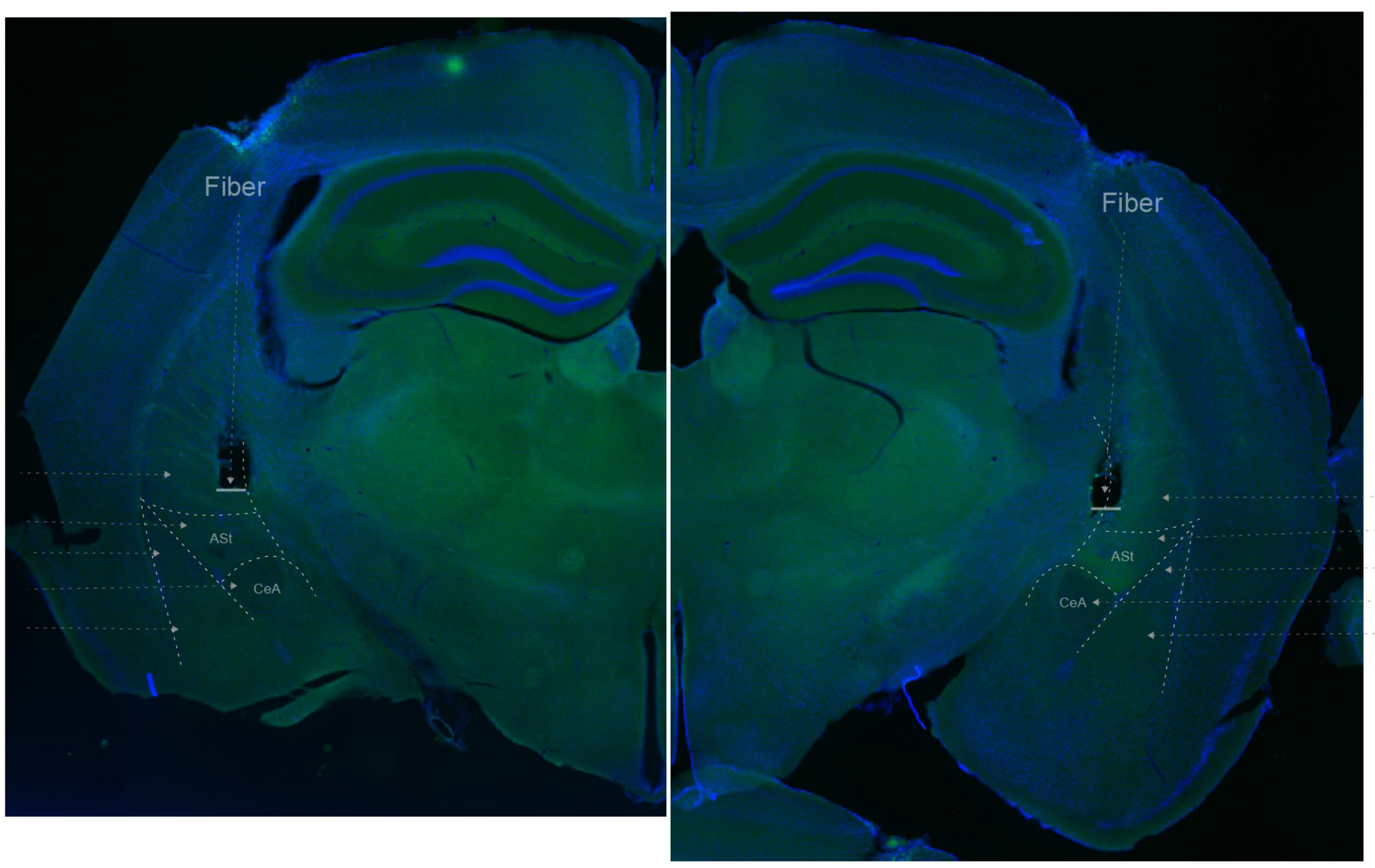

✓ TS: Low expression
✓ ASt: Low expression
✓ LA: No expression
✓ CeA: No expression
✓ BLA: No expression

TS: Some expression ✓
ASt: Good expression ✓
LA: No expression ✓
CeA: No expression ✓
BLA: No expression ✓

Drd1a:Cre NpHR — Mouse ID: C036644K

Histology okay, Mouse included

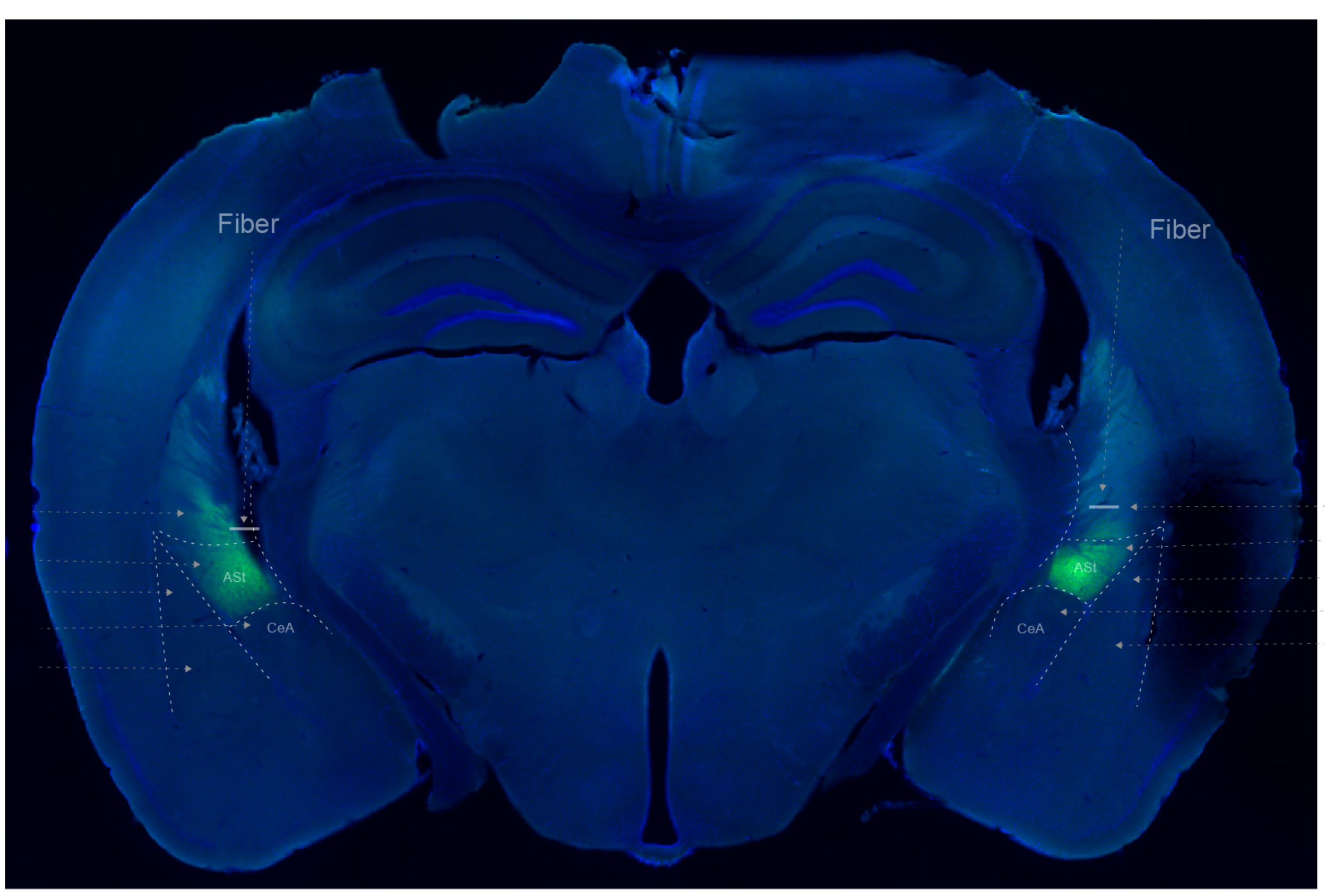

✓ TS: Low expression
✓ ASt: Good expression
✓ LA: No expression
✓ CeA: No expression
✓ BLA: No expression

TS: Some expression ✓
ASt: Good expression ✓
LA: No expression ✓
CeA: No expression ✓
BLA: No expression ✓

Appendix E - Histology - *Drd1a:Cre* mice, NpHR experiments:

Drd1a:Cre NpHR

Mouse ID: C036629

Histology okay, Mouse included

✓ TS: Low expression
✓ ASt: Low expression
✓ LA: No expression
✓ CeA: No expression
✓ BLA: No expression

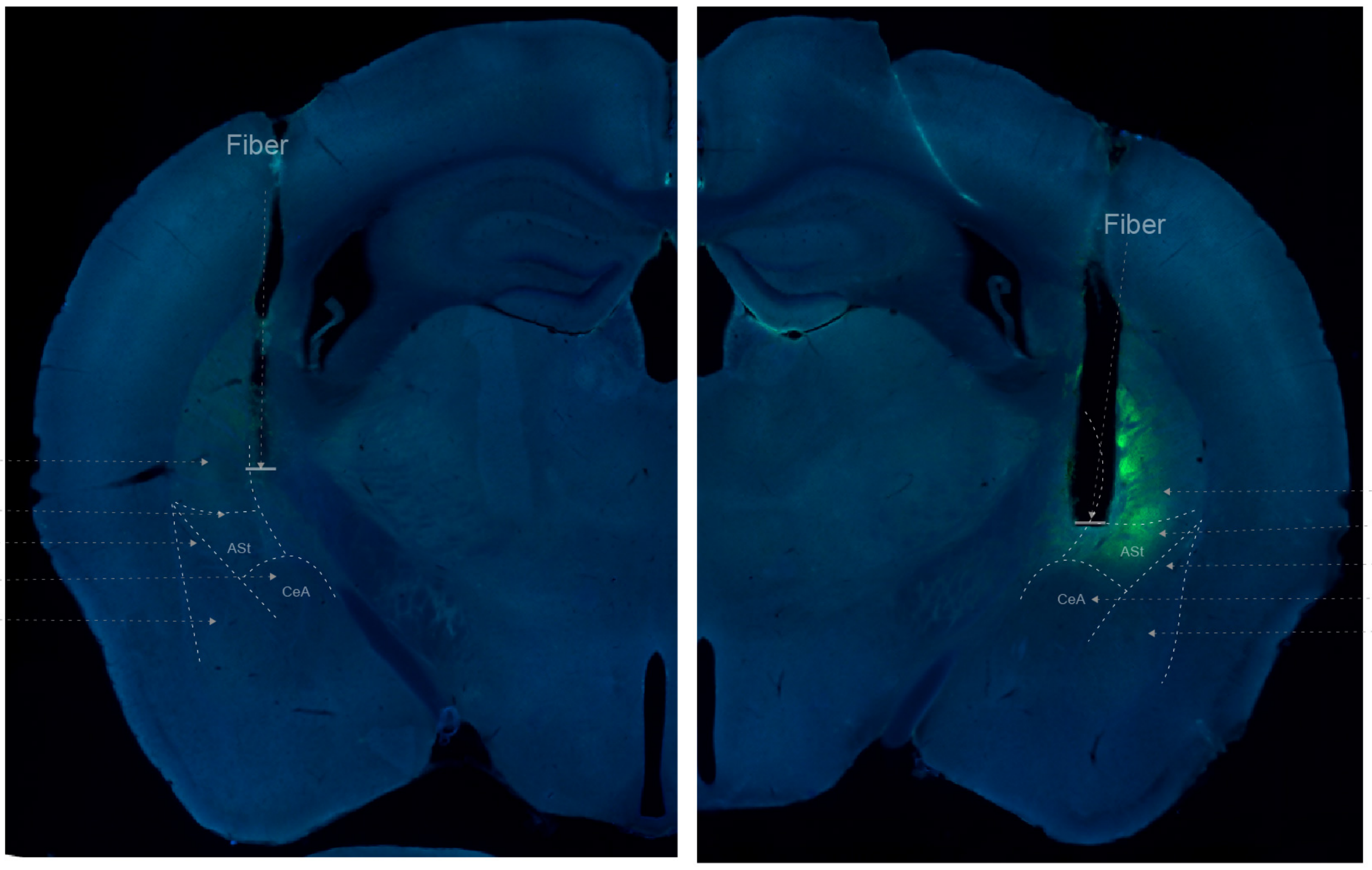

TS: Some expression ✓
ASt: Good expression ✓
LA: No expression ✓
CeA: No expression ✓
BLA: No expression ✓

Drd1a:Cre NpHR

Mouse ID: C05K165

Histology okay, Mouse included

✓ TS: Low expression
✓ ASt: Good expression
✓ LA: No expression
✓ CeA: No expression
✓ BLA: No expression

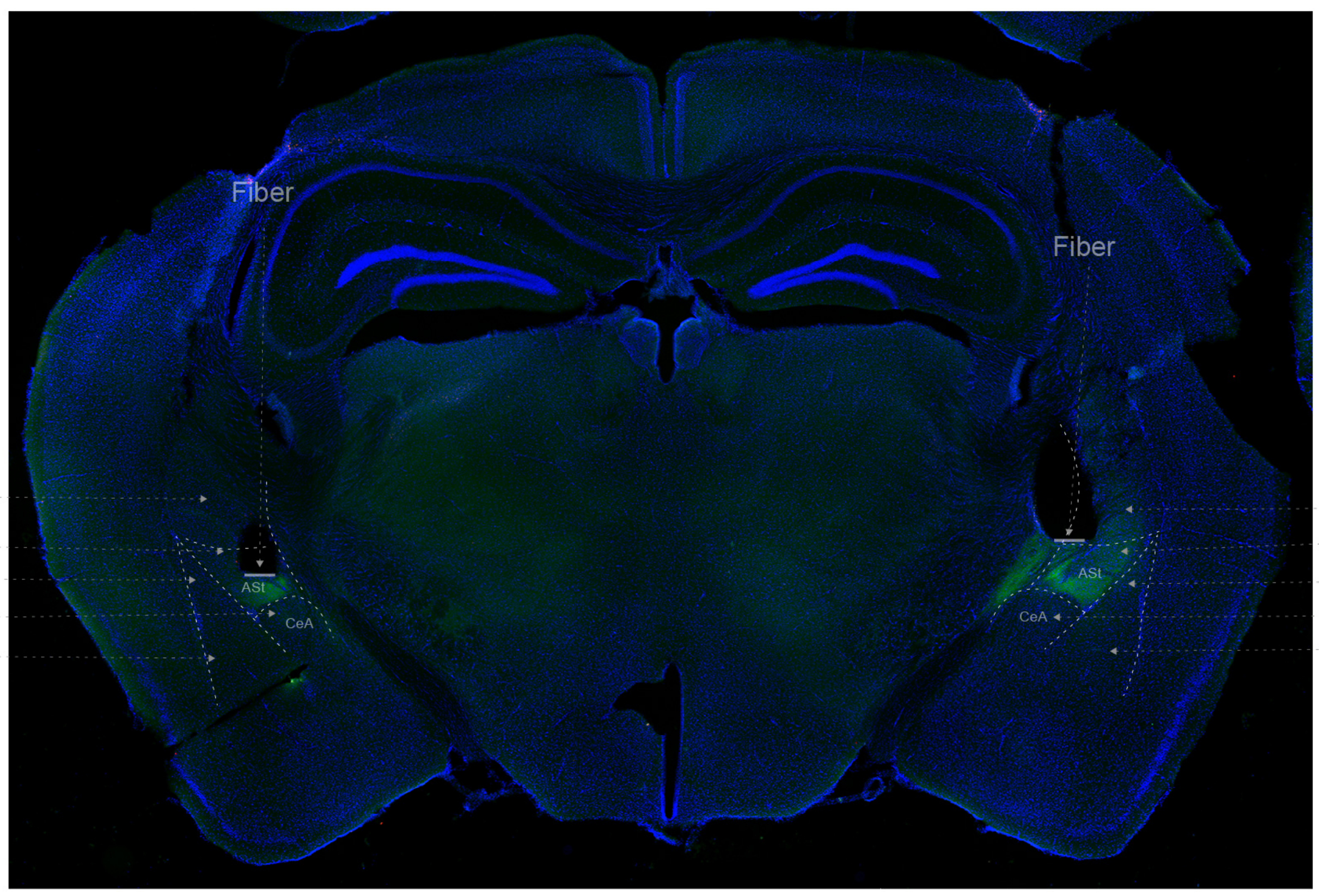

TS: Some lateral expression ✓
ASt: Good expression ✓
LA: No expression ✓
CeA: No expression ✓
BLA: No expression ✓

Drd1a:Cre NpHR

Mouse ID: C05K165

Histology okay, Mouse included

✓ TS: Low expression
✓ ASt: Good expression
✓ LA: No expression
✓ CeA: No expression
✓ BLA: No expression

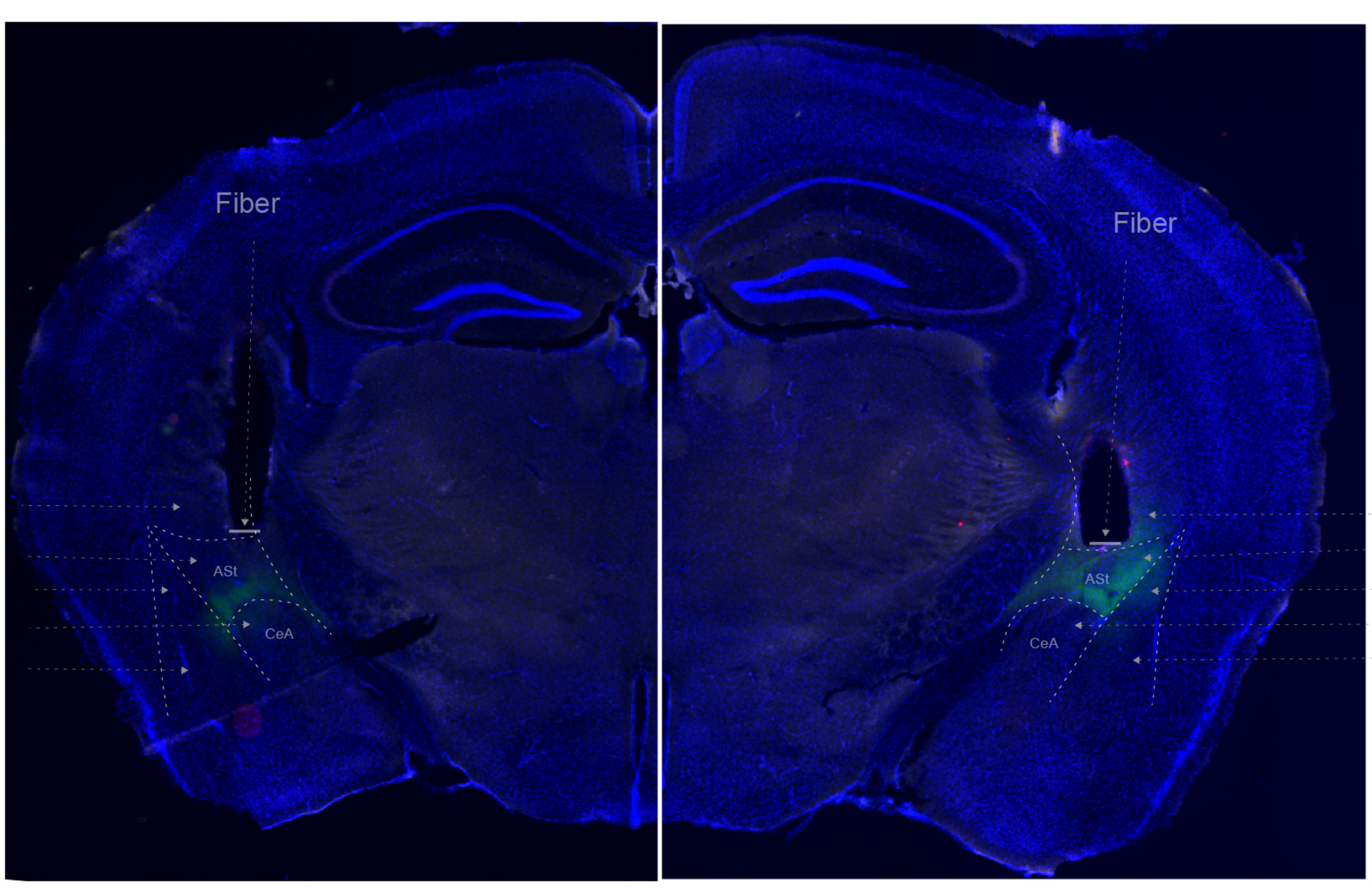

TS: Some expression ✓
ASt: Good expression ✓
LA: No expression ✓
CeA: No expression ✓
BLA: No expression ✓
